# Quantifying Evidence for Competing Biomedical Hypotheses using Large Language Models and Bayesian Analysis

**DOI:** 10.64898/2026.06.05.730173

**Published:** 2026-06-07

**Authors:** Bethany M. Moore, Jack Freeman, Robert J. Millikin, Chitrasen Mohanty, Kevin Shine George, Aviral Bal, Cannon Lock, John-Demian Sauer, Megan E. Spurgeon, Darcie L. Moore, Brittany G. Travers, Ron Stewart

**Affiliations:** 1 Morgridge Institute for Research; 2 Department of Medical Microbiology and Immunology, University of Wisconsin-Madison School of Medicine and Public Health,; 3 McArdle Laboratory for Cancer Research, Department of Oncology, University of Wisconsin School of Medicine and Public Health; 4 John W. and Jeanne M. Rowe Center for Research in Virology; 5 Department of Neuroscience, University of Wisconsin-Madison School of Medicine and Public Health; 6 Occupational Therapy Program, Department of Kinesiology, University of Wisconsin-Madison; 7 Waisman Center, University of Wisconsin-Madison

## Abstract

Science fundamentally depends on the generation and testing of hypotheses, many of them controversial. An explosion in scientific literature has made evaluating hypotheses even within a domain a problem of scale, and risks slowing an already extensive consensus-building process. While this challenge has prompted interest in automated hypothesis evaluation tools, existing methods have not yet proven effective for comparing hypotheses. Here, we introduce KM-GPT-DCH, an algorithm that combines co-occurrence methods with large language models (LLMs) to develop a transparent and reproducible literature-based algorithm to compare controversial hypotheses using a structured scoring approach with Bayesian methods to estimate confidence. When testing the algorithm on historical controversial hypotheses previously decided, KM-GPT-DCH chooses the correct hypothesis with high confidence several years before the scientific community or public do so. We further apply the algorithm to compare twenty unresolved controversial hypothesis pairs providing guidance for future research. The method can help researchers and the public to evaluate biomedical hypotheses such as “Is it more likely that monoamine deficiency or inflammation causes depression?” It can also be used to assess and visualize historical trends in the scientific literature. A web-based implementation of the algorithm is freely available at https://skim.morgridge.org.

## Introduction

Gathering and assessing evidence for and against a hypothesis has been an essential step in the scientific method for centuries. This process typically involves surveying the published literature to compile relevant findings and may also require conducting new experiments. The resulting body of evidence is then synthesized with domain knowledge to assess the plausibility of the hypothesis. Hypothesis evaluation can thus be viewed as resembling a Bayesian process^1^ (a resemblance inspiring some of the methodology here), where prior beliefs about a hypothesis, informed by domain expertise, are updated in proportion to the strength of the newly gathered evidence (the likelihood) to get a prediction about the hypothesis (the posterior). However, as the volume of scientific literature continues to grow at an accelerating pace^2^, it is difficult for even domain experts to maintain a comprehensive awareness of the available evidence in their own fields. Hypothesis generation algorithms present an additional challenge by producing more hypotheses (including many false positives) than can reasonably be evaluated^3–15^. Establishing the credibility of a hypothesis requires some degree of literature support. Years or even decades can pass before a hypothesis becomes generally accepted or rejected^16^, over a different competing hypothesis.

For all these reasons, computational methods to automate the search, evaluation, and summarization of the literature have become increasingly important. These methods may use co-occurrence models, knowledge graphs, literature mining, natural language processing, neural network analysis and other emerging techniques to evaluate articles and hypotheses on a large scale^17–21^. Large language models (LLMs) are an attractive avenue for automated hypothesis evaluation because they can parse literature at a scale that far exceeds the capacity of individual researchers. However, they suffer from important limitations: 1) fabrication of plausible-sounding but incorrect information (“hallucination”)^22^, 2) lack of transparency in the evidence used to arrive at their conclusions^23^, 3) non-peer-reviewed training data (*e.g*., social media misinformation), which compromises scientific provenance^23,24^, 4) inability to perform temporal filtering (where the model is required to only use data from a particular time period) due to leakage from the model’s parametric knowledge^25,26^, and 5) the inability to reference out-of-training documents^23^.

Some of these problems can be alleviated by retrieval-augmented generation (RAG), a technique that first retrieves documents and then provides them to the LLM to evaluate the hypothesis or claim in question^27,28^. Recent RAG-based tools aimed at aiding in hypothesis evaluation include^29^, SKiM-GPT^30^, ValSci^31^, and proprietary systems such as Scite^32^, Elicit (https://elicit.com/), and Consensus (https://consensus.app/). Tools like ValSci and OpenScholar focus primarily on claim verification, where LLMs query a database or search for papers that may support or refute a single statement. The proprietary Consensus tool uses a “Consensus Meter” to provide an aggregate distribution of supporting versus contesting evidence, yet this remains a statistical summary of search results rather than a comparison of two distinct theories. Similarly, Elicit excels at automated data extraction through organizing study characteristics into tables to assist in systematic reviews, but it leaves the higher-level task of weighing competing hypotheses to the human researcher. Scite offers citation context by identifying whether a paper is supported or contrasted, but it operates at the metadata level, lacking a framework to evaluate the underlying scientific validity of one hypothesis relative to another. This reliance on evaluating hypotheses in isolation tends to lead to score shrinkage (where individual evaluations tend to get similar scores) and thus the inability to effectively rank cases^33–35^.

None of these tools harness the fact that LLMs (like humans) are most effective when given a comparative task^33–36^. Direct pairwise comparisons tend to force the LLM to identify differences and lead to higher resolution and better alignment with human judgement^37–39^. Thus, giving an LLM a task to compare two hypotheses can result in a more accurate evaluation than examining a hypothesis in isolation. To our knowledge, there is no currently available automated tool that directly weighs the evidence of two competing hypotheses against each other.

Providing a score when evaluating hypotheses, as well as a confidence level for this score, is also challenging. Existing methods to estimate the confidence of an LLM fall mainly into the categories of verbalization, sequence probability, surrogate token, consistency, trained probing, and Bayesian methods^40^, but many depend on log probabilities or hidden layers that are not readily available from proprietary models, and are not implemented in a comparative way (see Supplemental Discussion). Furthermore, because of coarse retrieval mechanisms and a lack of time-stamping of training data, most hypothesis evaluation tools cannot perform temporal filtering of the literature, meaning they lack the ability to evaluate hypotheses across specific, discrete time intervals. This creates a significant gap in the evaluation of hypotheses over time, as these systems cannot analyze how the weight of evidence changes over time. Consequently, there remains no tool that provides a structured, evidence-weighted, and temporally aware methodology for the comparison of user-generated hypotheses. Here, we extend SKiM-GPT, our previous algorithm based on both co-occurrence and LLM-backed reasoning^30^. While SKiM-GPT is effective in evaluating a single hypothesis, it is not designed to compare two competing hypotheses. Our new algorithm, “KinderMiner and Generative Pretrained Transformers with Direct Comparison of Hypotheses” (KM-GPT-DCH), modifies the SKiM-GPT approach by directly comparing competing hypotheses to determine which hypothesis is more likely. The KM-GPT-DCH system provides a parameterized prompt template and retrieves documents as input to the LLM. This enables a reproducible, comparative evaluation of the hypotheses, in contrast to a chat-based dialogue.

Our approach generates an evidence-weighted score assessing which of the two hypotheses has greater support within the retrieved literature. By combining co-occurrence modeling on PubMed abstracts with RAG analysis and a structured output, we ameliorate the issues related to LLM-only approaches discussed above. Additionally, a Bayesian credible interval on this score is computed using a combination of multiple scores from model iterations and the number of retrieved documents supporting each hypothesis. Finally, we implement temporal filtering, showing we can effectively evaluate specific former time periods without leakage from the model’s parametric knowledge of newer information. We are unaware of any other tool capable of systematically comparing and scoring hypotheses to determine the most probable one, while also providing credible interval estimates in a transparent, reliable, and structured literature-based framework.

Historical controversial hypotheses provide apt (and fascinating) proof-of-concept testbeds for this capability. We evaluate the KM-GPT-DCH algorithm on five pairs of historical controversial hypotheses that took up to a decade to be resolved (Table 1). For these hypotheses, we find the KM-GPT-DCH algorithm arrives at the correct decision at least 5 to 12 years before the scientific and public community do so and, in our judgement, provides reasonable evaluations. KM-GPT-DCH provides transparency of reasoning, including complete and validated references from PubMed (unlike the LLM chatbots we compare to). We also apply the algorithm to multiple extant controversial hypotheses that have not yet been firmly accepted or rejected. In many cases, we find that one of the two competing hypotheses is significantly more favored, demonstrating that KM-GPT-DCH can help researchers evaluate recent domain evidence and guide future research directions.

The KM-GPT-DCH algorithm is available as a publicly accessible website (https://skim.morgridge.org) and as an open-source Python package (https://github.com/stewart-lab/skimgpt). The tool enables researchers to conduct systematic reviews, evaluate competing hypotheses, and track historical trends, including through integrated visualization features available to map the evolution of literature over time. It also offers the public a transparent, evidence-based view of scientific consensus.

## Results

### The KM-GPT-DCH algorithm

**Figure 1:**
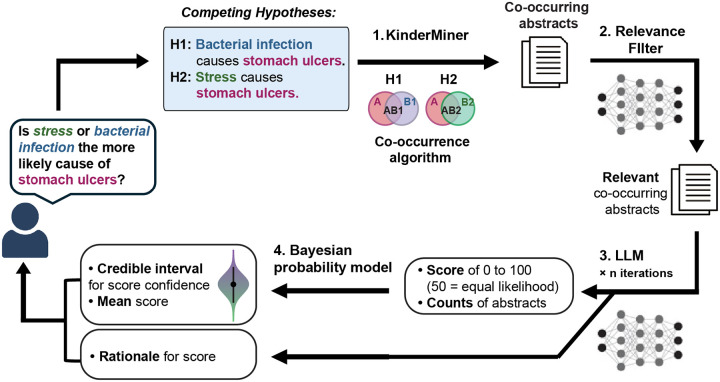
Overview of the KM-GPT-DCH algorithm **KinderMiner**: the user inputs an A term (e.g. “stomach ulcers”) and two B terms (e.g. “stress” and “bacterial infection”) to construct the two competing hypotheses. Optionally, the user can add a time interval to define the retrieval window for co-occurrence. The co-occurrence algorithm (KinderMiner “KM”^41,94^) retrieves PubMed abstracts where the A and B terms within a hypothesis co-occur.**Relevance filter.** The abstracts identified from step 1 above are evaluated for relevance with respect to each user-defined hypothesis through a fine-tuned small language model^30^.**Frontier LLM**. Next, the LLM adjudicates between the two hypotheses using the two relevant literature pools as its sole evidence, a standardized prompt, and output structure. This framework forces a direct comparison of the competing claims. For example, weighing whether stress or bacterial infection is the more probable cause of stomach ulcers requires the model to provide a score and rationale based on the relative strength of the cited retrieved data. See Methods and Freeman et al^30^. The score provided by the LLM ranges from 0 (very strong support for H2) to 100 (very strong support for H1).**Bayesian probability model**. Finally, a Bayesian credible interval (95% credibility) of the score is calculated (see Methods). KM-GPT-DCH: KinderMiner and Generative Pretrained Transformers with Direct Comparison of Hypotheses LLM: Large Language Model H1: Hypothesis 1 H2: Hypothesis 2

KM-GPT-DCH leverages both a co-occurrence algorithm (KinderMiner “KM”^13,41^) and language models (“GPT”) to directly compare two competing hypotheses (“DCH”). Here, we extend KM-GPT, our previous algorithm that evaluates single hypotheses^30^, to directly compare one hypothesis (H1) to a second (H2). The KM-GPT-DCH algorithm has several steps illustrated in Fig. 1, beginning with KinderMiner to obtain PubMed abstracts from co-occurring terms, followed by a relevance filtering step, iterative frontier LLM evaluations of sampled relevant abstracts, and a Bayesian probability model to determine score confidence. KM-GPT-DCH can be run across non-overlapping time retrieval windows to capture how the two hypotheses compare over time.

### Competing historical hypotheses

We used three sets of historical hypotheses to provide a proof of concept for the KM-GPT-DCH algorithm and to compare with other methods: 1) bacterial infection causes peptic ulcers^42,43^, 2) human papillomavirus (HPV) causes cervical cancer^44–46^, and 3) prions cause scrapie^16,47^. Each set has a clear pre-existing hypothesis that was subsequently supplanted by a new hypothesis that took approximately a decade to be accepted. Controversial hypotheses with their proposal date and acceptance or refutation date by the scientific community are listed in Table 1; for full table including public acceptance and refutation dates see Supplementary Table 1. See Supplementary Methods for how the hypothesis acceptance and refutation dates were determined. In the text, unless otherwise specified, acceptance and refutation dates refer to the scientific community acceptance and refutation dates. General public acceptance/refutation dates typically lag behind the scientific community’s dates.

### Insufficiencies in conversational LLMs for direct hypothesis comparison

Because of their widespread use and general usability, we first assessed if chat-based LLM-interfaces could reliably determine if one hypothesis is better supported than another. We tested five commonly used conversational LLM-interfaces and one multi-agent system (Crow) on citation accuracy and whether the correct historical hypothesis could be determined at the appropriate time. For fair analysis of these historical hypotheses, it is necessary to perform temporal filtering (only using references up to a specific date). With temporal filtering, we found that the five conversational LLMs only provided 15% to 25% error-free legitimate references (85% for Crow). All six systems get the correct answer (for the leading hypothesis at a given time) only 67% to 80% of the time. This highlights the unreliability of these systems as well as the inability to be temporally aware. Furthermore, chat-based LLM interfaces do not provide a level of confidence in their answer, nor can they perform hypothesis evaluations at scale. For details on the systems, versions, and methods see Supplementary Discussion; see Supplementary tables 2 and 3 for summarized results and Supplementary Data 1 for all LLM response results.

### Peptic ulcer hypotheses comparison using KM co-occurrence or KM-GPT or KM-GPT-DCH

Traditionally, peptic, gastric, or duodenal ulcer (“peptic ulcer”) was thought to be caused by psychological stress or other factors^48,49^. In 1984, the bacterium *Helicobacter pylori* was implicated in peptic ulcer by Warren and Marshall^42,50,51^. Bacterial infection was not generally accepted as a primary cause of peptic ulcer until 1994 when the National Institutes of Health came to a consensus^52^, with Warren and Marshall later winning the 2005 Nobel prize. (See Supplementary Discussion.)

**Figure 2.**
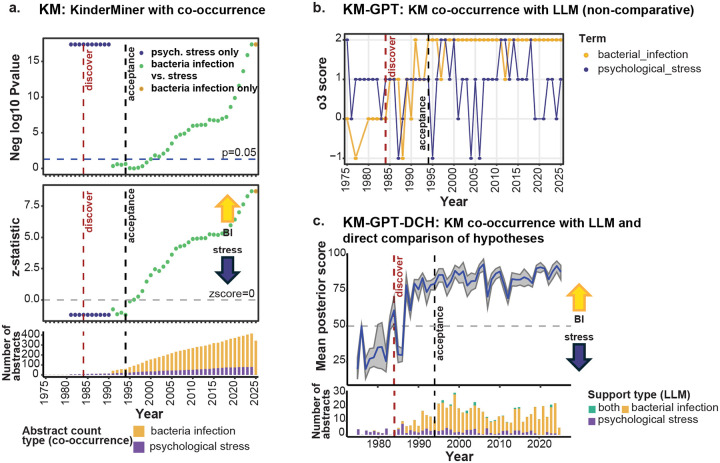
KM, KM-GPT, and KM-GPT-DCH applied to the competing hypotheses for peptic ulcer causation. **a.** KM co-occurrence results for each year between the competing hypotheses for the causation of peptic ulcer: bacterial infection (“BI”) vs. psychological stress (“stress”). The top panel shows the negative log10 of the *p*-value; the middle panel shows the *z-*statistic; the bottom panel shows the number of abstracts obtained for each term. Years range from 1975–2025. In all graphics in Fig. 2, the red vertical line indicates the discovery date for bacterial infection (1984), while the gray line indicates the acceptance date for bacterial infection (1994). **b.** KM-GPT (RAG, non-comparative) results when evaluating the two peptic ulcer hypotheses independently from 1975–2025, where the score from the OpenAI o3 model ranges from −2 to 2, with negative scores refuting the hypothesis and positive scores supporting the hypothesis. **c.** KM-GPT-DCH (RAG, comparative) results. The graph is the mean posterior score from KM-GPT-DCH (iterations=10) where a score above 50 is more supportive of H1 (bacterial infection), a score below 50 is more supportive of H2 (psychological stress), and a score of 50 indicates equivalent or inconclusive support or that no abstracts are available. Only the combined comparative algorithm KM-GPT-DCH successfully identifies bacterial infection as the more likely cause of peptic ulcer years before (1987) general acceptance (1994). A Bayesian credible interval (gray shaded region) for each time point is computed and the 5th and 95th percentile of the posterior is shown (see Methods). The bar plot at the bottom shows the number of abstracts used for the evaluation each year with the color scale designating the number supporting the first hypothesis, the second hypothesis, or both hypotheses. RAG: retrieval augmented generation LLM: large language model KM: KinderMiner co-occurrence algorithm DCH: direct comparison of hypotheses GPT: generative pre-trained transformer o3 score: score from the OpenAI o3 model.

Given that using a straightforward co-occurrence model would be more computationally efficient, we first tried our KM co-occurrence method alone^41^ to investigate these competing hypothetical causes of peptic ulcer. In contrast to both web based LLMs and agentic systems, KM always provides real references and can correctly perform time filtering. Using only KM, however, peptic ulcers were not found to have a stronger association with bacterial infection (compared to psychological stress) until 2001, well after the NIH consensus accepted this idea (Fig. 2a). We concluded that while KM is useful in reliably finding associated abstracts to review, the calculated *p*-values from co-occurrence alone are not sufficient to evaluate competing hypotheses.

We next utilized our RAG-based single hypothesis evaluator, KM-GPT^30^, to evaluate the hypotheses in a non-comparative fashion. When we applied KM-GPT to the two hypothetical causes of peptic ulcer separately (Fig. 2b), the hypotheses had similar scores, revealing that issues like score shrinkage make non-comparative analysis untenable^37–39^. In summary, neither the co-occurrence algorithm KM nor KM-GPT was able to provide early resolution of whether bacterial infection or psychological stress is a more likely cause of peptic ulcer.

It is only when we directly compare the hypotheses of psychological stress and bacterial infection that we can effectively determine the more plausible hypothesis for peptic ulcer causation. The KM-GPT-DCH algorithm performs this direct comparison by using KM to retrieve abstracts which are then provided to a frontier LLM to directly compare evidence for the two hypotheses. The algorithm provides a score from 0 to 100, where above 50 is more supportive of H1 (bacterial infection) and below 50 is more supportive of H2 (psychological stress). A Bayesian credible interval is computed for the score by resampling the LLM’s score with different abstract sets (see Methods). We applied KM-GPT-DCH over time from 1975–2025 to the peptic ulcer case (Fig. 2c). We found that before 1984, the score was mostly below 50, indicating stronger support for psychological stress as the cause of peptic ulcer. From 1987 onwards, the score remained high (75 or higher) indicating strong support for the bacterial infection hypothesis and demonstrating that KM-GPT-DCH resolved the comparison well before the general acceptance of the bacterial infection hypothesis in 1994. Mild support for psychological stress persisted, indicating that while bacterial infection was the stronger hypothesis, there was modest literature support for psychological stress as a risk or complicating factor in peptic ulcer^49^. Additionally, KM-GPT-DCH evaluates a wider range of papers to get a more comprehensive view than other LLM and agentic systems. See Supplementary Discussion for an example of the response and reasoning for the cause of peptic ulcer in 1987. All KM-GPT-DCH results, reasoning, and scores are in Supplemental data 2. The existence of a third cause hypothesized to be a plausible cause for peptic ulcer, NSAIDs, is addressed in the **Discussion**.

### KM-GPT-DCH evaluation of hypotheses about cervical cancer and scrapie

We next directly compare the two hypotheses for the cause of cervical cancer with KM-GPT-DCH run over time from 1975 to 2025. Herpes simplex virus (HSV) was thought to be a potential cause of cervical cancer prior to 1983, as HSV was highly correlated with carcinoma of the cervix^53^. Human papillomavirus (HPV) was proposed to be the cause of cervical cancer in 1983 by Harald zur Hausen and colleagues^44,54^. However, the HPV hypothesis was not generally accepted until 1996, when the National Institutes of Health came to a consensus on the primary cause of cervical cancer^55^. Dr. zur Hausen won the Nobel Prize in 2008.

**Figure 3.**
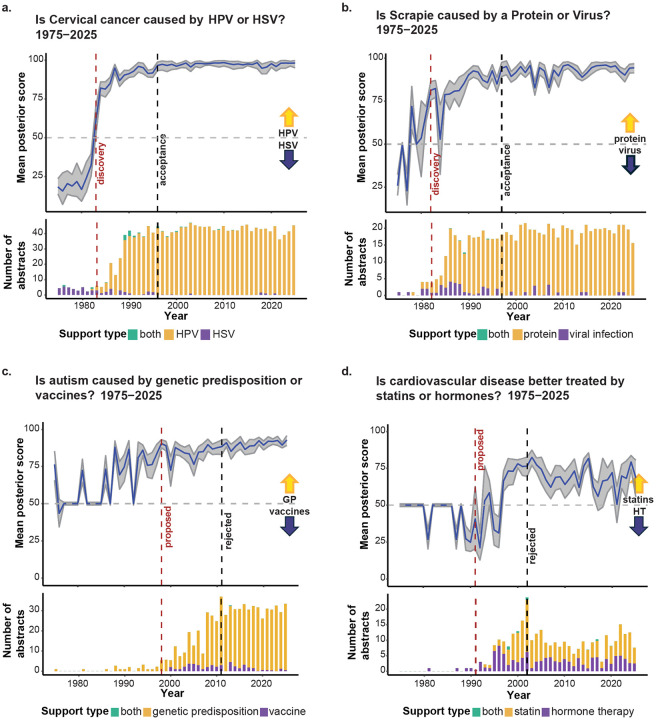
KM-GPT-DCH over time results on four historically controversial hypotheses. KM-GPT-DCH (iterations=10) comparisons on four controversial hypotheses from 1975 to 2025. **For a to d:** The graph is the mean posterior score where a score from 51–100 is more supportive of H1, a score from 0–49 is more supportive of H2, and a score of 50 indicates inconclusive or insufficient information. The gray shaded region is the Bayesian 95% credible interval of the posterior for each time point (see Methods). The bar plot at the bottom shows the number of abstracts used for the evaluation each year with the color scale designating the number supporting the first hypothesis, the second hypothesis, or both hypotheses. Discovery and acceptance dates are based on literature review (see Supplementary Methods) **a.** Cervical cancer controversy where the support for the Human papilloma virus (HPV) hypothesis is a score above 50 and support for the Herpes simplex virus (HSV) hypothesis is below 50. **b.** Scrapie controversy where the support for the protein causal hypothesis is a score above 50 and support for the virus causal hypothesis is below 50. **c.** Autism controversy where a score above 50 is more supportive of genetic predisposition (GP) as a cause, and a score below 50 is more supportive of vaccines as a cause. The ‘propose’ x-intercept line indicates when autism was proposed to be caused by vaccines by the 1998 Wakefield paper and the ‘reject’ x-intercept line indicates when the autism/vaccine hypothesis was rejected by the retraction of the Wakefield paper in 2010. **d.** Atherosclerotic cardiovascular disease (ASCVD) controversy where a score above 50 is supportive of statins as a preventive treatment, while a score below 50 is supportive of hormone therapy (HT) as a preventive treatment. The ‘propose’ x-intercept line indicates when HT was proposed to prevent ASCVD, the ‘reject’ x-intercept line indicates when the ASCVD/HT hypothesis was rejected by a large, randomized trial. H1: Hypothesis 1 H2: Hypothesis 2 KM: KinderMiner co-occurrence algorithm DCH: direct comparison of hypotheses GPT: generative pre-trained transformer

The results shown in Fig. 3a indicate that while HSV was the leading cause from 1975–1982 with an average score of 21.9, from 1983 onward, the algorithm favored HPV with average scores between 62 in 1983 to 97 in 2025. In our judgement, the system provides sound reasoning for its scoring based on the abstracts it is given. A score of 82 in 1984, just one year after the landmark paper was published, shows that KM-GPT-DCH detects what will be the prevailing hypothesis (HPV) 12 years before this is formally accepted as true (in 1996). Additionally, the credible interval, which is based both on the evaluation scores as well as the number of abstracts supporting each hypothesis (see Methods), is wider earlier on (1975–1982), indicating more score variability and less abstract support; but this narrows in later years, indicating an increase in both consistent scoring and number of abstracts (Fig. 3a).

Importantly, a score favoring HSV in 1975 indicates that the system’s evaluation is based on the retrieved texts and not its training data (which contains the fact that HPV is the cause of cervical cancer). Thus, not only does the KM-GPT-DCH method support the prevailing hypothesis for a given time but also gives plausible reasoning solely on the time period’s abstracts. In other words, KM-GPT-DCH mitigates “parametric memory leakage”, as does the SKiM-GPT algorithm on which KM-GPT-DCH is based^30^ .

The third historical set of competing hypotheses involves the causal agent of scrapie (Fig. 3b). Scrapie is a fatal neurodegenerative disease in sheep and goats that takes years to develop. Originally it was thought to be caused by a slow virus^56^, but in 1982 scrapie was proposed to be caused by a protein infection (“prions”)^57^. This proposal was not generally accepted until the mid 1990s^58^, with its proponent Prusiner winning the Nobel Prize in 1997. It is now known that scrapie is caused by a misfolded protein that causes other native versions of the protein to misfold, but this was considered a radical idea at the time as it was thought that causal agents must have nucleic acids^59^.

For the potential causes of scrapie, KM-GPT-DCH scores from 1975 to 1977 were generally supportive of viral infection as the leading cause, albeit with very few abstracts. Between 1978–1981 support for protein infection grew, but again with very few abstracts, leading to wide credible intervals. This support for the protein hypothesis occurred before the publication of the landmark paper in 1982 and is based on a study showing that the scrapie “agent” was not inactivated after exposure to UV light wavelengths that should degrade DNA and RNA, thus suggesting that the agent is not a nucleic acid^60^. Scores varied between 1982 and 1984 but are generally supportive of the protein hypothesis. From 1985 onwards, scores clearly favored the protein hypothesis (with scores in the 70–97 range indicating strong support for the protein hypothesis), well before the general acceptance date of 1997.

For all three sets of controversial hypotheses discussed so far (for the diseases of peptic ulcer, cervical cancer, and scrapie), KM-GPT-DCH was able to detect the correct hypothesis 7 to 12 years before it was accepted by the scientific community (Table 1).

### Evaluating new hypotheses that were refuted using KM-GPT-DCH

To show that our algorithm does not just pick the most recent hypothesis or that it can disagree with a hypothesis, we next evaluated two sets of hypotheses that were later refuted. The first example involves the hypothesis that the MMR (measles, mumps, rubella) vaccine caused autism (first published in 1998), which took more than a decade to be fully refuted and retracted in 2010^61^ (Table 1). KM-GPT-DCH showed that genetic predisposition was a more likely cause than vaccines for autism from 1993 to 2025 (with pre-1993 having few or no abstracts available, Fig. 3c). After the year 2000, the mean score per year was above 75 indicating strong support for a genetic cause rather than a vaccine cause. The algorithm never supports vaccines as a cause of autism when evaluating the evidence found in abstracts during the years of the controversy (1998–2011) and thus arrives at the correct conclusion 12 years ahead of the retraction. This conforms to evidence that suggests that autism is a neuropsychiatric condition with one of the highest rates of genetic heritability, estimated between 50 to 85%^62–65^.

With the next case we show that KM-GPT-DCH can also choose the more reliable treatment between two hypotheses that change over time. The second case involves the use of statins or menopausal hormone replacement therapy (HRT) to reduce atherosclerotic cardiovascular disease (ASCVD) risk. Prior to modern ASCVD reduction therapies (e.g. statins), clinical equipoise existed over whether hormone replacement therapy could reduce ASCVD risk^66^. However, this idea was subsequently refuted in two large randomized clinical trials: beginning with the Heart and Estrogen/progestin Replacement Study (HERS) in 1998 and more comprehensively in the Women’s Health Initiative (WHI) in 2002–2004^67–70^. Here KM-GPT-DCH reflects the changing knowledge about statin and HRT use for ASCVD prevention. In the early period before the establishment of statins for treatment of LDL-C and reduction of ASCVD risk, HRT was favored as a mechanism to reduce ASCVD risk. However, as statin trials demonstrated clear and beneficial primary and secondary reductions in ASCVD, and the increased risks of ASCVD events were uncovered for those undergoing HRT, statins became dominant (Fig. 3d).

In summary, KM-GPT-DCH can correctly refute hypotheses without support (in the case of autism and vaccines) as well as identify temporal trends in therapeutic approaches (in the case of reducing ASCVD risk with the correct identification of statins as the clear preferred treatment as of 1997, five years before the 2002 refutation of HRT as being useful for preventing or reducing ASCVD risk). See Supplemental Figures for KM (co-occurrence) only results (Supplementary Fig. 1) and KM-GPT non-comparative results (Supplementary Fig. 2) for each pair of hypotheses. See Supplemental Discussion for more information on each of the above hypotheses, LLM reasoning, and example outputs.

### Evaluating a more recent hypothesis and twenty existing controversial hypothesis pairs with KM-GPT-DCH

**Figure 4.**
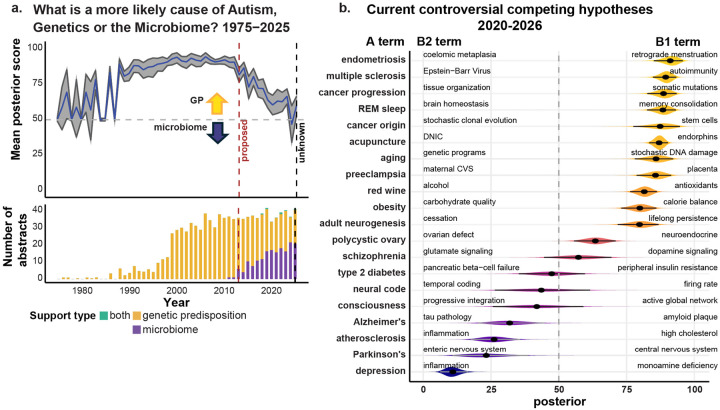
KM-GPT-DCH applied to currently unresolved competing hypotheses. **a.** KM-GPT-DCH (iterations=10) comparisons on the cause of autism, (genetic predisposition (GP) or microbiome), from 1975 to 2025. The graph shows the mean posterior score where a score above 50 is more supportive of H1 (genetic predisposition), a score below 50 is more supportive of H2 (microbiome), and a score of 50 indicates inconclusive or insufficient information. The gray shaded region is the Bayesian 95% credible interval of the posterior for each time point (see Methods). Additionally, the bar plot at the bottom shows the number of abstracts used for the evaluation each year with the color scale designating the number supporting H1, H2, or both hypotheses. Discovery and acceptance dates are based on literature review (see Supplementary Methods). **b.** Violin plots of competing hypotheses that are currently unresolved, where the plots show the KM-GPT-DCH posterior distribution of the scores and the dots in the middle are the mean posterior scores with error bars representing the 95% credible interval. For each set of hypotheses, scores above 50 are more supportive of H1 and the corresponding B1 term is shown close to the 100 mark, scores below 50 are more supportive of H2 and the corresponding B2 term is shown close to the 0 mark, and a score of around 50 when the credible interval line crosses the 50 mark indicates the competing hypotheses remain unresolved. H1: Hypothesis 1 H2: Hypothesis 2 REM: Rapid Eye Movement CVS: Cardiovascular System DNIC: Diffuse Noxious Inhibitory Control

A more recent hypothesis about the cause of autism concerns changes in the microbiome, and unlike the vaccine hypothesis, remains unresolved. In the mid 2000s and early 2010s, studies involving mice and microbiota indicated that the gut microbiome can influence changes in behavior in mice^71,72^. In 2011, a study correlated gastro-intestinal problems and lower levels of certain types of bacteria with autistic children^73^. In addition, short-chain fatty acids, fermentation products of certain bacteria that are associated with autism, were found to induce effects on the brain and alter behavior^74^. Using KM-GPT-DCH, we compared the pre-existing H1 (autism is caused by genetic factors) to the new H2 that autism may be caused by changes in the microbiome. KM-GPT-DCH indicates that from 2013, the score for the microbiome increases (as the genetic predisposition score decreases), but the credible interval becomes wider, and the score never confidently supports the microbiome hypothesis (Fig. 4a). As of now, genetic predisposition is still the more plausible causal hypothesis, but the microbiome hypothesis is perhaps worth considering as an additional factor.

The ideal use case of KM-GPT-DCH is to accelerate consensus-building of existing hypotheses. To this end, in addition to the autism–genetic predisposition–microbiome hypothesis pair, we evaluated an additional twenty pairs of competing hypotheses that are still somewhat controversial using abstracts from 2020 to 2026 (see Fig. 4b for all posterior scores and Supplementary Table 4 for brief descriptions of all hypotheses). Out of these twenty evaluations, four resulted in scores that have credible intervals which overlap with the neutral 50 score line, indicating that neither hypothesis is strongly supported over the other. An example is schizophrenia, a severe mental disorder, where no single cause has been identified. Both dysregulation of dopamine and glutamate signaling pathways have been identified as possible causes^75^. Some studies suggest that both pathways are altered in individuals with the disease^76^.

Sixteen of these sets of hypotheses do not overlap with the neutral line, indicating the existing literature leans towards one of the hypotheses. For example, in the case of the underlying biological cause of depression, the competing hypotheses are that it is due to either monoamine deficiency (H1) or neuroinflammation (H2), and KM-GPT-DCH leans heavily towards neuroinflammation with a mean posterior score of 10.9. In this case, while a few abstracts support the monoamine deficiency cause (or both causes), the majority of abstracts sampled (average 75% across 10 iterations) provide support for persistent inflammation as the main cause of anxiety and depression. Many studies have linked pro-inflammatory cytokines such as interleukin-6 (IL-6), with higher levels of depression^77–79^. Additionally, inflammation may induce suppression of monoamines and thus inflammation may be the primary cause^80^.

Another set of controversial hypotheses that leans toward one hypothesis is whether neurogenesis occurs into adulthood in the human hippocampus. Prior to the 1960s, neurogenesis was thought to end in childhood, but adult hippocampal neurogenesis gained support in the 1980s and 1990s, first through animal models, then in humans^81^. However, a more recent study from 2018 on post-mortem human brains found no evidence of neurons being generated into adulthood in the hippocampus^82^, and since then it has been highly debated. Using our approach targeting papers from 2020 to the present, we found a small number of studies that challenge the view that there is adult neurogenesis. For example, one study found no transcriptomic signatures of neurogenesis in humans compared to other animals, but had a relatively small sample size^83^; another study found that a commonly used marker for newly born neurons, doublecortin (DCX), is expressed in mature neurons throughout the adult brain and does not reliably indicate neurogenesis^84^. Despite this, our algorithm found many studies in support of neurogenesis, including post-mortem immunostaining and single-nucleus RNA sequencing evidence for immature neurons and neural progenitors in adult human brains^85–87^. Overall, our algorithm finds more current papers supportive of adult hippocampal neurogenesis (H1, ~10 times more), than those papers supportive of cessation of hippocampal neurogenesis in adulthood (H2), and gives a high mean score of 79.8 in support of lifelong persistence of hippocampal neurogenesis (Fig. 4b, Supplementary Table 4).

## Discussion

Many papers in PubMed present at least one hypothesis^88,89^. Over 1.5 million papers are added to PubMed each year^90^, so it is impossible for researchers to evaluate each new hypothesis presented in PubMed. Additionally, many hypotheses are controversial and may take years to be either accepted or rejected by the scientific community and general public. Here we describe and implement an algorithm (KM-GPT-DCH) for evaluating and comparing competing hypotheses presented in PubMed over time. We apply it to several historically controversial hypotheses and show that the algorithm can correctly choose between a new controversial hypothesis and a preexisting hypothesis in all five cases tested at least 5–12 years ahead of the scientific and public communities (see Table 1, Supplemental Table 1, Supplementary Methods).

Our algorithm should be of general utility to apply to any pair of hypotheses sufficiently represented in PubMed, to evaluate the relative plausibility of each hypothesis. KM-GPT-DCH provides a score and a credible interval around the score to give the user a measure of uncertainty. We further show that chat-based LLM-interfaces and agentic systems cannot effectively compare hypotheses in a trustworthy manner (see Supplementary Discussion). In contrast, our algorithm reliably cites published literature sources 100% of the time; this, combined with the structured prompt and output, increases trustworthiness, reproducibility and transparency when compared to chat-based LLM systems.

In addition to being useful for comparing hypotheses, KM-GPT-DCH, when applied over time, is useful for surfacing and visualizing historical trends in science. We found instances where the dominant hypothesis was clear (such as HPV being the cause of cervical cancer (Fig. 3a)) and where trends were more nuanced with a complicated history (such as bacterial infection being the cause of peptic ulcer but psychological stress being a contributing factor (Fig. 2c)). KM-GPT-DCH is effectively able to follow each trend with a score and credible interval that aligns with the literature at the time. We found that KM-GPT-DCH is able to refute false hypotheses such as the hypothesis that vaccines cause autism well before it was retracted (Fig. 3c), and correctly reflect changing views of hypotheses over time, such as the cardiovascular impacts of HRT for menopausal symptoms and the clear treatment benefit of statins for ASCVD risk reduction (Fig. 3d). Finally, we have investigated twenty current controversial hypothesis pairs and found that while four hypothesis pairs still do not have a prevailing hypothesis, sixteen hypothesis pairs trend towards a leading hypothesis. Thus, by analyzing trends in literature, KM-GPT-DCH can focus priorities on research topics, aiding in strategic decision-making for research directions, funding, or resource allocation.

KM-GPT-DCH is intended to be used by both scientists and the public. For both audiences, the web-based interface (https://skim.morgridge.org/) allows for users to easily access and use the algorithm. Scientists may find KM-GPT-DCH useful to narrow down sets of hypotheses to test, such as which genes may be more likely to be important in each hypothesis or what biological process or pathway is most likely involved in a certain condition. The public (and scientists curious about other domains) may also be interested in the algorithm for finding quick but reliable literature-based answers to scientific questions. Indeed, the tool offers a somewhat rarely shared opportunity for scientists and non-scientists to query the same literature in an accessible manner. This is an important advantage considering the persistence of controversy among the public even after consensus is built within the scientific community on certain topics – the cause of autism and the potential reasons behind that difference, for example, as addressed in Supplemental Discussion.

The KM-GPT-DCH algorithm has some limitations. First, the KM, KM-GPT, and KM-GPT-DCH algorithms do not currently search the main body of the papers, tables, figures, supplemental materials, or any numerical data. We plan to include some of these other data sources in subsequent updates. Second, when both hypotheses have little literature support, evaluation may be difficult because of low statistical power. Third, the algorithm can only compare two hypotheses at a time. An example of when comparing three or more hypotheses would be useful is the case of peptic ulcer. In the 1990s, it became clear that NSAIDs (non-steroid anti-inflammatory drugs) also can be a primary cause of peptic ulcer^91,92^, and these ulcers can be exacerbated by psychological stress^93^. Thus, there are actually three hypothetical causes for peptic ulcer: psychological stress, bacterial infection, and NSAIDs. Finally, the A terms and B terms used to search for a given hypothesis can impact the results; see Supplementary Methods for guidance on choosing terms.

Despite these limitations, KM-GPT-DCH is a powerful method for comparing two hypotheses in a reliable, reproducible, and transparent manner. In addition, by incorporating temporal windowing and RAG, KM-GPT-DCH can track how literature relationships between entities have changed over time. This capability illuminates emerging trends or shifts in understanding due to major scientific breakthroughs, regulatory approvals, or new clinical guidelines. These advancements provide researchers with an unprecedented ability to not only interpret the past but also forecast future directions, highlighting the tool as a dynamic system for monitoring and shaping the trajectory of biomedical knowledge inquiry.

## Methods

The details of the KM-GPT portion of the KM-GPT-DCH algorithm have been described previously^30^. See Supplementary Methods for more information. The DCH extension to the algorithm is described briefly in **Results**. The model used throughout the paper for the frontier LLM analysis is OpenAI’s o3 model. The relevance filter is based on a fine-tuned version of phi3-mini as described previously^30^.

### KM co-occurrence statistics

For Figure 2a, in which two KM results were compared, the z test of proportions was used to provide a *p*-value: $Z=\frac{\left(p_{1}-p_{2}\right)-0}{\sqrt{p\left(1-p\right)\left(\frac{1}{n_{1}}+\frac{1}{n_{2}}\right)}}$ where p1 is proportion 1 or the ratio of AB counts over B counts for B term 1, p2 is proportion 2, or the ratio of AB counts over B counts for B term 2, and p is the overall sample pooled proportion from both terms. n1 and n2 are the sample sizes for the respective groups. The *z*-score was then compared to the *z*-statistic associated with the given alpha level.

If co-occurring abstracts were only available for one of the two hypotheses, then the *p-*value was set to 1*e*^−300^ (capped) for the hypothesis with abstracts, and 1 for the hypothesis with no co-occurring abstracts. The *z*-statistic was set to the highest value if the hypothesis with the abstracts was the first hypothesis, or the lowest value if the hypothesis with abstracts was the second hypothesis.

### LLM score and statistics

The KM-GPT-DCH algorithm quantifies the strength of evidence between competing hypotheses through a combination of LLM verbalization and number of supporting abstracts using Bayesian statistics. To construct the evidence base for each evaluation, the system retrieves deduplicated PubMed abstracts and applies a stratified random sampling method to select a fixed set of 50 abstracts. This sampling enforces a minimum sampling floor of 6% to ensure that both hypothesis pools are represented even when one is much larger than the other. If the total pool of relevant abstracts exceeds 50, the algorithm utilizes sampling with replacement across 10 iterations to capture different document sets. Additionally, if the total pool of relevant abstracts is greater than 500, we set the iterations to 20 to get a larger abstract window. Because of random sampling with replacement, it is possible (and likely if abstract numbers are large) that a run with the same A and B terms will return different sets of abstracts to evaluate and therefore can change the resulting output. This is why multiple iterations are run. For each of these iterations, the LLM is prompted to provide a score ranging from 0 to 100 based on the abstracts it identifies as supportive for either H1 (represented by a score of 100) or H2 (represented by a score of 0); the specific prompt for this evaluation can be found in the Supplementary Methods.

If we divide the LLM score by 100, then the score range follows a beta distribution’s range (*i.e*., bounded by 0 and 1). If we consider our score a probability, we can use the beta distribution to model the score variation and draw a credible interval; in this case, a distribution towards zero indicates support for H2 while a distribution towards one indicates support for H1. A beta distribution has two parameters, Beta(*α, β*), where *α* is the number of successes (k) and *β* is the number of failures (n-k) with n trials. The mean (*μ*), or expected value, of a beta distribution is $\frac{\alpha }{\alpha +\beta }$, and the variance (*σ*^2^ ) of a beta distribution is given as $\frac{\alpha \beta }{\left(\alpha +\beta +1\right)\cdot {\left(\alpha +\beta \right)}^{2}}$, thus we can solve for *α* and *β* given the mean and variance by rearranging these equations. To get these parameters from the score we use the mean and variance of the 10 iterations with the following formulas: $\alpha =\left(\frac{\left(1-\mu \right)}{\sigma^{2}}-\frac{1}{\mu }\right)\mu^{2}$ and $\beta =\alpha \left(\frac{1}{\mu }-1\right)$. The Beta(*α*, *β*) of these parameters gives us our likelihood distribution.

In Bayesian statistics, prior information can be incorporated into the model. A beta distribution can also be used to model count data. We can therefore model a prior distribution using the beta distribution and our counts of abstracts, where the total number of abstracts is n, the number of abstracts that support H1 (k) is our *α* and our *β* is (n-k), or the number of abstracts that support H2. In the case of abstracts that support both, we divide them by two and add that number to both the *α* and *β*.

Finally, a posterior distribution is made by updating the prior distribution with the likelihood distribution. The alphas and betas from both likelihood and prior are combined to get parameters for a posterior distribution:

$$\text{Beta}\left(\alpha \;\text{posterior},\beta \;\text{posterior}\right)=\text{Beta}\left(\alpha \;\text{Likelihood}+\alpha \;\text{prior},\beta \;\text{likelihood}+\beta \;\text{prior}\right)$$

On this distribution we do an equal-tailed 95% credible interval, where 2.5% of the distribution lies on either side of the limits. Thus, the limits show the 2.5 and 97.5 percentiles. From this posterior distribution we take the mean to get the mean posterior score.

### Criteria for determining whether a hypothesis pair is “resolved”

Here we describe how to determine whether an H1 vs H2 question is resolved using KM-GPT-DCH. This is based on the LLM score and a Bayesian credible interval. For the purposes of this paper, we define a historical hypothesis to be resolved when the score is at least 67 (when H1 is approximately twice as likely than H2) and the confidence interval does not cross the 50-mark line.

### Finding new extant controversial hypothesis pairs

To identify additional controversial hypotheses, we used llm-council (GitHub: karpathy/llm-council). Given a query, llm-council orchestrates a panel of LLMs and a Chair; first the query is passed to the panel LLMs individually, and the responses are collected and anonymized responses are passed to other panel LLMs to rank them in accuracy and insight. The Chair collects all the models’ responses and compiles them. We provided our existing list of curated hypotheses to llm-council and asked to generate a newer set of controversial hypotheses (see Supplementary Table 4). The collected hypotheses were subsequently reviewed manually.

## Supplementary Material

Supplement 1

Supplement 2

Supplement 3

Supplement 4

Supplement 5

Supplement 6

## Figures and Tables

**Table 1.** Table of Controversial Hypotheses that are tested using KM-GPT-DCH

Disease/Condition	New Hypothesis	Existing Hypothesis	Relationship	Propose Date	Accept or Reject	Accept/Reject Date	KM-GPT-DCH Resolution Date	KM-GPT-DCH Ahead of Scientific Resolution Date
**Three Historically Controversial Hypotheses**								
Cervical Cancer	HPV	HSV	Causes	1983	Accept	1996	1984	12 years
Scrapie	Protein infection (Prion)	Viral infection	Causes	1982	Accept	1997	1986	11 years
Peptic Ulcer	Bacterial infection	Psychologic al stress	Causes	1984	Accept	1994	1987	7 years
**Additional Two Controversial Hypotheses**								
Autism	Vaccines	Genetic predisposition	Causes	1998	Reject	partial 2004, full 2011	1998	13 years
Cardiovascular Disease Risk	Hormone Replacement Therapy	Statins	Treats/Reduces	1991	Reject	rejected 2002	1997	5 years

## Data Availability

All code is available through GitHub: https://github.com/stewart-lab/skimgpt
