# Supplementary material for: Quantifying Evidence for Competing Biomedical Hypotheses using Large Language Models and Bayesian Analysis
Source: bioRxiv. 2026 Jun 7:2026.06.05.730173. Preprint. [Version 1] doi: 10.64898/2026.06.05.730173 (PMC13251926; doi:10.64898/2026.06.05.730173)
Supplement: Supplement 1 [file media-1.docx]

# Supplementary information

**Quantifying Evidence for Competing Biomedical Hypotheses using Large Language Models and Bayesian Analysis**

Authors: Bethany M. Moore^1^, Jack Freeman^1^, Robert J. Millikin^1^, Chitrasen Mohanty^1^, Kevin Shine George^1^, Aviral Bal^1^, Cannon Lock^1^, John-Demian Sauer^2^, Megan E. Spurgeon^1,3,4^, Darcie L. Moore^5^, Brittany G. Travers^6.7^, Ron Stewart^1,&^

1- Morgridge Institute for Research

2 - Department of Medical Microbiology and Immunology, University of Wisconsin-Madison School of Medicine and Public Health,

3 - McArdle Laboratory for Cancer Research, Department of Oncology, University of Wisconsin School of Medicine and Public Health

4 - John W. and Jeanne M. Rowe Center for Research in Virology

5 - Department of Neuroscience, University of Wisconsin-Madison School of Medicine and Public Health

6 - Occupational Therapy Program, Department of Kinesiology, University of Wisconsin-Madison

7 - Waisman Center, University of Wisconsin-Madison

&-Corresponding author: [rstewart@morgridge.org](mailto:rstewart@morgridge.org).

## **Supplementary Discussion**

### What is temporal filtering and why do we need it?

Temporal filtering is a popular method for evaluating literature-based discovery (LBD) systems^1^. In temporal filtering, the LBD system is restricted to only have access to papers or abstracts up to a particular cutoff date, which is typically before the discovery is made. The LBD system is considered to have been successful if it is able to suggest a particular discovery (such as a drug would be useful for treating a disease) using literature from before the discovery is made. While we are not performing LBD here, we use temporal filtering to allow KM-GPT-DCH to evaluate hypotheses only using literature at or before a particular date. Importantly, co-occurrence model-based algorithms like SKiM^2^ can perform temporal filtering easily by only allowing papers published before a particular date to be included in the analysis. However, LLMs out of the box are not well-suited for temporal filtering because of leakage from parametric memory (see next section). Parametric memory is based on the training data. The training data and thus the parametric memory is not typically time stamped.

### What is parametric memory leakage?

Parametric memory leakage (“leakage”), in the context of KM-GPT-DCH, refers to the unintended use of information stored in the parameters and weights of a large language model (LLM) to generate results without retrieval-augmented generation (RAG). This is also called “parametric memory”^3^. This occurs because LLMs are trained on large, untimed datasets, making them unsuitable for temporal analysis when not constrained by RAG. LLMs, without external frameworks, cannot effectively be used for temporal filtering analysis as the weights and parameters of the LLM are not time stamped. For instance, we can ask an LLM (with no RAG) “Does HPV cause cervical cancer?” We would expect the LLM to answer in the affirmative because frontier LLM’s training corpus contains documents published/written after HPV was discovered as the primary cause of cervical cancer. Proving the absence of leakage when RAG is applied is crucial to ensure KM-GPT-DCH’s viability and accuracy, as it guarantees that hypothesis evaluations rely exclusively on the provided, contextually relevant literature, rather than pre-existing biases or untimed knowledge within the model. We have previously shown that our RAG approach avoids parametric leakage^4^. The ability to perform RAG where information contained in the weights and parameters is not directly used to address the question to the LLM is very important as it allows for effective temporal filtering analysis^5^.

### Confidence in LLM scores

Providing a score to represent the confidence of an answer, or a confidence interval or *p*-value of that score, by a frontier LLM is challenging. Existing methods to assess the confidence of an LLM fall mainly into the categories of verbalization, sequence probability, surrogate token, consistency, and trained probing^6^. Verbalization relies on directly asking the LLM the level of confidence it has in its response. This method is fairly accurate, even surpassing sequence probability methods^7,8^. Sequence probability methods use the log probabilities from the output tokens to estimate confidence^9^. A drawback to this method is that the confidence predicted can be about how the statement is made, not about the actual statement^6^. Additionally, frontier LLMs no longer expose their log probabilities of output tokens, which are also needed for surrogate token methods and consistency methods. Surrogate token methods involve combining verbalization and sequence probability by finding the probability of one output token, like the probability of the output token to the proposition, “is this correct?”^10^. Consistency methods involve sampling multiple answers to a question of statement and measuring the consistency between answers^11^. However, these methods do not provide a confidence score, it just gives the most consistent answer. Trained probing methods employ machine learning to extract features from hidden layers of the LLM and then train a classifier on these features to determine if the statement is true or false^12^. However, in a frontier LLM, the hidden layers are not exposed and therefore cannot be used to train. If hidden layers can be used, such as in open source models, it is often difficult to determine which layer(s) to use^13^. This type of method also requires curated training data.

Recently, researchers have employed Bayesian methods to assess the credibility and stochasticity of LLM responses as Bayesian methods have advantages over traditional frequentist methods and have shown some promise in quantifying LLM output uncertainty^14,15^.

### Chat-based LLMs for evaluating competing hypotheses

To see differences between our method and asking an LLM via chat interface, we tested four different commonly used conversational LLM-interfaces: ChatGPT(gpt-5.0)-5 and ChatGPT-4o, Perplexity (Sonar (built on Meta Llama 3.3 70B) with routing from Mistral Large 2 and Gemini 2.5 Flash), Gemini (2.5 Flash), and DeepSeek (R1-0528), and one multi-agentic system: Robin, using the Crow interface (<https://platform.futurehouse.org/>). To determine how well each LLM performed, we checked the accuracy of the citations provided and whether the LLM could determine the correct hypothesis at a given time. When attempting to ask the LLM via chat, we found there were consistently four types of errors: 1) the reference would be after the cut-off date, 2) the citation was incorrect, incomplete or unverifiable, 3) the reference was non-existent, or 4) the reference was not relevant. To test, we used two cut-off dates, the first being two years before the landmark study was published (prior), the second being three years after the publication came out (post). For example, the peptic ulcer example (landmark paper in 1984) has a prior cut-off date of 1982, and a post cut-off date of 1987. The prior cutoff date should represent the previous hypothesis (before evidence for the new hypothesis) and the post cutoff date should represent the new hypothesis (after evidence for the new hypothesis is published). The overall response result and reference evaluation for the LLM-interfaces is summarized in **Supplementary Table 2,** and all LLM answers are found in **Supplementary Data 1: *Detailed Q and A Web LLMs***.

For the first type of error, we found LLMs via chat interface could not correctly perform temporal filtering and would consistently reference websites, blogs, and papers after the cut-off date to come up with an answer. We found that 60.4% of the total number of prior references fell into this error category, and in particular, the references from Perplexity tended to be in this category, where 39 out of 41 cases used references beyond the cutoff date. For the post-study cut-off date, 51.0% of references fell into this category. This highlights that using an LLM via chat interface may be insufficient for comparing current competing hypotheses. Interestingly, this happens even when using a post-study cut-off date. Many sources in this category are summaries of the findings like Wikipedia entries, websites, or reviews, indicating that the LLM uses summarized information frequently after the cut-off date to choose the correct hypothesis.

In the second error type, reference citations were often incorrect, with incorrect authors, titles, or DOI URLs. This occurred with 11.3% of the references cited by LLM chat interfaces for the prior study cut-off date and 16.0% of the references cited for the post study cut-off date.

In the third error type, references are completely fabricated. 11.6% of the references prior to the landmark study and 5.6% after publication were completely fabricated and did not exist at all.

Finally, the fourth error type represents irrelevant citations. 1.7% (prior) and 1.5% (post) of the references cited by the LLMs were not relevant to either of the hypotheses being tested.

In total, only 15.0% of references for the pre-study cut-off date and 25.9% for the post-study cut-off date were cited correctly and were relevant. We found that overall citation accuracy was better for Robin (tested only on the cervical cancer hypothesis), although not completely without error (~85% total legitimate references for both prior and post cut offs, **Supplementary Table 3**).

Additionally LLMs by themselves cannot always get the correct answer, as they tend to use parametric data, cannot time-filter their answer (using parametric information stored in the model based on training documents after the cut-off date), and again may hallucinate if they do not know the correct answer^16^. When testing LLM-interfaces on correctness using all hypotheses, before the landmark study came out, the LLMs only provided the correct response (the pre-existing hypothesis) 66.7% of the time. Even after the published study came out (but before general acceptance, the LLMs found the correct response (the new hypothesis) only 80% of the time (**Supplementary Table 2)**. Additionally, Robin tended to choose the new hypothesis before it had been proposed (50% of the time, **Supplementary Table 3**). This shows that these systems likely used data already present within the trained model to answer the question, thus making it unsuitable for historical analysis or for analysis where it is critical to restrict to only trusted sources of information (such as PubMed). These analyses demonstrate an inability to perform temporal filtering and the common “hallucinatory” problem with LLMs^17,18^ that hinders the model’s interpretability and reliability.

Finally, both LLM chat systems and agentic systems tended to cite a narrow breadth of evaluated scientific papers (~12-13 on average for LLM chat systems (**Supplementary Table 2**) and ~8 for Robin (**Supplementary Table 3**)). This contrasts with KM-GPT-DCH, which is able to obtain a wider breadth of papers (50 at one iteration for this study, and with 10 iterations with abstracts sampled with replacement, the algorithm can evaluate hundreds of abstracts). KM-GPT-DCH also has a finer resolution scoring system and a credible interval. See **Supplementary Data 1**: ***Detailed Q and A web-based LLMs*** for detailed questions and responses from web-based LLMs and agentic systems in evaluating competing hypotheses.

There are several advantages to the KM-GPT-DCH system over chat-based LLM systems:

1. No hallucinated citations, and greatly reduced LLM hallucinations in reasoning and evaluation.

2. For KM-GPT-DCH, the exact text that the LLM is reasoning about is transparent, which is not necessarily true for other LLM-based implementations.

3.  Parametric memory leakage is not an issue with KM-GPT-DCH but is a primary source of information for other LLM implementations. This makes determining the sources contributing to the output challenging and temporal filtering impossible.

4. Importantly, KM-GPT-DCH tightly controls dates and allows true temporal filtering to understand the history of a hypothesis and to be able to accurately assess the support for a hypothesis at a particular time.

5. Chat-based LLM implementations choose the papers to discuss, and it isn’t clear how they are chosen. In KM-GPT-DCH, the system reliably chooses the articles to reason about based on co-occurrence and temporal filtering dates.

6. KM-GPT-DCH can be as comprehensive as the user desires. It can evaluate 5 abstracts, or 500 abstracts. It is not generally possible to tune the level of comprehensiveness in web-based systems.

### KM co-occurrence results

Below are results from the KM model on the other four main historical hypothesis pairs. In many of the cases, KM results in the correct term being favored at a given time but of course cannot understand the relationship of the two terms.

**Cervical cancer**

With the co-occurrence model (KM), we use the two-proportion z-test (z-interval) to determine if there is a difference between the proportion of abstracts relevant to the new hypothesis (HPV) and the proportion of abstracts relevant to the preexisting hypothesis (herpes simplex virus or HSV). During this time (1983-1996), we see with the co-occurrence model (**Supplementary Fig. 1a**) that more papers were published with cervical cancer associations with HPV than HSV, and the proportion is significantly higher by 1984 (*p* <0.05). There was an even stronger association of HPV with cervical cancer when compared to HSV by 1987 (*p* < 1e-49), well before the time of general acceptance of HPV as causal to cervical cancer in 1996.

**Scrapie**

Similarly, we used KM to look at the second pair of competing hypotheses of whether scrapie is caused by a protein or a virus infection. We found that KM could detect a significantly higher proportion of abstracts associating scrapie with protein infection than with viral infection by 1986 (*p* < 0.05). By 1990, co-occurrence with protein infection was very strong (*p* < 1e-10) (**Supplementary Fig. 1b**), indicating that well before the general acceptance date of 1997, protein infection was significantly more highly associated with scrapie than viral infection.

**Autism**

KM shows strong co-occurrence numbers with autism and vaccine link in 1998 and for some years after, although the ratio from KM always favors the genetic predisposition term, if only by a low margin from 1998 into the early 2000s. In this case, some of these papers in which “vaccines” and “autism” co-occur after 1998 address and refute the claim that vaccines cause autism, causing the co-occurrence ratio to increase even though the actual support of this claim decreases (**Supplementary Fig. 1c)**. Although in **Supplementary Fig. S1c** we see high numbers of abstracts co-occurring between autism and vaccines, the total number of abstracts containing the term “vaccines” is also rising, so that the ratio of “autism AND vaccines” over “vaccines” is lower than the ratio of “autism AND genetics” over “genetics”. Thus, the z-score remains positive throughout time (favoring the genetics term) because the ratio of “autism AND genetics” over genetics is always higher. We also see a drop-off of “autism AND vaccines” papers to zero starting in 2023. This is because at this point, Fisher’s Exact Test no longer finds a significant number of “autism AND vaccines” abstracts relative to overall “vaccines” abstracts (*p*-value < 0.05), and we do not obtain any counts that are past this cutoff. This highlights a downside to using *p*-value cutoffs as they can distort the data based on what is deemed “significant”. It is also another reason KM-GPT-DCH is a better approach as this method employs a Bayesian credible interval around a score, but not a hard cutoff of when a result is considered significant or not.

**ASCVD**

Finally, KM analysis compares HRT (“hormones”) with statins showing statins with a significantly higher abstract count than hormone therapy by 1996. This trend continues to climb in favor of statins through 2025 (**Supplementary Fig. 1d**). This example underscores the importance of temporal trends in therapeutics. Prior to statin use for ASCVD risk reduction, clinical equipoise existed as to whether HRT could be used as a method to reduce ASCVD risk, but following the results of randomized controlled trials, HRT was no longer viewed as a method for ASCVD risk modification.**
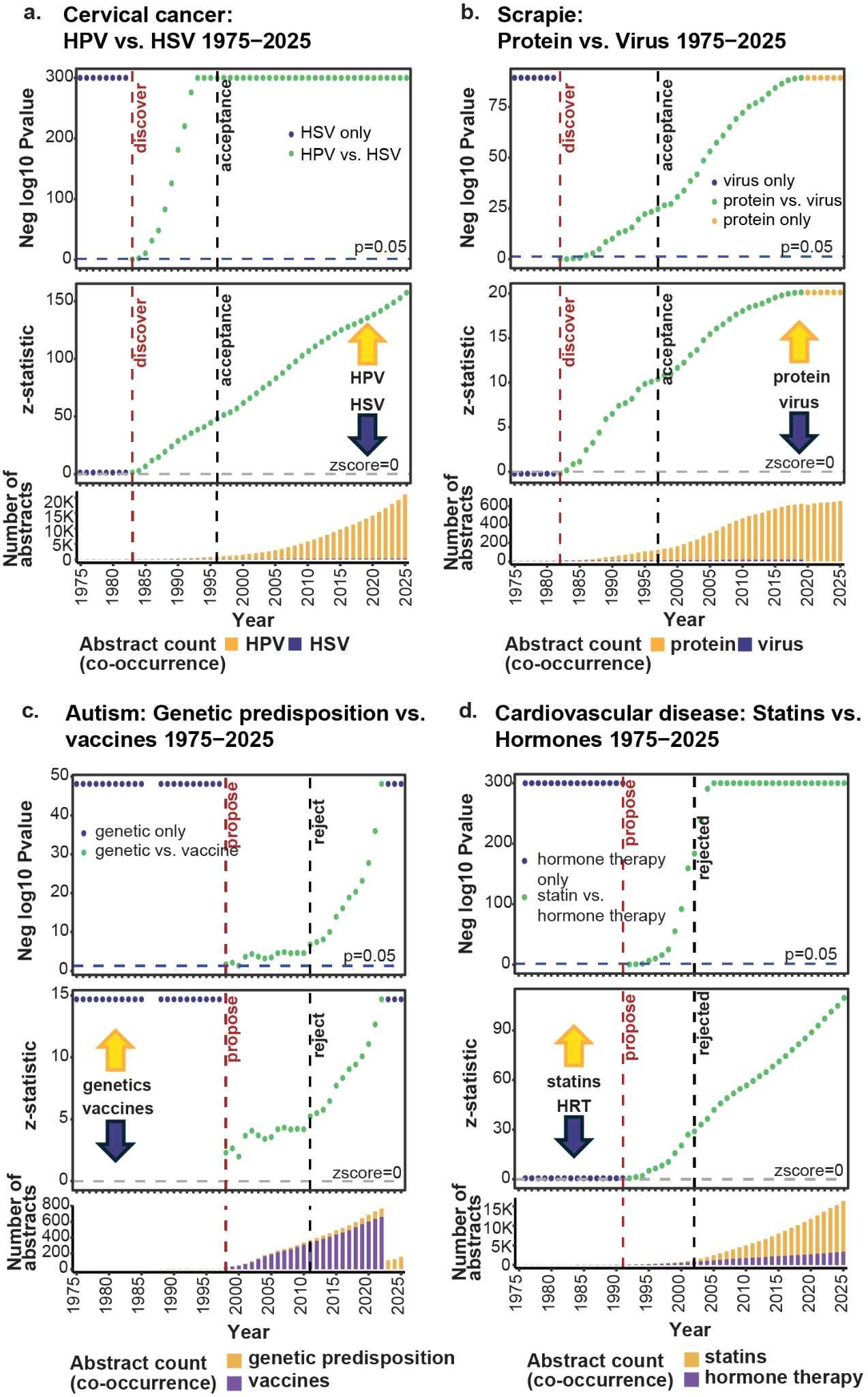
Supplementary Figure 1**: **KinderMiner with ratio of ratios z-statistic**

All panels (**a,b,c,d**) show the results from the two-proportion *z*-test for each year between the sets of competing hypotheses (H1 vs. H2) using the KM algorithm from 1975 to 2025, run cumulatively. The top panels show the negative log10 of the *p*-value, where the dashed y-intercept line is the adjusted *p*-value of 0.05. The middle panels show the *z*-statistic, where the dashed line represents *z*-statistic of 0, where above zero the algorithm favors H1 and below zero the algorithm favors H2. Finally, the bottom panels show the number of abstracts obtained for each B-term that co-occur with the corresponding A term.

**a**. KM results from cervical cancer causation hypotheses; H1: Human Papillomavirus (HPV), H2: Herpes Simplex virus (HSV). The ‘discover’ x-intercept line indicates when HPV was found as a causal agent and the ‘acceptance' x-intercept line indicates when the HPV cause was accepted by the scientific community. **b.** KM results from scrapie infection causation hypotheses; H1: protein infection, H2: virus infection. The ‘discover’ x-intercept line indicates when proteins were discovered to cause infections like scrapie and the ‘acceptance' x-intercept line indicates when prions were accepted as a causal agent. **c.** KM results of autism causation hypotheses, where genetic predisposition is H1 and vaccines is H2. The ‘propose’ x-intercept line indicates when autism was proposed to be caused by vaccines by the 1998 Wakefield paper and the ‘reject’ x-intercept line indicates when the autism/vaccine hypothesis was rejected by the retraction of the 1998 paper. **d.** KM results of atherosclerotic cardiovascular disease (ASCVD) preventative treatment hypotheses, where statins are H1 and hormone replacement therapy (HRT) is H2. The ‘propose’ x-intercept line indicates when hormone therapy was proposed to reduce ASCVD risk, the ‘reject’ x-intercept line indicates when the ASCVD/hormone therapy hypothesis was rejected by a large, randomized trial.

H1: Hypothesis 1

H2: Hypothesis 2

KM: KinderMiner co-occurrence algorithm

—------------------ end of figure legend —------------------

### Running KM-GPT in a non-comparative way

When KM-GPT is run in a non-comparative way, positive results for both hypotheses are usually produced. This is largely because when KM obtains abstracts for only one of the hypotheses, there may be correlative or weak evidence for the hypothesis and is not directly refuted, thus deserving a perhaps lower but still positive score.


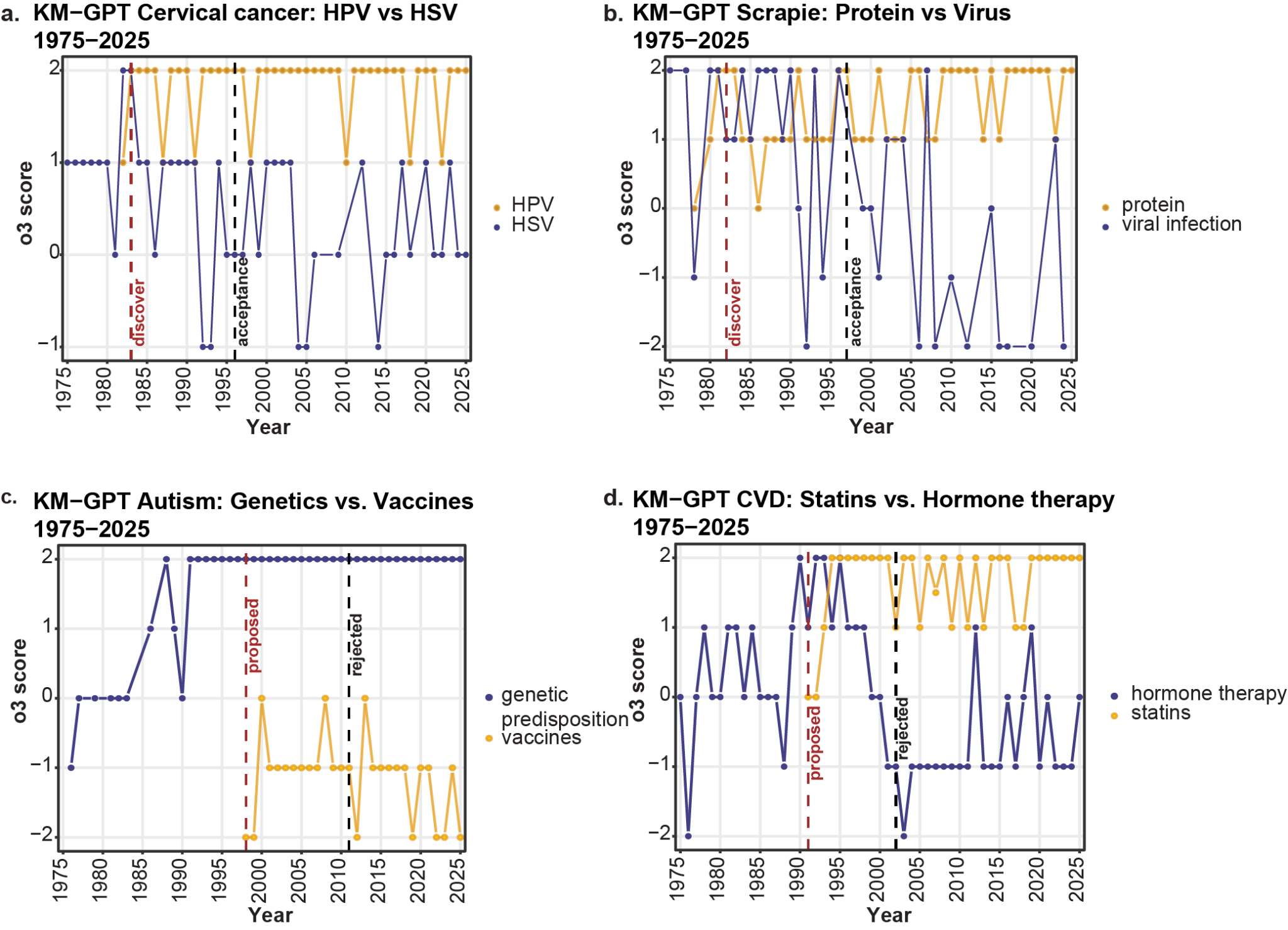


**Supplementary Figure 2.** **KM-GPT runs in a non-comparative way.**

KM-GPT scores over time (1975-2025) for each hypothesis when run as a single hypothesis, run with one iteration. The y-axis shows the score value ranging from +2 to -2, where +2 KM-GPT strongly agrees with the hypothesis and -2 KM-GPT strongly disagrees with the hypothesis. The x-axis shows the given year, and the lines are color coded for one of the hypotheses. **a.** The cause of cervical cancer, HPV (yellow) or HSV (purple). **b.** The cause of scrapie, protein (yellow) or viral infection (purple). **c.** The cause of autism, genetic predisposition (purple) or vaccines (yellow). D. The best treatment for cardiovascular disease, statins (yellow) or hormone therapy (purple). Note that after the mid 2000s, HRT was no longer viewed as a treatment for reducing ASCVD risk.

KM-GPT: KinderMiner with a Generative Pretrained Transformer.

—------------------ end of figure legend —------------------

In the case of cervical cancer, when running KM-GPT in a non-comparative way, positive scores are produced (with a range from -2 to +2, where +2 is highly supportive of the hypothesis and -2 indicates the hypothesis is strongly refuted) for both HSV and HPV, including positive scores for HSV as a causal agent well after general acceptance that HPV is the most likely cause of cervical cancer (**Supplementary Fig. 2a**). This is because HSV, while not the causative agent, is still strongly correlated with cervical cancer. If the LLM is not comparing this cause to anything else and only has abstracts citing correlations of HSV and cervical cancer, it will give this a positive score for being a cause. Thus KM-GPT-DCH provides a better evaluation of the cause of cervical cancer, as KM-GPT-DCH evaluates abstracts for both HSV and HPV at the same time, and can choose the more likely hypothesis.

Similarly, when running KM-GPT in a non-comparative way in the case of scrapie, positive scores are produced for both protein and virus as causal agents, including some positive scores (although also some negative scores) after the acceptance date (**Supplementary Fig. 2b**). When looking at the abstracts from 2007 when the viral hypothesis gets a high score, there are indeed abstracts that support a viral hypothesis (3 abstracts), but many more support a protein hypothesis (37 abstracts). Therefore, if run in a non-comparative way, the LLM only sees abstracts relevant to one of the hypotheses, which would make one hypothesis seem plausible even if the other hypothesis has more evidence. In contrast, when running KM-GPT on the autism causal hypotheses of genetic predisposition or vaccines, the vaccine hypothesis never gets a positive score while genetic predisposition gets a high positive score (+2) starting from the early 1990s continuing through today (**Supplementary Fig. 2c**). This indicates that there is little to no evidence that vaccines cause autism. Thus, when we compare two hypotheses, KM-GPT-DCH is able to choose the overall better one, while KM-GPT is not always able to clearly identify the better hypothesis. See **Supplemental Data 2** for all non-comparative KM-GPT runs.

### LLM reasoning for hypotheses

For each hypothesis, the LLM provides reasoning along with the score. Here the reasoning and the references used for one historical hypothesis pair is provided, to show that 1) the reasoning is indeed sound and 2) that the LLM bases its decision on the abstracts of the given time period and not its parametric data.

#### Cervical cancer

As mentioned in the main text, HSV was the dominant theoretical cause of cervical cancer from 1975-1982, for which our algorithm scored in favor of HSV in these years. In the years following the report by Durst *et al.* in 1983^19^ HPV was hypothesized as the leading cause, during which KM-GPT-DCH scored in favor of HPV. Beginning in 1975 (mean score of 18.7), the reasoning states that based on the 6 abstracts provided, 5 of them support HSV-2 as the cause of cervical cancer. These abstracts showed that there were higher amounts of HSV-2 antibodies in cervical cancer patients compared to matched controls^20^ or that HSV-2 is linked to an increased risk in cervical cancer^21^. In 1984, when the algorithm favored HPV (mean score of 82), there were 4 abstracts that support HPV as the cause of cervical cancer while only 2 support HSV. The four abstracts supporting HPV provided molecular evidence, including isolated HPV DNA from cervical cancer biopsies^22^ while the two abstracts supporting HSV-2 only show correlative antibody associations with cervical cancer. The reasoning confirms that the algorithm is basing its decision on these abstracts and the correct time period with no parametric memory leakage. See below for one iteration of results from a 1984 run by the KM-GPT-DCH algorithm and **Supplementary** **Table 1** for a list of the various hypotheses and when/if they were resolved by KM and KM-GPT-DCH. For all KM-GPT-DCH outputs, see **Supplemental Data 2**.

#### Peptic Ulcer

The peptic ulcer causation hypotheses are not as straightforward because as mentioned in the main text. While H. pylori is the bacterium that is the main causal agent to ulcers, psychological stress is also an aggravating factor. KM-GPT-DCH results vary, but eventually by 1987 consistently favored the bacterial hypothesis as the better one. As shown in the result example below, in 1987 multiple studies found H. pylori in duodenal ulcers^23–26^, while only one study found correlation with psychological stress^27^ but two others did not^28,29^. The algorithm correctly evaluates that the bacterial hypothesis was stronger with evidence from multiple studies, whereas evidence for a psychological stress causal component was weaker and, in some studies, refuted. We therefore agree with the overall reasoning of the LLM given the abstracts it evaluated.

### Example KM-GPT-DCH results

The following is an example of KM-GPT-DCH results for a 1984 run of the cause of cervical cancer, hypothesis 1: HPV, hypothesis 2: HSV. 1984 is a year after the landmark paper came out showing HPV as a causal agent, and the time when the HPV hypothesis clearly becomes the favorable hypothesis.

**Per abstract reasoning (LLM-generated):**

- PubMed ID: 6100284
  - label: supports_H1
  - evidence: The abstract notes an “association ... between HPV-6, -10, -11, -16, and -18 and cervical cancer”, implicating HPV in the disease.
- PubMed ID: 6399280
  - label: supports_H1
  - evidence: It states “Herpes simplex virus type 2 remains a candidate... but results... inconsistent” and that “Human papillomaviruses are clearly more important for cervical cancer”.
- PubMed ID: 6318956
  - label: supports_H1
  - evidence: Opening sentence: “Human papillomaviral (HPV) infection is now widely advanced as an important etiologic factor in cervical cancer.”
- PubMed ID: 6329740
  - label: supports_H1
  - Evidence: HPV-18 DNA was found in 9/36 cervical carcinomas while absent from benign lesions, showing viral presence specifically in cervical cancer biopsies.
- PubMed ID: 6100279
  - label: supports_H2
  - evidence: 73% of cervical cancer patients had HSV-2-specific antibodies vs 30.7% of matched controls; significant difference suggests HSV involvement.
- PubMed ID: 6086470
  - label: supports_H2
  - evidence: Study reports 96% HSV seropositivity in cervical cancer patients vs 87% controls; authors note previous HSV-2 infections “predispose women” for cervical cancer.

**Overall score rationale (LLM-generated):**

Four abstracts provide molecular or epidemiologic evidence linking HPV to cervical cancer (PubMed IDs 6100284, 6399280, 6318956, 6329740). Two abstracts report higher HSV-2 antibody prevalence in cervical-cancer patients (PubMed IDs 6100279, 6086470); evidence is serologic and authors concede causality unproven. HPV evidence includes viral DNA in tumors and a stated etiologic role, stronger than antibody associations for HSV. Thus support for H1 outweighs H2 in both quantity (4 vs 2) and strength; little to no evidence favors both, and none directly refutes HPV. Following guidelines, H1 appears roughly three times more plausible than H2, meriting a score around 80.

**Tallies:**

- support_H1: 4
- support_H2: 2
- both: 0
- neither_or_inconclusive: 0

**score:** 80

**decision:** H1

The following is an example of KM-GPT-DCH results for a 1987 run of the cause of peptic ulcer: hypothesis 1: bacterial infection or hypothesis 2: psychological stress. 1987 is the year that the algorithm begins to favor bacterial infection as the primary cause of peptic ulcers.

**Per abstract reasoning (LLM-generated):**

- PubMed ID: 3615089
  - label: neither
  - evidence: Associates C. pyloridis with primary antral gastritis in children; peptic ulcer not studied or mentioned.
- PubMed ID: 3310428
  - label: supports_H1
  - evidence: Ultrastructural localization of C. pylori in 40% of duodenal ulcer cases; authors suggest infection can lead to direct mucosal damage.
- PubMed ID: 3623220
  - label: neither
  - evidence: Study of ranitidine/vagotomy effects on gastric pH and bacteria; does not address ulcer causation by bacteria or stress.
- PubMed ID: 3576129
  - label: neither
  - evidence: Shows link between C. pyloridis and gastritis/duodenitis, but peptic ulcer not examined, so causal claim for ulcer not supported.
- PubMed ID: 3297911
  - label: inconclusive
  - evidence: Review notes correlation of Campylobacter-like organisms with ulcers but states pathogenetic significance 'remains unknown'.
- PubMed ID: 3582869
  - label: supports_H1
  - evidence: 19 of 21 duodenal ulcer patients were C. pyloridis positive (p<0.001); tight association with ulcers reported.
- PubMed ID: 3573680
  - label: supports_H1
  - evidence: Bacteria found at edge of active duodenal ulcers, penetrating mucosa; authors state organisms 'are indeed pathogenic' for ulcers.
- PubMed ID: 3652784
  - label: supports_H1
  - evidence: High culture positivity: 84% of duodenal ulcers and 77% of gastric ulcers carried C. pyloridis.
- PubMed ID: 3685879
  - label: neither
  - evidence: Deals with endotoxin in secondary peritonitis; no mention of peptic ulcer or psychological stress.
- PubMed ID: 3443224
  - label: neither
  - evidence: Personality traits did not differ between ulcer responders and non-responders; implies little psychological influence on relapse.
- PubMed ID: 3625586
  - label: supports_H2
  - evidence: Duodenal ulcer group showed higher state anxiety compared with community controls, suggesting psychological involvement.
- PubMed ID: 3672046
  - label: neither
  - evidence: Population study found no support for concept that peptic ulcer is related to psychological stress.
- PubMed ID: 3477993
  - label: inconclusive
  - evidence: Review says role of psychosomatic reactions in peptic ulcer development is 'still unclear'.

**Overall score rationale (LLM-generated):**

Four abstracts strongly associate Campylobacter/Helicobacter infection with duodenal or gastric ulcers (3310428, 3582869, 3573680, 3652784). Only one abstract presents evidence for psychological factors (3625586), while another explicitly finds no stress link (3672046) and others are neutral or unclear (3443224, 3477993). No abstract refutes bacterial involvement; several show high prevalence or tissue invasion implying causation. Given roughly 4:1 supportive evidence favoring infection and contradictory/weak evidence for stress, the bacterial hypothesis is about three times more likely.

**Tallies:**

- support_H1: 4
- support_H2: 1
- both: 0
- neither_or_inconclusive: 8

**score:** 78,

**decision:** H1

Finally, we show KM-GPT-DCH runs for the competing hypotheses for preventing atherosclerotic cardiovascular disease (ASCVD), statins (hypothesis 1) or hormone therapy (hypothesis 2). While early data raised the hypothesis of HRT for ASCVD reduction, in 2002, use of hormone therapy to reduce ASCVD risk was refuted by randomized control trials, highlighting the rejection of this clinical hypothesis. Our algorithm demonstrates this shifting temporal trends in therapies. Early on KM-GPT-DCH supports HRT, with robust demarcation when the hypothesis was rejected, and the rising and strong support of statins starting in the 1990s. Initial longitudinal cohort observational data in 1991^30^, demonstrated a reduction in ASCVD risk (relative risk reduction 0.50 with a 95% CI, 0.43-0.56) with the use of HRT, which led to widespread adoption of use to treat menopausal symptoms with the potential to reduce ASCVD events^31^. However, this idea was subsequently refuted in two large randomized clinical trials: the Heart and Estrogen/progestin Replacement Study (HERS) in 1998 and in the Women’s Health Initiative (WHI) in 2002-2004^32–35^, leading to significant concerns about HRT use and risk of harms in post-menopausal women resulting in drastic reductions (70-80%) in the use of HRT to treat menopausal symptoms^36^. Currently, all major societies recommend against using HRT for ASCVD prevention at any age, this was further supported by a 2025 Cochrane review showing no evidence that HRT has any role in the treatment or prevention of ASCVD^37,38^. Despite these findings, the clinical need to manage menopausal symptoms persisted. Subsequent secondary analyses suggested that the cardiovascular harms of HRT may be time-dependent, with lower risks when initiated closer to menopause onset, informing current guidelines that support risk/benefit discussions for HRT use for bothersome menopausal symptoms in eligible, recently menopausal individuals^36,39,40^. Currently, in clinical practice, HRT is used primarily to address moderate-severe vasomotor and genitourinary symptoms of menopause in eligible individuals, with therapy individualized based on discussion of risks and benefits^41^.

Example KM-GPT-DCH result from 2002:

**Per abstract reasoning (LLM-generated):**

- PubMed ID: 12523144
  - label: supports_H1
  - evidence: Simvastatin 40 mg reduced non-fatal MI or coronary death from 11.8 % to 8.7 % in 20,536 high-risk patients adding to evidence cholesterol-lowering with statins is indicated.
- PubMed ID: 11966668
  - label: supports_H1
  - evidence: Good long-term persistence with statins is noted and authors remark younger drop-outs may miss the benefit of decreased coronary morbidity and mortality obtainable with statin therapy.
- PubMed ID: 12002257
  - label: supports_H1
  - evidence: Review states statins significantly reduce cardiovascular mortality and morbidity and describes pleiotropic vasoprotective mechanisms.
- PubMed ID: 12441891
  - label: supports_H1
  - evidence: Heart Protection Study showed LDL lowering to 1.7 mmol/l produced significant clinical benefit with no apparent threshold, supporting aggressive statin use.
- PubMed ID: 12454325
  - label: supports_H1
  - evidence: Estimated 10-yr CHD risk fell more on statin therapy (19.7% to 13.2 %) than on antihypertensives, leading authors to urge wider statin use in hypertension.
- PubMed ID: 11872916
  - label: supports_H1
  - evidence: Invited review highlights statins as a new avenue for stroke prevention via plaque stabilization and anti-inflammatory effects.
- PubMed ID: 12817199
  - label: supports_H1
  - evidence: Landmark trials (4S, WOSCOPS, HPS, etc.) collectively show statins cut adverse cardiovascular events even in low-cholesterol or primary prevention settings.
- PubMed ID: 12243846
  - label: supports_H1
  - evidence: Landmark clinical trials have demonstrated benefit of lipid lowering with statins for primary and secondary CHD prevention; additional pleiotropic effects noted.
- PubMed ID: 12804322
  - label: both
  - evidence: Statins lower CVD risk and, when combined with hormone replacement therapy, produced significant beneficial effects on endothelial function.
- PubMed ID: 12530513
  - label: supports_H1
  - evidence: Statins u2018reduce myocardial infarction, stroke, and deathu2019 by modifying atherosclerosis in symptomatic or asymptomatic patients.
- PubMed ID: 12448270
  - label: supports_H1
  - evidence: Guideline recommends statins as first-choice drugs; sets LDL targets for high-risk persons.
- PubMed ID: 12432482
  - label: supports_H1
  - evidence: Review lists statins among evidence-based secondary prevention measures with good risk-benefit ratio.
- PubMed ID: 12369881
  - label: supports_H1
  - evidence: Accumulating evidence shows statins cut CVD events and offer plaque stabilization, vascular relaxation and other benefits beyond lipids.
- PubMed ID: 12114036
  - label: supports_H1
  - evidence: Randomized 20,536-patient trial: simvastatin cut major vascular events by 24 % irrespective of baseline cholesterol; clear mortality benefit.
- PubMed ID: 12467937
  - label: supports_H1
  - evidence: States statin monotherapy benefits combined dyslipidemia patients; combination can yield additional lipid improvements.
- PubMed ID: 15562337
  - label: supports_H1
  - evidence: Pleiotropic effects of statins well documented; growing use reflects aggressive treatment of hypercholesterolemia.
- PubMed ID: 12415329
  - label: supports_H1
  - evidence: Review notes benefits of lipid-lowering medications (specifically statins) in atherogenesis and CVD prevention.
- PubMed ID: 12049990
  - label: supports_H1
  - evidence: Statins can lower LDL up to 55 %; listed as key agents in lipid-modifying armamentarium for atherosclerosis.
- PubMed ID: 12100064
  - label: neither
  - evidence: Discusses growth hormone deficiency and vascular risk; statins or hormone replacement not addressed.
- PubMed ID: 12190834
  - label: supports_H2
  - evidence: Natural menopause linked to metabolic risk; Hormone replacement therapy has been shown to attenuate these changes.
- PubMed ID: 15765625
  - label: neither
  - evidence: Recent randomized trials show HRT does not confer cardioprotection and actually increases cardiovascular risk.
- PubMed ID: 14727955
  - label: supports_H2
  - evidence: In Turner syndrome, short-term studies show HRT lowers blood pressure, suggesting cardiovascular benefit.
- PubMed ID: 11934187
  - label: supports_H2
  - evidence: Much evidence suggests estrogen exerts a protective effect against CVD and HRT continues this effect post-menopause.
- PubMed ID: 12433033
  - label: supports_H2
  - evidence: Authors state lifelong estrogen therapy will protect against osteoporosis and cardiovascular disease in patients with gonadal dysgenesis.
- PubMed ID: 12379955
  - label: supports_H2
  - evidence: Experimental and clinical evidence indicate part of cardiovascular benefits of estradiol stem from direct endothelial actions.
- PubMed ID: 12434666
  - label: inconclusive
  - evidence: HRT improves lipid profile but oral estrogen raises CRP; authors note possible early risk increaseu2014mixed cardiovascular impact.
- PubMed ID: 11861028
  - label: supports_H2
  - evidence: Review notes estrogen diminishes homocysteine levels, a CVD risk factor, offering a potential protective mechanism.

**Tallies:**

- support_H1: 17
- support_H2: 6
- both: 1
- neither_or_inconclusive: 3

**Overall score rationale (LLM-generated):**

Robust RCTs and multiple reviews (PubMed IDs 12114036, 12523144, 12817199) consistently confirm statins lower MI, stroke, and mortality. 17 abstracts support H1, only 6 support H2. One citation (15765625) explicitly reports increased CV risk with HRT, tempering Hormone-therapy evidence; others are observational or mechanistic. Overall evidence is roughly triple in volume and higher quality for statins versus hormone therapy, aligning with guideline recommendations (12448270).

**score:** 78

**decision:** H1

### Additional information on peptic ulcer

The first description of a spirochete and suggestion of its role in gastric ulcers was first mentioned in 1875 by Bottcher and Letulle^42^. Warren first noted the spirochetes associated with gastric ulcer in 1979, isolated them in 1982, and published the results in 1983^43^. *Helicobacter pylori* was originally called *Campylobacter pyloridis* in 1982, changed to *Campylobacter pylori* in 1987^44^, and finally reclassified as *Helicobacter pylori* in 1989^45^.

Approximately 10% of peptic ulcer cases are idiopathic. It is possible that stress or some other genetic or infectious cause could be primary in these cases.

### Additional information on the prion hypothesis

The causal agent of scrapie has long been debated. An early proposal was the slow virus hypothesis^46^, followed by the virino hypothesis proposed in the late 1970s and early 1980s^47^ followed by prions being a distinct infectious agent^48^. Note that the self-replicating protein idea was first proposed by Griffith, with support from experiments by Alper and Pattinson in 1967^49^. Support for the prion hypothesis strengthened during the 1990s^50^. In recent years, many other neurodegenerative diseases are being termed prion-like (such as Alzheimer's disease, ALS) having the alteration in protein conformation as an important contributor or underlying cause^51,52^.

### Additional information on genetic predisposition, vaccines, and autism

While scientific evidence supports the genetic predisposition theory but does not support the vaccine theory of autism, controversy persists in the public. Despite the evidence against the vaccine hypothesis and for the genetic predisposition hypothesis, childhood vaccination rates have been decreasing in the US^53^. Indeed, numerous factors likely contribute to portions of the public continuing to endorse the vaccine-autism theory despite the existing scientific evidence for genetic predisposition. For one, the gene theory of autism is complex, suggesting that the genetic susceptibility of autism stems from a combination of multiple sources (*e.g.*, common inherited variation, rare inherited genes, and rare *de novo* genes)^54–57^ that may contribute differently to autistic traits, motor skills, and intellectual disability^54,58,59^. Because of the gene-theory complexities, people may prefer the simpler, albeit less data-based theory, of vaccines and autism. Additionally, the movement to democratize health decisions by eschewing expertise may contribute to people rejecting the scientific consensus^60^. However, there remains no link between autism and vaccines (for a systematic review and meta-analysis see^61^). Meanwhile, the evidence for genetic contributions and their ability to account for the considerable heterogeneity in the autism behavioral profile^62^ and neurobiological profile^63^ continues to grow.

### Current competing hypotheses

Additional detail on the 20 currently controversial and/or competing hypotheses is provided here, as well as in **Supplementary Table 4**.

#### Alzheimer’s disease

Alzheimer's disease (AD) is characterized by severe dementia and memory loss, and is thought to be caused by either the accumulation of a mutated form of the amyloid‑beta (Aβ) protein in the brain that results in a plaque (H1) or the hyperphosphorylation of the tau (τ) protein that then aggregates and forms neurofibrillary tangles (H2)^64^. In support of the Aβ plaque hypothesis, mouse models show a reduction of Aβ plaques by drugs or exercise improved cognitive abilities^65,66^. Additionally, in autosomal dominant Alzheimer's disease (ADAD), Aβ plaques accumulate higher in ADAD carriers of the gene than non-carriers, and the plaques accumulate before cognitive impairment occurs^67^. In the tau protein hypothesis, this microtubule binding protein undergoes hyperphosphorylation which causes the dissociation of tau from microtubules, leading to neurofibrillary tangles and the onset of AD^68^. It has been shown that tau-targeted immunotherapies can rescue cognitive deficits in multiple animal or clinical studies^69–71^. Additionally, a longitudinal human study shows that tau accumulation is associated with memory impairment independent of Aβ status, indicating tau proteins may play a more primary role in AD^72^. Finally there is some evidence of a synergistic interaction between Aβ and tau, implicating both hypotheses as causal^73,74^. Our model finds about 3 times more abstracts supporting tau pathology as being the primary cause, compared to abstracts that support the amyloid plaque hypothesis. Overall, our model gives a posterior mean score of 31.9 with the credible interval just shy of the 50 mark, so the tau pathology hypothesis is slightly more favored.

#### Endometriosis

What is the mechanism of the pathogenesis of endometriosis? Endometriosis develops when endometrial tissue grows outside of the uterus, causing lesions, pain, nodules, infertility, and/or other symptoms^75,76^. Two of the main competing theories for the pathogenesis of this are retrograde menstruation (H1) and coelomic metaplasia (H2). Retrograde menstruation happens when menstrual blood containing endometrial cells flows backward into the peritoneal cavity and then these cells then implant and grow lesions ^76^. However, this method does not explain the rarer occurrences of endometriosis that happens outside of the peritoneal cavity. The coelomic metaplasia theory, on the other hand, suggests that coelomic epithelial cells transdifferentiate (metaplasia) and form endometrial stroma. This would explain endometriosis that occurs in other places, such as the ovary^76^. Our model leans toward the main causal hypothesis being retrograde menstruation (with a mean score of 91.1), in which most of the abstracts support, but it is possible different types of endometriosis have different causes.

#### REM sleep

What is the primary function of REM sleep? REM sleep is characterized by rapid eye movement, enhanced brain activity, and often dreaming, and oscillates between non-REM sleep 4-5 times per night^77^. The purpose of REM sleep is debated, with two main hypotheses being memory consolidation (H1) and brain homeostasis (H2). Memory consolidation involves the coding and storage of important memories^77^. The evidence for this occurring during REM sleep includes inability to recall recent memories that have not already been learned after REM sleep deprivation^78^. The counter-argument of brain homeostasis is that REM sleep is essential for emotional well-being and optimizing stress response, and REM sleep abnormalities are linked to psychiatric disorders like depression and PTSD^78,79^. Our model leans heavily toward the hypothesis of memory consolidation (mean score 88.4) with most literature supporting this hypothesis; however, it is possible that both functions could be happening together.

#### Red wine

Cardiovascular health benefits have been associated with red wine^80^, but it is unclear as to whether this is due to antioxidants present in the skin of grapes or due to the alcohol itself ^81^. Polyphenol antioxidants in red wine, such as resveratrol, have been shown to have therapeutic benefits to conditions such as pulmonary arterial hypertension and reduce oxidative stress ^80,82^, and alcohol abuse can have negative cardiovascular effects^83^. However, alcohol in moderation is thought to have an antithrombotic effect through the inhibition of platelet function and also decrease in LDL cholesterol concentration, both of which help prevent cardiovascular disease^84^. With a score of 81.6, our algorithm finds most papers support the antioxidant hypothesis.

#### Polycystic Ovary Syndrome

What is the driver of polycystic ovary syndrome (PCOS)? PCOS is a common infertility disorder and symptoms include hyperandrogenism and polycystic ovaries^85^. It is not well known what the underlying cause is for PCOS, whether it is primarily an ovarian or a neuroendocrine abnormality. The ovarian hypothesis states that defects begin in the ovary and affect androgen production while the neuroendocrine hypothesis indicates that abnormalities in the brain cause the ovary dysfunction. In support of the ovarian hypothesis, when compared to normal theca cells (TCs) from the ovaries, TCs from PCOS patients produce androgens at a higher rate, causing hyperandrogenism, which in turn causes a decrease in autophagy, which can ultimately cause PCOS^86^. In support of a neuroendocrine cause, women with PCOS have been shown to have frequent [gonadotropin-releasing hormone](https://en.wikipedia.org/wiki/Gonadotropin-releasing_hormone) (GnRH) pulses, which is a neurohormone from the pituitary gland in the brain, and higher levels of the signaling molecule kisspeptin, which initiates GnRH secretion^87^. Our algorithm mostly supports a neuroendocrine causal model with a mean score of 63.5, with slightly more relevant abstracts citing central neuroendocrine dysfunction, although not without some support for the ovarian hypothesis.

#### Aging

What is the primary mechanism of aging? The first hypothesis is that aging is the result of stochastic damage to the DNA and cells that accumulates over time. The second hypothesis is that specific genes regulate changes and hormonal signaling to induce aging, likely to limit population size and induce generational turnover. It has been shown that deletions in mitochondrial DNA correlate with aging as well as other disorders^88^, that somatic mutations throughout the body accumulate with age^89,90^, and that DNA damage induces senescence in cells^91^, supporting the first hypothesis that accumulated DNA damage can cause aging. However, loss of function of specific gene products (like parathymosin) have been shown to cause neurodegeneration and a reduced lifespan^92^ and genome-wide association studies have linked multiple loci with longevity^93^, supporting the genetic hypothesis. Our algorithm favors the stochastic damage hypothesis with a mean score of 85.9 because this has been shown in a mechanistic way, and more publications support this hypothesis.

#### Acupuncture

How does acupuncture treatment produce analgesic effects? The first hypothesis attributes the analgesic effects to the release of endorphins when needling^94^. The second hypothesis argues that pain is alleviated due to diffuse noxious inhibitory controls (DNIC), in which a counter irritation inhibits wide dynamic range (WDR) neurons, which in turn inhibits the pain^95^. Studies have found that acupuncture increases β-endorphin levels^96,97^, supporting hypothesis one. Some evidence for the DNIC mechanism (hypothesis two) exists as well in rats where electro-acupuncture suppressed the firing of C-fiber-evoked WDR neurons^98^. However, the number of studies that link pain-relief from acupuncture to increases in endorphins include clinical trials and outnumber studies with supporting DNIC as the causal mechanism, and our algorithm favors the first hypothesis with a mean score of 87.0.

#### Cancer Progression

What is the primary reason for the progression of cancer? Two main competing hypotheses in the field include the Somatic Mutation Theory (SMT) and the Tissue Organization Field Theory (TOFT)^99^. The SMT argues that cancer begins with a single cell that accumulates mutations in its DNA which allow it to replicate uncontrollably, and this initiates and drives cancer progression^100^. Evidence for SMT includes the sequencing of several different types of tumors and finding hundreds of cancer driver genes with mutations under positive selection that cause the progression of cancer^101^. Additionally many different types of cancer identify somatic mutations in tumor suppressor or oncogenes that facilitate the progression of cancer, supporting SMT^102–106^. In contrast, the tissue organization theory (TOFT) posits that the default state of cells is proliferation with variation and motility, but cells are constrained by cell-cell interactions within a tissue. When these constraints are disrupted, they return to the default state of proliferation, causing cancer^99^. Additionally, tissue organization can become dysregulated by changes in bioelectricity which exists from the cell membrane potential. A study that supports this shows that cell depolarization can cause a change in cell state and can propagate this state to other cells without any genomic mutation^107^, providing evidence for TOFT. Multicellularity produces a petrified cell phenotype in which a fully differentiated sessile somatic cell ultimately cannot contribute to the organism’s reproduction. If the cell is freed from these constraints of the tissue, it can become cancerous, gaining plasticity and uncontrolled replication^108^. Our model reasons that the SMT theory is more likely because SMT-supporting papers span multiple tumor types and methodologies (genomics, evolutionary analyses) and have a higher number of papers supporting it, whereas TOFT support is largely conceptual or from mechanical studies, ultimately giving a mean score of 88.6.

#### Cancer Origin

What is the primary origin of cancer? Whether cancer originates from cancer stem cells or through stochastic clonal evolution is currently being debated. Cancer stem cells are tumor cells that have stem-cell like properties such as the ability to self-renew, resistance to cytotoxicity, and multipotency which are thought to initiate cancer^109^. Cancer stem cells have been identified for many different types of cancers (breast cancer, lung cancer, gastric cancer, ovarian cancer, etc.) characterized by specific surface markers, enhanced DNA repair capacity, and plasticity that both initiate these cancers and help the tumors evade treatment^110–114^. supporting the cancer stem cell theory. In contrast, stochastic clonal evolution is similar to the somatic mutation theory, but in addition once a mutation occurs in a somatic cell, it may incur a selective advantage on the cell resulting in proliferation and cancer, therefore the origin of cancer occurs through positive selection of these cells^115^. Evidence for this theory includes instances of tumor heterogeneity that have arisen through distinct clonal populations, consistent with positive selection of individual cells^116,117^. Additionally, selective resistance of clonal populations to apoptosis supports that stochastic mutations driving this resistance are under positive selection and allow the progression of cancer^118^. With a mean of 87.4, our model has higher support for the cancer stem cell theory, indicating that while there is support for stochastic clonal evolution, in the literature this process often focuses on later resistance or hypermutated settings rather than primary initiation.

#### Pre-eclampsia

What causes the pathogenesis of pre-eclampsia? Two leading theories are that the pathogenesis originates from the placenta or the maternal cardiovascular system. Pre-eclampsia (PE) is characterized by hypertension and significant proteinuria on or after 20 weeks of pregnancy, and is a leading contributor to maternal mortality^119^. The placental hypothesis indicates that there is dysfunction in placental homeostasis, where there is inadequate trophoblast invasion (connection between the placenta and uterus) and thus incomplete remodeling of uterine spiral arteries. This leads to placental ischemia, hypoxia and thus oxidative stress, and then hypertension later in the pregnancy^119,120^. Single cell and spatial transcriptomics and metabolomics has revealed that PE placentas show an increase in placental macrophages, enrichment of transcriptomic pathways related to oxidative stress, and dysregulation of lipid and energy metabolism, supporting a placenta dysfunction hypothesis^120^. Additionally, other studies show gene or protein levels linked to trophoblast invasion from the placenta are altered in PE placentas, supporting the hypothesis that the placental defects are the main cause of PE^121,122^. In contrast the maternal cardiovascular hypothesis posits that there is a concurrent maladaptation of the maternal cardiovascular system along with abnormal placentation, which predisposes the mother to PE^123^. A large study (including > 187,000 women) found that women with pregnancy complications such as PE had many cardiometabolic risk factors, such as higher blood pressure, glucose levels, and body mass indices, 10 years before their pregnancy compared to women without complications^124^. An additional study linked high cardiovascular health with a lower risk of PE^125^. This supports that poor cardiometabolic health before pregnancy (and thus the development of the placenta) may contribute to developing PE. Some papers suggest that early and late onset of preeclampsia may have different causes, the early onset being the placenta-dominant form while the late onset being due to maternal cardiac maladaptation^126^. Our model finds more papers associated with placental defects as a cause of PE than cardiovascular dysfunction but acknowledges that cardiovascular health can be a contributing factor, giving a mean score of 85.7, which is in favor of the placental hypothesis.

#### Multiple Sclerosis

How does Multiple Sclerosis (MS) develop? MS is a chronic inflammatory disease that is characterized by demyelination of the central nervous system (CNS)^127^. Two main hypotheses exist for the cause and development of MS, one is that it is an autoimmune disorder, where the body’s own immune cells attack healthy myelin (the protective sheath around nerve fibers), while the other is that it is caused by the Epstein-Barr virus, which then triggers the immune system^127^. A study finding elevated levels of specific immune-related proteins had causal effects on risk of MS and that may be targeted to treat MS supports the hypothesis that it is an autoimmune disease^128^. Other evidence includes elevated levels of pro-inflammatory cytokines in response to dysregulated T-cells attacking the CNS in MS patients, and how treating inflammation through exercise can help MS symptoms^129^. In contrast, other studies claim that MS is initiated by a viral attack by the Epstein-Barr virus (EBV), where antigens from EBV mimic host antigens and cause dysregulation of T-cells and B-cells, which then results in autoimmunity^130^. Our model finds more references that support an autoimmunity causality that also detail immune-mediated mechanisms, while there are fewer abstracts supporting EBV as causal, and these are more based on associations. The model strongly tilts toward autoimmunity as the main cause of MS with a mean posterior score of 89.4.

#### Type 2 Diabetes

What is the primary defect in Type 2 Diabetes? Type 2 Diabetes mellitus (T2DM) is a chronic disease with high rates of morbidity that is characterized by a progressive loss of insulin secretion by beta cells in the pancreas that is not due to auto-immunity and the inability of tissues to respond to insulin^131,132^. It is unclear as to whether T2DM is ultimately caused by peripheral insulin resistance in tissues or pancreatic beta-cell failure. In the pancreatic beta cell failure hypothesis, due to high levels of glucose in the blood, pancreatic beta cells attempt to increase insulin secretion to maintain normal levels, but cannot secrete enough insulin, and their function declines. This leads to reduced glucose uptake by target tissues, hyperglycemia, and insulin resistance^132^. Therefore beta cell dysfunction or apoptosis could be the initiating factor in T2DM, supported by the fact that many people with T2DM have a reduced beta-cell mass compared to healthy individuals^133^. However, there are some indications that insulin resistance happens before pancreatic beta cell decline. Obesity has been shown to cause insulin deficiency by producing chronic low-grade inflammation, which can impair insulin signaling leading to hyperglycemia and ultimately the development of T2DM^132^, supporting an insulin resistance hypothesis. Some references state that T2DM is caused by a combination of the two hypotheses, dysfunctional pancreatic beta cells that do not secrete enough insulin and tissues not able to respond to insulin^134^. Our model acknowledges that there are an approximately even number of papers that support either hypothesis, and some that support both, so with a mean posterior score of 47.4 and a credible interval that overlaps the 50 mark, it concludes that both hypotheses are equally valid.

#### Atherosclerosis

What is the primary driver of atherosclerosis? Atherosclerosis is characterized by the build-up of plaque in the arteries, driven by the retention of apolipoprotein (apoB)-containing lipoproteins, particularly low-density lipoproteins (LDL), within the arterial wall^135^. According to the response-to-retention model, subendothelial trapping of these lipoproteins provokes a maladaptive inflammatory response that initiates and sustains plaque formation^136^. These two processes, lipid accumulation and inflammation are not competing hypotheses but interconnected mechanisms that operate synergistically^137,138^. The causal role of LDL is supported by the fact that humans with high LDL cholesterol have an increased risk of atherosclerotic cardiovascular disease,^139^ that animal studies demonstrate a high fat / high cholesterol diet causes atherosclerotic lesions^140^ and that drugs like statins and other lipid lowering therapies that lower LDL cholesterol reduce the risk of cardiovascular events^141^ The role of inflammation is supported by the CANTOS trial, which demonstrated that canakinumab, an anti-inflammatory therapy targeting interleukin-1β, reduced recurrent cardiovascular events independent of lipid-level lowering^142,143^. Additionally, patients with rheumatoid arthritis, an autoimmune disease characterized by systemic inflammation, have a 1.5- to 2-fold increased risk of coronary artery disease^144^. Our model finds that more papers between the years of 2020-2026 attribute the primary driver of atherosclerosis to inflammation than cholesterol. Our model gives a mean posterior score of 26.1, indicating that the underlying cause of atherosclerosis is more likely to be inflammation.

However, this finding likely reflects temporal trends in scientific discovery rather than a true hierarchy of causation. The lipid hypothesis of atherosclerosis predates the inflammation hypothesis by decades, atherosclerosis was viewed primarily as a lipid storage disease for much of the 20th century, with the role of inflammation only gaining prominence from the 1980s onward^145^. As a result, the foundational evidence supporting the causal role of LDL in atherosclerosis is well-established and has largely been translated into clinical practice through aggressive lipid-lowering therapies^146^. Importantly, current investigations of anti-inflammatory therapies for ASCVD are conducted as adjuncts to background statin and lipid-lowering therapy, not as replacements^146^. Thus, the model's finding of greater recent mechanistic attention to inflammation is consistent with the current scientific landscape, in which the LDL pathway is well-characterized and therapeutically addressed, while the inflammatory pathway represents an active and evolving area of investigation built upon a foundation of aggressive LDL reduction.

#### Parkinson’s Disease

Where does Parkinson’s Disease originate? Parkinson’s disease (PD) is a neurodegenerative disease that affects the ability to move and is characterized by tremors and rigidity^147^, as well as the accumulation of α-synuclein proteins in the central nervous system^148^. Competing hypotheses suggest the disease originates in the central nervous system (CNS) and spreads outward, or that the pathological proteins form in the enteric nervous system (ENS) and travel up to the brain^149^. In the CNS hypothesis, α-synuclein proteins accumulate in the amygdala or connected structure, then spread to the brain stem, and this is when motor symptoms appear^149^. Some neurons were found to be susceptible to accumulation of α-synuclein proteins because of dysfunction in their mitochondria, and mitochondrial dysfunction in the brain as well as mitochondrial gene mutations have been found in Parkison’s patients, supporting the CNS hypothesis^150^. The ENS hypothesis suggests that PD begins in the gut, and that gut dysfunction causes the marker protein α-synuclein to misfold, and then this protein travels up the vagus nerve to the brain. This is supported by the findings that misfolded α-synuclein proteins can be found in the ENS sometimes years before motor symptoms appear in PD patients, and that gut symptoms also appear before motor symptoms^147,151^. Additionally inoculation of the α-synuclein protein to the gut wall in aged mice results in the progression of this protein to the midbrain and resulting motor symptoms^152^. Our model finds more abstracts supporting the ENS hypothesis, while there is some evidence for the CNS hypothesis, it is also proposed that there could be two separate subtypes^153^. Thus, there is moderately more support for the ENS hypothesis, meriting a mean posterior score of 23.2.

#### Consciousness

Understanding the brain’s cognitive architecture may help unravel long-standing questions about how biological mechanisms give rise to intelligence, emotion, and behavioral decision-making. This area has gained increasing attention, as such insights could have far-reaching implications for the design of future AI systems. The Global Neuronal Workspace Theory (GNWT) (H1) proposes that the brain consists of many specialized, modular, unconscious processes, and that sufficiently strong stimuli trigger a rapid and sustained increase in neural activity that propagates from local sensory regions to a distributed network, linking frontal and posterior areas^154^. General anesthesia was found to decrease information integration across the brain with a sharp drop in connectivity. This suggests a breakdown in network communication, preventing the brain from broadcasting information and resulting in a transition to an unconscious state, supporting the GNW theory^155^. Multiple other studies^156–158^ also support this framework. In contrast, the more recent Integrated Information Theory (IIT) (H2) suggests that consciousness arises from the progressive integration of domain-specific macro-consciousness emerging from numerous micro-consciousness units. In a fMRI study, it was observed that the quantification and characterization of consciousness through a measure known as Φ decreased during anesthesia as predicted by IIT^159^. Additional studies^160,161^ provide supporting evidence for IIT, and while our analysis leans towards more support for the IIT theory of neural consciousness with a posterior score of 41.9, the credible interval score overlaps with 50, indicating both theories are equally likely.

#### Neural Code

How does the neuronal respond to stimuli and signaling responses among the network of neurons? While action potentials are primarily observed as a primary carrier of neural coding, the exact encoding mechanism in the action potential sequences is not yet resolved. This first hypothesis (H1) is the frequency or the rate that action potentials are fired, termed rate coding. This is believed to contain most of the information about the stimulus and explains muscle contractions^162^. Rate coding is robust against noise; averaging spikes over time allows the brain to ignore random errors in timing. Working memory is maintained through persistent neural firing in the prefrontal cortex even after a stimulus is no longer present, with mean firing rates directly reflecting the strength of the memory representation^163^. This supports rate coding theory, as it is the rate of neuronal discharge, not spike timing or patterns, that encodes and sustains information over time. However, the rate coding fails to explain the encoding mechanisms in case of faster reactions during visual scene changes. The competing ‘temporal coding’ hypothesis (H2) argues that precise spike timing is a significant element in neural coding^164^. While the rate coding model assumes the high frequency fluctuations in action potentials as noise, the temporal coding model suggests that they encode information. Millisecond-scale synchrony between molecular layer interneurons dictates superposition vs cancellation at target Purkinje-cells, showing precise spike timing carries functional information^165^. Additional studies^166–168^ provide evidence that precise spike timing, phase, or spike order conveys key information. Our analysis finds marginally higher support towards ‘temporal coding’ with a posterior score of 43.6; however, the credible interval spans the 50 mark, indicating neither hypothesis is dominant.

#### Schizophrenia

Schizophrenia is a chronic, severe mental disorder often characterized by hallucinations and a distorted perception of reality, along with other symptoms such as delusions and social withdrawal^169^. Although no single known cause has been identified, leading hypotheses suggest that dysregulation in dopamine (H1) or glutamate (H2) signaling pathways may contribute to the development of schizophrenia. Studies have found schizophrenia symptoms to be associated with abnormalities with dopamine regulation, including with dopamine receptors^170^. A random-effects meta-analysis of 49 in vivo PET studies involving 692 patients and 730 controls found significant regional and population heterogeneity in dopamine D2 and D3 receptors (D_2/3_) availability^171^. This primarily manifested as a moderate D_2/3_ increase among drug-free patients, whereas drug-treated patients showed a large decrease in D_2/3_ receptor availability. The study concluded that different dopamine subsystems in schizophrenia are selectively involved across brain regions. On the other hand, several studies^172–174^ suggest that glutamate may be a primary driver of schizophrenia. These included a meta-analysis on the metabolomics of schizophrenia patients that found dysregulated glutamate/glutamine pathways related to cognitive impairment in schizophrenia spectrum disorders^172^. Our analysis suggests that both hypotheses are approximately equal in likelihood, with the mean posterior score of 57.3 and the credible interval overlapping the 50-mark line.

#### Obesity

What is the primary cause of obesity? Obesity, defined by the accumulation of excessive fat and a body mass index ≥ 30 kg/m^2 175^. Current hypotheses for the cause of obesity are caloric surplus/calorie imbalance, where calories in exceed calories out, causing obesity or that obesity is caused by the quality of carbohydrates that are eaten, and high-glycemic carbohydrates trigger insulin that then causes fat storage. In support of the calorie imbalance hypothesis, studies in animals show that an increase in weight gain is caused by feeding animals high caloric diets, and that weight gain can be stopped by reducing caloric intake^176,177^. Another study found that daily caloric intake was the main determinant of childhood obesity^178^. In contrast to calorie imbalance, the second hypothesis indicates that the type of carbohydrate eaten can cause obesity. There is evidence from studies on children that excessive fructose consumption may contribute to obesity regardless of overall energy intake^179^. Additionally, other studies show diets high in whole grains and fiber are negatively associated with adiposity^180^. These studies support the hypothesis that the type of carbohydrate eaten affects weight change and thus low-complexity carbohydrates could be causal to obesity. Our model gives a posterior mean score of 79.9, acknowledging that while evidence for both hypotheses exist, about 5 times more papers support the calorie imbalance hypothesis than the low-quality carbohydrate hypothesis.

**Time needed for KM-GPT-DCH to correctly choose a hypothesis**

How much time needs to pass after a new hypothesis is presented before KM-GPT-DCH can determine if the newer or older hypothesis is more likely? For the hypotheses presented here, this period varies from 0-6 years after the first proposal. All these determination dates from KM-GPT-DCH are before the new hypothesis was either generally accepted correctly or rejected correctly by the scientific community and public. So, while KM-GPT-DCH is likely useful for speeding up the process of correct acceptance or rejection of a new hypothesis or statement, it may not be typically capable of effectively evaluating a brand-new hypothesis. It is probably more typical that some literature about the newer hypothesis **after** its initial publication needs to exist for KM-GPT-DCH to make a significant evaluation.

## Supplementary Methods

### The KM-GPT-DCH algorithm additional notes

Here, we use the simpler KM (KinderMiner) algorithm^181^ for the co-occurrence step, versus Serial KinderMiner. One could, in theory, use SKiM (Serial KinderMiner)^2^ to retrieve co-occurring documents of interest and then still perform the GPT (Generative Pretrained Transformer) step^4^ and DCH (Direct Comparison of Hypotheses) analysis on these documents. This could be useful if one wanted to generate hypotheses and then test competing generated hypotheses.

### Example KM-GPT-DCH prompt

The following is the prompt input to the LLM in the KM-GPT-DCH approach:

**Biomedical Abstracts** (verbatim):

PMID: XXX

Title: XX

Abstract: XX

===END OF ABSTRACT===

**Available PMIDs for citation:** XXX

**Task:**

Compare Hypothesis 1 vs Hypothesis 2 using ONLY the abstracts above that mention terms relevant to either hypothesis.

Classify each abstract, produce tallies, assign a continuous 0\100 score, and choose a decision.

**Hypothesis 1:**

Peptic ulcer is caused by bacterial infection.

**Hypothesis 2:**

Peptic ulcer is caused by psychological stress.

**Continuous Scoring Guidelines (0\100):**

**0:** Hypothesis 1 is **strongly refuted** by overwhelming and consistent evidence indicating that peptic ulcer is caused by bacterial infection. **Directly contradicts** the proposed outcome, while Hypothesis 2 is **strongly supported** by overwhelming and consistent evidence indicating that peptic ulcer is caused by psychological stress.

**Directly aligns with** the proposed outcome.

**25:** Hypothesis 2 (peptic ulcer is caused by psychological stress.) is three times more likely than Hypothesis 1 (peptic ulcer is caused by bacterial infection.).

**50:** Hypothesis 1 (peptic ulcer is caused by bacterial infection.) is equally likely compared to Hypothesis 2 (peptic ulcer is caused by psychological stress.).

**75:** Hypothesis 1 (peptic ulcer is caused by bacterial infection.) is three times more likely than Hypothesis 2 (peptic ulcer is caused by psychological stress.).

**100:** Hypothesis 1 is **strongly supported** by overwhelming and consistent evidence indicating that peptic ulcer is caused by bacterial infection. **Directly aligns with** the proposed outcome, while Hypothesis 2 is **strongly refuted** by overwhelming and consistent evidence indicating that peptic ulcer is caused by psychological stress. **Directly contradicts** the proposed outcome.

**Guidelines for Intermediate Scores:**

- **Proportional Scoring:** Assign scores between the defined anchor points based on the relative strength and consistency of the evidence. For example, a score of +66 would indicate approximately twice as much support for Hypothesis 1 compared to Hypothesis 2. In the case where there is no evidence for one of the Hypotheses, but there is supporting evidence for the other hypothesis, then this should generate a score in favor of the other hypothesis, the score value being dependent on the actual level of support for the other hypothesis. For instance, if there is no evidence at all for Hypothesis 2, and there are 10 abstracts that exhibit reasonably strong support for Hypothesis 1 (but do not provide absolutely unequivocal support for Hypothesis 1), then this should generate a score strongly in favor of Hypothesis 1, but not necessarily the max score of 100.
- **Avoid Clustering:** Do not disproportionately favor specific integer values unless the evidence explicitly warrants it. Ensure that scores are distributed across the range to reflect the nuanced degree of support or refutation.
- **Evidence-Based Justification:** Base the score on the cumulative evidence from the abstracts. Consider the number of abstracts supporting or refuting each hypothesis, the quality of the evidence, and the presence of any contradictory information.
- **Neutrality and Insufficiency:** A score close to **50** should be assigned when the evidence is inconclusive, mixed, or insufficient to determine the support of both hypotheses. Note that in the case where there is no evidence for Hypothesis 1 and no evidence for Hypothesis 2, this should generate a score of 50.
- **Consistent Reasoning:** Ensure that the reasoning provided for the score directly correlates with the assigned value, clearly demonstrating how the evidence leads to that specific score.

**Best Practices to Maintain Objectivity:**

- **Clear Definitions:** Utilize the anchor points to anchor your scoring decisions, ensuring each score has a clear and objective basis.
- Comprehensive Review: Thoroughly analyze all provided abstracts to capture the full scope of evidence before assigning a score.
- Unit Consistency: Keep the scoring consistent across different hypotheses by adhering strictly to these guidelines, avoiding subjective or arbitrary score assignments.

By following these continuous scoring guidelines, you can provide a nuanced and objective assessment of the support level for Hypothesis 1 compared to Hypothesis 2 based on the hypotheses that peptic ulcer is caused by bacterial infection and peptic ulcer is caused by psychological stress.

**Output policy:**

Return ONLY a single JSON object matching the schema below, inside a ```json code block.

JSON schema (for reference; do not print this schema):

{'type': 'object', 'additionalProperties': False, 'properties': {'per_abstract': {'type': 'array', 'items': {'type': 'object', 'additionalProperties': False, 'properties': {'pmid': {'type': 'string', 'pattern': '^[0-9]+$'}, 'label': {'type': 'string', 'enum': ['supports_H1', 'supports_H2', 'both', 'neither', 'inconclusive']}, 'evidence': {'type': 'array', 'items': {'type': 'string', 'maxLength': 300}}}, 'required': ['pmid', 'label']}}, 'score_rationale': {'type': 'array', 'items': {'type': 'string', 'maxLength': 1000, 'description': \"Evidence-based rationale with PMIDs, e.g., 'Two RCTs report X (PMID: 123, 456)' that uses the scoring guidelines to justify the score.\"}, 'minItems': 1, 'maxItems': 6}, 'tallies': {'type': 'object', 'additionalProperties': False, 'properties': {'support_H1': {'type': 'integer', 'minimum': 0}, 'support_H2': {'type': 'integer', 'minimum': 0}, 'both': {'type': 'integer', 'minimum': 0}, 'neither_or_inconclusive': {'type': 'integer', 'minimum': 0}}, 'required': ['support_H1', 'support_H2', 'both', 'neither_or_inconclusive']}, 'score': {'type': 'number', 'minimum': 0, 'maximum': 100}, 'decision': {'type': 'string', 'enum': ['H1', 'H2', 'tie', 'insufficient_evidence']}}, 'required': ['per_abstract', 'score_rationale', 'tallies', 'score', 'decision']}

### Determining the acceptance/refutation date for the hypotheses

Acceptance and refutation dates for the hypotheses discussed in this paper come from review of the literature and other available data. Acceptance by the scientific community is based on the following literature. For the case of HPV (human papillomavirus) and cervical cancer, the 1996 National Institute of Health (NIH) conference where HPV was accepted as a leading cause of cervical cancer is the most relevant piece of information^182^. For the case of Scrapie being caused by prions (protein infection), the acceptance date of 1997 is supported by this paper^183^. For the case of bacterial infection being a leading cause of peptic ulcer, the acceptance date of 1994 is supported by the NIH conference in that year when it was concluded that antibiotic treatment is crucial for treatment of peptic ulcer^184^. For the case of vaccines causing autism, that was refuted by the Lancet retraction in 2010^185^. For the case of hormone replacement therapy (HRT) being useful for prevention of cardiovascular disease, this was refuted by a large clinical trial in 2002^32^.

General public acceptance/refutation is difficult to assess but typically lags behind scientific acceptance. For the case of HPV causing cervical cancer, even as late as 2005, less than 20% of U.S. women were aware that HPV causes cervical cancer^186^. In general, men are less aware about HPV and cervical cancer^187^. General public awareness increased with the release of the Gardisil vaccine in 2006^188^, but awareness of the link between HPV and cervical cancer was still low as of 2007 with only 2.5% of British women aware that HPV causes cervical cancer^189^. Thus, we chose 2006 as a community “acceptance” date for HPV and cervical cancer, but the actual public awareness at this point was probably still low.

For the case of prions being infectious agents that cause diseases like scrapie, as of 2003, only 40% of university students were aware that prions cause mad cow disease^190^. General public awareness of the infectious agent being a non-living protein remains low as indicated by surveys of high-stakes individuals (hunters) in 2020-2022 indicate that only 21-38% of respondents were aware that prions cause CWD (chronic wasting disease)^191^. We set the public acceptance date as “2020+” to indicate that even as of 2020 or later many high-stakes individuals still were unaware that prions cause CWD. For the case of a bacterium causing peptic ulcer, as of 1997 only 27% of the U.S. public understood that a bacterial infection causes peptic ulcer^192^. It is difficult to find accurate surveys in the remaining several years. A 2012 study found low awareness of *H. pylori* (21-35%) amongst Chinese migrant workers^193^. However a 2024 study in which more than 50% had a college degree suggested that a high percentage (82.5%) of survey participants in Riyadh, Saudi Arabia were aware that *H. pylori* can cause peptic ulcer^194^. Thus, the acceptance rate will probably depend on education, socioeconomic status, and many other factors. We conservatively set the public acceptance date for the hypothesis that bacterial infection is a cause of peptic ulcer as “1997+”.

For the case of the incorrect claim that vaccines cause autism, the number of people that think vaccines cause autism is increasing in the U.S. A 2020 U.S. Gallup poll showed that 10% believe vaccines cause autism, and 46% are unsure if vaccines cause autism or not^195^. These percentages increase in the 2024 U.S. Gallup poll which showed 13% of people believe vaccines cause autism, and 51% are unsure^196^. Thus, we set the public refutation date for the claim of vaccines causing autism to be “?”.

For the case of the incorrect claim the HRT would be useful for preventing ASCVD in peri- or post-menopausal women, after the publication of the Women’s Health Initiative study in 2002^32^, there was substantial media coverage, but this resulted in only moderate understanding by the public with 29% of women being aware of the WHI results by 2004^197^. However, the response by clinicians was quick and substantial with the number of hormone therapy claims dropping by ~30% by the end of 2002. Claims continued to drop by 70% by 2009^198^. Of course, clinicians may not be representative of the public. Based on the information above, we set the public “refutation date” for HRT as a preventive treatment for ASCVD to be 2004+.

See **Table 1** for scientific acceptance dates for historical hypotheses, and **Supplemental Table 1** for scientific and public acceptance dates for historical hypotheses.

### Choosing A and B terms

Note that because KM uses an exact text matching search method, it is important to choose A and B terms to broadly capture the abstracts of interest. The user can choose multiple versions of terms using the pipe symbol (*e.g.* “peptic ulcer| gastric ulcer”). Care must be taken to not include synonyms that might be used in other contexts; however, the relevance filter will likely remove such abstracts before consideration by the frontier LLM. The number of abstracts retrieved and resulting analysis will depend on the A and B terms chosen. Because of the random sampling, if there is a large A-B abstract pool, different abstracts would be retrieved for different iterations (up to 50 per iteration), resulting in score variation. It is therefore recommended to run multiple iterations especially for a large pool of abstracts.

## Supplementary References

1. Moreau, E. Literature-based discovery: addressing the issue of the subpar evaluation methodology. *Bioinformatics* **39**, btad090 (2023).

2. Millikin, R. J. *et al.* Serial KinderMiner (SKiM) discovers and annotates biomedical knowledge using co-occurrence and transformer models. *BMC Bioinformatics* **24**, 412 (2023).

3. Mallen, A. *et al.* When Not to Trust Language Models: Investigating Effectiveness of Parametric and Non-Parametric Memories. in *Proceedings of the 61st Annual Meeting of the Association for Computational Linguistics (Volume 1: Long Papers)* 9802–9822 (Association for Computational Linguistics, Toronto, Canada, 2023). doi:10.18653/v1/2023.acl-long.546.

4. Freeman, J. *et al.* SKiM-GPT: combining biomedical literature-based discovery with large language model hypothesis evaluation. *BMC Bioinformatics* https://doi.org/10.1186/s12859-025-06350-7 (2025) doi:10.1186/s12859-025-06350-7.

5. Preiss, J. Avoiding background knowledge : literature based discovery from important information. *BMC Bioinformatics* 1–9 (2022) doi:10.1186/s12859-022-04892-8.

6. Mahaut, M. *et al.* Factual Confidence of LLMs: on Reliability and Robustness of Current Estimators. in 4554–4570 (2024). doi:10.18653/v1/2024.acl-long.250.

7. Yang, D., Tsai, Y.-H. H. & Yamada, M. On Verbalized Confidence Scores for LLMs. Preprint at https://doi.org/10.48550/ARXIV.2412.14737 (2024).

8. Tian, K. *et al.* Just Ask for Calibration: Strategies for Eliciting Calibrated Confidence Scores from Language Models Fine-Tuned with Human Feedback. in *Proceedings of the 2023 Conference on Empirical Methods in Natural Language Processing* (Association for Computational Linguistics, Singapore, 2023). doi:10.18653/v1/2023.emnlp-main.330.

9. Fomicheva, M. *et al.* Unsupervised Quality Estimation for Neural Machine Translation. *Transactions of the Association for Computational Linguistics* **8**, 539–555 (2020).

10. Kadavath, S. *et al.* Language Models (Mostly) Know What They Know. Preprint at https://doi.org/10.48550/arXiv.2207.05221 (2022).

11. Wang, X. *et al.* Self-Consistency Improves Chain of Thought Reasoning in Language Models. Preprint at https://doi.org/10.48550/ARXIV.2203.11171 (2022).

12. Azaria, A. & Mitchell, T. The Internal State of an LLM Knows When It’s Lying. in *Findings of the Association for Computational Linguistics: EMNLP 2023* (Association for Computational Linguistics, Singapore, 2023). doi:10.18653/v1/2023.findings-emnlp.68.

13. Zou, A. *et al.* Representation Engineering: A Top-Down Approach to AI Transparency. Preprint at https://doi.org/10.48550/ARXIV.2310.01405 (2023).

14. Bowyer, S., Aitchison, L. & Ivanova, D. R. Position: Don’t Use the CLT in LLM Evals With Fewer Than a Few Hundred Datapoints. Preprint at https://doi.org/10.48550/arXiv.2503.01747 (2025).

15. Longjohn, R., Wu, S., Kher, S., Belém, C. & Smyth, P. Bayesian Evaluation of Large Language Model Behavior. Preprint at https://doi.org/10.48550/arXiv.2511.10661 (2025).

16. Banerjee, S., Agarwal, A. & Singla, S. LLMs Will Always Hallucinate, and We Need to Live With This. Preprint at https://doi.org/10.48550/arXiv.2409.05746 (2024).

17. Liu, N. F., Zhang, T. & Liang, P. Evaluating Verifiability in Generative Search Engines. Preprint at https://doi.org/10.48550/arXiv.2304.09848 (2023).

18. Huang, L. *et al.* A Survey on Hallucination in Large Language Models: Principles, Taxonomy, Challenges, and Open Questions. *ACM Trans. Inf. Syst.* **43**, 1–55 (2025).

19. Dürst, M., Gissmann, L., Ikenberg, H. & zur Hausen, H. A papillomavirus DNA from a cervical carcinoma and its prevalence in cancer biopsy samples from different geographic regions. *Proc Natl Acad Sci U S A* **80**, 3812–3815 (1983).

20. Koenig, U. D., Haag, A. & Lehmk, null. [Seroepidemiological studies related to the association of genital herpes to cervical cancer (author’s transl)]. *Geburtshilfe Frauenheilkd* **35**, 909–913 (1975).

21. Chow, A. W. Genital Infection With Type 2 Herpes Simplex Virus: A Common Venereal Disease. *Postgraduate Medicine* **58**, 66–70 (1975).

22. Boshart, M. *et al.* A new type of papillomavirus DNA, its presence in genital cancer biopsies and in cell lines derived from cervical cancer. *The EMBO Journal* **3**, 1151–1157 (1984).

23. Huang, W. K. *et al.* [Campylobacter pyloridis in active chronic gastritis and peptic ulcer]. *Zhonghua Min Guo Wei Sheng Wu Ji Mian Yi Xue Za Zhi* **20**, 148–153 (1987).

24. Bode, G., Malfertheiner, P. & Ditschuneit, H. Invasion of campylobacter-like organisms in the duodenal mucosa in patients with active duodenal ulcer. *Klin Wochenschr* **65**, 144–146 (1987).

25. Lamouliatte, H., Megraud, F., de Mascarel, A., Roux, D. & Quinton, A. ‘Campylobacter pyloridis’ and epigastric pain: endoscopic, histological, and bacteriological correlations. *Gastroenterol Clin Biol* **11**, 212–216 (1987).

26. Malfertheiner, P., Bode, G., Stanescu, A. & Ditschuneit, H. [Campylobacter pylori in duodenal ulcer--a concomitant phenomenon or causal association?]. *Z Gastroenterol* **25 Suppl 3**, 112–118 (1987).

27. Langeluddecke, P., Goulston, K. & Tennant, C. Type A behaviour and other psychological factors in peptic ulcer disease. *J Psychosom Res* **31**, 335–340 (1987).

28. Adami, H. O. *et al.* Is duodenal ulcer really a psychosomatic disease? A population-based case-control study. *Scand J Gastroenterol* **22**, 889–896 (1987).

29. Magni, G. *et al.* Personality and duodenal ulcer response to antisecretory treatment. *Digestion* **38**, 152–155 (1987).

30. Stampfer, M. J. & Colditz, G. A. Estrogen Replacement Therapy and Coronary Heart Disease: A Quantitative Assessment of the Epidemiologic Evidence. *Preventitive Medicine* **20**, 47–63 (1991).

31. LaMonte, M. J. *et al.* Contributions of the Women’s Health Initiative to Cardiovascular Research. *Journal of the American College of Cardiology* **80**, 256–275 (2022).

32. Rossouw, J. & et.al. Risks and Benefits of Estrogen Plus Progestin in Healthy Postmenopausal Women: The Women’s Health Initiative. *JAMA-Express* **288**, 321–333 (2002).

33. Hulley, S. Randomized Trial of Estrogen Plus Progestin for Secondary Prevention of Coronary Heart Disease in Postmenopausal Women. *JAMA* **280**, 605 (1998).

34. Manson, J. E., Assaf, A. R., Heckbert, S. R. & Crouse, J. R. Estrogen plus Progestin and the Risk of Coronary Heart Disease. *n engl j med* (2003).

35. Anderson, J. G., Limacher, M. & et .al. Effects of conjugated equine estrogen in postmenopausal women with hysterectomy: the Women’s Health Initiative randomized controlled trial. *JAMA: The Journal of the American Medical Association* **291**, 1775–1776 (2004).

36. Cho, L. *et al.* Rethinking Menopausal Hormone Therapy: For Whom, What, When, and How Long? *Circulation* **147**, 597–610 (2023).

37. Bofill Rodriguez, M. *et al.* Long-term hormone therapy for perimenopausal and postmenopausal women. *Cochrane Database of Systematic Reviews* **2025**, (2025).

38. US Preventive Services Task Force *et al.* Hormone Therapy for the Primary Prevention of Chronic Conditions in Postmenopausal Persons: US Preventive Services Task Force Recommendation Statement. *JAMA* **328**, 1740 (2022).

39. Rossouw, J. E. *et al.* Postmenopausal Hormone Therapy and Risk of Cardiovascular Disease by Age and Years Since Menopause. *JAMA* **297**, (2007).

40. Hodis, H. N. & Mack, W. J. Menopausal Hormone Replacement Therapy and Reduction of All-Cause Mortality and Cardiovascular Disease: It Is About Time and Timing. *Cancer J* **28**, 208–223 (2022).

41. Pinkerton, J. V. Hormone Therapy for Postmenopausal Women. *N Engl J Med* **382**, 446–455 (2020).

42. Jemilohun, A. C. & Otegbayo, J. A. Helicobacter pylori infection: past, present and future. *Pan Afr Med J* **23**, (2016).

43. Warren, J. R. & Marshall, B. J. Unidentified curved bacilli on gastric epithelium in active chronic gastritis. *The Lancet* **321**, 1273–1275 (1983).

44. Marshall, B. J. & Goodwin, C. S. Revised Nomenclature of Campylobacter pyloridis. *International Journal of Systematic Bacteriology* **37**, 68–68 (1987).

45. Goodwin, C. S. *et al.* Transfer of Campylobacter pylori and Campylobacter mustelae to Helicobacter gen. nov. as Helicobacter pylori comb. nov. and Helicobacter mustelae comb. nov., Respectively. *International Journal of Systematic Bacteriology* **39**, 397–405 (1989).

46. Hotchin, J. & Buckley, R. Latent form of scrapie virus: a new factor in slow-virus disease. *Science* **196**, 668–671 (1977).

47. Dickinson, A. G. & Outram, G. W. Genetic Aspects of Unconventional Virus Infections: The Basis of the Virino Hypothesis. in *Novartis Foundation Symposia* (eds Bock, G. & Marsh, J.) 63–83 (Wiley, 2007). doi:10.1002/9780470513613.ch5.

48. Prusiner, S. B. Novel proteinaceous infectious particles cause scrapie. *Science* **216**, 136–144 (1982).

49. Zabel, M. D. & Reid, C. A brief history of prions. *Pathogens and Disease* **73**, 1–8 (2015).

50. Bessen, R. A. & Marsh, R. F. Biochemical and physical properties of the prion protein from two strains of the transmissible mink encephalopathy agent. *J Virol* **66**, 2096–2101 (1992).

51. Walker, L. C. & Jucker, M. The prion principle and Alzheimer’s disease. *Science* **385**, 1278–1279 (2024).

52. McAlary, L., Yerbury, J. J. & Cashman, N. R. The prion-like nature of amyotrophic lateral sclerosis. in *Progress in Molecular Biology and Translational Science* vol. 175 261–296 (Elsevier, 2020).

53. Seither, R. Coverage with Selected Vaccines and Exemption Rates Among Children in Kindergarten — United States, 2023–24 School Year. *MMWR Morb Mortal Wkly Rep* **73**, (2024).

54. Antaki, D. *et al.* A phenotypic spectrum of autism is attributable to the combined effects of rare variants, polygenic risk and sex. *Nat Genet* **54**, 1284–1292 (2022).

55. Bicks, L. K. & Geschwind, D. H. Functional neurogenomics in autism spectrum disorders: A decade of progress. *Current Opinion in Neurobiology* **86**, 102858 (2024).

56. Cirnigliaro, M. *et al.* The contributions of rare inherited and polygenic risk to ASD in multiplex families. *Proc. Natl. Acad. Sci. U.S.A.* **120**, e2215632120 (2023).

57. Weiner, D. J. *et al.* Statistical and functional convergence of common and rare genetic influences on autism at chromosome 16p. *Nat Genet* **54**, 1630–1639 (2022).

58. Iossifov, I. *et al.* The contribution of de novo coding mutations to autism spectrum disorder. *Nature* **515**, 216–221 (2014).

59. Rolland, T. *et al.* Phenotypic effects of genetic variants associated with autism. *Nat Med* **29**, 1671–1680 (2023).

60. Davidson, M. Vaccination as a cause of autism—myths and controversies. *Dialogues in Clinical Neuroscience* **19**, 403–407 (2017).

61. Taylor, L. E., Swerdfeger, A. L. & Eslick, G. D. Vaccines are not associated with autism: An evidence-based meta-analysis of case-control and cohort studies. *Vaccine* **32**, 3623–3629 (2014).

62. Litman, A. *et al.* Decomposition of phenotypic heterogeneity in autism reveals underlying genetic programs. *Nat Genet* 1–9 (2025) doi:10.1038/s41588-025-02224-z.

63. Leyhausen, J. *et al.* Parsing Autism Heterogeneity: Transcriptomic Subgrouping of Imaging-Derived Phenotypes in Autism. *Biological Psychiatry: Cognitive Neuroscience and Neuroimaging* https://doi.org/10.1016/j.bpsc.2025.07.001 (2025) doi:10.1016/j.bpsc.2025.07.001.

64. Kvetnoy, I. *et al.* Signaling Molecules and Diagnosis of Cognitive Disorders: Current State and Prospects. *Int J Mol Sci* **27**, 372 (2025).

65. Takomthong, P. *et al.* Kaempferia parviflora extract and its methoxyflavones as potential anti-Alzheimer assessing in vitro, integrated computational approach, and in vivo impact on behaviour in scopolamine-induced amnesic mice. *PLoS One* **20**, e0316888 (2025).

66. Thomas, R. *et al.* Exercise Training Results in Lower Amyloid Plaque Load and Greater Cognitive Function in an Intensity Dependent Manner in the Tg2576 Mouse Model of Alzheimer’s Disease. *Brain Sci* **10**, 88 (2020).

67. Ghisays, V. *et al.* PET evidence of preclinical cerebellar amyloid plaque deposition in autosomal dominant Alzheimer’s disease-causing Presenilin-1 E280A mutation carriers. *Neuroimage Clin* **31**, 102749 (2021).

68. Zhang, H. *et al.* Interaction between Aβ and Tau in the Pathogenesis of Alzheimer’s Disease. *Int J Biol Sci* **17**, 2181–2192 (2021).

69. Hulse, J., Maphis, N., Peabody, J., Chackerian, B. & Bhaskar, K. Virus-like particle (VLP)-based vaccine targeting tau phosphorylated at Ser396/Ser404 (PHF1) site outperforms phosphorylated S199/S202 (AT8) site in reducing tau pathology and restoring cognitive deficits in the rTg4510 mouse model of tauopathy. Preprint at https://doi.org/10.1101/2024.04.05.588338 (2024).

70. Hulse, J. P. *et al.* pS396/pS404 (PHF1) tau vaccine outperforms pS199/pS202 (AT8) in rTg4510 tauopathy model. *NPJ Vaccines* **10**, 94 (2025).

71. Kovacech, B. *et al.* Post hoc analysis of ADAMANT, a phase 2 clinical trial of active tau immunotherapy with AADvac1 in patients with Alzheimer’s disease, positive for plasma p-tau217. *Alzheimers Res Ther* **16**, 254 (2024).

72. Fonseca, C. S. *et al.* Tau accumulation and atrophy predict amyloid independent cognitive decline in aging. *Alzheimers Dement* **20**, 2526–2537 (2024).

73. Therriault, J. *et al.* Amyloid-dependent and amyloid-independent effects of Tau in individuals without dementia. *Ann Clin Transl Neurol* **8**, 2083–2092 (2021).

74. Benbow, S. J., Strovas, T. J., Darvas, M., Saxton, A. & Kraemer, B. C. Synergistic toxicity between tau and amyloid drives neuronal dysfunction and neurodegeneration in transgenic C. elegans. *Hum Mol Genet* **29**, 495–505 (2020).

75. Kong, Y., Shao, Y., Ren, C. & Yang, G. Endometrial stem/progenitor cells and their roles in immunity, clinical application, and endometriosis. *Stem Cell Res Ther* **12**, 474 (2021).

76. Lamceva, J., Uljanovs, R. & Strumfa, I. The Main Theories on the Pathogenesis of Endometriosis. *Int J Mol Sci* **24**, 4254 (2023).

77. E Said, S. & Miyamoto, D. Multi-region processing during sleep for memory and cognition. *Proc Jpn Acad Ser B Phys Biol Sci* **101**, 107–128 (2025).

78. Elliott, J. G., Metha, J. A., Hoyer, D. & Jacobson, L. H. Rapid eye movement sleep: Who needs it? Creativity mechanisms and psychiatric applications of REM sleep enhancement. *J Psychopharmacol* **39**, 1245–1257 (2025).

79. Schaefke, B., Li, J., Zhao, B., Wang, L. & Tseng, Y.-T. Slumber under pressure: REM sleep and stress response. *Prog Neurobiol* **249**, 102771 (2025).

80. Bakht, D. *et al.* Examining the French paradox: a systematic review of red wine consumption and cardiovascular risk. *Ann Med Surg (Lond)* **87**, 8757–8769 (2025).

81. Serio, F. *et al.* Moderate red wine intake and cardiovascular health protection: a literature review. *Food Funct* **14**, 6346–6362 (2023).

82. Mirhadi, E., Roufogalis, B. D., Banach, M., Barati, M. & Sahebkar, A. Resveratrol: Mechanistic and therapeutic perspectives in pulmonary arterial hypertension. *Pharmacol Res* **163**, 105287 (2021).

83. Succi, M., Coppola, F., Testa, B., Pellegrini, M. & Iorizzo, M. Alcohol or No Alcohol in Wine: Half a Century of Debate. *Foods* **14**, 1854 (2025).

84. Marumo, M. & Wakabayashi, I. [Antithrombotic Action of Alcohol in Relation to Platelet Function]. *Nihon Eiseigaku Zasshi* **79**, (2024).

85. Gurule, S. C. *et al.* Reproductive neuroendocrine defects programmed by prenatal testosterone treatment between gestational days 60-90 are amplified by postnatal obesity in sheep. *Front Physiol* **15**, 1436954 (2024).

86. Kumariya, S., Ubba, V., Jha, R. K. & Gayen, J. R. Autophagy in ovary and polycystic ovary syndrome: role, dispute and future perspective. *Autophagy* **17**, 2706–2733 (2021).

87. Szeliga, A. *et al.* Neuroendocrine Determinants of Polycystic Ovary Syndrome. *Int J Environ Res Public Health* **19**, 3089 (2022).

88. Fontana, G. A. & Gahlon, H. L. Mechanisms of replication and repair in mitochondrial DNA deletion formation. *Nucleic Acids Res* **48**, 11244–11258 (2020).

89. Grońska-Pęski, M., Srinivasa, A. & Evrony, G. D. Divergent somatic mutation patterns among human cerebellar neuron types. *bioRxiv* 2025.09.29.679392 (2025) doi:10.1101/2025.09.29.679392.

90. Ren, P., Dong, X. & Vijg, J. Age-related somatic mutation burden in human tissues. *Front Aging* **3**, 1018119 (2022).

91. Zhang, X. *et al.* Senescent Mesenchymal Stromal Cells Differentially Alter Adipogenesis in Adipose Tissue, Skeletal Muscle, and Bone Marrow. *Obesity (Silver Spring)* **34**, 652–661 (2026).

92. Jung, H.-G. *et al.* Control of aging-associated neurodegeneration via hypothalamic extracellular vesicles containing parathymosin. *Cell Rep* **44**, 116561 (2025).

93. Hsieh, M.-S. *et al.* Age-stratified genomic analyses reveal population-specific genetic determinants of exceptional longevity in Taiwan. *Geroscience* https://doi.org/10.1007/s11357-026-02222-x (2026).

94. P, P., Krishnakumar, S., Suresh, M., Sacrapani, L. & Venkatesh, V. Acupuncture as a Complementary Therapy in Conservative and Endodontic Procedures: A Narrative Review. *Cureus* **16**, e75768 (2024).

95. Sirucek, L., Ganley, R. P., Zeilhofer, H. U. & Schweinhardt, P. Diffuse noxious inhibitory controls and conditioned pain modulation: a shared neurobiology within the descending pain inhibitory system? *Pain* **164**, 463–468 (2023).

96. Wu, M.-M., Liu, X.-H., Wang, L.-J., Chen, X.-Y. & Li, Y.-X. Clinical observation on deep needling at Xiaguan (ST 7) with round sharp needle combined with plum-blossom needle for trigeminal neuralgia of wind and heat. *Zhongguo Zhen Jiu* **41**, 1089–1094 (2021).

97. Zhang, T. *et al.* Acupuncture effects of post-stroke thalamic pain: a systematic review and meta-analysis of randomized controlled trials. *Front Neurol* **16**, 1528956 (2025).

98. Qu, Z. *et al.* Electro-acupuncture inhibits C-fiber-evoked WDR neuronal activity of the trigeminocervical complex: Neurophysiological hypothesis of a complementary therapy for acute migraine modeled rats. *Brain Res* **1730**, 146670 (2020).

99. Sonnenschein, C. & Soto, A. M. Carcinogenesis explained within the context of a theory of organisms. *Progress in Biophysics and Molecular Biology* **122**, 70–76 (2016).

100. Vaux, D. L. In defense of the somatic mutation theory of cancer. *BioEssays* **33**, 341–343 (2011).

101. Martínez-Jiménez, F. *et al.* A compendium of mutational cancer driver genes. *Nat Rev Cancer* **20**, 555–572 (2020).

102. Melo, C. M. *et al.* The Role of Somatic Mutations on the Immune Response of the Tumor Microenvironment in Prostate Cancer. *IJMS* **22**, 9550 (2021).

103. Roosan, M. R. *et al.* Evaluation of Somatic Mutations in Solid Metastatic Pan-Cancer Patients. *Cancers* **13**, 2776 (2021).

104. Nelakurti, D. D., Pappula, A. L., Rajasekaran, S., Miles, W. O. & Petreaca, R. C. Comprehensive Analysis of MEN1 Mutations and Their Role in Cancer. *Cancers* **12**, 2616 (2020).

105. Kamdee, K. *et al.* Comprehensive germline and somatic profiling of high-risk Thai breast cancer via next-generation sequencing. *Sci Rep* **15**, 11427 (2025).

106. Davila, J. I. *et al.* Frequent POLE-driven hypermutation in ovarian endometrioid cancer revealed by mutational signatures in RNA sequencing. *BMC Med Genomics* **14**, 165 (2021).

107. Carvalho, J. A bioelectric model of carcinogenesis, including propagation of cell membrane depolarization and reversal therapies. *Sci Rep* **11**, 13607 (2021).

108. Hammarlund, E. U., Amend, S. R. & Pienta, K. J. The issues with tissues: the wide range of cell fate separation enables the evolution of multicellularity and cancer. *Med Oncol* **37**, 62 (2020).

109. Barjij, I. & Meliani, M. Cancer stem cells and post-therapy tumour recurrence: a systematic review of mechanistic pathways and translational gaps. *Ecancermedicalscience* **19**, 2016 (2025).

110. Li, W., Xue, H., Li, P. & Zhang, X. FOXP1-transcriptionally regulated AEG-1 enhances tumor cell stemness to promote hepatocellular carcinoma radioresistance. *Mol Cell Biochem* **481**, 547–559 (2025).

111. Huang, M. *et al.* SAA restricts T cell mediated anti-tumor immunity by limiting antigen presentation in lung cancer. *Front. Immunol.* **16**, 1735479 (2025).

112. Rao, X. *et al.* Targeting Gastric Cancer Stem Cells to Enhance Treatment Response. *Cells* **11**, 2828 (2022).

113. Liu, D., Yi, H. & Yi, C. Redox–Amino Acid Metabolic Crosstalk in Ovarian Cancer Stem Cells: Integrating Metabolic Reprogramming, Signaling, and the Tumor Microenvironment. *Antioxidants* **14**, 1413 (2025).

114. Quaglino, E., Conti, L. & Cavallo, F. Breast cancer stem cell antigens as targets for immunotherapy. *Seminars in Immunology* **47**, 101386 (2020).

115. Williams, M. J., Sottoriva, A. & Graham, T. A. Measuring Clonal Evolution in Cancer with Genomics. *Annu. Rev. Genom. Hum. Genet.* **20**, 309–329 (2019).

116. Chan, D. K. H. & Buczacki, S. J. A. Tumour heterogeneity and evolutionary dynamics in colorectal cancer. *Oncogenesis* **10**, 53 (2021).

117. Weideman, A. M. K., Wang, R., Ibrahim, J. G. & Jiang, Y. Canopy2: tumor phylogeny inference by bulk DNA and single-cell RNA sequencing. *bioRxiv* 2024.03.18.585595 (2024) doi:10.1101/2024.03.18.585595.

118. Lacy, M. S. & Jenner, A. L. Impact of Resistance on Therapeutic Design: A Moran Model of Cancer Growth. *Bull Math Biol* **86**, 43 (2024).

119. Steegers, E. A., Von Dadelszen, P., Duvekot, J. J. & Pijnenborg, R. Pre-eclampsia. *The Lancet* **376**, 631–644 (2010).

120. Chen, Y. *et al.* Subtype specific immune-metabolic reprogramming in preeclampsia revealed by multiomics and serum biomarkers. *Hypertens Res* **49**, 641–657 (2026).

121. Tan, L. *et al.* Placental trophoblast-specific overexpression of chemerin induces preeclampsia-like symptoms. *Clinical Science* **136**, 257–272 (2022).

122. Zhang, R. *et al.* PD-L1 enhances migration and invasion of trophoblasts by upregulating ARHGDIB via transcription factor PU.1. *Cell Death Discov.* **8**, 395 (2022).

123. Thilaganathan, B. Placental syndromes: getting to the heart of the matter. *Ultrasound Obstet Gynecol* **49**, 7–9 (2017).

124. Birnie, K. *et al.* Life course trajectories of maternal cardiovascular disease risk factors by obstetric history: a UK cohort study using electronic health records. *BMC Med* **23**, 91 (2025).

125. Mathew, V. *et al.* Genetic Risk and First-Trimester Cardiovascular Health Predict Hypertensive Disorders of Pregnancy in Nulliparous Women. *J Am Coll Cardiol* **85**, 1488–1500 (2025).

126. Matot, R. *et al.* Divergent profiles in early and late postpartum preeclampsia. *Pregnancy Hypertension* **43**, 101422 (2026).

127. Arachchige, A. S. P. M. *et al.* A review of multiple sclerosis: From pathophysiology to latest therapeutic advances. *AIMS Neurosci* **12**, 514–538 (2025).

128. Lin, J., Zhou, J. & Xu, Y. Potential drug targets for multiple sclerosis identified through Mendelian randomization analysis. *Brain* **146**, 3364–3372 (2023).

129. Hejazi, K., Rahimi, G. R. M. & Hofmeister, M. Effects of exercise training on some cytokine profiles in patients with multiple sclerosis: A systematic review and meta-analysis of randomized controlled trials. *Mult Scler Relat Disord* **107**, 106951 (2026).

130. Gupta, S., Reddy, V., Lodha, L. & Ashwini, M. A. Epstein‐Barr Virus (EBV) and Autoimmune Diseases: Pathogenic Mechanisms and Therapeutic Insights. *Journal of Medical Virology* **98**, e70785 (2026).

131. Galicia-Garcia, U. *et al.* Pathophysiology of Type 2 Diabetes Mellitus. *Int J Mol Sci* **21**, 6275 (2020).

132. Varra, F.-N., Theodosis-Nobelos, P., Varra, V.-K. & Varras, M. Mechanistic Insights into Antioxidant Interventions Targeting Obesity-Induced Oxidative Stress in the Pathogenesis and Complications of Type 2 Diabetes Mellitus. *Curr Issues Mol Biol* **47**, 1063 (2025).

133. Wysham, C. & Shubrook, J. Beta-cell failure in type 2 diabetes: mechanisms, markers, and clinical implications. *Postgrad Med* **132**, 676–686 (2020).

134. Xu, H. *et al.* Pancreatic β cell microRNA-26a alleviates type 2 diabetes by improving peripheral insulin sensitivity and preserving β cell function. *PLoS Biol* **18**, e3000603 (2020).

135. Manojlovic, M., Mahmoud, D., Pantic, M. & Taye Amogne, M. Genetic determinants of lipid metabolism in cardioprotection: From mechanisms to clinical practice. *Biomol Biomed* **26**, 872–884 (2025).

136. Ference, B. A., Graham, I., Tokgozoglu, L. & Catapano, A. L. Reprint of: Impact of Lipids on Cardiovascular Health. *Journal of the American College of Cardiology* **72**, 2980–2995 (2018).

137. Kong, P. *et al.* Inflammation and atherosclerosis: signaling pathways and therapeutic intervention. *Signal Transduct Target Ther* **7**, 131 (2022).

138. Zaman, S. *et al.* The Lancet Commission on rethinking coronary artery disease: moving from ischaemia to atheroma. *The Lancet* **405**, 1264–1312 (2025).

139. Anderson, S. & Botti, C. The genetics of autosomal dominant familial hypercholesterolemia. *J Am Assoc Nurse Pract* **36**, 136–142 (2024).

140. Puppala, S. *et al.* Hepatic transcript signatures predict atherosclerotic lesion burden prior to a 2-year high cholesterol, high fat diet challenge. *PLoS ONE* **17**, e0271514 (2022).

141. Masana, L., Ibarretxe, D. & Plana, N. Reasons Why Combination Therapy Should Be the New Standard of Care to Achieve the LDL-Cholesterol Targets: Lipid-lowering combination therapy. *Curr Cardiol Rep* **22**, 66 (2020).

142. Libby, P. Inflammation during the life cycle of the atherosclerotic plaque. *Cardiovasc Res* **117**, 2525–2536 (2021).

143. Ridker, P. M. *et al.* Antiinflammatory Therapy with Canakinumab for Atherosclerotic Disease. *N Engl J Med* **377**, 1119–1131 (2017).

144. Weber, B. N. *et al.* Novel Imaging Approaches to Cardiac Manifestations of Systemic Inflammatory Diseases. *Journal of the American College of Cardiology* **82**, 2128–2151 (2023).

145. Libby, P. & Hansson, G. K. From Focal Lipid Storage to Systemic Inflammation. *Journal of the American College of Cardiology* **74**, 1594–1607 (2019).

146. Ridker, P. M. *et al.* Inflammation and cholesterol as predictors of cardiovascular events among patients receiving statin therapy: a collaborative analysis of three randomised trials. *The Lancet* **401**, 1293–1301 (2023).

147. Armas, C. & Kaufman, P. Gut-Brain Axis: The Role of Gastrointestinal Issues in Parkinson’s Disease. *Integr Med (Encinitas)* **24**, 20–27 (2025).

148. Vascellari, S. *et al.* Gut Microbiota and Metabolome Alterations Associated with Parkinson’s Disease. *mSystems* **5**, e00561-20 (2020).

149. Horsager, J. *et al.* Brain-first versus body-first Parkinson’s disease: a multimodal imaging case-control study. *Brain* **143**, 3077–3088 (2020).

150. Ge, P., Dawson, V. L. & Dawson, T. M. PINK1 and Parkin mitochondrial quality control: a source of regional vulnerability in Parkinson’s disease. *Mol Neurodegener* **15**, 20 (2020).

151. Benvenuti, L. *et al.* Enteric Glia at the Crossroads between Intestinal Immune System and Epithelial Barrier: Implications for Parkinson Disease. *Int J Mol Sci* **21**, 9199 (2020).

152. Challis, C. *et al.* Gut-seeded α-synuclein fibrils promote gut dysfunction and brain pathology specifically in aged mice. *Nat Neurosci* **23**, 327–336 (2020).

153. Cicero, C. E. *et al.* Exploring the relation between REM sleep behavior disorder onset and striatal dopaminergic dysfunction in Parkinson’s Disease. *J Neurol* **272**, 75 (2024).

154. Mashour, G. A., Roelfsema, P., Changeux, J.-P. & Dehaene, S. Conscious Processing and the Global Neuronal Workspace Hypothesis. *Neuron* **105**, 776–798 (2020).

155. Liang, Z. *et al.* The EEG complexity, information integration and brain network changes in minimally conscious state patients during general anesthesia. *J Neural Eng* **20**, (2023).

156. Farisco, M. & Changeux, J.-P. About the compatibility between the perturbational complexity index and the global neuronal workspace theory of consciousness. *Neurosci Conscious* **2023**, niad016 (2023).

157. Hagger-Vaughan, N., Kolnier, D. & Storm, J. F. Non-apical plateau potentials and persistent firing induced by metabotropic cholinergic modulation in layer 2/3 pyramidal cells in the rat prefrontal cortex. *PLoS One* **19**, e0314652 (2024).

158. Zelmann, R. *et al.* Differential cortical network engagement during states of un/consciousness in humans. *Neuron* **111**, 3479-3495.e6 (2023).

159. Onoda, K., Miyauchi, S., Kan, S. & Akama, H. Decrease and recovery of integrated information Φ during anesthesia and sleep on human functional magnetic resonance imaging. *Neurosci Conscious* **2025**, niaf024 (2025).

160. Grigis, A. *et al.* Revisiting the standard for modeling functional brain network activity: Application to consciousness. *PLoS One* **19**, e0314598 (2024).

161. Leung, A., Cohen, D., van Swinderen, B. & Tsuchiya, N. Integrated information structure collapses with anesthetic loss of conscious arousal in Drosophila melanogaster. *PLoS Comput Biol* **17**, e1008722 (2021).

162. Enoka, R. M. & Duchateau, J. Rate Coding and the Control of Muscle Force. *Cold Spring Harb Perspect Med* **7**, a029702 (2017).

163. Li, S., Zhou, X., Constantinidis, C. & Qi, X.-L. Plasticity of Persistent Activity and Its Constraints. *Front Neural Circuits* **14**, 15 (2020).

164. Zhu, H. *et al.* Temporal coding carries more stable cortical visual representations than firing rate over time. *Nat Commun* **16**, 7162 (2025).

165. Fakharian, M. A., Taeckens, E. A., Vasserman, A. N., Shoup, A. M. & Shadmehr, R. Coordination of spike timing among the neurons of the cerebellum. *bioRxiv* 2025.12.03.692114 (2025) doi:10.64898/2025.12.03.692114.

166. Martel, A.-C. & Apicella, P. Temporal processing in the striatum: Interplay between midbrain dopamine neurons and striatal cholinergic interneurons. *Eur J Neurosci* **53**, 2090–2099 (2021).

167. Gabbiani, F., Preuss, T. & Dewell, R. B. Approaching object acceleration differentially affects the predictions of neuronal collision avoidance models. *Biol Cybern* **117**, 129–142 (2023).

168. Tabuchi, M. Dynamic neuronal instability generates synaptic plasticity and behavior: Insights from Drosophila sleep. *Neurosci Res* **198**, 1–7 (2024).

169. Dean, L. Schizophrenia. in *Medical Genetics Summaries* (eds Pratt, V. M. et al.) (National Center for Biotechnology Information (US), Bethesda (MD), 2012).

170. Simpson, E. H., Gallo, E. F., Balsam, P. D., Javitch, J. A. & Kellendonk, C. How changes in dopamine D2 receptor levels alter striatal circuit function and motivation. *Mol Psychiatry* **27**, 436–444 (2022).

171. Zhao, Z. *et al.* Resolving the heterogeneity of dopamine subsystems dysfunction in schizophrenia: a PET meta-analysis. *Schizophrenia (Heidelb)* **11**, 139 (2025).

172. Panganiban, K. J. *et al.* The cognitive metabolomic signatures in schizophrenia spectrum disorders: A systematic review. *Psychiatry Res* **353**, 116742 (2025).

173. Lian, K. *et al.* Identification of biomarkers and therapeutic targets of schizophrenia using glutamine metabolism. *Asian J Psychiatr* **114**, 104724 (2025).

174. Jeon, P. *et al.* Activity-dependent modulation of glutamate, glutamine and glutathione in first-episode schizophrenia: A 7T functional MRS study. *Schizophr Res* **285**, 339–348 (2025).

175. Chooi, Y. C., Ding, C. & Magkos, F. The epidemiology of obesity. *Metabolism* **92**, 6–10 (2019).

176. Almehmadi, K., Fourman, S., Buesing, D. & Ulrich-Lai, Y. M. Western diet-induced obesity interferes with the HPA axis-blunting effects of palatable food in male rats. *Physiol Behav* **270**, 114285 (2023).

177. Cantu, A. E. *et al.* Triterpenoid CDDO-EA Protects from Hyperglycemia, Hyperinsulinemia, and Obesity by Decreasing Energy Intake. *Int J Mol Sci* **26**, 5485 (2025).

178. Nobre, I. G. *et al.* Food consumption habits, gestational age and birth weight are predictive for children with excess weight: An analysis based on artificial neural network. *Nutr Health* **32**, 287–296 (2026).

179. Giussani, M., Lieti, G., Orlando, A., Parati, G. & Genovesi, S. Fructose Intake, Hypertension and Cardiometabolic Risk Factors in Children and Adolescents: From Pathophysiology to Clinical Aspects. A Narrative Review. *Front Med (Lausanne)* **9**, 792949 (2022).

180. Zamanillo-Campos, R. *et al.* Longitudinal association of dietary carbohydrate quality with visceral fat deposition and other adiposity indicators. *Clin Nutr* **41**, 2264–2274 (2022).

181. Kuusisto, F. *et al.* A Simple Text Mining Approach for Ranking Pairwise Associations in Biomedical Applications. *AMIA Jt Summits Transl Sci Proc* **2017**, 166–174 (2017).

182. Braly, P. National Institutes of Health Consensus Development Conference Statement on Cervical Cancer. *Gynecologic Oncology* **66**, 351–361 (1997).

183. Josefson, D. The prion hypothesis is finally accepted by the establishment. *BMJ* **315**, 967–972 (1997).

184. Yamada, T. *et al.* Helicobacter pylori in Peptic Ulcer Disease. *JAMA* **272**, 65–69 (1994).

185. Rao, T. S. S. & Andrade, C. The MMR vaccine and autism: Sensation, refutation, retraction, and fraud. *Indian Journal of Psychiatry* **53**, 95–96 (2011).

186. Tiro, J. A., Meissner, H. I., Kobrin, S. & Chollette, V. What do women in the U.S. know about human papillomavirus and cervical cancer? *Cancer Epidemiol Biomarkers Prev* **16**, 288–294 (2007).

187. Davis, E. N. & Doyle, P. C. An Assessment of Young Adults’ Awareness and Knowledge Related to the Human Papillomavirus (HPV), Oropharyngeal Cancer, and the HPV Vaccine. *Cancers (Basel)* **17**, 344 (2025).

188. Gollust, S. E., Attanasio, L., Dempsey, A., Benson, A. M. & Fowler, E. F. Political and News Media Factors Shaping Public Awareness of the HPV Vaccine. *Women’s Health Issues* **23**, e143–e151 (2013).

189. Marlow, L. A. V., Waller, J. & Wardle, J. Public awareness that HPV is a risk factor for cervical cancer. *Br J Cancer* **97**, 691–694 (2007).

190. Harakeh, S. *et al.* Knowledge, attitude, and behavior of students regarding ‘mad cow disease’. *Science of The Total Environment* **312**, 255–260 (2003).

191. Vaske, J. J. & Miller, C. A. Knowledge, Beliefs, Agency Trust, and Perceived Risks Associated With Chronic Wasting Disease Among Kansas, North Dakota, and Illinois Deer Hunters. *Environment and Behavior* **56**, 516–541 (2024).

192. Centers for Disease Control and Prevention (CDC). Knowledge about causes of peptic ulcer disease -- United States, March-April 1997. *Morbidity and mortality weekly report* **46**, 985–987 (1997).

193. Xia, P., Ma, M.-F. & Wang, W. Status of Helicobacter pylori Infection among Migrant Workers in Shijiazhuang, China. *Asian Pacific Journal of Cancer Prevention* **13**, 1167–1170 (2012).

194. Abdelrahman, H. H. *et al.* Awareness and Practice of Patients with Helicobacter pylori Infection Toward their Disease in the Riyadh Region, Saudi Arabia. *Journal of Pharmacy and Bioallied Sciences* **16**, S473–S476 (2024).

195. Gallup, Inc. Fewer in U.S. Continue to See Vaccines as Important. *Gallup.com* https://news.gallup.com/poll/276929/fewer-continue-vaccines-important.aspx (2020).

196. Gallup, Inc. Far Fewer in U.S. Regard Childhood Vaccinations as Important. *Gallup.com* https://news.gallup.com/poll/648308/far-fewer-regard-childhood-vaccinations-important.aspx (2024).

197. Rigby, A. J., Ma, J. & Stafford, R. S. Women’s awareness and knowledge of hormone therapy post-Women’s Health Initiative. *Menopause* **14**, 853–858 (2007).

198. Ettinger, B. *et al.* Evolution of postmenopausal hormone therapy between 2002 and 2009. *Menopause* **19**, 610–615 (2012).

## Other Supplementary files

**Supplementary Table 1:** Acceptance/ Refutation dates of Historical Hypotheses and KM-GPT-DCH resolution dates.

**Supplementary Table 2:** Summary of reference evaluations by LLM-interfaces for pre-study time cut-offs (sheet1) and post-study time cut-offs (sheet 2).

**Supplementary Table 3:** Summary of reference evaluations by Robin, a multi-agent system for literature review using Crow interface.

**Supplementary Table 4:** Summary of current controversial or competing hypotheses that were evaluated using KM-GPT-DCH.

**Supplementary Data 1**: ***Detailed Q and A web-based LLMs.*** All questions and answers posed to and given by the chat-based LLMs and Robin for evaluation that are summarized in Supplementary Tables 2 and 3.

**Supplementary Data 2: *All KM-GPT-DCH outputs.*** All responses for every time point and iteration from KM-GPT-DCH for each set of hypotheses discussed. Zenodo DOI: **10.5281/zenodo.20547913.**
