# Supplementary material for: Quantifying Evidence for Competing Biomedical Hypotheses using Large Language Models and Bayesian Analysis
Source: bioRxiv. 2026 Jun 7:2026.06.05.730173. Preprint. [Version 1] doi: 10.64898/2026.06.05.730173 (PMC13251926; doi:10.64898/2026.06.05.730173)
Supplement: Supplement 6 [file media-6.docx]

**Detailed questions and responses from web-based LLMs in evaluating competing hypotheses**

Below are the responses from each LLM including the references they use, starting with the prior to the study cut-off date and then the post-study cut-off date.

## Question- response to LLMs about cervical cancer being caused by HPV or HSV prior to 1982

### Question:

Please evaluate the following competing hypotheses using only information from 1981 or before. Please provide references showing where you got this information. Here are the hypotheses: "Is it more likely that cervical cancer is caused by Human Papillomavirus or by Herpes Simplex virus?"

### Response from ChatGPT

As of 1981, the dominant hypothesis in the scientific community was that **Herpes Simplex Virus (HSV)**—particularly **HSV-2**—was more likely associated with cervical cancer than Human Papillomavirus (HPV). This view was based on a number of epidemiological and laboratory studies available at the time. The role of **HPV as a primary causative agent** of cervical cancer was **not yet well established** in 1981.

1. Evidence Supporting the HSV Hypothesis (Pre-1981)

During the 1970s and early 1980s, multiple studies found **correlations between HSV-2 infection and cervical cancer**, leading researchers to hypothesize a causal role.

- **Nahmias et al. (1970)** suggested a relationship between HSV-2 and cervical cancer due to higher antibody titers in women with cervical dysplasia and carcinoma:

   *Nahmias, A. J., Dowdle, W. R., Clarke, L. M., et al. (1970). Genital herpes simplex virus infections: studies on the role of virus in the etiology of cervical carcinoma. Cancer Research, 30(8), 2173–2176.*
- **Rawls et al. (1968)** found that HSV-2 DNA sequences were sometimes present in cervical cancer biopsies:

   *Rawls, W. E., Tompkins, W. A. F., Melnick, J. L., & Hill, W. B. (1968). Herpesvirus type 2: association with carcinoma of the cervix. Science, 161(3837), 1255–1256. https://doi.org/10.1126/science.161.3837.1255*
- **Kaufman et al. (1973)** and others reported epidemiological links:

   *Kaufman, R. H., Adam, E., & Reeves, W. C. (1973). Herpesvirus type 2 and cervical neoplasia. American Journal of Obstetrics and Gynecology, 116(4), 573–580.*

These studies showed **serological associations and some laboratory data**, suggesting HSV might have an oncogenic effect, though a direct mechanism was unclear.

2. HPV Hypothesis Status in 1981

As of 1981, **HPV had been identified**, and some researchers had begun to suspect it might play a role in cervical cancer, but **clear epidemiological or mechanistic links were not yet established** in the way they would be later in the 1980s.

- **Zur Hausen**, who would later win the Nobel Prize for establishing HPV's role in cervical cancer, had begun investigating HPV in the 1970s but had not yet produced conclusive evidence by 1981:

   *zur Hausen, H., Gissmann, L., Steiner, W., Dippold, W., & Dreger, I. (1975). Human papilloma viruses and cancer. Bibl Haematol, (43), 569–571.*
- The key breakthroughs came **after 1981**, particularly with the cloning of HPV-16 and HPV-18 in the mid-1980s.

Summary of 1981-Era Likelihood

**As of 1981, it was more likely—according to the prevailing scientific consensus—that Herpes Simplex Virus was associated with cervical cancer than HPV.** This was primarily due to:

- More robust epidemiological data available on HSV.
- Early-stage investigations of HPV with insufficient evidence.
- Lack of molecular tools at the time to adequately study HPV DNA integration, which would become key evidence in the 1980s.

References

1. Rawls, W. E., Tompkins, W. A. F., Melnick, J. L., & Hill, W. B. (1968). Herpesvirus type 2: association with carcinoma of the cervix. *Science*, 161(3837), 1255–1256. https://doi.org/10.1126/science.161.3837.1255
2. Nahmias, A. J., Dowdle, W. R., Clarke, L. M., et al. (1970). Genital herpes simplex virus infections: studies on the role of virus in the etiology of cervical carcinoma. *Cancer Research*, 30(8), 2173–2176.
3. Kaufman, R. H., Adam, E., & Reeves, W. C. (1973). Herpesvirus type 2 and cervical neoplasia. *American Journal of Obstetrics and Gynecology*, 116(4), 573–580.
4. zur Hausen, H., et al. (1975). Human papilloma viruses and cancer. *Bibl Haematol*, (43), 569–571.

Let me know if you’d like details from any of these studies or information on how the HPV hypothesis eventually overtook HSV in the scientific consensus.

### Comments on above:

Searching for the references by both Google scholar and PubMed, we find that most of the articles cited here are either made up, have incorrect citations, or are irrelevant.

1. Your search - Nahmias, A. J., Dowdle, W. R., Clarke, L. M., et al. (1970). Genital herpes simplex virus infections: studies on the role of virus in the etiology of cervical carcinoma. Cancer Research, 30(8), 2173–2176. - **did not match any articles**.
   1. There is a Nahmias 1971 article in Science about HSV-2 in monkeys and using them as a model in studying cervical cancer: [DOI: 10.1126/science.171.3968.297](https://doi.org/10.1126/science.171.3968.297). There is also a 1973 article by Nahmias in Cancer Research about the correlation of HSV and cervical anaplasia: PMID: 4352387. There is also a 1974 article: André J. Nahmias, Zuher M. Naib, William E. Josey; Epidemiological Studies Relating Genital Herpetic Infection to Cervical Carcinoma^1^. Cancer Res 1 May 1974; 34 (5): 1111–1117. It appears that this one from ChatGPT, however, is made up.
2. Rawls, W. E., Tompkins, W. A. F., Melnick, J. L., & Hill, W. B. (1968). Herpesvirus type 2: association with carcinoma of the cervix. Science, 161(3837), 1255–1256. https://doi.org/10.1126/science.161.3837.1255. - **this article exists.**
   1. This is a correct article that does associate HSV with cervical cancer, however the doi is slightly off and should be: https://doi.org/10.1126/science.161.3847.1255
3. Your search - Kaufman, R. H., Adam, E., & Reeves, W. C. (1973). Herpesvirus type 2 and cervical neoplasia. American Journal of Obstetrics and Gynecology, 116(4), 573–580. - **did not match any articles**.
   1. There is this study: RAWLS, WILLIAM E. MD; KAUFMAN, RAYMOND H. MD; GARDNER, HERMAN L. MD. Relation of Herpesvirus Type 2 to Carcinoma of the Cervix. Clinical Obstetrics and Gynecology 15(4):p 919-928, December 1972.
4. zur Hausen, H., et al. (1975). Human papilloma viruses and cancer. Bibl Haematol, (43), 569–571.
   1. is a book chapter but **the book name is wrong**, should be: Current Studies in Hematology and Blood Transfusion and was published in 1976. Thus this reference is **incorrect.**

### Response from Gemini:

Evaluating Competing Hypotheses for Cervical Cancer Etiology: Human Papillomavirus vs. Herpes Simplex Virus (Pre-1982)

**I. Executive Summary**

By 1981, the scientific understanding of cervical cancer's viral etiology stood at a critical juncture, marked by the gradual re-evaluation of the long-standing Herpes Simplex Virus Type 2 (HSV-2) hypothesis and the rapid emergence of the Human Papillomavirus (HPV) theory. While HSV-2 had been the prevailing candidate, supported by robust seroepidemiological associations and its venereal transmission pattern, its direct causal claim was significantly undermined by the persistent inability to consistently detect its DNA within cervical tumor cells. This molecular "absence" posed a fundamental challenge to its direct oncogenic role, suggesting its association might be a covariable of sexual behavior rather than a direct cause. Simultaneously, the HPV hypothesis, championed by Harald zur Hausen since 1976, was gaining substantial momentum. Although definitive molecular proof linking specific high-risk HPV types directly to cervical cancer was still forthcoming (occurring after 1981), the hypothesis was bolstered by several compelling lines of evidence. These included zur Hausen's critical negative findings for HSV-2 DNA, the clear clinical association between HPV and genital warts, and the increasingly recognized significance of koilocytotic atypia as a specific cytological marker of papillomavirus infection in precancerous cervical lesions. The successful molecular cloning of HPV6 from genital warts in 1981 represented a crucial technical advancement, opening new avenues for precise molecular investigations of genital HPV types. Considering the available information strictly up to 1981, the balance of evidence was shifting, making it more likely that Human Papillomavirus (HPV) was the primary causative agent of cervical cancer than Herpes Simplex Virus (HSV-2). The scientific community was actively engaged in a paradigm shift, driven by new molecular techniques and a re-evaluation of causality criteria, moving away from a hypothesis challenged by its own molecular inconsistencies towards one with growing, albeit still indirect, molecular and strong cytological support. This period represents a dynamic scientific environment where new technologies and persistent inquiry were actively reshaping long-held beliefs.

**II. Introduction: The Quest for Cervical Cancer's Viral Origin**

**Historical Context: Cervical Cancer as a Suspected Sexually Transmitted Disease**

For many decades prior to 1981, clinical and epidemiological observations strongly suggested that cervical cancer behaved as a sexually transmitted disease.^1^ This foundational understanding was rooted in consistent findings from epidemiological studies worldwide. Key risk factors repeatedly identified included early age at first coitus and having multiple sex partners.^5^ These factors consistently supported the concept that the development of cervical cancer was linked to a venereal disease.^5^ The number of marriages was often used as an indirect measure to reduce potential cultural bias in ascertaining the number of sex partners outside marriage.^5^ The consistent epidemiological link to sexual activity served as the fundamental premise guiding the search for a sexually transmitted viral agent. This context is crucial because it explains why viruses like HSV and HPV, known to be sexually transmitted, became the primary suspects in the etiology of cervical cancer. It established the initial biological plausibility for a viral connection.

**Introduction of the Two Primary Viral Candidates: Herpes Simplex Virus Type 2 (HSV-2) and Human Papillomavirus (HPV)**

By the late 1970s and early 1980s, Herpes Simplex Virus Type 2 (HSV-2) was firmly established as the leading candidate for the viral etiology of cervical cancer.^1^ This prevailing hypothesis was largely substantiated by a significant body of seroepidemiological data.^1^ However, an alternative hypothesis, which proposed Human Papillomavirus (HPV) as the causative agent, was gaining increasing scientific attention and momentum.^1^ This theory was notably championed by German virologist Harald zur Hausen, who formally published his hypothesis in 1976.^1^ The simultaneous presence of a prevailing hypothesis (HSV-2) and an emerging alternative (HPV) signified a period of intense scientific contention and methodological evolution, setting up the comparative evaluation as a historical scientific debate where established beliefs were being challenged by new observations and experimental approaches.

**The Broader Scientific Landscape of Viral Oncogenesis as Understood in the Late 1970s and Early 1980s**

The concept of viruses causing cancer, known as oncoviruses, was already established by this period. Landmark discoveries, such as the avian sarcoma leukosis virus (Rous sarcoma) in chickens in 1910-1911 and the Epstein-Barr virus (EBV) identified in Burkitt lymphoma in 1964, provided a theoretical and empirical foundation for investigating viral involvement in human cancers.^12^ This provided a crucial framework for researchers exploring cervical cancer etiology.^6^ A critical criterion for establishing viral oncogenesis, particularly emphasized by zur Hausen, was the expectation that the viral genome should be persistently present and transcriptionally active within the tumor cells.^1^ This implied that the viral DNA might exist in a non-productive state, meaning it would not be actively replicating or producing infectious virus particles, and thus would only be detectable through specific searches for viral DNA.^1^ This criterion profoundly influenced the molecular approaches taken to investigate both HSV and HPV. The understanding of viral oncogenesis was still evolving, particularly concerning how DNA viruses like HSV and HPV might cause cancer without necessarily producing infectious particles in the tumor. Zur Hausen's causality criteria were forward-thinking and critical for shaping the molecular investigations that followed, especially for HPV. This highlights the methodological challenges and conceptual shifts occurring in the field of viral oncology, which directly impacted the interpretation of evidence for both hypotheses.

III. The Herpes Simplex Virus Type 2 (HSV-2) Hypothesis: Evidence and Challenges by 1981

**Epidemiological and Seroepidemiological Associations**

By 1981, numerous seroepidemiological investigations had repeatedly demonstrated a significant excess of antibodies to HSV-2 among women diagnosed with cervical cancer when compared to control groups.^1^ For instance, a 1981 study indicated a higher frequency of previous HSV-2 infection in women with cervical cancer, with 85% of cancer patients' sera containing type-specific HSV-1 antibody, and 100% of matched controls also having HSV-1 antibodies, but the key finding was the higher frequency of HSV-2 antibodies in cancer patients.^14^ This consistent finding across multiple studies lent strong statistical support to an association between HSV-2 exposure and cervical cancer risk.

HSV-2 is a venereally transmitted infection.^5^ Its prevalence was found to correlate strongly with established epidemiological risk factors for cervical cancer, such as multiple marriages (used as an indirect measure of multiple sex partners) and early age at first coitus.^5^ The alignment of HSV-2's transmission patterns with the known sexual epidemiology of cervical cancer significantly bolstered its perceived role as a causative agent.^5^ Studies observed a correlation between the age-adjusted occurrence of HSV-2 antibodies among control women and the incidence of cervical cancer across different populations.^5^ For example, black women in Houston, Texas, exhibited both high cervical cancer rates (85.1 per 100,000) and a high prevalence of HSV-2 antibodies (60%), while women in London, England, showed significantly lower rates (9.3 per 100,000 cancer rate and 7.4% HSV-2 antibody prevalence).^5^ This geographical concordance further supported the epidemiological link. Epidemiological studies indicated a plausible temporal sequence, demonstrating that the highest age prevalence for genital herpes occurred several years before that for cervical dysplasia, in situ cancer, and invasive cancer.^15^ This observation suggested that HSV-2 infection could precede and potentially initiate the neoplastic process. The pervasive seroepidemiological association and its strong correlation with established sexual risk factors made HSV-2 the most plausible viral candidate based on epidemiological data alone by 1981. The consistency of these findings across diverse populations amplified its perceived importance and established it as the prevailing hypothesis.

**Biological Plausibility and Experimental Observations**

A critical criterion for a causal agent is its ability to directly affect the target tissue. Studies confirmed that the cervix, particularly the squamocolumnar junction (the site where cervical neoplasms most often develop), is a common site of both primary and recurrent HSV infection.^15^ This demonstrated the virus's ability to involve the relevant anatomical location. The HSV-2 hypothesis included the idea that specific strains of HSV might possess differing oncogenic potential.^15^ Experimental models provided evidence of the oncogenicity of HSV-1 ^15^, suggesting a plausible mechanism for HSV-2. Furthermore, HSV infections were known to lead to unscheduled cellular DNA synthesis, chromosomal amplifications, and mutations ^3^, all of which are cellular changes consistent with the initiation of neoplastic transformation. HSV was observed to replicate preferentially in squamous cervical cells.^15^ This observation was considered highly significant, given that approximately 95% of cervical cancers are squamous cell carcinomas.^15^ This tropism suggested a direct biological fit for HSV-2 in the pathogenesis of the most common form of cervical cancer. The biological plausibility and experimental evidence, while not direct proof of human carcinogenesis, provided crucial mechanistic support for the HSV-2 hypothesis by demonstrating the virus's ability to infect relevant cells and induce cellular changes consistent with oncogenesis in in vitro or animal models.^3^ This moved the hypothesis beyond mere correlation, offering a potential pathway for how the virus might contribute to cancer development.

**Limitations and Counter-Evidence**

A significant and growing challenge to the HSV-2 hypothesis was the inconsistent or failed detection of HSV-2 DNA sequences in cervical tumor tissues.^1^ Harald zur Hausen, a leading virologist, notably reported repeated failures to find HSV-2 DNA in cervical cancer cells using nucleic acid hybridization techniques.^1^ This was particularly impactful because zur Hausen had successfully applied the same molecular methods to identify Epstein-Barr virus in other cancer types, such as transformed lymphoblastoid cells, Burkitt's lymphoma, and nasopharyngeal carcinomas.^1^ This lack of consistent viral DNA presence in the tumor cells directly contradicted a key tenet of viral oncogenesis—that the viral genome should be persistently present and transcriptionally active in transformed cells.^1^ This molecular void was a major scientific hurdle and a critical contradiction to the prevailing hypothesis, introducing significant doubt about HSV-2's direct causal role and opening the door for alternative theories. It highlighted a growing disconnect between epidemiological associations and molecular evidence.

Even within the studies supporting an association, there was an acknowledgment that the link between cervical cancer and HSV-2 could represent either a direct causal relationship or a situation where HSV-2 infection was merely a "covariable of sexual behavior," associated with exposure to some other venereally transmitted carcinogenic factor.^5^ This nuance raised the possibility that HSV-2 might act as a cofactor, contributing to carcinogenesis, rather than being the sole or primary causal agent.^3^ Observations that continued viral gene expression was not apparently necessary for neoplastic transformation, coupled with the nature of HSV infections leading to unscheduled DNA synthesis and chromosomal changes, led some researchers to suggest that HSV-2 was "not a typical DNA tumor virus".^3^ This implied that its mechanism of oncogenesis, if any, might be indirect or different from established viral oncogenes.

**Table 1: Summary of Evidence for HSV-2 in Cervical Cancer (Pre-1982)**

| **Type of Evidence** | **Key Findings Supporting HSV-2 Hypothesis (Pre-1982)** | **Key Findings Challenging HSV-2 Hypothesis (Pre-1982)** |
| --- | --- | --- |
| **Epidemiological/ Seroepidemiological** | - High prevalence of HSV-2 antibodies in cervical cancer patients.  - Strong correlation with venereal risk factors (multiple partners, early coitus).  - Temporal relationship (genital herpes preceding cancer). | - Association could be a covariable of sexual behavior, not direct causality.  - Mixed results from epidemiological studies, some concluding HSV-2 not a major risk factor. |
| **Biological/ Molecular** | - Ability to infect cervical cells and squamocolumnar junction.  - Oncogenic potential in experimental models (e.g., HSV-1).  - Preferential replication in squamous cervical cells.  - Induces unscheduled DNA synthesis, chromosomal changes. | - Inconsistent or failed detection of HSV-2 DNA in cervical tumor tissues.  - Not considered a "typical DNA tumor virus"; continued viral gene expression not necessary for transformation. |

**IV. The Emerging Human Papillomavirus (HPV) Hypothesis: Early Insights by 1981**

**Harald zur Hausen's Paradigm Shift**

Harald zur Hausen embarked on a research program from 1977 to 1987 specifically to investigate whether HPV caused cervical cancer, thereby directly challenging the then-accepted HSV-2 hypothesis.^1^ His initial work at the University of Erlangen-Nuremberg from 1972-1977 involved analyzing cervical samples for HSV-2 DNA using nucleic acid hybridization. Crucially, he repeatedly failed to find any HSV-2 DNA in these samples.^1^ This significant negative finding led him to conclude that HSV-2 did not cause cervical cancer.^6^

In February 1976, driven by his findings and observations of patients whose genital warts progressed to cervical cancer, zur Hausen published "Condylomata Acuminata and Human Genital Cancer".^1^ In this seminal work, he formally stated his hypothesis that HPV caused cervical cancer, directly contrasting it with the prevailing HSV-2 theory.^1^ He proposed that if viruses induced cancer, the tumors would contain viral DNA, thus identifying the virus as an oncovirus.^6^ He specifically hypothesized that HPV-caused genital warts, condylomata acuminata, were implicated in inducing cervical cancer.^1^ Zur Hausen's work represents a fundamental shift in the scientific understanding of cervical cancer etiology. His conclusion that HSV-2 was not the cause, based on rigorous molecular evidence (or lack thereof), was a crucial turning point that redirected the focus of cervical cancer research. This was not merely proposing an alternative; it was a direct refutation of the dominant theory based on a specific and increasingly powerful type of evidence (molecular detection of viral DNA). This highlights the scientific process of challenging established beliefs with new data.

**Clinical and Cytological Manifestations**

HPV was already known to be the causative agent of genital warts (condylomata acuminata).^1^ Zur Hausen's hypothesis was partly predicated on clinical observations suggesting that patients with genital warts often went on to develop cervical cancer.^6^ This clinical link provided early, albeit indirect, support for HPV's involvement.

A significant cytological marker, koilocytotic atypia, was increasingly recognized as a distinct manifestation of papillomavirus-induced cytopathic change in cervical dysplasia.^1^ This condition, characterized by cytoplasmic vacuolization and peripheral cytoplasmic condensation, was formally defined in Acta Cytologica in 1981.^17^ Its association with cervical intraepithelial neoplasia (CIN) and viral infection was becoming well-established by 1981.^9^ Alexander Meisels and Roger Fortin described these characteristic "koilocytes" in 1976 ^20^, noting their morphological similarity to cells found in vulvar condylomata acuminata and explicitly suggesting a relationship of HPV with cancer of the cervix.^21^ The recognition and characterization of koilocytotic atypia provided a crucial morphological link between HPV infection and precancerous cervical lesions. This observable cellular change, directly associated with a known HPV-caused lesion (genital warts), provided compelling clinical and pathological evidence for HPV's involvement, even before widespread molecular detection of specific oncogenic HPV types in cancer was feasible. This represented a significant step in visually identifying HPV's impact on cervical tissue.

**Initial Molecular Investigations**

Zur Hausen's initial attempts in 1974 to find HPV DNA in cervical cancer and genital wart biopsies using nucleic acid hybridization with complementary RNA (cRNA) derived from plantar wart HPV DNA were consistently negative for genital lesions.^1^ This early difficulty in detecting HPV DNA in genital samples, despite electron microscopic evidence of HPV particles in plantar warts, led him to hypothesize genetic heterogeneity among HPV virus types.^1^ This indicated that the probes available at the time were not sufficient to capture the diversity of HPV types in genital lesions.

A pivotal molecular breakthrough occurred in 1981 with the successful molecular cloning of viral DNA from human genital warts, which led to the identification of the first genital HPV type, HPV6.^1^ DNA from HPV6 was subsequently repeatedly isolated from genital warts and condylomata acuminata.^1^ This was a crucial step because it provided a specific, identifiable HPV type associated with genital lesions, enabling more precise and targeted molecular research into the broader family of HPV types and their potential links to cervical cancer.^1^ The successful molecular cloning of HPV6 in 1981 was a methodological breakthrough that provided the essential tools to move beyond indirect associations and initial detection failures. While the high-risk HPV types (e.g., HPV16 and HPV18) directly linked to cervical cancer were not yet identified by 1981, the cloning of HPV6 demonstrated the feasibility of isolating and characterizing genital HPV types. This success significantly advanced the field, setting the stage for subsequent, more definitive discoveries and representing a clear trend towards molecular confirmation of HPV's role.

**Table 2: Summary of Evidence for HPV in Cervical Cancer (Pre-1982)**

| **Type of Evidence** | **Key Findings Supporting HPV Hypothesis (Pre-1982)** | **Limitations/Uncertainties by 1981** |
| --- | --- | --- |
| **Hypothesis/Conceptual** | - Harald zur Hausen's 1976 hypothesis proposing HPV as the cause.  - Zur Hausen's failure to detect HSV-2 DNA in cervical tumors, which prompted his HPV hypothesis. | - HPV hypothesis was newer and less established than HSV-2 hypothesis. |
| **Clinical/ Cytological** | - Association of HPV with genital warts (condylomata acuminata).  - Identification of koilocytotic atypia as an HPV-induced cytopathic effect in cervical lesions. | - Clinical associations were largely circumstantial for direct cancer causation. |
| **Molecular** | - Molecular cloning of HPV6 from genital warts in 1981. | - Lack of consistent, direct detection of HPV DNA in cervical cancer cells (initial attempts were negative for genital lesions).  - Specific high-risk HPV types (e.g., HPV16/18) not yet definitively identified in cervical cancer by 1981. |

**V. Comparative Analysis and Conclusion: The Balance of Evidence in 1981**

**Direct Comparison of Strengths and Weaknesses**

By 1981, the HSV-2 hypothesis still represented the prevailing view regarding the viral etiology of cervical cancer.^1^ This dominance was primarily attributed to the robust and consistent seroepidemiological associations, which repeatedly demonstrated a higher prevalence of HSV-2 antibodies in cervical cancer patients.^1^ Furthermore, the epidemiological patterns of HSV-2 infection aligned well with the established sexual risk factors for cervical cancer.^5^ The biological plausibility, including HSV's ability to infect cervical cells and its observed oncogenic potential in experimental models, provided additional support.^3^

However, the fundamental and increasingly problematic weakness of the HSV-2 hypothesis lay in the repeated failure to consistently detect viral DNA within cervical tumor cells.^1^ This molecular disconnect was a significant contradiction to the evolving understanding of viral oncogenesis, which posited the persistent presence of viral genetic information in transformed cells.^1^ This inconsistency raised profound questions about HSV-2's direct causal role, leading to an ongoing debate about whether it was a primary carcinogen or merely a cofactor.^3^ The characterization of HSV-2 as "not a typical DNA tumor virus" ^3^ further underscored these molecular challenges.

The HPV hypothesis, while more nascent, was rapidly gaining scientific traction due to compelling clinical and cytological observations. The well-established link between HPV and genital warts^1^ provided a clear association with sexually transmitted genital lesions. More importantly, the recognition and detailed description of koilocytotic atypia as a distinct, HPV-specific cytopathic effect in cervical dysplastic lesions^1^ offered a tangible, observable link between papillomavirus infection and precancerous changes in the cervix. Crucially, Harald zur Hausen's direct refutation of HSV-2 DNA presence in cervical tumors^1^ provided a strong impetus for pursuing alternative viral etiologies. The molecular cloning of HPV6 in 1981^1^ represented a significant methodological breakthrough, demonstrating the feasibility of isolating and characterizing genital HPV types, even if the specific high-risk types directly involved in cervical cancer were not yet identified. By 1981, the debate was characterized by a fundamental tension between strong epidemiological correlation (HSV-2) and emerging molecular and cytological evidence (HPV) that directly challenged the former. The lack of consistent molecular detection of HSV-2 DNA in tumor cells was a critical flaw that, despite its widespread acceptance, made its direct causal role increasingly questionable. This highlights the scientific method at play: new evidence (or the lack thereof) challenging established beliefs and driving a re-evaluation of hypotheses.

**Assessment of "More Likely" based on available data in 1981**

Based solely on the information available by the end of 1981, while HSV-2 still held a dominant position in the collective scientific consciousness due to its overwhelming epidemiological and serological support, the scientific ground was demonstrably shifting. The consistent failure to detect HSV-2 DNA in cervical tumor cells by advanced molecular techniques^1^ represented a profound and growing challenge to its direct causal role, suggesting that its association might be indirect or that it acted as a cofactor.

Conversely, the HPV hypothesis, though less established in terms of direct, consistent molecular proof of its presence within cervical cancer cells by 1981, was gaining significant credibility through compelling indirect evidence. This included the clear association of HPV with genital warts, the increasingly recognized and distinct cytological marker of koilocytotic atypia in cervical lesions^1^, and the groundbreaking molecular cloning of HPV6.^1^ This cloning demonstrated that genital HPV types could be isolated and characterized, providing the necessary tools for more definitive future investigations.

Therefore, considering the limitations and contradictions facing the HSV-2 hypothesis, coupled with the promising, albeit nascent, molecular and cytological evidence for HPV, it was becoming **more likely that Human Papillomavirus (HPV) was the primary cause of cervical cancer**. The decisive factor shifting the balance was the critical negative evidence against HSV-2's persistent presence in tumors, combined with the positive, albeit indirect, evidence for HPV's association with precancerous lesions and the significant methodological advancements in HPV research. The HSV-2 hypothesis was increasingly challenged by its inability to meet the evolving molecular criteria for viral oncogenesis, while HPV offered a more promising and scientifically robust path forward, even if the definitive molecular smoking gun (e.g., the identification of HPV16/18 in cervical cancer) was still on the horizon. The assessment of "more likely" is not simply about which hypothesis had more pieces of evidence, but rather which had more compelling and less contradictory evidence, particularly at the molecular level, which was gaining increasing prominence in viral oncology. The absence of HSV-2 DNA in tumors was a more fundamental negative indicator for its direct causal role than the absence of definitive HPV-cancer DNA by 1981, because HPV research was still in its very early stages of identifying relevant types and their presence in cancer. This represents a subtle but critical shift in scientific confidence driven by methodological advancements and the identification of a clear path for future research.

**Discussion of the Scientific Momentum Shifting Towards HPV, Despite the Lack of Definitive Molecular Proof of its Direct Causal Role in Cervical Cancer by this Specific Year**

The scientific community was increasingly aware of the limitations of the HSV-2 hypothesis, particularly regarding the elusive viral DNA in tumors. This created an intellectual vacuum and a strong impetus for exploring alternative etiologies, which HPV research was beginning to fill. The identification of koilocytes as a distinct morphological marker of HPV infection in cervical lesions^1^ provided a tangible, observable link between papillomavirus infection and cervical pathology, offering a clear target for further investigation. The success in molecularly cloning HPV6 in 1981^1^ was a critical technological advancement. It signaled that molecular techniques were advancing rapidly and would likely yield more definitive answers soon regarding the presence and role of specific HPV types in cervical cancer, even if the specific high-risk types (HPV16/18) were yet to be identified in cancer by this precise year.^1^ This breakthrough provided the necessary tools to directly test zur Hausen's hypothesis on a molecular level. The shift in scientific momentum is a crucial underlying theme. It reflects not just the accumulation of facts, but the collective scientific intuition and the perceived most fruitful direction for future research. The methodological advancements, particularly in molecular cloning, were key drivers of this shift, promising the ability to resolve the DNA detection paradox that plagued the HSV-2 hypothesis and to definitively identify the true viral culprit.

VI. References

1. Adam, E., Kaufman, R. H., Melnick, J. L., Levy, A. H., & Rawls, W. E. (1981). Herpes simplex virus type 2 and human cervical cancer: relationship between cellular and immune assays for the detection of previous infection. Journal of the National Cancer Institute, 66(1), 117-122. 14
2. Castellsagué, X., & Muñoz, N. (2003). Human papillomavirus and cervical cancer: an epidemiological perspective. Journal of Clinical Pathology, 56(12), 887-892. (Information pertaining to pre-1982 understanding, specifically HSV-2 seroepidemiology). 4
3. Embryo Project Encyclopedia. (n.d.). Harald zur Hausen's Experiments on Human Papillomavirus Causing Cervical Cancer (1976–1987). (Information pertaining to pre-1982 understanding and discoveries). 6
4. Glowm. (n.d.). Cervical Intraepithelial Neoplasia: History and Detection. (Information pertaining to pre-1982 understanding, specifically zur Hausen's link in 1977). 9
5. Hartveit, F., & Thoresen, S. (1981). Koilocytes in cervical smears from women with malignant melanoma. Acta Dermato-Venereologica, 61(4), 349-351. 18
6. Mammas, I. N., Sourvinos, G., & Spandidos, D. A. (2014). Human papillomavirus (HPV) infection in children and adolescents: Historical aspects. Molecular Medicine Reports, 10(6), 2735-2740. (Information pertaining to pre-1982 understanding and discoveries, specifically 1978 report). 8
7. Meisels, A. (1991). Condylomatous lesions of the cervix and vagina. 1. Cytologic patterns. Acta Cytologica, 20(6), 505-509. (Information pertaining to Meisels A, Fortin R 1976 paper). 21
8. Mungall, A. J., & Laimins, L. A. (2014). Herpes simplex virus type 2 (HSV-2) and cervical cancer: a review. Virology Journal, 11(1), 1-10. (Information pertaining to pre-1982 understanding, specifically HSV-2 as risk factor and cofactor debate). 3
9. National Cancer Institute, Division of Cancer Prevention. (n.d.). Harald zur Hausen, MD, D.Sc. HPV and Cervical Cancer. (Information pertaining to pre-1982 understanding and discoveries, specifically 1976 hypothesis). 2
10. National Center for Biotechnology Information. (n.d.). Koilocytotic Atypia. (Definition from Acta Cytol 1981;25:377-82). 17
11. Nobel Prize. (2008). Advanced Information on the Nobel Prize in Physiology or Medicine 2008. (Information pertaining to pre-1982 understanding and discoveries, specifically 1974 & 1976 work, koilocytotic atypia, HPV6 cloning in 1981). 1
12. Pilotti, S., Rilke, F., & Della Torre, G. (1981). Papilloma virus infections of the squamous cervix: a review. Acta Cytologica, 25(4), 393-401. (Information pertaining to koilocytotic atypia and HPV association). 22
13. Rawls, W. E. (1981). An analysis of the association between herpes simplex virus type 2 antibodies and cervical cancer. Gorgas Memorial Institute of Tropical and Preventive Medicine. 5
14. Rawls, W. E. (1978). The Genital Herpes-Cervical Cancer Hypothesis—10 Years Later. In Oncogenesis and Herpesviruses III (pp. 861-872). Karger Publishers. (Information pertaining to pre-1982 understanding and discoveries). 15
15. ResearchGate. (n.d.). Flat wart with pronounced koilocytosis Haematoxylin and eosin x 80. (Information pertaining to pre-1982 understanding, specifically Pilotti et al., 1981). 19
16. Seth, P., & Sharma, S. (2003). Herpes simplex virus type 2 (HSV-2) is not a cofactor to human papillomavirus in cancer of the uterine cervix. American Journal of Obstetrics and Gynecology, 188(1), 129-134. (Information pertaining to pre-1982 understanding, specifically HSV-2 as not a typical DNA tumor virus). 3
17. Syrjänen, K. J. (2018). Two Landmark Studies Published in 1976/1977 Paved the Way for the Recognition of Human Papillomavirus as the Major Cause of the Global Cancer Burden. Acta Cytologica, 61(4-5), 316-320. (Information pertaining to Meisels A, Fortin R 1976 paper). 20
18. Wikipedia. (n.d.). Harald zur Hausen. (Information pertaining to pre-1982 understanding and discoveries, specifically 1976 hypothesis). 10
19. Wikipedia. (n.d.). Oncovirus. (Historical information on viral oncogenesis before 1981). 12

### Comments on above:

First, the LLM says the more likely hypothesis is HPV, which at the time in 1981 was not the prevailing hypothesis as it was before the landmark paper and before HPV DNA was isolated from cervical cancer biopsies. Based on the references cited above, most are cited incorrectly or are incomplete, or do not exist or in some cases are beyond the cutoff date. Additionally, not all of the references shown here are found in the text. The references found in the text are: 1,3,5,6,9,12,14,15,17,20, and 21. Therefore 10 references are not actually cited. Furthermore, there are no references 20 and 21 in the reference list. Finally, reference 1 which is cited several times as a reference for HPV is actually a reference for HSV-2, and does not mention HPV.

1. Reference 1: Adam, E., Kaufman, R. H., Melnick, J. L., Levy, A. H., & Rawls, W. E. (1981). Herpes simplex virus type 2 and human cervical cancer: relationship between cellular and immune assays for the detection of previous infection. Journal of the National Cancer Institute, 66(1), 117-122. 14
   1. a real reference (based on title and journal) but:
   2. the authors are **incorrect**, should be: Smith, Jerry W.; Torres, Jose E.; Holmquist, Nelson D.
   3. Additionally, this reference is exclusively talking about HSV 1 & 2, no mention of HPV. The LLM, however, cites this reference for many of its HPV “facts or findings”.
2. Reference 2: Castellsagué, X., & Muñoz, N. (2003). Human papillomavirus and cervical cancer: an epidemiological perspective. Journal of Clinical Pathology, 56(12), 887-892.
   1. Date is 2003, not before 1981
   2. **Article does not exist**
3. Reference 3: Embryo Project Encyclopedia. (n.d.). Harald zur Hausen's Experiments on Human Papillomavirus Causing Cervical Cancer (1976–1987).
   1. Date range goes to 1987
   2. **Article does not exist**
4. Reference 4: Glowm. (n.d.). Cervical Intraepithelial Neoplasia: History and Detection.
   1. **Article does not exist**- also incomplete
5. Reference 5: Hartveit, F., & Thoresen, S. (1981). Koilocytes in cervical smears from women with malignant melanoma. Acta Dermato-Venereologica, 61(4), 349-351.
   1. **Article does not exist**
6. Reference 6: Mammas, I. N., Sourvinos, G., & Spandidos, D. A. (2014). Human papillomavirus (HPV) infection in children and adolescents: Historical aspects. Molecular Medicine Reports, 10(6), 2735-2740.
   1. **Article is from 2014**
   2. The **reference is wrong**, there is a paper by Mammas, I. N., Sourvinos, G., & Spandidos, D. A. in 2014 but it is entitled “The paediatric story of human papillomavirus” and was published in Oncology letters: <https://doi.org/10.3892/ol.2014.2226>
7. Reference 7: Meisels, A. (1991). Condylomatous lesions of the cervix and vagina. 1. Cytologic patterns. Acta Cytologica, 20(6), 505-509.
   1. **Article from 1991**
   2. there is an article with this same title and author in Acta cytologica, but it is from 1976: PMID: 1069445, so **reference is wrong**
8. Reference 8: Mungall, A. J., & Laimins, L. A. (2014). Herpes simplex virus type 2 (HSV-2) and cervical cancer: a review. Virology Journal, 11(1), 1-10.
   1. Article from 2014
   2. **Article does not exist**
9. Reference 9: National Cancer Institute, Division of Cancer Prevention. (n.d.). Harald zur Hausen, MD, D.Sc. HPV and Cervical Cancer.
   1. **Incomplete** (no date, title, journal)- looks just like the place where zur Hausen works or worked
10. Reference 10: National Center for Biotechnology Information. (n.d.). Koilocytotic Atypia. (Definition from Acta Cytol 1981;25:377-82).
    1. **Incomplete**- no authors
    2. Article Acta Cytol 1981;25:377-82 is entitled “Histopathologic features of koilocytotic atypia: a detailed description.”
11. Reference 11: Nobel Prize. (2008). Advanced Information on the Nobel Prize in Physiology or Medicine 2008.
    1. This is not a journal article - There is a website for the Nobel Prize: <https://www.nobelprize.org/prizes/medicine/2008/advanced-information/> or LLM could just be referring to its parametric data when zur Hausen won the noble prize, but either way that would mean it is taking evidence from 2008. So it is **beyond the cutoff date**.
12. Reference 12: Pilotti, S., Rilke, F., & Della Torre, G. (1981). Papilloma virus infections of the squamous cervix: a review. Acta Cytologica, 25(4), 393-401.
    1. **Article does not exist**
13. Reference 13: Rawls, W. E. (1981). An analysis of the association between herpes simplex virus type 2 antibodies and cervical cancer. Gorgas Memorial Institute of Tropical and Preventive Medicine
    1. **Article does not exist**
14. Reference 14: Rawls, W. E. (1978). The Genital Herpes-Cervical Cancer Hypothesis—10 Years Later. In Oncogenesis and Herpesviruses III (pp. 861-872). Karger Publishers.
    1. **Information is incorrect.** There is an article with this title that was published in 1978, but the authors are different: A J Nahmias, S Sawanabori, and the journal is Prog Exp Tumor Res., doi: 10.1159/000400861
15. Reference 15: ResearchGate. (n.d.). Flat wart with pronounced koilocytosis Haematoxylin and eosin x 80.
    1. This is from research gate, **which did not exist in or prior to 1981**- the paper that is referenced in this post: “Human papillomavirus infection of the uterine cervix: Histological appearances in 28 cases identified by immunohistochemical techniques” is **from 1984**
16. Reference 16: Seth, P., & Sharma, S. (2003). Herpes simplex virus type 2 (HSV-2) is not a cofactor to human papillomavirus in cancer of the uterine cervix. American Journal of Obstetrics and Gynecology, 188(1), 129-134
    1. This is **reference incorrect**- the authors are wrong, they should be: <https://doi.org/10.1067/mob.2003.66>
    2. Also it is from 2003
17. Reference 17: Syrjänen, K. J. (2018). Two Landmark Studies Published in 1976/1977 Paved the Way for the Recognition of Human Papillomavirus as the Major Cause of the Global Cancer Burden. Acta Cytologica, 61(4-5), 316-320
    1. This reference is correct but was **published in 2018- after the cutoff date.**
18. References 18 & 19 are both **wikipedia articles, so obviously not available prior to 1981.**

### Response from DeepSeek:

To evaluate the competing hypotheses regarding the causation of cervical cancer—Human Papillomavirus (HPV) vs. Herpes Simplex Virus (HSV)—using only information available up to 1981, we can rely on the scientific literature from that time period.

**Key Findings (Pre-1981):**

1. Herpes Simplex Virus (HSV) Hypothesis:

- In the 1960s and 1970s, HSV-2 was initially suspected as a potential cause of cervical cancer due to epidemiological associations. Women with cervical cancer often had higher HSV-2 antibody levels.

- References:

- Nahmias et al. (1970) reported seroepidemiological evidence linking HSV-2 to cervical cancer (American Journal of Epidemiology).

- Frenkel et al. (1972) found HSV-2 DNA fragments in cervical tumor cells, but this was not consistently reproducible (Proceedings of the National Academy of Sciences).

2. Human Papillomavirus (HPV) Hypothesis:

- By the late 1970s, HPV (particularly HPV-16 and HPV-18) emerged as a stronger candidate due to histological and early molecular evidence.

- References:

- Zur Hausen (1976, 1977) proposed HPV as a likely cause, noting similarities between warts and cervical lesions (Cancer Research).

- Meisels & Fortin (1976) identified koilocytes (HPV-associated cellular changes) in cervical smears (Acta Cytologica).

- Laverty et al. (1978) detected HPV particles in cervical cancer biopsies (Journal of Clinical Pathology).

**Conclusion (Pre-1981):**

By 1981, **HPV was considered the more likely cause** of cervical cancer than HSV, though the evidence was still emerging. HSV-2 research had methodological limitations (e.g., antibody studies could not prove causation), whereas HPV was consistently detected in precancerous lesions. Zur Hausen’s work was particularly influential in shifting focus toward HPV.

Key References:

1. Zur Hausen, H. (1977). Cancer Research, 37(3), 794-795. (HPV hypothesis)

2. Meisels, A., & Fortin, R. (1976). Acta Cytologica, 20(6), 505-509. (Koilocytes)

3. Nahmias, A. J., et al. (1970). Am J Epidemiol, 91(6), 547-552. (HSV-2 serology)

Would you like more details on any specific study?

### Comments about above:

1. Reference Nahmias, A. J., et al. (1970). Am J Epidemiol, 91(6), 547-552
   1. A **real reference, before 1981**
2. Frenkel et al. (1972) PNAS
   1. **Incomplete reference**
   2. When we asked a follow up to give the full reference, it did give **real and relevant reference** about HSV2 and a fragment of HSV2 DNA being found in cervical cancer tissue DOI: [10.1073/pnas.69.12.3784](https://doi.org/10.1073/pnas.69.12.3784)
3. Reference: zur Hausen, H. (1977). *Cancer Research*, 37(3), 794-795.
   1. **Incomplete, or not real reference**
   2. When we asked for the full reference, it gave me a different zur Hausen 1977 reference from Current Topics in Microbiology and Immunology: <https://doi.org/10.1007/978-3-642-66800-5_1>. This reference is **real and is relevant**
4. Reference: Laverty et al. (1978),
   1. **Incomplete reference**
   2. When asked to give the full reference it **made up a reference**, complete with a doi to an unrelated paper: Title: "The Detection of Human Papillomavirus DNA in Cervical Smears and Cancers by *In Situ* Hybridization" Authors: Laverty, C. R., Russell, P., Hills, E., & Booth, N. Journal: *Journal of Clinical Pathology* https://doi.org/10.1136/jcp.31.6.527
5. Meisels, A., & Fortin, R. (1976). Acta Cytologica, 20(6), 505-509.
   1. Is a **real and relevant reference before 1981**

### Response from Perplexity

Prior to 1981, the prevailing evidence suggested Herpes Simplex Virus (HSV) was more strongly suspected as a cause of cervical cancer, though emerging research began implicating Human Papillomavirus (HPV). Here’s the breakdown of pre-1981 evidence:

**Herpes Simplex Virus (HSV) Hypothesis**

- **Epidemiological association**: Multiple studies linked HSV-2 to cervical cancer through sexual behavior patterns (early sexual activity, multiple partners)[2](https://pmc.ncbi.nlm.nih.gov/articles/PMC172875/)[4](https://pmc.ncbi.nlm.nih.gov/articles/PMC1769629/).
- **Biological plausibility**: HSV-2 was known to cause cellular changes and unscheduled DNA synthesis, though its role in cancer remained unclear due to its cell-killing nature[1](https://pmc.ncbi.nlm.nih.gov/articles/PMC7089001/)[2](https://pmc.ncbi.nlm.nih.gov/articles/PMC172875/).
- **Serological evidence**: Higher HSV-2 antibody levels were found in cervical cancer patients compared to controls[1](https://pmc.ncbi.nlm.nih.gov/articles/PMC7089001/)[2](https://pmc.ncbi.nlm.nih.gov/articles/PMC172875/).

**Human Papillomavirus (HPV) Hypothesis**

- **Emerging animal model evidence**: By the 1970s, papillomaviruses were recognized to cause animal cancers and genital warts, leading Harald zur Hausen to hypothesize HPV’s role in cervical cancer in 1976[1](https://pmc.ncbi.nlm.nih.gov/articles/PMC7089001/)[3](https://prevention.cancer.gov/about-dcp/history-and-timeline/commemoration-50th/harald-zur-hausen).
- **Early virological challenges**: Initial attempts to detect HSV-2 in cervical tumors failed, shifting focus to HPV[3](https://prevention.cancer.gov/about-dcp/history-and-timeline/commemoration-50th/harald-zur-hausen).
- **Indirect clinical observations**: Association of genital warts (HPV-induced) with cervical cancer progression supported HPV’s potential role[3](https://prevention.cancer.gov/about-dcp/history-and-timeline/commemoration-50th/harald-zur-hausen).

**Pre-1981 Consensus**

While **HSV-2 had stronger epidemiological support** by 1981, **HPV gained traction due to**:

1. zur Hausen’s inability to confirm HSV-2 in tumors[3](https://prevention.cancer.gov/about-dcp/history-and-timeline/commemoration-50th/harald-zur-hausen)
2. Papillomaviruses’ known oncogenic potential in animals[1](https://pmc.ncbi.nlm.nih.gov/articles/PMC7089001/)
3. Recognition that HPV’s persistence (unlike HSV’s lytic cycle) better matched cancer biology[1](https://pmc.ncbi.nlm.nih.gov/articles/PMC7089001/)[4](https://pmc.ncbi.nlm.nih.gov/articles/PMC1769629/).

**Key pre-1981 references**:

- zur Hausen’s 1976 HPV hypothesis[3](https://prevention.cancer.gov/about-dcp/history-and-timeline/commemoration-50th/harald-zur-hausen)
- Serological HSV-2 studies from the 1970s[1](https://pmc.ncbi.nlm.nih.gov/articles/PMC7089001/)[2](https://pmc.ncbi.nlm.nih.gov/articles/PMC172875/)
- Papillomavirus animal cancer research predating 1981[1](https://pmc.ncbi.nlm.nih.gov/articles/PMC7089001/)[4](https://pmc.ncbi.nlm.nih.gov/articles/PMC1769629/).

Conclusive HPV evidence (e.g., HPV-16/18 discovery) emerged post-1981[1](https://pmc.ncbi.nlm.nih.gov/articles/PMC7089001/)[3](https://prevention.cancer.gov/about-dcp/history-and-timeline/commemoration-50th/harald-zur-hausen)[4](https://pmc.ncbi.nlm.nih.gov/articles/PMC1769629/).

1. <https://pmc.ncbi.nlm.nih.gov/articles/PMC7089001/>
2. <https://pmc.ncbi.nlm.nih.gov/articles/PMC172875/>
3. <https://prevention.cancer.gov/about-dcp/history-and-timeline/commemoration-50th/harald-zur-hausen>
4. <https://pmc.ncbi.nlm.nih.gov/articles/PMC1769629/>
5. <https://www.sciencedirect.com/science/article/pii/S0042682208007721>
6. <https://pubmed.ncbi.nlm.nih.gov/6941038/>
7. <https://medicine.yale.edu/news/yale-medicine-magazine/article/the-virus-behind-the-cancer/>
8. <https://www.cdc.gov/pinkbook/hcp/table-of-contents/chapter-11-human-papillomavirus.html>
9. <https://obgyn.onlinelibrary.wiley.com/doi/10.1111/jog.15997>
10. <https://www.cdc.gov/mmwr/volumes/68/wr/mm6833a3.htm>
11. <https://www.aacr.org/blog/2021/09/28/the-past-present-and-future-of-hpv-can-vaccination-help-eliminate-cervical-cancer/>
12. <https://www.nature.com/articles/nrc798>
13. <https://pmc.ncbi.nlm.nih.gov/articles/PMC10378257/>
14. <https://pmc.ncbi.nlm.nih.gov/articles/PMC145302/>

### Comments on above:

All of the references used here are **beyond the cutoff date** **except for one**. Additionally, they tend to be webpages, wikipedia articles, or blog posts, not actual peer-reviewed articles.

1. Reference 1 is an editorial review from 2019
2. Reference 2 is from 1995
3. Reference 3 is a webpage from the National Cancer Institute about zur Hausen (so obviously a reference post 1981)
4. Reference 4 is from 2002
5. Reference 5 is a historical account written by zur Hausen in 2009
6. Reference 6 is from 1981 and is **relevant** about associating HSV with cervical cancer and is within the **correct time range**.
7. Reference 7 is a post from Yale medicine magazine in 2006
8. Reference 8 is a chapter in a book published by the CDC in 2024
9. Reference 9 is also from 2024 and is more about diagnosing, treating, and preventing cervical cancer and about the HPV vaccine
10. Reference10 is a report from the CDC in 2019
11. Reference 11 is a post from the American Association for Cancer research from 2021
12. Reference 12 is a 2002 review
13. Reference 13 is a 2023 review
14. Reference 14 is a 2003 review

See **Supplementary Table 1** for summary.

### Response from Chat-GPT 5

Excellent historical question — here’s a careful evaluation based **only on scientific knowledge and publications available up to 1981** about the two competing hypotheses for the cause of **cervical cancer**:

**Hypothesis 1:** Cervical cancer is caused by *Human Papillomavirus* (HPV).
**Hypothesis 2:** Cervical cancer is caused by *Herpes Simplex Virus* (HSV), particularly HSV-2.

**1. Background (as of 1981)**

By 1981, the etiology of cervical cancer was still uncertain. Both *Herpes Simplex Virus type 2 (HSV-2)* and *Human Papillomavirus (HPV)* were being actively studied as possible sexually transmitted carcinogens. The prevailing consensus among most cancer epidemiologists in the 1970s leaned toward **HSV-2** as the likely agent, but **HPV** was emerging as an intriguing and mechanistically plausible alternative.

**2. Evidence for Herpes Simplex Virus (HSV)**

a. Epidemiological association

- Several case–control studies in the 1970s reported higher HSV-2 antibody prevalence in women with cervical cancer:
  - **Rawls et al. (1970)**, *American Journal of Epidemiology*, 91(6): 531–540 — found a correlation between HSV-2 antibody titers and cervical carcinoma.
  - **Nahmias et al. (1972)**, *Science*, 178: 160–161 — reported that women with cervical cancer were more likely to have antibodies to HSV-2.
  - **Frenkel et al. (1972)**, *Proceedings of the National Academy of Sciences USA*, 69(9): 2641–2645 — detected HSV-like antigens in cervical tumor tissue.

These findings supported the idea that HSV-2, a sexually transmitted virus, could act as a cofactor or initiator in cervical carcinogenesis.

b. Biological plausibility

- HSV-2 was known to establish **latent infections** in genital tissues, and herpesviruses were already linked to other cancers (e.g., Epstein–Barr virus and Burkitt’s lymphoma).
- Some studies reported that HSV DNA or antigens could be found in cervical tumor cells (e.g., **Rapp et al., 1973**, *Nature New Biology*, 241: 157–159), suggesting possible integration or persistence.

c. Weaknesses of HSV hypothesis (known by 1981)

- Results were inconsistent — not all studies detected HSV DNA in tumors.
- HSV could cause cytopathic effects but not stable transformation in human cells.
- By the late 1970s, some researchers began questioning whether HSV-2 was merely a **marker of sexual activity** rather than a causal agent (e.g., **Kessler, 1976**, *Cancer*, 38: 2004–2008).

**3. Evidence for Human Papillomavirus (HPV)**

a. Early clinical and histological observations

- **Zur Hausen and Schneider (1970s)** had reported that papillomaviruses cause genital warts (condylomata acuminata) and that these lesions sometimes showed **dysplastic or precancerous changes** (Zur Hausen et al., *International Journal of Cancer*, 1974, 13: 657–664).
- HPV was already known to cause **skin papillomas** and cancers in animals (e.g., **Rous and Beard, 1935**, *Journal of Experimental Medicine*, 62: 523–548), supporting biological plausibility for a viral oncogene mechanism.

b. Detection of HPV in cervical lesions

- By **1976**, **Harald zur Hausen** proposed that certain types of HPV, distinct from those causing warts, might be involved in cervical cancer (Zur Hausen, *Bacteriological Reviews*, 1976, 40(1): 1–26).
- **1977–1979:** Attempts to detect HPV DNA in cervical carcinomas were beginning to succeed:
  - **Gissmann & zur Hausen (1976)**, *International Journal of Cancer*, 17: 418–427 — identified HPV DNA in genital warts.
  - **Gissmann & zur Hausen (1980)**, *Proceedings of the National Academy of Sciences USA*, 77(12): 8137–8141 — cloned HPV-6 DNA from genital warts (still benign lesions, but crucial methodological groundwork).
  - **Syrjänen et al. (1980)**, *Archives of Gynecology*, 231: 169–182 — described morphological evidence of HPV infection (koilocytosis) in cervical precancerous lesions.

c. Weaknesses of HPV hypothesis (by 1981)

- Direct detection of HPV in invasive cervical carcinoma was **not yet achieved** (HPV-16 and -18 were not identified until 1983–84).
- The link was largely circumstantial — based on histology and analogy to animal papillomaviruses.

**4. Comparative Evaluation (as of 1981)**

| **Criterion** | **HSV Hypothesis** | **HPV Hypothesis** |
| --- | --- | --- |
| **Epidemiological evidence** | Stronger (many serological studies showing correlation) | Weak or indirect (no clear epidemiologic data yet) |
| **Detection in tumors** | Inconsistent detection of HSV DNA or antigens | HPV DNA not yet conclusively detected in cancers |
| **Biological plausibility** | Plausible, based on other herpesvirus oncogenicity | Plausible, based on papillomavirus oncogenicity in animals |
| **Mechanistic understanding** | Weak: HSV-2 did not stably transform human cells | Growing: animal papillomaviruses known to integrate and transform |
| **Leading hypothesis in 1981** | HSV-2 widely considered the more likely cause | HPV emerging as an alternative but minority view |

**5. Conclusion (as of 1981)**

Based on data available up to 1981, **the Herpes Simplex Virus type 2 (HSV-2) hypothesis was more widely supported and considered more likely** by the scientific community.
 However, a few visionary researchers (especially **Harald zur Hausen**) were already arguing that **Human Papillomaviruses** might be the true cause — a hypothesis that would be decisively confirmed only later (HPV-16/18 discovery in 1983–84, followed by mechanistic studies in the 1980s–1990s).

**6. Key References (pre-1981)**

- Rawls, W. E., Tompkins, W. A. F., Figueroa, M. E., & Nahmias, A. J. (1970). *Association of Herpesvirus type 2 and carcinoma of the cervix.* **Am J Epidemiol**, 91(6): 531–540.
- Nahmias, A. J. et al. (1972). *Antibodies to Herpesvirus hominis types 1 and 2 in humans.* **Science**, 178: 160–161.
- Rapp, F., Buss, J., & Post, J. E. (1973). *Herpesvirus type 2: association with cervical carcinoma and possible role in oncogenesis.* **Nature New Biology**, 241: 157–159.
- Kessler, I. I. (1976). *Epidemiology of cervical cancer: critical review.* **Cancer**, 38(4): 2004–2008.
- Zur Hausen, H. (1976). *Condylomata acuminata and human genital cancer.* **Bacteriological Reviews**, 40(1): 1–26.
- Gissmann, L., & Zur Hausen, H. (1976). *Human papilloma virus DNA in genital and laryngeal papillomas.* **Int J Cancer**, 17: 418–427.
- Syrjänen, K. et al. (1980). *Morphological evidence of HPV infection in cervical intraepithelial neoplasia.* **Arch Gynecol**, 231: 169–182.

**In summary:**

As of 1981, the *Herpes Simplex Virus type 2* hypothesis was better supported empirically, though later evidence would overturn this view in favor of *Human Papillomavirus*.

### Comments on above:

The LLM correctly predicts that in 1981 HSV-2 was thought to be the more likely cause of cervical cancer. However in this case, all of the references are wrong.

**References:**

1. The first reference, Rawls, W. E., Tompkins, W. A. F., Figueroa, M. E., & Nahmias, A. J. (1970), **is incorrect.** There is a 1968 paper by Rawls, W. E., Tompkins, W. A. F., Figueroa, M. E., & Melnick in Science entitled “Herpesvirus Type 2: Association with Carcinoma of the Cervix”, but the authors, title, date, journal are all different from reference 1.
2. For the Nahmias 1972 reference, this reference **is incorrect.** There are two references from 1970: ‘Nahmias AJ, Josey WE, Naib ZM, Luce CF, Duffey A. Antibodies to Herpesvirus hominis types 1 and 2 in humans. I. Patients with genital herpetic infections. Am J Epidemiol. 1970 Jun;91(6):539-46. doi: 10.1093/oxfordjournals.aje.a121165.’ ; ‘Nahmias AJ, Josey WE, Naib ZM, Luce CF, Guest BA. Antibodies to Herpesvirus hominis types 1 and 2 in humans. II. Women with cervical cancer. Am J Epidemiol. 1970 Jun;91(6):547-52. doi: 10.1093/oxfordjournals.aje.a121166. ‘; but these are in a different journal for a different year.
3. The third reference, Rapp, F., Buss, J., & Post, J. E. (1973). *Herpesvirus type 2: association with cervical carcinoma and possible role in oncogenesis.* **Nature New Biology**, 241: 157–159. **Does not exist.**
4. The Kessler, I.I. reference from 1976 **is incorrect/ non-existent**. There is a paper by Kessler in 1976, but the title and journal information are all wrong. Here is the paper: Kessler II. Human cervical cancer as a venereal disease. Cancer Res. 1976 Feb;36(2 pt 2):783-91. PMID: 175940.
5. For the zur Hausen 1976 reference, there is an article by zur Hausen with this title in 1976, but the journal is wrong, thus the citation **is incorrect**. It should be: Harald zur Hausen; Condylomata Acuminata and Human Genital Cancer. *Cancer Res* 1 February 1976; 36 (2_Part_2): 794.
6. The Gissmann, L., & Zur Hausen, H. (1976) reference **does not exist**. There is a paper from 1983 with Gissmann, L., & Zur Hausen, H, but there are also other authors, a different title, and journal. This is the paper: Gissmann L, Wolnik L, Ikenberg H, Koldovsky U, Schnürch HG, zur Hausen H. Human papillomavirus types 6 and 11 DNA sequences in genital and laryngeal papillomas and in some cervical cancers. Proc Natl Acad Sci U S A. 1983 Jan;80(2):560-3. doi: 10.1073/pnas.80.2.560.
7. Finally the Syrjänen, K. et al. (1980) reference **does not exist**. There is a paper by Syrjänen, K. et al. in 1983, but the title, journal, and date of publication are different, and it is about oral cancer.

## Question - response to LLMs about scrapie being caused by protein or virus prior to 1980

### Question:

Please evaluate the following competing hypotheses using only information from 1980 or before. Please provide references showing where you got this information. Here are the hypotheses: "Is it more likely that scrapie is caused by a viral infection or by a protein infection?"

### Response from ChatGPT:

As of 1980, the scientific community had not reached a consensus on whether scrapie, a fatal neurodegenerative disease affecting sheep and goats, was caused by a viral infection or by a protein-based agent. Two primary hypotheses were under consideration:[PMC+1Wikipedia+1](https://pmc.ncbi.nlm.nih.gov/articles/PMC6760854/?utm_source=chatgpt.com)

**1. Viral Hypothesis**

The prevailing theory for much of the 20th century posited that scrapie was caused by an unconventional virus, often referred to as a "slow virus" due to the disease's long incubation period. This hypothesis was supported by observations of strain variations in transmissible spongiform encephalopathies (TSEs), which suggested the presence of an agent carrying genetic information. Additionally, the agent's resistance to standard viral inactivation methods led to the proposal of the "virino" hypothesis. This concept described the agent as a small nucleic acid molecule protected by a host-derived protein coat, explaining both its resilience and the lack of an immune response .[PMC+1Wikipedia+1](https://pmc.ncbi.nlm.nih.gov/articles/PMC6760854/?utm_source=chatgpt.com)[Wikipedia+3FAOHome+3The New Yorker+3](https://www.fao.org/4/W8656E/W8656E00.htm?utm_source=chatgpt.com)[Wikipedia+3FAOHome+3Wikipedia+3](https://www.fao.org/4/T0573E/T0573E06.htm?utm_source=chatgpt.com)

**2. Protein-Only (Prion) Hypothesis**

An alternative hypothesis emerged in the 1960s, challenging the central dogma of molecular biology, which held that nucleic acids were necessary for replication. Researchers like Tikvah Alper demonstrated that the scrapie agent was remarkably resistant to treatments that destroy nucleic acids, such as ultraviolet and ionizing radiation, suggesting the agent might lack nucleic acids altogether . In 1967, J.S. Griffith proposed that a misfolded protein could act as an infectious agent by inducing normal proteins to adopt the misfolded conformation. Building on this idea, Stanley Prusiner coined the term "prion" in 1982 to describe this proteinaceous infectious particle .[Wikipedia](https://en.wikipedia.org/wiki/Virino?utm_source=chatgpt.com)[ADS+5PMC+5PMC+5](https://pmc.ncbi.nlm.nih.gov/articles/PMC6760854/?utm_source=chatgpt.com)[PMC+1PMC+1](https://pmc.ncbi.nlm.nih.gov/articles/PMC3056934/?utm_source=chatgpt.com)[PNAS+9PMC+9The New Yorker+9](https://pmc.ncbi.nlm.nih.gov/articles/PMC4626585/?utm_source=chatgpt.com)

**Conclusion**

By 1980, both hypotheses had garnered support, but definitive evidence was lacking. The viral hypothesis was grounded in traditional understandings of infectious agents and was bolstered by observations of strain variability. Conversely, the protein-only hypothesis presented a radical departure from established biological principles, supported by experimental data indicating the agent's resistance to nucleic acid-targeting treatments. The scientific community remained divided, with ongoing research aimed at elucidating the true nature of the scrapie agent.[The New Yorker+1Wikipedia+1](https://www.newyorker.com/magazine/1997/12/01/pathological-science?utm_source=chatgpt.com)[FAOHome+1FAOHome+1](https://www.fao.org/4/T0573E/T0573E06.htm?utm_source=chatgpt.com)[PMC](https://pmc.ncbi.nlm.nih.gov/articles/PMC6760854/?utm_source=chatgpt.com)[FAOHome+6PMC+6PMC+6](https://pmc.ncbi.nlm.nih.gov/articles/PMC4626585/?utm_source=chatgpt.com)

### Comments on above:

The LLM says that the support for each hypothesis was divided in 1980, which is probably mostly true (as there were indications that scrapie was not a DNA virus). However, all of the references here are substantially beyond the cutoff date of 1980.

1. Reference 1: <https://doi.org/10.1042/bse0560181> is 2019 review about the protein only hypothesis and gives a history about the scrapie disease.
2. Reference 2: <https://www.fao.org/4/W8656E/W8656E00.htm>? MANUAL ON BOVINE SPONGIFORM ENCEPHALOPATHY is an animal health manual from 1998, which talks briefly about scrapie but also other transmissable spongiforms..
3. Reference 3: <https://www.fao.org/4/T0573E/T0573E06.htm>? Bovine spongiform encephalopathy is a book about scrapie and related diseases from 1993.
4. Reference 4: <https://en.wikipedia.org/wiki/Virino>? Is a Wikipedia article about virinos. This was thought to be the cause of scrapie: DNA covered with a protective coat of proteins, but never widely accepted.
5. Reference 5: <https://doi.org/10.1016/j.tibs.2010.11.001> is a review paper published in 2010 about prions.
6. Reference 6: <https://doi.org/10.1093/femspd/ftv087> is a review paper published in 2015 about the history of prions.
7. Reference 7: <https://www.newyorker.com/magazine/1997/12/01/pathological-science>? Is a New Yorker article from 1997 that talks about Stanley Prusiner winning the Nobel Prize for discovery of prions.

### Response from Gemini:

Based on information available in 1980 or before, the competing hypotheses for the cause of scrapie were actively debated, with evidence supporting both viral and proteinaceous agents. However, at that time, **neither hypothesis had definitively proven its case**, and the nature of the scrapie agent remained largely unknown.

Here's an evaluation of the evidence available for each hypothesis before or in 1980:

**Evidence for a Viral Infection (Pre-1980)**

The "slow virus" hypothesis was a prominent explanation for scrapie due to its long incubation period.

- **Transmissibility:** Scrapie was known to be transmissible, suggesting an infectious agent. Early in the 20th century, Cuille and Chelle hypothesized it was caused by a "slow virus" as early as 1938. Inoculating brain and cerebrospinal fluid from scrapie-infected sheep into healthy ones successfully transmitted the disease. (Cuille and Chelle, 1938, as cited in "A brief history of prions," PMC)
- **Filterable Agent:** The infectious agent could pass through filters that would retain bacteria, suggesting a very small size, consistent with a virus.
- **Long Incubation Periods:** The disease's characteristic long incubation periods (months to years) were similar to those observed with certain "slow viruses" identified in other diseases. Sigurðsson (1954) specifically suggested a slow virus due to this characteristic. ("A brief history of prions," PMC)
- **Genetic Influence on Incubation Period:** A. G. Dickinson identified a gene that seemed to control scrapie incubation periods in some mouse strains (Dickinson, Meikle, and Fraser 1968), which could be interpreted as host genetic factors influencing viral replication or pathology. ("A brief history of prions," PMC)

**Evidence for a Protein Infection (Pre-1980)**

While the concept of an infectious protein contradicted the central dogma of molecular biology, some early experimental observations pointed towards it.

- **Resistance to Inactivation Methods:** Crucial experiments demonstrated that the scrapie agent was remarkably resistant to treatments that typically inactivate nucleic acids (DNA and RNA), such as:
  - **Ionizing radiation:** Tikvah Alper and colleagues, in experiments starting around 1966 and published in 1967, found that the scrapie agent exhibited unusual resistance to ionizing radiation, suggesting a very small or absent nucleic acid component. They questioned, "Does the agent of scrapie replicate without nucleic acid?" (Alper, Cramp, Haig, & Clarke, 1967).
  - **Ultraviolet (UV) radiation:** Similar resistance was observed with UV radiation. (Alper et al., 1967)
  - **Heat, Formalin, and other chemicals:** The agent also showed resistance to extreme heat, formalin, and various chemicals known to deactivate viruses and bacteria. (Pattison, 1965; "A brief history of prions," PMC)
- **Proteinaceous Nature Inferred:** Based on these inactivation studies, some "prescient scientists" like Tikvah Alper, I.H. Pattison, and J.S. Griffith began to speculate that the scrapie agent could be of protein origin. (Pattison, 1965; "A brief history of prions," PMC)
- **Griffith's Hypothesis:** J.S. Griffith, in 1967, boldly proposed that the scrapie agent was proteinaceous and offered theoretical mechanisms for how a protein could be infectious and self-replicate without nucleic acid. (Griffith, 1967).
- **Further suggestive data:** Several researchers continued to accumulate data that suggested the scrapie agent's dependence on protein, including work by Hunter et al. (1969) and early work by Prusiner et al. (1978, 1980). ("A brief history of prions," PMC)

**Conclusion in 1980**

In 1980, the scientific community was still grappling with the unusual properties of the scrapie agent. While the "slow virus" hypothesis had historical precedent and some supporting evidence (transmissibility, filterability, long incubation), the increasing body of evidence demonstrating the agent's extraordinary resistance to nucleic acid-inactivating procedures strongly challenged the conventional understanding of viruses.

The "protein infection" hypothesis, though revolutionary and seemingly contradictory to the central dogma of molecular biology, gained traction because it could better explain these unusual inactivation profiles. However, a definitive *protein* had not yet been identified or purified as the sole infectious agent by 1980. The term "prion" (proteinaceous infectious particle) itself was coined by Stanley Prusiner in *1982*, reflecting the culmination of this pre-1980 research and his subsequent work.

Therefore, in 1980, it was **more likely that the scientific community was leaning towards the idea of a highly unconventional agent with a significant protein component and a very small or absent nucleic acid**, heavily questioning the traditional viral paradigm, rather than definitively stating it was a protein infection. The evidence for a protein infection was stronger in terms of explaining the agent's *resistance* to inactivation, while the evidence for a viral infection mostly relied on its *transmissibility* and disease characteristics (like incubation period). The exact nature of the agent remained an enigma, but the protein-only concept was certainly gaining ground as a viable, albeit radical, alternative to the prevailing viral theories.

**References:**

1. Alper, T., Cramp, W. A., Haig, D. A., & Clarke, M. C. (1967). Does the agent of scrapie replicate without nucleic acid? *Nature*, *214*(5090), 764-766.
2. "A brief history of prions." (n.d.). *PubMed Central (PMC)*. Retrieved from<https://pmc.ncbi.nlm.nih.gov/articles/PMC4626585/> (This source discusses pre-1980 research, including Cuille and Chelle (1938), Sigurðsson (1954), Dickinson, Meikle, and Fraser (1968), and early work by Alper, Pattison, Griffith, Hunter, and Prusiner).
3. Griffith, J. S. (1967). Self-replication and scrapie. *Nature*, *215*(5105), 1043-1044.
4. Pattison, I. H. (1965). Experiments with scrapie in the mouse: some difficulties in the interpretation of results. In *Slow, latent and temperate virus infections (NINDB Monograph No. 2)* (pp. 247-257). U.S. Government Printing Office. (This reference highlights challenges in interpreting results, which contributed to the unusual nature of the agent being recognized).

### Comments on above:

Here the LLM says that it was controversial at the time, which was true as the virus theory would mean it was a very unconventional virus, while the protein theory went against what everyone believed as the central dogma. Here two out of the four references are legitimate, while the others either don’t exist or are beyond the cut-off date of 1980.

1. Reference 1 (Alper, et al., 1967) is a **real and relevant reference.**
2. Reference 2 (PMC462585) was **published in 2015, and thus beyond the cut-off date.**
3. Reference 3 (Griffith, J. S. (1967)) is a **real and relevant reference.**
4. Reference 4 Pattison, I. H. (1965) **does not exist.**

### Response from DeepSeek

To evaluate the hypotheses regarding the cause of scrapie using only information available before or during 1980, we need to examine the scientific understanding of scrapie at that time. Here's the analysis based on historical references:

**1. Viral Hypothesis**

Before 1980, the dominant theory was that scrapie (and similar diseases like kuru and Creutzfeldt-Jakob disease) was caused by a "slow virus"—a hypothetical infectious agent with a long incubation period.

- Key References:
  - Sigurdsson (1954) proposed the concept of "slow infections," suggesting that scrapie might be caused by an unconventional virus (British Veterinary Journal).
  - Gajdusek (1976, Nobel Prize in Medicine) argued that transmissible spongiform encephalopathies (TSEs) like scrapie were caused by a virus-like agent (Science, 1977).
  - Marsh & Hanson (1969) demonstrated experimental transmission of scrapie in animals, supporting an infectious origin (Journal of Infectious Diseases).

**2. Protein Hypothesis (Prion Theory)**

By the late 1970s, an alternative hypothesis emerged, suggesting that scrapie might be caused by an infectious protein rather than a virus.

- Key References:
  - Alper et al. (1967) found that scrapie infectivity resisted treatments that typically inactivate nucleic acids (Nature), hinting at a non-viral agent.
  - Griffith (1967) proposed three theoretical mechanisms by which a protein could replicate without nucleic acids (Nature).
  - Prusiner (1982, but based on pre-1980 work) later coined the term "prion," but early evidence (late 1970s) suggested a proteinaceous infectious particle (PNAS).

**Conclusion (Pre-1980 Evidence)**

Before 1980, the viral hypothesis was more widely accepted, but experimental results (e.g., resistance to UV radiation, lack of detectable nucleic acid) were raising doubts. The protein hypothesis was gaining traction but was not yet dominant.

Final Assessment (Pre-1980):

- More likely before 1980: Viral infection (due to prevailing slow virus theory).
- Emerging alternative: Protein infection (supported by unconventional agent research).

Would you like more details on any specific study?

### Comments on above

The LLM correctly determines that in pre-1980, the hypothesis that scrapie is caused by a viral infection was more likely. However there are some references that are incorrect or beyond the cut-of date.

Assessing the references:

1. **Sigurdsson (1954):** After asking for a full reference, the LLM did give me a **viable reference** that describes “Rida” (ataxia or tremor) as a slow virus of sheep: Sigurdsson, B. (1954)."Rida, a chronic encephalitis of sheep: With general remarks on infections which develop slowly and some of their special characteristics." British Veterinary Journal, 110(8), 341-354. Rida is a synonym of scrapie
2. After asking for the full reference, we get this information which is a **legitimate reference** about encephalopathies caused by “unconventional viruses”: The second reference: **Gajdusek, D. C. (1977).** "Unconventional viruses and the origin and disappearance of kuru." *Science, 197*(4307), 943-960.
3. **Marsh & Hanson (1969)** <https://doi.org/10.1128/jvi.3.2.176-180.1969> was actually from the Journal of Virology and is about the transmissibility of mink encephalopathy and its biochemical properties that are similar to that of scrapie agent. Thus this is relevant but the **reference was incorrect.**
4. **Alper et al. (1967)** <https://doi.org/10.1038/214764a0> **is correct** and shows that scrapie does not need a nucleic acid to replicate.
5. **Griffith, J. S. (1967).** "Self-replication and scrapie." *Nature, 215*(5105), 1043-1044. [DOI: [10.1038/2151043a0](https://doi.org/10.1038/2151043a0)]. This is a **legitimate reference** discussing how scrapie might be caused by a self-replicating protein
6. For reference 6, this is the landmark paper showing scrapie to be caused by a protein, published after 1980 (Note is from DeepSeek). **So it is beyond the cutoff date**. **Prusiner, S. B. (1982).**"Novel proteinaceous infectious particles cause scrapie."
   *Science, 216*(4542), 136-144.
   [DOI: [10.1126/science.6801762](https://doi.org/10.1126/science.6801762)]
   (Note: Published slightly after 1980 but built on pre-1980 evidence.)

### Response from Perplexity

**Viral Hypothesis Challenges**

1. Filterable Agent but Non-Viral Properties
   Early experiments (1936) demonstrated transmission using filtered brain tissue, initially suggesting a virus[1](https://pmc.ncbi.nlm.nih.gov/articles/PMC1114482/)[3](https://www.cabidigitallibrary.org/doi/10.1079/cabicompendium.64847). However, subsequent studies revealed the agent’s extraordinary resistance to UV radiation, heat, and formaldehyde-treatments that typically inactivate viruses[3](https://www.cabidigitallibrary.org/doi/10.1079/cabicompendium.64847)[4](https://www.nature.com/articles/39120)[5](https://en.wikipedia.org/wiki/Scrapie). For example:
   - UV resistance (Alper et al., 1966): Scrapie infectivity persisted after UV exposure, implying the absence of nucleic acid, a critical viral component[2](https://pmc.ncbi.nlm.nih.gov/articles/PMC4626585/)[4](https://www.nature.com/articles/39120)[5](https://en.wikipedia.org/wiki/Scrapie).
   - Heat/formaldehyde resistance (Pattison, 1965): The agent survived autoclaving and formaldehyde treatment, inconsistent with viral structure[3](https://www.cabidigitallibrary.org/doi/10.1079/cabicompendium.64847).
2. Genetic Determinants
   Dickinson et al. identified host genes influencing scrapie susceptibility and strain variation in the 1960s, suggesting the agent interacted directly with host proteins rather than relying on viral replication mechanisms[1](https://pmc.ncbi.nlm.nih.gov/articles/PMC1114482/).

**Emerging Protein Hypothesis**

1. Protein-Only Theory
   J.S. Griffith proposed in 1967 that scrapie could arise from a self-replicating protein conformation[2](https://pmc.ncbi.nlm.nih.gov/articles/PMC4626585/)[4](https://www.nature.com/articles/39120). This aligned with:
   - No detectable immune response: Unlike viruses, the agent evaded immune detection[5](https://en.wikipedia.org/wiki/Scrapie).
   - Host-encoded prion protein: By 1985, Prusiner’s team later confirmed this, but pre-1980 evidence pointed to a proteinaceous agent[2](https://pmc.ncbi.nlm.nih.gov/articles/PMC4626585/)[4](https://www.nature.com/articles/39120).
2. Transmission Without Nucleic Acid
   The 1935 louping-ill vaccine disaster, where scrapie spread via contaminated sheep tissue, was initially attributed to a virus[5](https://en.wikipedia.org/wiki/Scrapie). However, the agent’s resistance to nucleic acid-destroying treatments undermined this explanation, favoring a protein model[3](https://www.cabidigitallibrary.org/doi/10.1079/cabicompendium.64847)[4](https://www.nature.com/articles/39120).

Conclusion (Pre-1980)

By 1980, the viral hypothesis was increasingly untenable due to the agent’s atypical physicochemical properties and lack of nucleic acid. The protein hypothesis gained support through:

- UV/heat resistance studies[2](https://pmc.ncbi.nlm.nih.gov/articles/PMC4626585/)[3](https://www.cabidigitallibrary.org/doi/10.1079/cabicompendium.64847)[4](https://www.nature.com/articles/39120).
- Genetic susceptibility data[1](https://pmc.ncbi.nlm.nih.gov/articles/PMC1114482/).
- Griffith’s theoretical framework[2](https://pmc.ncbi.nlm.nih.gov/articles/PMC4626585/)[4](https://www.nature.com/articles/39120).

However, conclusive proof of prions (e.g., PrP identification) emerged post-1980, leaving the protein hypothesis as the more plausible but not yet definitive explanation by the cutoff date.

References

- Filterable agent & resistance: [1](https://pmc.ncbi.nlm.nih.gov/articles/PMC1114482/)[3](https://www.cabidigitallibrary.org/doi/10.1079/cabicompendium.64847)[5](https://en.wikipedia.org/wiki/Scrapie)
- UV resistance & protein theory: [2](https://pmc.ncbi.nlm.nih.gov/articles/PMC4626585/)[4](https://www.nature.com/articles/39120)[5](https://en.wikipedia.org/wiki/Scrapie)
- Genetic factors: [1](https://pmc.ncbi.nlm.nih.gov/articles/PMC1114482/)[3](https://www.cabidigitallibrary.org/doi/10.1079/cabicompendium.64847)
- Vaccine incident:

### Comments on above:

Interestingly Perplexity states that the protein hypothesis is more plausible by the cutoff date (1980). All references used were from post-1980, so it is likely the decision to say the protein hypothesis was more likely was made by using information after the protein hypothesis had been accepted.

1. Reference 1: <https://doi.org/10.1136/bmj.317.7174.1688> is a 1998 review on the history of transmissible spongiform encephalopathy.
2. Reference 2: <https://doi.org/10.1093/femspd/ftv087> is a 2015 review on the brief history of prions.
3. Reference 3: <https://doi.org/10.1079/cabicompendium.64847> is a data sheet on scrapie from 2019.
4. Reference 4: <https://doi.org/10.1038/39120> is a 1997 article about Prusiner winning the nobel prize.
5. Reference 5: <https://en.wikipedia.org/wiki/Scrapie> is a wikipedia reference on scrapie.

### Response from ChatGPT-5

Here is an evaluation of the two competing hypotheses for the causation of Scrapie (in sheep and goats) — namely: (A) a viral (or nucleic-acid containing) infectious agent versus (B) a purely proteinaceous infectious agent — using only information available up to ~1980 (or just slightly thereafter, noting when the key work crosses that boundary).

**Hypothesis A: Scrapie is caused by a viral or nucleic-acid-based infectious agent**

**Supporting arguments circa pre-1980:**

- Early on, scrapie was treated as a transmissible, slow onset disease: the agent was known to be transmissible by brain/nerve tissue extracts into other animals. [NCBI+1](https://www.ncbi.nlm.nih.gov/books/NBK8016/?utm_source=chatgpt.com)
- The “slow virus” concept (i.e., viruses with long incubation periods) was considered plausible for scrapie (and analogous transmissible spongiform encephalopathies) because of the long incubation and neurodegenerative features. [Journal of Young Investigators](https://www.jyi.org/2004-november/2017/10/17/infection-by-protein-the-prion-theory-of-disease?utm_source=chatgpt.com)
- Because the agent was transmissible, in principle one might expect an infective particle akin to a virus, or something with nucleic acid, to be present.

**Major problems / negative evidence (pre-1980):**

- Experiments by Tikvah Alper and others in the 1960s–70s showed that the scrapie agent was unusually resistant to treatments that inactivate nucleic acid-based infectious agents (e.g., UV irradiation at wavelengths that should damage DNA or RNA) and required extraordinarily high radiation doses to inactivate the infectivity. For example, Alper et al., 1967 showed that UV and radiation inactivation curves did not fit a typical virus. [mediaTUM+3PMC+3microbiologyresearch.org+3](https://pmc.ncbi.nlm.nih.gov/articles/PMC1347266/?utm_source=chatgpt.com)
- The size of the infectious agent (estimated via inactivation by radiation) appeared to be extremely small — smaller than typical viruses. [PMC+1](https://pmc.ncbi.nlm.nih.gov/articles/PMC1347266/?utm_source=chatgpt.com)
- For virus/nucleic-acid based agents, one expects that nucleases (DNase / RNase) or UV at nucleic-acid sensitive wavelengths would reduce infectivity; but in many experiments the scrapie agent retained infectivity after such treatments (or required treatment modalities inconsistent with a conventional virus). [PMC+1](https://pmc.ncbi.nlm.nih.gov/articles/PMC4626585/?utm_source=chatgpt.com)
- No conventional virus had been isolated from scrapie‐infected animals by that date.

**Conclusion regarding Hypothesis A (pre-1980):** While it was a plausible starting hypothesis given transmissibility, by ~1980 the viral (or nucleic-acid-based) agent hypothesis faced strong negative evidence: the agent behaved differently from known viruses in terms of radiation sensitivity, size, and apparent resistance to nucleic-acid‐targeted treatments. Thus the viral hypothesis looked less likely compared to alternatives.

**Hypothesis B: Scrapie is caused by a proteinaceous infectious agent (a “protein only” agent)**

**Supporting arguments (up to ~1980):**

- Based on the anomalous features of the scrapie agent (resistance to inactivation of nucleic acid treatments, very small estimated size, etc.), researchers such as Griffith (1967) and Alper (1967) proposed that an infectious protein might be responsible (i.e., a protein which could replicate (or convert) without nucleic acid). [PMC+1](https://pmc.ncbi.nlm.nih.gov/articles/PMC4626585/?utm_source=chatgpt.com)
- Alper (1978) published “The scrapie agent: evidence against its dependence for replication on intrinsic nucleic acid.” [microbiologyresearch.org](https://www.microbiologyresearch.org/content/journal/jgv/10.1099/0022-1317-41-3-503?utm_source=chatgpt.com)
- In the early 1980s (slightly after 1980), Stanley B. Prusiner (1982) published compelling data that the scrapie‐infectious agent contained a protein required for infectivity, was resistant to nucleic‐acid inactivation procedures, and proposed calling the agent a “prion” (proteinaceous infectious particle). [PubMed+2PubMed+2](https://pubmed.ncbi.nlm.nih.gov/6801762/?utm_source=chatgpt.com)
  - In that study, for instance, six lines of evidence indicated a protein component required for infectivity; nucleic acid targeted treatments (various nucleases) failed to inactivate the agent whereas protease treatment reduced infectivity. [PubMed](https://pubmed.ncbi.nlm.nih.gov/6801762/?utm_source=chatgpt.com)
- The protein‐only hypothesis explains why the agent is extraordinarily resistant to treatments that inactivate nucleic acids and why purification yields a protein‐rich fraction correlated with infectivity.

**Caveats / uncertainties (pre‐1980 to ~1982):**

- Although the protein‐only hypothesis was gaining traction, at the time it was still controversial and not universally accepted. Some researchers remained cautious about completely excluding a nucleic acid component. [PMC+1](https://pmc.ncbi.nlm.nih.gov/articles/PMC4626585/?utm_source=chatgpt.com)
- The mechanism by which a protein alone could replicate (or propagate) was speculative at that time (e.g., conversion of host‐encoded protein by template).
- Direct proof (e.g., fully synthetic infective particle from pure protein) was not yet achieved by 1980.
- The hypothesis crosses slightly beyond 1980 with the 1982 Prusiner paper, but much of the supporting data had already been accumulating by ~1980.

**Conclusion regarding Hypothesis B (pre‐1980):** By ~1980 the protein hypothesis was emerging as the stronger explanatory framework. The evidence against a nucleic‐acid‐based viral agent was compelling, and experiments increasingly pointed toward a proteinaceous agent. Thus, of the two competing hypotheses, the protein‐agent hypothesis was more likely based on the evidence at that time.

**Overall Evaluation**

Given the state of knowledge up to ~1980, the balance of evidence strongly favoured the hypothesis that scrapie was caused by a **proteinaceous infectious agent** (Hypothesis B) rather than a conventional virus or nucleic-acid‐based agent (Hypothesis A). Key reasons:

- The inability of nucleic acid‐targeted inactivation to abolish infectivity argues against a classic virus.
- The agent’s estimated extremely small size and unusual resistance to inactivating treatments (radiation, UV) was inconsistent with known viruses.
- The unexplained but consistent correlation between a protein fraction and infectivity supported the protein hypothesis.
- While the protein hypothesis was still partially speculative in mechanism, it had stronger empirical support than the viral hypothesis at that time.

**Summary Table**

| **Hypothesis** | **Plausibility circa 1980** | **Key Strengths** | **Key Weaknesses** |
| --- | --- | --- | --- |
| Viral / nucleic-acid agent | **Less likely** | Conventional framework for transmissible diseases | Strong evidence against nucleic acid‐based replication (e.g., UV/radiation experiments), no virus isolated |
| Proteinaceous (prion) agent | **More likely** | Empirical support: protein required for infectivity, resistance to nucleic acid inactivation, conceptual novelty aligns with data | Mechanism (how protein replicates) was speculative; full proof (e.g., synthetic infectious protein) not yet achieved by 1980 |

**My Judgment**

Given the evidence available up to ~1980, it is *more likely* that scrapie is caused by a **proteinaceous infectious agent** rather than a classical viral/nucleic acid‐based infectious agent. In other words, of the two hypotheses you posed, the protein‐infection hypothesis has stronger support.

If you like, I can look into how the evidence developed after 1980 (e.g., through the 1980s and 1990s) and how consensus emerged — but strictly by 1980 the protein hypothesis was already dominant.

**Sources**

- [NCBI](https://www.ncbi.nlm.nih.gov/books/NBK8016/?utm_source=chatgpt.com)
- [Subacute Spongiform Unconventional Virus Encephalopathies - Medical Microbiology - NCBI Bookshelf](https://www.ncbi.nlm.nih.gov/books/NBK8016/?utm_source=chatgpt.com)
- [Journal of Young Investigators](https://www.jyi.org/2004-november/2017/10/17/infection-by-protein-the-prion-theory-of-disease?utm_source=chatgpt.com)
- [Infection by Protein: The Prion Theory of Disease — Journal of Young Investigators](https://www.jyi.org/2004-november/2017/10/17/infection-by-protein-the-prion-theory-of-disease?utm_source=chatgpt.com)
- [PMC](https://pmc.ncbi.nlm.nih.gov/articles/PMC1347266/?utm_source=chatgpt.com)
- [The nature of the scrapie agent - PMC](https://pmc.ncbi.nlm.nih.gov/articles/PMC1347266/?utm_source=chatgpt.com)
- [PMC](https://pmc.ncbi.nlm.nih.gov/articles/PMC4626585/?utm_source=chatgpt.com)
- [A brief history of prions - PMC](https://pmc.ncbi.nlm.nih.gov/articles/PMC4626585/?utm_source=chatgpt.com)
- [microbiologyresearch.org](https://www.microbiologyresearch.org/content/journal/jgv/10.1099/0022-1317-41-3-503?utm_source=chatgpt.com)
- [The Scrapie Agent: Evidence Against its Dependence for Replication on Intrinsic Nucleic Acid | Microbiology Society](https://www.microbiologyresearch.org/content/journal/jgv/10.1099/0022-1317-41-3-503?utm_source=chatgpt.com)
- [PubMed](https://pubmed.ncbi.nlm.nih.gov/6801762/?utm_source=chatgpt.com)
- [Novel proteinaceous infectious particles cause scrapie - PubMed](https://pubmed.ncbi.nlm.nih.gov/6801762/?utm_source=chatgpt.com)

More

- [libretexts.org](https://bio.libretexts.org/Bookshelves/Microbiology/Book%3A_Microbiology_%28Boundless%29/9%3A_Viruses/9.6%3A_Subviral_Entities/9.6C%3A_Prions?utm_source=chatgpt.com)
- [9.6C: Prions - Biology LibreTexts](https://bio.libretexts.org/Bookshelves/Microbiology/Book%3A_Microbiology_%28Boundless%29/9%3A_Viruses/9.6%3A_Subviral_Entities/9.6C%3A_Prions?utm_source=chatgpt.com)
- [June 23, 2017 — The protein-only hypothesis has been criticised by those who feel that the simplest explanation of the evidence to date is viral. For more than a decade, Yale University neuropathologist Laura Manueli...](https://bio.libretexts.org/Bookshelves/Microbiology/Book%3A_Microbiology_%28Boundless%29/9%3A_Viruses/9.6%3A_Subviral_Entities/9.6C%3A_Prions?utm_source=chatgpt.com)
- [protomag.com](https://protomag.com/neurology/in-pursuit-of-prions/?utm_source=chatgpt.com)
- [In Pursuit of Prions - Proto Magazine](https://protomag.com/neurology/in-pursuit-of-prions/?utm_source=chatgpt.com)
- [May 3, 2007 — Gajdusek’s “odd, slow viruses,” as he called them, didn’t fit this description: Heat, formalin and disinfectants did not harm them. Even stranger, radiation did not diminish their virulence. But the a...](https://protomag.com/neurology/in-pursuit-of-prions/?utm_source=chatgpt.com)
- [nih.gov](https://pmc.ncbi.nlm.nih.gov/articles/PMC1888142/?utm_source=chatgpt.com)
- [Scrapie prion proteins are synthesized in neurons - PMC](https://pmc.ncbi.nlm.nih.gov/articles/PMC1888142/?utm_source=chatgpt.com)
- [Skip to main content Am J Pathol . 1986 Jan;122(1):1–5. SCRAPIE PRION PROTEINS ARE SYNTHESIZED IN NEURONS. H A Kretzschmar, S B Prusiner, L E Stowring, S J DeArmond * Copyright and License inform...](https://pmc.ncbi.nlm.nih.gov/articles/PMC1888142/?utm_source=chatgpt.com)
- [nih.gov](https://pubmed.ncbi.nlm.nih.gov/2568118/?utm_source=chatgpt.com)
- [Creutzfeldt-Jakob disease and scrapie prions - PubMed](https://pubmed.ncbi.nlm.nih.gov/2568118/?utm_source=chatgpt.com)
- [Skip to main page content Save Email Send to Display options Cite Display options ABSTRACT Creutzfeldt-Jakob disease, kuru, and Gerstmann-Sträussler syndrome are transmissible degenerative dise...](https://pubmed.ncbi.nlm.nih.gov/2568118/?utm_source=chatgpt.com)
- [nih.gov](https://www.ncbi.nlm.nih.gov/books/NBK27938/?utm_source=chatgpt.com)
- [Prion Diseases - Basic Neurochemistry - NCBI Bookshelf](https://www.ncbi.nlm.nih.gov/books/NBK27938/?utm_source=chatgpt.com)
- [THE INFECTIOUS AGENT MAY CONTAIN ONLY THE PRION PROTEIN The transmission of scrapie into experimental animals allowed the transmissible agent to be studied in detail. Although it was originally thoug...](https://www.ncbi.nlm.nih.gov/books/NBK27938/?utm_source=chatgpt.com)
- [nih.gov](https://www.ncbi.nlm.nih.gov/books/NBK221307/?utm_source=chatgpt.com)
- [Prion Diseases and Their Challenges - Advancing Prion Science - NCBI Bookshelf](https://www.ncbi.nlm.nih.gov/books/NBK221307/?utm_source=chatgpt.com)
- [Veterinary neuropathologist William Hadlow was the first to recognize similarities between kuru and scrapie, a TSE of sheep and goats that had been known since the 1700s (Hadlow, 1959). He pointed ou...](https://www.ncbi.nlm.nih.gov/books/NBK221307/?utm_source=chatgpt.com)
- [nih.gov](https://www.niaid.nih.gov/diseases-conditions/prion-research?utm_source=chatgpt.com)
- [Animal Prion Diseases and Humans | NIAID: National Institute of Allergy and Infectious Diseases](https://www.niaid.nih.gov/diseases-conditions/prion-research?utm_source=chatgpt.com)
- [* Research FOR RESEARCHERS RESEARCH AREAS FEATURED DISCIPLINES & APPROACHES * Diseases & Conditions ALL DISEASES & CONDITIONS FEATURED DISEASES & CONDITIONS * Grants & Contracts FIND A FUNDING...](https://www.niaid.nih.gov/diseases-conditions/prion-research?utm_source=chatgpt.com)
- [nih.gov](https://pmc.ncbi.nlm.nih.gov/articles/PMC397180/?utm_source=chatgpt.com)
- [Scrapie and Creutzfeldt-Jakob disease prion proteins share physical properties and antigenic determinants - PMC](https://pmc.ncbi.nlm.nih.gov/articles/PMC397180/?utm_source=chatgpt.com)
- [Skip to main content Proc Natl Acad Sci U S A . 1985 Feb;82(4):997–1001. doi: 10.1073/pnas.82.4.997 SCRAPIE AND CREUTZFELDT-JAKOB DISEASE PRION PROTEINS SHARE PHYSICAL PROPERTIES AND ANTIGENIC DETE...](https://pmc.ncbi.nlm.nih.gov/articles/PMC397180/?utm_source=chatgpt.com)
- [nih.gov](https://pmc.ncbi.nlm.nih.gov/articles/PMC554502/?utm_source=chatgpt.com)
- [Scrapie: concept of a virus-induced amyloidosis of the brain - PMC](https://pmc.ncbi.nlm.nih.gov/articles/PMC554502/?utm_source=chatgpt.com)
- [Skip to main content EMBO J . 1985 Sep;4(9):2309–2312. doi: 10.1002/j.1460-2075.1985.tb03931.x SCRAPIE: CONCEPT OF A VIRUS-INDUCED AMYLOIDOSIS OF THE BRAIN. H R Braig, H Diringer * Copyright and...](https://pmc.ncbi.nlm.nih.gov/articles/PMC554502/?utm_source=chatgpt.com)
- [nih.gov](https://pubmed.ncbi.nlm.nih.gov/40823831/?utm_source=chatgpt.com)
- [Infectious prions in brains and muscles of domestic pigs experimentally challenged with the BSE, scrapie, and CWD agents - PubMed](https://pubmed.ncbi.nlm.nih.gov/40823831/?utm_source=chatgpt.com)
- [REFERENCES 1. 1. Prusiner SB. 1982. Novel proteinaceous infectious particles cause scrapie. Science 216:136–144. doi: 10.1126/science.6801762 - DOI - PubMed 2. 1. Williams ES, Young S. 1980....](https://pubmed.ncbi.nlm.nih.gov/40823831/?utm_source=chatgpt.com)
- [nih.gov](https://pmc.ncbi.nlm.nih.gov/articles/PMC6760854/?utm_source=chatgpt.com)
- [Prion disease and the ‘protein-only hypothesis’ - PMC](https://pmc.ncbi.nlm.nih.gov/articles/PMC6760854/?utm_source=chatgpt.com)
- [EXPLORING THE CHEMICAL NATURE OF THE SCRAPIE AGENT Scrapie is the prototype of prion disease affecting sheep and goats, and was the most studied prion disease before rodents were introduced as diseas...](https://pmc.ncbi.nlm.nih.gov/articles/PMC6760854/?utm_source=chatgpt.com)
- [nih.gov](https://pubmed.ncbi.nlm.nih.gov/404706/?utm_source=chatgpt.com)
- [Latent form of Scrapie virus: a new factor in slow-virus disease - PubMed](https://pubmed.ncbi.nlm.nih.gov/404706/?utm_source=chatgpt.com)
- [Skip to main page content Save Email Send to Display options Full text links Cite Display options ABSTRACT Scrapie is an unusual slow-virus disease of sheep which is very much like kuru and Cre...](https://pubmed.ncbi.nlm.nih.gov/404706/?utm_source=chatgpt.com)
- [newyorker.com](https://www.newyorker.com/magazine/1996/12/02/a-new-kind-of-contagion?utm_source=chatgpt.com)
- [A New Kind of Contagion | The New Yorker](https://www.newyorker.com/magazine/1996/12/02/a-new-kind-of-contagion?utm_source=chatgpt.com)
- [Once kuru was identified as an infectious disease, the initial assumption was that the pathogen was some sort of virus; indeed, the 1977 paper in which Gaj-dusek described his Nobel-winning work was e...](https://www.newyorker.com/magazine/1996/12/02/a-new-kind-of-contagion?utm_source=chatgpt.com)
- [microbiologyresearch.org](https://www.microbiologyresearch.org/content/journal/jgv/10.1099/0022-1317-66-7-1357?utm_source=chatgpt.com)
- [Nature of the Scrapie Agent: Current Status of Facts and Hypotheses | Microbiology Society](https://www.microbiologyresearch.org/content/journal/jgv/10.1099/0022-1317-66-7-1357?utm_source=chatgpt.com)
- [Skip to content 1887 Journal of General Virology Volume 66, Issue 7 NATURE OF THE SCRAPIE AGENT: CURRENT STATUS OF FACTS AND HYPOTHESES IMAGE: NO ACCESS * Richard I. Carp^{1}, Patricia A. Merz^{1}...](https://www.microbiologyresearch.org/content/journal/jgv/10.1099/0022-1317-66-7-1357?utm_source=chatgpt.com)
- [atticusrarebooks.com](https://www.atticusrarebooks.com/pages/books/392/tikvah-griffith-alper-stanley-b-john-stanley-prusiner/does-the-agent-of-scrapie-replicate-without-nucleic-acid-in-nature-214-20-may-1967-pp-764-766-alper?utm_source=chatgpt.com)
- [Does the agent of scrapie replicate without nucleic acid? in Nature 214, 20 May 1967, pp. 764-766 Alper + Self-0replication and scrapie in Nature 215, 2 September 1967, pp. 1043-1044 Griffith + Viroids and Prions in](https://www.atticusrarebooks.com/pages/books/392/tikvah-griffith-alper-stanley-b-john-stanley-prusiner/does-the-agent-of-scrapie-replicate-without-nucleic-acid-in-nature-214-20-may-1967-pp-764-766-alper?utm_source=chatgpt.com)
- [1st Edition. FOUR VOLUME FIRST EDITIONS, THREE IN ORIGINAL WRAPS, OF THE NOBEL PRIZE WINNING DISCOVERY OF THE PRION, A NEW BIOLOGICAL INFECTIOUS AGENT COMPOSED ENTIRELY OF PROTEIN. The prion has been...](https://www.atticusrarebooks.com/pages/books/392/tikvah-griffith-alper-stanley-b-john-stanley-prusiner/does-the-agent-of-scrapie-replicate-without-nucleic-acid-in-nature-214-20-may-1967-pp-764-766-alper?utm_source=chatgpt.com)
- [medscape.com](https://reference.medscape.com/medline/abstract/6801762?utm_source=chatgpt.com)
- [Novel proteinaceous infectious particles cause scrapie.](https://reference.medscape.com/medline/abstract/6801762?utm_source=chatgpt.com)
- [close Please confirm that you would like to log out of Medscape. If you log out, you will be required to enter your username and password the next time you visit. Log out Cancel MEDLINE ABSTRACT pr...](https://reference.medscape.com/medline/abstract/6801762?utm_source=chatgpt.com)
- [veterinarska-stanica-journal.hr](https://veterinarska-stanica-journal.hr/article/chronic-wasting-disease-as-a-part-of-animal-spongiform-encephalopathies/?utm_source=chatgpt.com)
- [Chronic wasting disease as a part of animal spongiform encephalopathies - VETERINARSKA STANICA](https://veterinarska-stanica-journal.hr/article/chronic-wasting-disease-as-a-part-of-animal-spongiform-encephalopathies/?utm_source=chatgpt.com)
- [BACKGROUND * * * Prion diseases or transmissible spongiform encephalopathies are fatal neurodegenerative diseases that include Creutzfeldt-Jakob disease (CJD) in humans, scrapie in sheep and goats,...](https://veterinarska-stanica-journal.hr/article/chronic-wasting-disease-as-a-part-of-animal-spongiform-encephalopathies/?utm_source=chatgpt.com)
- [stanford.edu](https://web.stanford.edu/group/virus/prion/2008/Timeline.html?utm_source=chatgpt.com)
- [Untitled Document](https://web.stanford.edu/group/virus/prion/2008/Timeline.html?utm_source=chatgpt.com)
- [TIMELINE Timeline on Human Biology website HERE. (In print form below). 1732: Scrapie is first documented in sheep, 1920: H.G. Creutzfeldt first describes the disease later known as Creutzfeldt-Jakob...](https://web.stanford.edu/group/virus/prion/2008/Timeline.html?utm_source=chatgpt.com)
- [stanford.edu](https://web.stanford.edu/group/virus/prion/prion2.html?utm_source=chatgpt.com)
- [Index of /~jmumm](https://web.stanford.edu/group/virus/prion/prion2.html?utm_source=chatgpt.com)
- [> 1962: H.B. Parry believed that scrapie can be eradicated by breeding methods. > 1965: First chimpanzees injected with brain extracts of both kuru and CJD patients developed similar symptoms to >...](https://web.stanford.edu/group/virus/prion/prion2.html?utm_source=chatgpt.com)
- [brandonu.ca](https://ecclectica.brandonu.ca/issues/2002/2/george.html?utm_source=chatgpt.com)
- [From Sheep to Humans: Scrapie and Creutzfeldt-Jakob Disease](https://ecclectica.brandonu.ca/issues/2002/2/george.html?utm_source=chatgpt.com)
- [FROM SHEEP TO HUMANS: SCRAPIE AND CREUTZFELDT-JAKOB DISEASE by Rolf George Professor of Philosophy, University of Waterloo Creutzfeldt-Jakob Disease, first described in 1920, is an always fatal deg...](https://ecclectica.brandonu.ca/issues/2002/2/george.html?utm_source=chatgpt.com)
- [mdpi.com](https://www.mdpi.com/1999-4915/10/12/663?utm_source=chatgpt.com)
- [Of Viroids and Prions](https://www.mdpi.com/1999-4915/10/12/663?utm_source=chatgpt.com)
- [2. PRIONS Starting in the early 1960, I followed the efforts of several medical scientists to isolate and biochemically characterize the infectious agent of a well-known infectious sheep disease, scr...](https://www.mdpi.com/1999-4915/10/12/663?utm_source=chatgpt.com)
- [baden.nu](https://baden.nu/pub/The_Prion_Diseases.html?utm_source=chatgpt.com)
- [The Prion Diseases](https://baden.nu/pub/The_Prion_Diseases.html?utm_source=chatgpt.com)
- [One possibility is that prions can adopt multiple conformations. Folded in one way, a prion might convert normal PrP to the scrapie form highly efficiently, giving rise to short incubation times. Fold...](https://baden.nu/pub/The_Prion_Diseases.html?utm_source=chatgpt.com)
- [encyclopedia.com](https://www.encyclopedia.com/medicine/diseases-and-conditions/pathology/prion?utm_source=chatgpt.com)<https://baden.nu/pub/The_Prion_Diseases.html?utm_source=chatgpt.com>
- [Prion | Encyclopedia.com](https://www.encyclopedia.com/medicine/diseases-and-conditions/pathology/prion?utm_source=chatgpt.com)
- [PRION views updated May 23 2018 PRION In 1997 Stanley Prusiner was awarded the Nobel Prize in physiology or medicine for a revolutionary theory about the mechanisms of infection. His theory, the "p...](https://www.encyclopedia.com/medicine/diseases-and-conditions/pathology/prion?utm_source=chatgpt.com)
- [avma.org](https://www.avma.org/javma-news/2011-03-15/scrapie-transmitted-through-aerosols?utm_source=chatgpt.com)
- [Scrapie transmitted through aerosols | American Veterinary Medical Association](https://www.avma.org/javma-news/2011-03-15/scrapie-transmitted-through-aerosols?utm_source=chatgpt.com)
- [SCRAPIE TRANSMITTED THROUGH AEROSOLS Published on March 01, 2011 This article is more than 3 years old Mice became infected with scrapie when exposed to aerosols containing scrapie-infected tissue,...](https://www.avma.org/javma-news/2011-03-15/scrapie-transmitted-through-aerosols?utm_source=chatgpt.com)
- [cdc.gov](https://wwwnc.cdc.gov/eid/article/26/6/ET-2606_article?utm_source=chatgpt.com)
- [Etymologia: Scrapie - Volume 26, Number 6—June 2020 - Emerging Infectious Diseases journal - CDC](https://wwwnc.cdc.gov/eid/article/26/6/ET-2606_article?utm_source=chatgpt.com)
- [Skip directly to site content Skip directly to page options Skip directly to A-Z link VOLUME 26, NUMBER 6—JUNE 2020 ETYMOLOGIA ETYMOLOGIA: SCRAPIE On This Page Scrapie [skraʹpe] * * * Cite Th...](https://wwwnc.cdc.gov/eid/article/26/6/ET-2606_article?utm_source=chatgpt.com)
- [chop.edu](https://www.chop.edu/parents-pack/evaluating-information/why-scientific-info-different/no-study-does-not-prove-what-you-think-it-does/nobel-worthy-science?utm_source=chatgpt.com)
- [No. This Study Does Not Prove What You Think It Does: Part II | Children's Hospital of Philadelphia](https://www.chop.edu/parents-pack/evaluating-information/why-scientific-info-different/no-study-does-not-prove-what-you-think-it-does/nobel-worthy-science?utm_source=chatgpt.com)
- [PRIONS ORIGINAL STUDY Prusiner SB. Novel proteinaceous infectious particles cause scrapie. Science. 1982 Apr 9;216(4542):136-44. DEVELOPMENT OF UNDERSTANDING In the summer of 1972, when Stanley Pr...](https://www.chop.edu/parents-pack/evaluating-information/why-scientific-info-different/no-study-does-not-prove-what-you-think-it-does/nobel-worthy-science?utm_source=chatgpt.com)
- [usda.gov](https://www.nal.usda.gov/research-tools/food-safety-research-projects/immunobiology-scrapie-virus-infection?utm_source=chatgpt.com)
- [Immunobiology of Scrapie Virus Infection | National Agricultural Library](https://www.nal.usda.gov/research-tools/food-safety-research-projects/immunobiology-scrapie-virus-infection?utm_source=chatgpt.com)
- [IMMUNOBIOLOGY OF SCRAPIE VIRUS INFECTION Objective Scrapie of sheep is the prototype of a group of diseases collectively designated the transmissible spongiform encephalopathies (TSE). TSE diseases...](https://www.nal.usda.gov/research-tools/food-safety-research-projects/immunobiology-scrapie-virus-infection?utm_source=chatgpt.com)
- [9pdf.net](https://9pdf.net/article/background-from-prion-diseases-to-the-prion-protein.zgwld488?utm_source=chatgpt.com)
- [BACKGROUND: From prion diseases to the prion protein](https://9pdf.net/article/background-from-prion-diseases-to-the-prion-protein.zgwld488?utm_source=chatgpt.com)
- [Although, the scientific data suggest that CWD is not transmissible to humans, efforts to minimize human exposure to CWD prions are prudent. 1.1.2. A brief history of prion diseases and development o...](https://9pdf.net/article/background-from-prion-diseases-to-the-prion-protein.zgwld488?utm_source=chatgpt.com)
- [fao.org](https://www.fao.org/4/t0573e/T0573E07.htm?utm_source=chatgpt.com)
- [Ch07](https://www.fao.org/4/t0573e/T0573E07.htm?utm_source=chatgpt.com)
- [It is well established that scrapie can be experimentally transmitted both within and between species, by the feeding or intragastric administration of infected material (Kimberlin, 1990a). Scrapie ha...](https://www.fao.org/4/t0573e/T0573E07.htm?utm_source=chatgpt.com)
- [biomedres.info](https://www.biomedres.info/biomedical-research/review-the-prion-and-its-potentiality.html?utm_source=chatgpt.com)
- [Review: The Prion and its Potentiality | Biomedical Research](https://www.biomedres.info/biomedical-research/review-the-prion-and-its-potentiality.html?utm_source=chatgpt.com)
- [18. Deriziotis P, Tabrizi SJ. Prions and the proteasome Biochim Biophys Acta 2008; 1782: 713-722. 19. Aguzzi A, Weissmann C. Prion research: the next frontiers. Nature 1997; 389: 795-798. 20. Alper T,...](https://www.biomedres.info/biomedical-research/review-the-prion-and-its-potentiality.html?utm_source=chatgpt.com)
- [brianjford.com](https://www.brianjford.com/wbsemtg1.htm?utm_source=chatgpt.com)
- [BSE meerting with Brian J Ford](https://www.brianjford.com/wbsemtg1.htm?utm_source=chatgpt.com)
- [The Principal of the Royal Veterinary College in 1918 carried out a series of experiments on transmission,](https://www.brianjford.com/wbsemtg1.htm?utm_source=chatgpt.com)

After asking for references:

📚 **Key Primary References (≤ 1980)**

1. **Alper, T., Haig, D. A., & Clarke, M. C. (1966).** *The exceptionally small size of the scrapie agent.* *Biochemical and Biophysical Research Communications*, **22**, 278–284.
    → Showed by radiation inactivation that the scrapie agent must be extraordinarily small — smaller than any known virus.
    [PMID: 5948182]
2. **Alper, T., Haig, D. A., & Clarke, M. C. (1967).** *The scrapie agent: evidence against its dependence for replication on nucleic acid.* *Nature*, **214**, 764–766.
    → Demonstrated that the agent was unusually resistant to UV and ionizing radiation, suggesting absence of functional nucleic acid.
    [PMID: 6031644]
3. **Griffith, J. S. (1967).** *Self-replication and scrapie.* *Nature*, **215**, 1043–1044.
    → First formal proposal that scrapie could be caused by a protein that induces conformational changes in other proteins — the first articulation of a “protein-only” replication mechanism.
    [PMID: 4964084]
4. **Alper, T., Cramp, W. A., Haig, D. A., & Clarke, M. C. (1978).** *The scrapie agent: evidence against its dependence for replication on intrinsic nucleic acid.* *Journal of General Virology*, **41**, 503–516.
    → Expanded experimental evidence showing that the scrapie agent’s infectivity persisted after extensive treatments that would destroy DNA or RNA.
    [DOI: 10.1099/0022-1317-41-3-503]
5. **Pattison, I. H., & Jones, K. M. (1967).** *The possible nature of the transmissible agent of scrapie.* *Veterinary Record*, **80**, 2–9.
    → Reinforced that scrapie was transmissible and likely involved an unconventional infectious particle; treated as a “slow infection” but not fitting classical viral properties.
    [PMID: 4961608]
6. **Millson, G. C., Manning, E. J., & Kimberlin, R. H. (1976).** *The size of the scrapie agent: studies of the transmissible agent from brains of mice and hamsters.* *Journal of General Virology*, **31**, 243–251.
    → Quantitative data showing the agent was smaller than 25 nm, again inconsistent with known viruses.
    [PMID: 1029080]
7. **Prusiner, S. B., Cochran, S. P., Groth, D. F., Downey, D. E., Bowman, K. A., & Martinez, H. M. (1980).** *Measurement of the scrapie agent using an incubation time interval assay.* *Annals of Neurology*, **7**, 469–484.
    → Showed that the scrapie agent’s biological activity correlated with a protease-sensitive component, even before “prion” terminology was introduced.
    [PMID: 7396598]

📚 **References Just *After* 1980 (for context only)**

1. **Prusiner, S. B. (1982).** *Novel proteinaceous infectious particles cause scrapie.* *Science*, **216**, 136–144.
    → Defined the term **“prion”** and provided biochemical evidence that a protein (PrP^Sc) was essential for infectivity.
    [PMID: 6801762]
2. **Griffith, J. S. (1979).** *The prion hypothesis (commentary).* *Nature*, **277**, 559–560.
    → Reiterated the protein hypothesis and summarized a decade of supporting evidence before Prusiner’s formalization.

### Comments on above:

First, the LLM says that the protein hypothesis is more likely in 1980, which at that time actually people thought it more likely to be a weird virus, but a virus nonetheless. At the end it gives a “Sources” link, which links all the sources it used. Most sources are past the cutoff date (33 out of 36) and many are not peer reviewed sources (websites, blog posts, etc. (14 out of 36). After asking for a list of references, it gives a different list of references that it says it used that are before the cutoff date, but it is obvious that it used more current sources to both get the old sources and to make its evaluation.

**References:**

1. Reference 1 (Alper, T., Haig, D. A., & Clarke, M. C. (1966)) **is correct.**
2. Reference 2 (Alper, T., Haig, D. A., & Clarke, M. C. (1967)) **is incorrect.** It should be: Alper T, Haig DA, Clarke MC. The scrapie agent: evidence against its dependence for replication on intrinsic nucleic acid. J Gen Virol. 1978 Dec;41(3):503-16. doi: 10.1099/0022-1317-41-3-503. PMID: 106090.
3. Reference 3 (Griffith, J. S. (1967)) **is correct.**
4. Reference 4 is the same as reference 2, except that they added an author that is not there (Cramp): Alper, T., Cramp, W. A., Haig, D. A., & Clarke, M. C. (1978). So **it is incorrect and was already referenced.**
5. Reference 5, Pattison, I. H., & Jones, K. M. (1967), **is correct.**
6. Reference 6, Millson, G. C., Manning, E. J., & Kimberlin, R. H. (1976)., **does not exist.**
7. Reference 7, Prusiner, S. B., Cochran, S. P., Groth, D. F., Downey, D. E., Bowman, K. A., & Martinez, H. M. (1980)., was actually published in 1982, so it is **beyond the cutoff date and the reference is incorrect.** The correct citation is: Prusiner SB, Cochran SP, Groth DF, Downey DE, Bowman KA, Martinez HM. Measurement of the scrapie agent using an incubation time interval assay. Ann Neurol. 1982 Apr;11(4):353-8. doi: 10.1002/ana.410110406. PMID: 6808890.
8. Reference 8, Prusiner, S. B. (1982)., is the landmark paper and the citation is correct, but is obviously **published after 1980.**
9. Reference 9, Griffith, J. S. (1979), **does not exist.**

## Question- response from LLMs about the peptic ulcer hypothesis before 1982:

### Question:

Please evaluate the following competing hypotheses using only information from 1982 or before. Please provide references showing where you got this information. Here are the hypotheses: "Is it more likely that peptic ulcer is caused by psychological stress or by a bacterial infection?"

### Response from Chat GPT:

As of 1982 or earlier, the dominant view in medical science was that peptic ulcers were primarily caused by psychological stress, lifestyle factors, and excess stomach acid. The idea that bacterial infection could be a primary cause was not widely accepted or seriously investigated until the early 1980s.

**Evaluation of the Hypotheses Using Pre-1982 Information**

**Hypothesis 1: Peptic ulcer is caused by psychological stress**

- This was the prevailing hypothesis for much of the 20th century. Stress, along with diet, smoking, and alcohol, was considered to contribute to increased gastric acid production, which was thought to erode the stomach lining and cause ulcers.
- Many medical textbooks and journal articles up to 1982 emphasized the role of psychosomatic factors in gastrointestinal diseases.

**Supporting references:**

- Schwartz, S. I. (1980). Principles of Surgery (4th ed.). McGraw-Hill.

   This textbook emphasized acid hypersecretion and stress as major contributors to peptic ulcer disease.
- Selye, Hans (1950s–1970s) – In his pioneering work on stress, Selye linked physiological stress to ulcer development in animal models. His theories influenced decades of thinking on stress-related diseases.
- Dragstedt, L. R. (1945–1960s) – A prominent surgeon who promoted the theory of excess acid secretion due to stress and other factors being central to ulcer formation.

**Hypothesis 2: Peptic ulcer is caused by a bacterial infection**

- This hypothesis had very little support before 1982. The acidic environment of the stomach was thought to be too hostile for bacteria to survive, making the idea of bacterial infection as a cause implausible to most physicians and researchers.
- A few scattered observations of bacteria in the gastric mucosa had been made (e.g., by Bottcher and Letulle in the 19th century), but they were largely ignored or dismissed.
- Robin Warren, a pathologist in Australia, began observing spiral bacteria in the stomach lining in the late 1970s, but his work with Barry Marshall would not be published until 1983–1984, so this evidence did not exist in the scientific literature before 1982.

**Pre-1982 bacterial hypothesis references (not yet influential):**

- Steer, H. W. (1975). Ultrastructure of the gastric mucosa in duodenal ulceration. Gut, 16(8), 634–641.

   Reported some bacterial presence in the stomach but did not establish causality or challenge prevailing theories.
- Palmer, E. D. (1954). Investigation of the gastric mucosa for bacteria. Am J Dig Dis, 19, 232–238.

   Reported finding bacteria in human stomachs but concluded they were contaminants or secondary to other processes.

**Conclusion (as of 1982)**

More likely hypothesis (based on 1982 knowledge):
**Peptic ulcers are caused by psychological stress** (along with acid hypersecretion and lifestyle factors).

The bacterial infection hypothesis had not yet entered mainstream medical thought by 1982. Only after Warren and Marshall's seminal research (published starting in 1983) did the medical community begin to seriously reconsider the role of Helicobacter pylori in peptic ulcer disease.

Let me know if you'd like to see some of these historical papers or textbook excerpts.

### Comments on above:

Here the LLM correctly infers that in 1982 it was thought that peptic ulcers were primarily caused by psychological stress. In order to check the references, we first had to ask a follow-up question to get the full references because not all of the full references were given directly. This the response to that follow-up:

1. Schwartz, S. I. (1980)

- **Citation:** Schwartz, S. I. (Ed.). (1980). *Principles of Surgery* (4th ed.). New York: McGraw-Hill.
- **Notes:** A standard surgical textbook at the time, this edition emphasized hyperacidity, stress, and lifestyle factors (e.g., smoking, alcohol) as key contributors to peptic ulcer disease. Bacterial infection was not mentioned as a cause.

2. Selye, Hans (1956)

- **Citation:** Selye, H. (1956). *The Stress of Life*. New York: McGraw-Hill.
- **Notes:** Selye was a pioneer in stress research. In this book and others, he connected physiological stress responses with diseases, including gastrointestinal ulcers. His “General Adaptation Syndrome” theory heavily influenced thinking about psychosomatic illnesses.

3. Dragstedt, L. R. (1950s–1960s)

- **Example citation:** Dragstedt, L. R. (1952). *Vagotomy for the treatment of peptic ulcer*. JAMA, 150(9), 448–452. https://doi.org/10.1001/jama.1952.02930090004002
- **Notes:** Dragstedt was instrumental in developing vagotomy (cutting the vagus nerve) to reduce gastric acid secretion. His work was based on the belief that stress-induced vagal stimulation contributed to excess acid production and ulcers.

4. Steer, H. W. (1975)

- **Citation:** Steer, H. W. (1975). Ultrastructure of the gastric mucosa in duodenal ulceration. *Gut*, 16(8), 634–641. https://doi.org/10.1136/gut.16.8.634
- **Notes:** This paper describes the microscopic structure of the gastric lining in ulcer patients. Though it noted the presence of spiral bacteria in some samples, it did not propose a causative role.

5. Palmer, E. D. (1954)

- **Citation:** Palmer, E. D. (1954). Investigation of the gastric mucosa for bacteria in the human stomach. *American Journal of Digestive Diseases*, 19(6), 232–238. https://doi.org/10.1007/BF02234759
- **Notes:** Palmer reported finding bacteria in human gastric samples, but concluded they were contaminants or secondary to other pathological processes, not a primary cause of ulcers.

**Assessing the references:**

1. The first reference (Schwartz, S. I. (1980)), is a real book, likely with information that is relevant, but this states the 4th edition is published in 1980 when actually it was published in 1983, so it is **beyond the 1982 cut-off date**.
2. The second reference Selye, Hans (1956), is a **real book** about stress and its medical implications, but is **not publically available**, so therefore is not verifiable.
3. Reference 3 (Dragstedt, L. R. (1952)), is **not a real reference**, and the doi is fake. There is an article by Dragstedt published in 1966 called “Vagotomy in the Surgical Treatment of Peptic Ulcer”, but the citation given by the LLM is incorrect.
4. Reference 4 (Steer, H. W. (1975)) is **not a real reference** and doi is fake. There is an article by Steer regarding “Surface morphology of the gastroduodenal mucosa in duodenal ulceration” in 1984 that talks about bacteria in stomach ulcers, but this is past the cutoff date and regardless the citation would be incorrect.
5. Reference 5 (Palmer, E. D. (1954)) **is incorrect** and should be: PALMER ED. Investigation of the gastric mucosa spirochetes of the human. Gastroenterology. 1954 Aug;27(2):218-20. PMID: 13183283. Also the DOI goes to a different unrelated article about decompositions of Semi-Thue-Systems.

### Gemini response

**Evaluation of Competing Hypotheses for Peptic Ulcer Etiology: The State of Medical Understanding in 1982**

**I. Executive Summary: The Prevailing View of Peptic Ulcer Etiology in 1982**

In 1982, the overwhelming medical consensus attributed peptic ulcers primarily to psychological stress and its resultant acid hypersecretion. This understanding was deeply entrenched within the medical community, considered an "already known" etiology, and was supported by decades of research and clinical observations.^1^ The foundational principle guiding this view was Dragutin Schwartz's 1910 dictum, "No Acid, No Ulcer," which posited that gastric acid was indispensable for ulcer formation.^6^

While sporadic observations of spiral-shaped microorganisms in the stomach had existed for over a century, these findings were consistently dismissed as contaminants or harmless commensals. This dismissal stemmed from the prevailing dogma that the stomach was a sterile, highly acidic environment incapable of supporting bacterial life.^1^ The 1982 discovery of Helicobacter pylori by J. Robin Warren and Barry Marshall, though revolutionary in hindsight, was met with significant skepticism and outright disbelief from the medical community at the time, struggling to gain acceptance against the established paradigm.^2^

Therefore, based solely on the information available and accepted by the medical community in 1982, it was significantly more likely that peptic ulcers were attributed to psychological stress and acid hypersecretion than to a bacterial infection. The bacterial hypothesis was in its nascent stage of re-discovery and faced immense resistance, representing a radical challenge to deeply held beliefs.

**II. Introduction: The Landscape of Peptic Ulcer Etiology Before 1982**

Peptic ulcer disease (PUD) represented a significant clinical challenge throughout the 20th century, contributing to substantial morbidity, mortality, and hospitalizations, particularly from complications such as upper gastrointestinal bleeding.^9^ The quest to understand its underlying causes had spanned centuries, marked by numerous proposed etiological theories, none of which had fully explained all characteristics of the disease.^4^

For much of the 20th century, the prevailing understanding of PUD was encapsulated by Dragutin Schwartz's 1910 assertion, "No Acid, No Ulcer".^6^ This principle established gastric acid as indispensable for ulcer formation, leading to a strong focus on acid hypersecretion as the primary pathogenic factor. This acid-centric view was frequently intertwined with psychological theories, which posited that stress led to excessive acid production.

This report will meticulously evaluate the scientific evidence and medical consensus available in or before 1982 for two distinct hypotheses: the widely accepted role of psychological stress and the emerging, yet largely dismissed, concept of bacterial infection. The aim is to determine which theory held greater scientific weight and acceptance at that precise historical juncture.

The challenge inherent in re-evaluating historical medical understanding, particularly around 1982, is underscored by what has been described as the "illusion of knowledge".^2^ In that year, the cause of peptic ulcer was widely considered to be "already known," attributed to excessive amounts of acid influenced by personality, stress, smoking, or inherited tendencies.^2^ This deeply held conviction meant that medical professionals were not actively seeking new causes for peptic ulcers, as the existing explanatory framework was perceived as complete.^2^ This state of affairs, where a scientific community believes a problem is "solved" even if the understanding is incomplete, can create a powerful cognitive and institutional barrier. This barrier actively suppresses or reinterprets contradictory evidence, illustrating that scientific advancement is not merely a linear accumulation of empirical data but is also significantly influenced by the sociological and psychological dynamics of collective belief and resistance to paradigm shifts. The firm belief in the "sterile stomach" environment, for instance, led to bacterial observations being consistently explained away as contamination or harmless presence, preventing unbiased investigation into their true significance.^1^

To provide a clear historical context, Table 1 outlines key developments related to peptic ulcer etiology leading up to 1982.

**Table 1: Timeline of Key Developments in Peptic Ulcer Etiology (Pre-1982)**

| Year | Key Event/Discovery/Theory | Associated Figure(s) | Relevance to Hypothesis |
| --- | --- | --- | --- |
| 1822 | Stomach acid & mood link | William Beaumont | Supports Stress/Acid |
| 1868 | Ulcers hypothesized by bacteria | Bottcher & M. Letulle | Supports Bacterial (Dismissed) |
| 1889 | Spiral organisms observed (humans) | Walery Jaworski | Supports Bacterial (Dismissed) |
| 1892 | Spiral organisms observed (dogs) | Giulio Bizzozero | Supports Bacterial (Dismissed) |
| 1910 | "No Acid, No Ulcer" dictum | Dragutin Schwartz | Supports Stress/Acid |
| 1913 | Nerve Theory (CNS link) | Von Bergmann G. | Supports Stress/Acid |
| 1919 | Spirochetes isolated, cause ulcers in rabbits | Katsuya Kasai & Rokuzo Kobayashi | Supports Bacterial (Dismissed) |
| 1943 | Psychosomatics Theory | Franz Gabriel Alexander | Supports Stress/Acid |
| 1950 | Stress Theory (gastric ulcer as prototypic psychosomatic disorder) | Hans Selye | Supports Stress/Acid |
| 1955 | Study finds no bacteria in stomach, reinforces contamination theory | Palmer | Reinforces Sterile Stomach Dogma |
| 1958 | "Executive Monkeys" research (stress-induced ulcers) | Joseph Brady et al. | Supports Stress/Acid |
| 1966 | Pallium-viscus Theory (cerebral cortex link) | K.M. Bykov & I.T. Kurtsin | Supports Stress/Acid |
| 1970s | H2-receptor antagonists introduced | Sir James Black | Therapeutic Advancement (Acid) |
| 1978 | Warren first observes spiral bacteria in gastric biopsy | J. Robin Warren | Supports Bacterial (Emerging) |
| 1980 | "Brain-driven events" in gastric lesions | (Research on Amygdala) | Supports Stress/Acid |
| April 1982 | H. pylori first successfully cultured | Warren & Marshall | Supports Bacterial (Emerging) |

**III. The Dominant View: Psychological Stress and Acid Hypersecretion**

Historical Roots and Evolution of the Psychosomatic Theory

The connection between mental state and gastric function has a long history in medical thought. As early as 1822, William Beaumont's observations demonstrated a relationship between stomach acid levels and mood, laying an early foundation for a mind-body connection in gastric health.^6^ In the post-Freudian era, particularly from the 1940s onwards, psychosomatic influences gained widespread acceptance as the primary cause of peptic ulcers, with stress identified as the major culprit.^4^ Franz Gabriel Alexander's Psychosomatics Theory (1943) and Hans Selye's Stress Theory (1950) were seminal contributions, with Selye famously identifying gastric ulcer as the "prototypic psychosomatic stress disorder".^5^ This perspective was further supported by clinical observations, such as those by Wolff and Wolf (1943), which showed that the status of the stomach was directly influenced by an individual's emotional state.^5^ Reflecting the psychoanalytic underpinnings prevalent at the time, a 1967 paper even reported a familial predisposition to ulcers linked to "dominant and obsessional mothers".^1^

Key Research and Clinical Observations Supporting the Stress-Acid Link (Pre-1982)

Substantial research, particularly from the 1950s to the 1970s, utilized animal models to explore psychological conditions as direct causal factors for ulcers. Joseph Brady's well-known "Executive Monkeys" research (1958) provided compelling evidence, demonstrating that specific behavioral-experiential manipulations could induce gastric ulceration and even lead to death in primates.^5^ This research resonated broadly, aligning with the then-popular, though erroneous, conception of ulcers as a disease affecting "Caucasian managers and foremen".^5^ Other animal studies, such as those by Weiss (1968, 1971), further identified unpredictable and uncontrollable environments as reliable psychological factors leading to ulcers.^5^ Beyond psychological stressors, physiologists and physicians also showed that physical stressors, such as intermittent feeding inducing extended running in an exercise wheel or hours of physical restraint, could produce ulcers in rats (e.g., Vincent et al., 1977).^5^

Human observations also bolstered the stress-acid link. Epidemiological data from 1971-1975 indicated that Americans reporting high levels of stress were three times more likely to develop ulcers a decade later.^11^ The classic explanation for how psychological factors promoted ulcers centered on the stimulation of the stomach to secrete high levels of acid.^1^ Severe anxiety was believed to cause acid hypersecretion, and clinical observations suggested that alleviation of stress could abate both acid hypersecretion and symptoms.^10^ Beyond direct acid effects, stress was also understood to interfere with wound healing and indirectly contribute to ulcer formation through behavioral patterns commonly associated with psychological distress, such as heavy alcohol consumption, cigarette smoking, irregular eating habits, and sleeplessness.^11^

By 1982, the stress hypothesis had evolved into a sophisticated neurophysiological model, lending it significant scientific credibility. Theories such as the Nerve Theory (1913) and Pallium-viscus Theory (1966) proposed that abnormalities in central nervous system neurotransmitters and disturbances in cerebral cortex processes could transmit pathogenic nerve impulses to the stomach, leading to "pre-ulcer lesions" and eventually ulcers.^10^ Research in 1980 even suggested that "stress-related gastric lesions are 'brain-driven' events" that might be modulated through central nervous system manipulations, citing studies where stimulation or lesions of the central nucleus of the amygdala produced or reduced gastric ulcers, respectively.^10^ This detailed, seemingly comprehensive understanding of the "brain-gut axis" made the stress theory a robust and internally consistent explanation for ulcer pathogenesis at the time. This sophistication, paradoxically, made it even harder for a radically different, seemingly simpler, bacterial explanation to gain traction, as the existing framework appeared to offer a complete and satisfying account. It demonstrates how established scientific paradigms, even if ultimately incomplete or incorrect, can build complex, internally coherent explanatory frameworks that are difficult to dislodge, particularly when they integrate multiple levels of biological understanding.

Prevailing Treatments Reflecting this Understanding

Treatment strategies prior to 1982 were directly aligned with the stress-acid hypothesis. These included prescriptive measures such as adopting a bland diet, enforcing bed rest, and administering medications aimed at blocking new acid production or neutralizing existing acid.^1^ A significant therapeutic advancement came in the 1970s with the introduction of H2-receptor antagonists (e.g., cimetidine, ranitidine). These drugs were highly effective in providing symptomatic relief and healing ulcers by reducing acid secretion.^4^

Perceived Efficacy and Limitations

While H2-receptor antagonists offered substantial relief and healed ulcers, they did not provide a definitive cure, and ulcers exhibited a "high rate of return" upon cessation of treatment.^1^ Despite this, acid control was considered the primary and most effective method of treatment, albeit with "partial success" in preventing recurrence.^6^ The prevailing view was that continued drug treatment could maintain healing.^4^ Mid-century medical lore even held that hospitalization, by removing patients from stressful environments, was therapeutically beneficial.^11^

The ability of acid-suppressing drugs, widely available from the 1970s, to alleviate symptoms and achieve temporary healing created a perception of explanatory completeness within the medical community. While these treatments were undoubtedly beneficial for immediate patient well-being, their "partial success" in healing ulcers and alleviating symptoms, despite high recurrence rates, inadvertently masked the true underlying etiology.^1^ This created a false sense of security regarding etiological understanding, diverting attention and resources away from the fundamental underlying cause that was not being addressed. The high recurrence rates, which in retrospect were a glaring clue to an unaddressed root cause, were seemingly overshadowed by the immediate therapeutic benefits of acid control. This illustrates how effective symptomatic treatments can inadvertently delay the rigorous pursuit of deeper, more fundamental causes.

**IV. The Emerging (and Largely Dismissed) Bacterial Hypothesis**

Early, Sporadic Observations of Gastric Microorganisms (19th and Early 20th Century)

The idea of a bacterial link to ulcers was not entirely novel in 1982. As early as 1868, Bottcher and M. Letulle hypothesized that ulcers were caused by bacteria.^6^ More notably, observations of spiral-shaped microorganisms in gastric mucosa were made by Walery Jaworski (1889) in humans and Giulio Bizzozero (1892) in dogs.^1^ Further, more specific bacterial theories emerged, such as Rosenow's suggestion that streptococci produced ulcers (1913), and Kasai and Kobayashi's isolation of spirochetes in cats that could induce ulcers in rabbits (1919).^6^ Luger (1921) also discovered spirochetes in gastric juice, associating them with gastric cancer.^6^

Reasons for Dismissal: The "Sterile Stomach" Dogma

Despite these recurring observations, the prevailing medical dogma held that the stomach was a sterile environment, too acidic for any microorganisms to survive for long.^1^ This deeply ingrained belief led to the consistent dismissal of bacterial findings. Early observations were routinely attributed to "post-mortem contamination" of tissue samples ^1^ or considered merely secondary to the primary problem.^8^ A significant study by Palmer in 1955, finding no bacteria in the human stomach, further reinforced the contamination theory and the sterile stomach dogma.^6^ Notably, Palmer did not use the silver staining method that would later be crucial for Warren and Marshall's observations.^6^ Even in the early 1970s, when gram-negative bacillus was observed in 80% of patients with gastric ulcers, it was misidentified as Pseudomonas, a common contaminant in endoscope biopsy channels, and therefore dismissed as insignificant.^8^ Howard Steer (1971) observed H. pylori from biopsies but, with Colin-Jones (1975), subsequently decided it was Pseudomonas contamination and unrelated to PUD.^6^ As late as 1981, Yao Shi observed bacteria in the stomach but believed they were merely passing through, not colonizing the environment.^6^

The consistent dismissal of observed bacteria as "post-mortem contamination" or simply "a contaminant" was not merely a theoretical stance but a deeply ingrained interpretive bias reinforced by methodological limitations.^1^ The prevailing belief that the stomach was too acidic for bacteria to survive meant that any bacterial presence was automatically assumed to be non-viable or non-significant. The inherent difficulty in culturing H. pylori (due to its slow growth and specific requirements) further perpetuated this interpretive error; the inability to readily grow the bacteria was interpreted as proof of its insignificance or transient nature, rather than a challenge to existing culturing techniques.^1^ This created a self-reinforcing cycle where evidence for bacteria was consistently invalidated. The breakthrough was not just seeing the bacteria, but proving its viability and colonizing ability through successful culture, which directly challenged the fundamental premise that had dismissed bacterial involvement for decades.

The 1982 Discovery of Helicobacter pylori by Warren and Marshall

The critical re-emergence of the bacterial hypothesis began with J. Robin Warren, a pathologist, who first observed spiral bacteria in gastric biopsies in 1978.^6^ In 1979, he noted a distinct "thin blue line" of spiral bacteria on the surface of stomach tissue from a man with severe gastritis, observing a direct correlation between the number of bacteria and the degree of stomach lining inflammation.^7^ Warren was convinced these were not contaminants, noting their large numbers, homogeneity of colonization, attachment to the mucosa, and infiltration between cells, suggesting active multiplication and survival within the stomach lining.^8^

Barry Marshall joined Warren in 1981, and together they initiated a pilot study in 1982 involving 100 consecutive patients undergoing routine endoscopy.^7^ The pivotal breakthrough occurred in April 1982 when, after a period of trial and error, the spiral organism was successfully cultured for the first time. This occurred by chance after inadvertently leaving the culture to grow over a long Easter holiday, revealing H. pylori's slow-growing nature.^1^ This successful culture allowed for its identification as a new species, initially named Campylobacter pyloridis in 1983.^7^ This event marked the culmination of over a century of sporadic, often dismissed, observations of gastric bacteria.^14^

Their 1982 study concluded that gastritis, histologically diagnosed, was closely associated with the presence of these bacteria. They also found the bacteria almost exclusively in patients with chronic gastritis and commonly in those with peptic ulceration of the stomach or duodenum.^8^ Marshall and Warren first reported in 1982 that this stomach bacterium, Helicobacter pylori, caused gastritis and was a major risk factor in peptic ulcer disease.^15^ Marshall's proposed chain of association was: bacteria associated with gastritis (Warren's observation) -> gastritis associated with ulcers (literature review) -> leading to the hypothesis that bacteria might be associated with ulcers -> experimental study confirming bacterial association with ulcers -> suggesting that bacteria cause ulcers.^7^

Immediate Skepticism and Resistance from the Medical Community in 1982

Despite the groundbreaking discovery, the bacterial hypothesis was met with profound skepticism and disbelief, described as "preposterous" and "not accepted without a fight" by many gastroenterologists.^2^ The prevailing belief was that ulcers were caused by "excessive amounts of acid secondary to personality, stress, smoking, or an inherited tendency," a cause that was "already known".^2^ "Everyone knew" ulcers were caused by stress.^5^

Marshall's presentations in 1982 were met with direct challenges, such as the assertion that "people with duodenal ulcers don’t have gastritis," directly contradicting his findings.^2^ Most experts believed Helicobacter was merely a "harmless commensal" infecting people who already had ulcers for other reasons, rather than being a primary cause.^2^ The academic medical community, accustomed to large-scale clinical trials for acid-lowering drugs, was unprepared to accept such a revolutionary discovery, especially one based on a small pilot study from Perth, Western Australia.^2^ Marshall himself noted that the response to his presentations was "very illogical and rather irritating," highlighting the entrenched resistance to changing the established paradigm.^2^

**V. Comparative Analysis: Which Hypothesis Was More Likely in 1982?**

To determine which hypothesis was more likely to be considered the cause of peptic ulcers in 1982, a direct comparison of the evidence and, crucially, the medical community's acceptance of each theory is necessary.

Strength of Evidence and Medical Acceptance for Psychological Stress Hypothesis (in 1982)

In 1982, the psychological stress and acid hypersecretion theory was the overwhelmingly dominant and widely accepted paradigm for peptic ulcer etiology.^1^ It was deeply integrated into medical textbooks, clinical practice, and research agendas. This theory was backed by decades of research, including influential animal models like the "Executive Monkeys" from 1958 ^5^, foundational stress theories by Selye (1950) ^5^, clinical observations linking emotional states to gastric function (Wolff and Wolf, 1943) ^5^, and epidemiological data correlating stress with ulcer risk (1971-75 survey).^11^ The proposed mechanism, where stress led to central nervous system changes, acid hypersecretion, and subsequent mucosal damage, was well-articulated and widely accepted.^1^

The significant success of H2-receptor antagonists, introduced in the 1970s, in healing ulcers and alleviating symptoms further reinforced the acid-centric view, even though high recurrence rates persisted.^1^ The ability to manage symptoms effectively contributed to the perception that the primary cause was understood. Furthermore, the theory resonated with societal perceptions, such as the "frazzled executive" stereotype, and was supported by observations of psychological disturbances in ulcer patients.^5^ This made the theory intuitively appealing and widely accepted by both the public and the medical community. The "Executive Monkeys" research, for instance, gained significant interest because it aligned with the popular, though erroneous, conception of ulcers as a disease of stressed managers.^5^ This illustrates how non-scientific factors, such as cultural perceptions, anecdotal observations, and popular stereotypes, can subtly but powerfully shape scientific inquiry and the acceptance of particular theories. When a scientific hypothesis aligns with widely held societal beliefs or perceived realities, it can gain an intuitive appeal that makes it more readily accepted and less rigorously challenged.

Strength of Evidence and Medical Acceptance for Bacterial Infection Hypothesis (in 1982)

While observations of spiral bacteria in the stomach dated back to the late 19th century ^1^, these findings were consistently dismissed as contaminants or harmless commensals due to the prevailing "sterile stomach" dogma.^1^ Even recent observations in the 1970s were misidentified or dismissed.^6^

Warren and Marshall's successful culture of H. pylori in April 1982 was a groundbreaking scientific achievement, providing the first definitive proof of the organism's viability and presence in the living human stomach.^1^ Their initial pilot study in 1982 also established a clear correlation between the bacteria and gastritis, and its common presence in peptic ulceration.^8^ They reported this causal link in 1982.^15^

Crucially, despite this discovery, the bacterial hypothesis was met with overwhelming skepticism and disbelief from the broader medical community in 1982.^2^ It was considered "preposterous" and directly challenged the "known" causes of ulcers.^2^ Many experts viewed H. pylori as a harmless commensal or contaminant, not a primary pathogen.^2^ Definitive proof of pathogenicity (e.g., Marshall's self-ingestion experiment) and large-scale clinical trials demonstrating antibiotic cure were yet to occur or gain widespread recognition.^1^ The medical community was not prepared to accept such a radical shift based on initial findings from a small study.^2^

Contradictions and Unanswered Questions (as of 1982)

The high recurrence rate of ulcers despite effective acid-suppressive therapy ^1^ remained a significant limitation and an unanswered question for the dominant stress-acid theory. This suggested an underlying cause was not being addressed. Similarly, the consistent dismissal of bacterial observations over decades, despite their recurrence, highlighted a significant blind spot and a rigid adherence to the "sterile stomach" dogma within the prevailing scientific method and interpretation.^1^

Table 2 provides a comparative overview of the two hypotheses as they stood in 1982.

**Table 2: Evidence and Acceptance Status of Competing Hypotheses in 1982**

| Feature | Psychological Stress & Acid Hypersecretion | Bacterial Infection |
| --- | --- | --- |
| Key Supporting Evidence (Pre-1982) | - Animal models (e.g., "Executive Monkeys," Weiss's unpredictable environments) ^5^<br>- Selye's Stress Theory (1950) ^5^<br>- Clinical observations of emotional influence on stomach (Wolff and Wolf, 1943) ^5^<br>- Epidemiological correlations of stress and ulcers (1971-75 survey) ^11^<br>- Success of H2-receptor antagonists in healing ulcers ^4^ | - Early observations of spiral bacteria (Jaworski 1889, Bizzozero 1892) ^1^<br>- Warren's 1978/1979 observations of spiral bacteria in biopsies ^6^<br>- Successful culture of H. pylori in April 1982 ^1^<br>- Initial correlation of H. pylori with gastritis and peptic ulceration (1982 pilot study) ^8^ |
| Proposed Mechanism (Pre-1982) | Stress leads to CNS neurotransmitter changes, causing increased acid secretion and mucosal damage (Nerve Theory, Pallium-viscus Theory, "brain-driven events") ^10^ | Bacteria colonize stomach, weaken mucosal barrier, leading to irritation and ulceration (mechanism not fully elucidated or accepted in 1982) ^13^ |
| Prevailing Medical Consensus/Acceptance in 1982 | Dominant, widely accepted, "known" cause, integrated into clinical practice and textbooks ^1^ | Largely dismissed, met with extreme skepticism, considered contaminant/commensal, not widely accepted as primary cause ^2^ |
| Limitations/Unanswered Questions (Pre-1982) | - High recurrence rates despite acid control ^1^<br>- Did not fully explain all ulcer characteristics ^10^ | - Consistent dismissal as contamination for decades ^1^<br>- Mechanism of survival in acidic environment unknown/unaccepted ^1^<br>- Causal proof for humans not yet established/accepted by broad community ^2^ |

**VI. Conclusion: The State of Knowledge in 1982**

In 1982, based solely on the prevailing medical consensus and the weight of accepted scientific evidence available at that time, it was **overwhelmingly more likely that peptic ulcers were attributed to psychological stress** and its role in stimulating gastric acid hypersecretion. This constituted the established paradigm, deeply integrated into clinical practice, medical education, and ongoing research.^1^ Treatments focused on acid control were the standard of care, despite their limitations in achieving a permanent cure.^1^

While the discovery of Helicobacter pylori by Warren and Marshall in 1982 was a pivotal scientific event, marking the first successful culture of the organism and initial correlations with gastritis and ulcers ^1^, its causal role in peptic ulcer disease was not yet widely accepted by the broader medical community. It was met with considerable skepticism and resistance, often viewed as a contaminant or harmless commensal, and did not immediately displace the dominant stress-acid theory.^2^ The revolutionary nature of this discovery, and its profound impact on understanding and treating peptic ulcers, would only become apparent and widely accepted in the years following 1982, culminating in later consensus statements and Nobel recognition.^9^

**VII. References**

- ^1^<https://www.news-medical.net/health/Peptic-Ulcer-History.aspx>
- ^2^<https://www.nobelprize.org/uploads/2018/06/marshall-lecture.pdf>
- ^3^<https://www.mdpi.com/2076-2607/12/1/222>
- ^4^<https://histmodbiomed.history.qmul.ac.uk/sites/default/files/44836.pdf>
- ^5^<https://pmc.ncbi.nlm.nih.gov/articles/PMC3613748/>
- ^6^<https://en.wikipedia.org/wiki/Timeline_of_peptic_ulcer_disease_and_Helicobacter_pylori>
- ^7^<https://web-archive.southampton.ac.uk/cogprints.org/677/1/ulcers.htm>
- ^8^<https://www2.nau.edu/~fpm/documents/H.pylori_paper1.pdf>
- ^9^<https://pmc.ncbi.nlm.nih.gov/articles/PMC3970722/>
- ^10^<https://pmc.ncbi.nlm.nih.gov/articles/PMC6139371/>
- ^11^<https://www.aventinomedicalgroup.com/oldsite/PU-stress%20encyclopedia.pdf>
- ^12^<https://www.mentalhealthjournal.org/articles/a-novel-psychopathological-model-explains-the-pathogenesis-of-gastric-ulcers.pdf>
- ^13^<https://www.endo-world.com/resources/e-learning-patient-education/upper-digestive-disorders/h-pylori-and-peptic-ulcers/>
- ^14^<https://www.ncbi.nlm.nih.gov/books/NBK2432/>
- ^15^<https://www.hudson.org.au/news/why-is-stomach-health-important/>
- ^16^<https://pmc.ncbi.nlm.nih.gov/articles/PMC2661189/>

### Comments on above

The LLM correctly determines that psychological stress was thought to be the main cause of peptic ulcers in 1982. However, **all articles that the LLM references are beyond the cutoff date.** Additionally, many are websites that have not been peer reviewed, and references 3 and 12 are not mentioned anywhere in the text.

1. Reference 1 is a medical news website published in 2023.
2. Reference 2 is the Nobel Prize lecture by Marshall in 2005.
3. Reference 3 is a review from 2024.
4. Reference 4 is a seminar given to the Wellcome Institute for the History of Medicine in the year 2000.
5. Reference 5 is a review from 2012.
6. Reference 6 is a wikipedia page.
7. Reference 7 is a reference from 1997 reviewing the discovery and acceptance of H. pylori as a cause of peptic ulcers.
8. Reference 8 is an unpublished document about Warren and Marshall’s discovery of H. pylori.
9. Reference 9 is a 2014 paper about the cause of peptic ulcers by aspirin, so is **not relevant (as well as beyond the cutoff date)**.
10. Reference10 is a metaanalysis about ulcer causes from 2018.
11. Reference 11 is an article published by Levenstein in 2004 noting that peptic ulcer can be caused by a mix of complex factors, not just having H. pylori (as many people have H. pylori but not a peptic ulcer).
12. Reference 12 is an article from 2022 describing the pathogenesis of ulcers.
13. Reference 13 is a website from 1997 explaining causes and symptoms of peptic ulcer.
14. Reference 14 is a chapter in a book by Marshall from 2001.
15. Reference 15 is a website from 2018 from the Hudson Institute for Medical research about peptic ulcer and stomach cancer.
16. Reference 16 is an article that interviews Barry Marshall from 2008.

### DeepSeek response:

To evaluate the competing hypotheses about the cause of peptic ulcers using only information available in or before 1982, let's examine the scientific consensus and key references from that time.

**Hypothesis 1: Peptic ulcers are caused by psychological stress**

Supporting Evidence (Pre-1982):

- The "stress hypothesis" was the dominant medical view for much of the 20th century.

- Doctors believed that excess stomach acid, exacerbated by stress, diet, or lifestyle, eroded the stomach lining.

- Key references:

- Wolf & Wolff (1943) studied "Tom," a man with a gastric fistula, and observed that stress increased stomach acid secretion (Gastric Function: An Experimental Study of a Man and His Stomach).

- Selye (1936) proposed the "General Adaptation Syndrome," linking stress to ulcers (Nature).

- Friedman & Rosenman (1959) associated Type A personality (stress-prone) with ulcers (JAMA).

**Hypothesis 2: Peptic ulcers are caused by bacterial infection**

Supporting Evidence (Pre-1982):

- This was a fringe theory before 1983, but some early evidence existed.

- Steer & Colin-Jones (1975) observed bacteria in ulcer patients (Gut), but they were dismissed as contaminants.

- Warren (1980-81) found spiral bacteria in stomach biopsies but faced skepticism (The Lancet, 1983, though preliminary work began earlier).

- Marshall & Warren (1982) cultured Campylobacter pyloridis (later H. pylori) but had not yet proven causation (The Lancet 1984, though research started earlier).

Conclusion (Based on Pre-1982 Evidence):

Before 1983, psychological stress was the widely accepted cause of peptic ulcers, with bacterial infection considered speculative. The bacterial hypothesis gained traction only after Marshall & Warren's 1984 Lancet paper, though their work began earlier.

**Final Answer:**

Before 1983, the medical consensus strongly favored **psychological stress as the primary cause of peptic ulcers**, with bacterial infection being a marginal, unproven theory.

**Key References (Pre-1982):**

1. Wolf & Wolff (1943) – Stress and acid secretion.

2. Selye (1936) – Stress physiology.

3. Friedman & Rosenman (1959) – Personality and ulcers.

4. Steer & Colin-Jones (1975) – Early bacterial observations.

5. Warren (1980-81) – Preliminary H. pylori findings (published later).

Would you like more details on any of these?

**Reply to above:**

I then ask to follow up with full references

1. Psychological Stress Hypothesis

Wolf & Wolff (1943)

- Title: Human Gastric Function: An Experimental Study of a Man and His Stomach
- Authors: Stewart Wolf, Harold G. Wolff
- Journal/Publisher: Oxford University Press (1943)
- Summary: This book detailed observations of "Tom," a man with a gastric fistula, showing that emotional stress increased stomach acid secretion.

Selye (1936)

- Title: A Syndrome Produced by Diverse Nocuous Agents
- Author: Hans Selye
- Journal: Nature (1936)
- Volume/Issue: 138, pp. 32
- DOI/Link: [Nature (1936)](https://www.nature.com/articles/138032a0)
- Summary: Introduced the "General Adaptation Syndrome," linking chronic stress to ulcers and other diseases.

Friedman & Rosenman (1959)

- Title: Association of Specific Overt Behavior Pattern with Blood and Cardiovascular Findings
- Authors: Meyer Friedman, Ray H. Rosenman
- Journal: Journal of the American Medical Association (JAMA) (1959)
- Volume/Issue: 169(12), pp. 1286-1296
- DOI/Link: [JAMA (1959)](https://jamanetwork.com/journals/jama/article-abstract/326228)
- Summary: Proposed that "Type A" (stress-prone) personalities had higher ulcer rates.

2. Bacterial Infection Hypothesis (Pre-1983 Evidence)

Steer & Colin-Jones (1975)

- Title: Mucosal Changes in Gastric Ulceration and Their Response to Carbenoxolone Sodium
- Authors: H.W. Steer, D.G. Colin-Jones
- Journal: Gut (1975)
- Volume/Issue: 16(8), pp. 590-597
- DOI/Link: [Gut (1975)](https://gut.bmj.com/content/16/8/590)
- Summary: Observed bacteria in ulcer patients but dismissed them as contaminants.

Warren (1980-1981, Preliminary Work)

- Note: Robin Warren’s early observations of H. pylori were made in 1979-1981 but were not formally published until 1983 (The Lancet).
- First Major Publication (Post-1982):
  - Warren, J.R., Marshall, B.J. (1983). Unidentified Curved Bacilli on Gastric Epithelium in Active Chronic Gastritis. The Lancet, 1(8336), 1273-1275. [DOI](https://doi.org/10.1016/S0140-6736(83)92719-8)

### Comments about above:

The LLM correctly determined that psychological stress is a more likely cause of peptic ulcer in 1982. While some references seem legitimate, these are often books that are difficult to verify without going to the library or buying them. Other references are wrong or incomplete. It is also unclear as to whether secondary sources that were published much later, like a recent review or wikipedia article, are the actual source of the “reference”.

1. Wolf & Wolff, 1943, "Human Gastric Function": This was a book, although there are wikipedia pages on the authors, as well as a movie. **Seems like a legitimate reference** linking emotional stress with gastric functions
2. Selye (1936), has nothing to do with stress or the digestive system, and is about what happens if rats are severely damaged by non-specific nocuous agents. There is nothing about gastric function or peptic ulcers here, thus this **reference is irrelevant.**
3. Friedman & Rosenman (1959): This study in JAMA finds links between psychological stress and coronary heart disease, not peptic ulcer, and is thus **irrelevant.** doi:10.1001/jama.1959.03000290012005
4. Steer & Colin-Jones (1975): this is a **legitimate study** linking ulcers to bacteria.
5. Warren, J.R., Marshall, B.J. (1983), is the landmark study, but as the LLM noted, this was in 1983, and thus **beyond the cut-off date**.

### Response from Perplexity (Same question):

Before 1983, the prevailing medical consensus attributed peptic ulcers primarily to **psychological stress** and excess stomach acid, with limited consideration of bacterial causes. Here's an evaluation of the competing hypotheses based on pre-1983 evidence:

**Psychological Stress Hypothesis**

1. **Dominant Theory**:
    From the 1930s to the 1980s, peptic ulcers were widely viewed as a **classic psychosomatic illness**. Stress, repressed emotions, and lifestyle factors (e.g., diet, smoking) were considered primary causes[3](https://mecfsskeptic.com/the-dark-psychosomatic-history-of-peptic-ulcer-part-iii/)[7](https://en.wikipedia.org/wiki/Peptic_ulcer_disease).
2. **Franz Alexander's Theory**:
    In the 1950s, psychoanalyst Franz Alexander proposed that unresolved dependency needs and repressed desires for nurturing led to excess acid production, resulting in ulcers. This theory dominated clinical practice[3](https://mecfsskeptic.com/the-dark-psychosomatic-history-of-peptic-ulcer-part-iii/).
3. **Treatment Practices**:
    Therapies focused on bed rest, milk diets, and psychotherapy, reflecting the belief that emotional factors drove the disease[3](https://mecfsskeptic.com/the-dark-psychosomatic-history-of-peptic-ulcer-part-iii/).

**Pre-1983 References**:

- A 1952 *JAMA* review asserted that psychic factors played a "prominent role" in ulcer causation[3](https://mecfsskeptic.com/the-dark-psychosomatic-history-of-peptic-ulcer-part-iii/).
- Studies in the 1970s–1980s began challenging stress's role, but the paradigm remained entrenched until H. pylori's discovery[3](https://mecfsskeptic.com/the-dark-psychosomatic-history-of-peptic-ulcer-part-iii/)[7](https://en.wikipedia.org/wiki/Peptic_ulcer_disease).

**Bacterial Infection Hypothesis**

1. **Early Observations**:
   - 19th-century scientists noted spiral-shaped bacteria in stomachs, but these findings were dismissed due to the belief that bacteria couldn't survive stomach acid[4](https://en.wikipedia.org/wiki/Helicobacter_pylori)[6](https://www.animalresearch.info/en/medical-advances/nobel-prizes/discovery-of-h-pylori-and-its-role-gastric-and-peptic-ulcer/).
   - John Lykoudis treated ulcers with antibiotics in 1958, but his work was ignored[7](https://en.wikipedia.org/wiki/Peptic_ulcer_disease).
2. **1954 Study**:
    A U.S. study failing to find bacteria in stomach biopsies solidified the acid/stress theory, sidelining bacterial hypotheses[1](https://en.wikipedia.org/wiki/Timeline_of_peptic_ulcer_disease_and_Helicobacter_pylori)[4](https://en.wikipedia.org/wiki/Helicobacter_pylori).
3. **Pre-1983 Challenges**:
   - Warren observed bacteria in gastric biopsies in 1979 but struggled to culture them[4](https://en.wikipedia.org/wiki/Helicobacter_pylori)[6](https://www.animalresearch.info/en/medical-advances/nobel-prizes/discovery-of-h-pylori-and-its-role-gastric-and-peptic-ulcer/).
   - Marshall and Warren's 1982 breakthrough (published in 1983) faced skepticism until they demonstrated H. pylori's causative role via self-experimentation[5](https://pmc.ncbi.nlm.nih.gov/articles/PMC1246068/)[6](https://www.animalresearch.info/en/medical-advances/nobel-prizes/discovery-of-h-pylori-and-its-role-gastric-and-peptic-ulcer/).

**Pre-1983 References**:

- Giulio Bizzozero (1893) and Walery Jaworski (1899) documented gastric bacteria but lacked tools to prove causation[4](https://en.wikipedia.org/wiki/Helicobacter_pylori).
- The 1954 study[1](https://en.wikipedia.org/wiki/Timeline_of_peptic_ulcer_disease_and_Helicobacter_pylori)[4](https://en.wikipedia.org/wiki/Helicobacter_pylori) and prevailing dogma suppressed bacterial theories until the 1980s.

**Conclusion**

Before 1983, **psychological stress** was the dominant explanation for peptic ulcers, supported by clinical practices and influential theories like Alexander's. While bacterial observations date back to the 19th century, they were largely dismissed until Marshall and Warren's 1982 discovery of *H. pylori*[1](https://en.wikipedia.org/wiki/Timeline_of_peptic_ulcer_disease_and_Helicobacter_pylori)[4](https://en.wikipedia.org/wiki/Helicobacter_pylori)[6](https://www.animalresearch.info/en/medical-advances/nobel-prizes/discovery-of-h-pylori-and-its-role-gastric-and-peptic-ulcer/). The stress hypothesis persisted due to a lack of conclusive evidence for infection and the medical community's resistance to paradigm shifts[3](https://mecfsskeptic.com/the-dark-psychosomatic-history-of-peptic-ulcer-part-iii/)[7](https://en.wikipedia.org/wiki/Peptic_ulcer_disease).

**Final Assessment**:
 Pre-1983, the evidence overwhelmingly favored psychological stress as the accepted cause, with bacterial infection remaining a marginalized hypothesis until later validated.

1. <https://en.wikipedia.org/wiki/Timeline_of_peptic_ulcer_disease_and_Helicobacter_pylori>
2. <https://web-archive.southampton.ac.uk/cogprints.org/677/1/ulcers.htm>
3. <https://mecfsskeptic.com/the-dark-psychosomatic-history-of-peptic-ulcer-part-iii/>
4. <https://en.wikipedia.org/wiki/Helicobacter_pylori>
5. <https://pmc.ncbi.nlm.nih.gov/articles/PMC1246068/>
6. <https://www.animalresearch.info/en/medical-advances/nobel-prizes/discovery-of-h-pylori-and-its-role-gastric-and-peptic-ulcer/>
7. <https://en.wikipedia.org/wiki/Peptic_ulcer_disease>
8. <https://www.jyi.org/2005-february/2005/2/9/delayed-gratification-why-it-took-everybody-so-long-to-acknowledge-that-bacteria-cause-ulcers>
9. <https://www.nobelprize.org/prizes/medicine/2005/press-release/>
10. <https://www.news-medical.net/health/Peptic-Ulcer-History.aspx>
11. <https://pmc.ncbi.nlm.nih.gov/articles/PMC1283743/>
12. <https://www.mountsinai.org/health-library/report/peptic-ulcers>
13. <https://pubmed.ncbi.nlm.nih.gov/25111233/>
14. <https://www.sciencedirect.com/science/article/abs/pii/S1542356514011367>
15. <https://www.sciencedirect.com/science/article/abs/pii/036192308090307X>
16. <https://www.jwatch.org/na36353/2014/11/25/psychological-stress-and-peptic-ulcer>
17. <https://www.ncbi.nlm.nih.gov/books/NBK2432/>
18. <https://pmc.ncbi.nlm.nih.gov/articles/PMC4017034/>
19. <https://blogs.the-hospitalist.org/content/stress-independently-predicts-peptic-ulcers>
20. <https://journals.lww.com/md-journal/fulltext/2017/08250/the_association_between_peptic_ulcer_diseases_and.26.aspx>
21. <https://pubmed.ncbi.nlm.nih.gov/3625586/>
22. <https://badgut.org/information-centre/a-z-digestive-topics/nobel-prize-for-h-pylori-discovery/>

### Comments on above

While overall this LLM is correct in that in 1982 and before the most likely hypothesis for peptic ulcer was psychological stress, the references are all from modern sources that would not have been available in 1982. Additionally, many sources (webpages, blogs, etc.) lack peer review.

1. Reference 1 is a wikipedia article (post 1982).
2. Reference 2 is a webpage from 1997.
3. Reference 3: The dark psychosomatic history of peptic ulcer part III, is a blog post (continued from parts I and II, posted on [July 25, 2022](https://mecfsskeptic.com/the-dark-psychosomatic-history-of-peptic-ulcer-part-iii/). While containing good information, real references require digging.
4. Reference 4 is a wikipedia article on Helicobacter pylori (post 1982).
5. Reference 5 is a 2005 article about Marshall and Warren winning the nobel prize.
6. Reference 6 is a webpage about Marshall and Warren from 2014.
7. Reference 7 is a wikipedia article about peptic ulcers.
8. Reference 8 is an article from 2005 about why it took so long to accept H. pylori as a cause of peptic ulcer.
9. Reference 9 is a press release from 2005 about Marshall and Warren winning the nobel prize.
10. Reference 10 is a news article from 2023.
11. Reference 11 is a review from 2005.
12. Reference 12 is a Mount Sinai health webpage (not dated- but obviously after 1982).
13. Reference 13 is a 2014 paper by Levenstein et al.
14. Reference 14 is the same 2014 paper, just getting there with a different url.
15. Reference 15 is a legitimate reference from 1980 questioning the impact of psychological stress on ulcers.
16. Reference 16 is a commentary on the 2014 Levenstein paper.
17. Reference 17 is a 2001 book chapter by Marshall about 100 years of H. pylori.
18. Reference 18 is a 2014 history of H. pylori by Graham
19. Reference 19 is a 2019 blog post.
20. Reference 20 is a 2017 article about peptic ulcers and stress.
21. Reference 21 is a 1987 article about behaviors and peptic ulcers.
22. Reference 22 is a webpage from the Canadian society for gastrointestinal research that talks about the winning of the Nobel prize in 2005.

### Response from GPT-5

Please evaluate the following competing hypotheses using only information from 1982 or before. Please provide references showing where you got this information. Here are the hypotheses: "Is it more likely that peptic ulcer is caused by psychological stress or by a bacterial infection?"

**ChatGPT said:**

Excellent question — and an important one to ground historically. Let’s evaluate the two competing hypotheses strictly using information that was available in or before 1982, with references to primary and review literature available up to that date.

**Competing Hypotheses (pre-1982)**

1. Peptic ulcer is caused by psychological stress.
2. Peptic ulcer is caused by a bacterial infection.

**Background: Peptic Ulcer Understanding Before 1982**

Before 1982, the dominant medical consensus was that peptic ulcers (gastric and duodenal) resulted from an imbalance between acid-pepsin secretion and mucosal defense, modulated by stress, personality, and lifestyle factors, rather than infection.

**Hypothesis 1: Psychological Stress as the Primary Cause**

Evidence and Views up to 1982

- Psychosomatic theory of ulcer disease:
   Throughout the mid-20th century, ulcers were widely regarded as psychosomatic diseases. The concept stemmed from the work of Franz Alexander (1934, 1950), who proposed that emotional conflicts, particularly involving dependency and aggression, could lead to increased gastric acid secretion and ulceration.
  - Source: Alexander, F. Psychosomatic Medicine: Its Principles and Applications. New York: Norton, 1950.
- Experimental animal models:
   Studies in rats subjected to restraint stress or cold exposure demonstrated gastric erosions or ulcers, supporting a stress mechanism.
  - Source: Wolf, S., & Wolff, H. G. Human Gastric Function: An Experimental Study of a Man and His Stomach. New York: Oxford University Press, 1943.
  - Source: Selye, H. The Stress of Life. New York: McGraw-Hill, 1956.
- Observational and clinical studies:
   Many clinicians observed that ulcer symptoms worsened with emotional stress, anxiety, or life changes (e.g., during war).
  - Source: Wolf, S. “The Psychosomatic Concept of Peptic Ulcer.” Annals of Internal Medicine 49, no. 6 (1958): 1369–1384.
  - Source: Doll, R., & Jones, F. A. “Occupational Stress and Gastric Ulcer.” Lancet 1 (1951): 129–131.
- Lack of consistent organic cause found:
   By the 1970s, it was accepted that ulcers involved acid hypersecretion and mucosal vulnerability, both of which were thought to be influenced by stress, diet, and smoking rather than microbes.
  - Source: Grossman, M. I. “Peptic Ulcer: Pathogenesis.” Annals of Internal Medicine 73 (1970): 903–914.

**Summary (Pre-1982)**

By 1982, the psychological stress hypothesis remained the dominant view among gastroenterologists, with stress considered a key precipitating factor acting through neuroendocrine and acid-secretion mechanisms.

**Hypothesis 2: Bacterial Infection as the Primary Cause**

Evidence and Views up to 1982

- Occasional observation of bacteria:
   Spiral bacteria (later Helicobacter pylori) had occasionally been reported in the stomach by pathologists since the late 19th and early 20th centuries:
  - Bizzozero (1893) observed spiral organisms in canine stomachs.
  - Krienitz (1906) noted similar bacteria in human gastric mucosa.
     However, these findings were considered incidental, as bacteria were thought unable to survive in the acidic environment of the stomach.
- Mid-20th century skepticism:
   Numerous studies in the 1940s–1970s concluded that bacteria did not cause peptic ulcers:
  - Schindler (1947): Diseases of the Stomach and Duodenum — declared that bacterial colonization of gastric mucosa did not occur in normal or ulcerated stomachs.
  - Palmer, E. D. (1954). “Investigation of the Role of Bacteria in Gastric and Duodenal Ulceration.” Gastroenterology 27: 218–222. Palmer examined hundreds of gastric biopsy specimens and found no consistent bacterial presence, concluding infection was not a factor.
- Consensus through the 1970s:
   Textbooks and reviews consistently stated that ulcers were not infectious:
  - Wyngaarden, J. B., & Smith, L. H. Jr. (eds.) Cecil Textbook of Medicine, 15th ed. Philadelphia: W. B. Saunders, 1979 — no mention of bacteria in ulcer causation.
  - Bockus, H. L. (ed.) Gastroenterology, 3rd ed. Philadelphia: W. B. Saunders, 1976 — attributes ulcer etiology to acid-pepsin imbalance, genetics, and stress.
- No cultured organism associated:
   No bacterial species had been isolated or cultured from gastric mucosa in a reproducible way before 1982. (Marshall and Warren’s first culture of Campylobacter pyloridis—later H. pylori—was reported in 1983.)

**Summary (Pre-1982)**

By 1982, there was no accepted evidence that any bacterium caused peptic ulcer disease. The stomach was regarded as sterile, and bacterial infection was considered biologically implausible.

**Comparative Evaluation (using pre-1982 knowledge)**

| Criterion | Psychological Stress Hypothesis | Bacterial Infection Hypothesis |
| --- | --- | --- |
| Empirical support (pre-1982) | Many experimental and clinical studies linking stress to ulcer formation and exacerbation. | Only rare, inconsistent histological reports; no reproducible bacterial culture or mechanism. |
| Biological plausibility (pre-1982) | Fits accepted models of neuroendocrine and acid secretion control. | Considered implausible due to gastric acidity killing bacteria. |
| Medical consensus (pre-1982) | Dominant paradigm in textbooks and practice. | Almost entirely rejected by experts. |
| Representative reviews/textbooks | Grossman 1970; Bockus 1976; Cecil Textbook 1979 | Palmer 1954 (negative findings) |

**Conclusion (as of 1982)**

Using only knowledge and evidence available up to 1982, it was far more likely, according to prevailing evidence and expert consensus, that peptic ulcer disease was caused or strongly influenced by psychological stress, not by bacterial infection.

The bacterial hypothesis was regarded as speculative and unsupported until after 1982, when Barry Marshall and Robin Warren (1983) isolated and cultured Campylobacter pyloridis (later Helicobacter pylori) from human gastric mucosa — findings that would eventually overturn the older view.

**Key Pre-1982 References**

1. Alexander, F. (1950). Psychosomatic Medicine: Its Principles and Applications. New York: Norton.
2. Wolf, S. & Wolff, H. G. (1943). Human Gastric Function. Oxford University Press.
3. Wolf, S. (1958). “The Psychosomatic Concept of Peptic Ulcer.” Annals of Internal Medicine 49: 1369–1384.
4. Selye, H. (1956). The Stress of Life. McGraw-Hill.
5. Palmer, E. D. (1954). “Investigation of the Role of Bacteria in Gastric and Duodenal Ulceration.” Gastroenterology 27: 218–222.
6. Grossman, M. I. (1970). “Peptic Ulcer: Pathogenesis.” Annals of Internal Medicine 73: 903–914.
7. Bockus, H. L. (ed.) (1976). Gastroenterology, 3rd ed. W. B. Saunders.
8. Wyngaarden, J. B., & Smith, L. H. Jr. (eds.) (1979). Cecil Textbook of Medicine, 15th ed. W. B. Saunders.
9. Schindler, R. (1947). Diseases of the Stomach and Duodenum. Lippincott.

**Summary Judgment (Pre-1982):**
 → More likely cause: Psychological stress and related physiological factors.
 → Bacterial infection: Considered highly unlikely before 1982.

### Comments on above:

Here the LLM says the “correct” answer for1982, which is that ulcers are caused by psychological stress, but many of the references cited are incorrect or non-existent.

**References:**

1. Reference 1 is **correct.**
2. Reference 2 is **correct** although the actual abstract or paper can’t be found- but there is a citation.
3. For reference 3 there is an article with this title, but it is from 1956 with a different author: KAPLAN H. The psychosomatic concept of peptic ulcer. J Nerv Ment Dis. 1956 Feb;123(2):93-111. doi: 10.1097/00005053-195602000-00001. PMID: 13367822. So this **reference is incorrect.**
4. Reference 4 by Selye, H. **is correct,** although the actual text is not online, only the citation.
5. For Reference 5, Palmer, E. D. (1954)., there is a 1954 article but it is entitled “Investigation of the gastric mucosa spirochetes of the human”, thus the **reference is incorrect.**
6. Reference 6, Grossman, M. I. (1970)., **does not exist.**
7. Reference 7, Bockus, H. L. (ed.) (1976)., is a textbook, but is not available to read online so the contents cannot be verified. But it is **a real citation.**
8. For reference 8, there is a 1979 Cecil Textbook of Medicine, 15th ed. W. B. Saunders, but the editors are Beeson, McDermott, and Wyngaarden. Also it is not online (only available for purchase), so we can’t really verify. Anyway, the **citation is incorrect.**
9. Reference 9, Schindler, R. (1947)., **does not exist.**

## Question- response to LLMs about cervical cancer being caused by HPV or HSV prior to 1986

### Cervical cancer question:

Please evaluate the following competing hypotheses using only information from 1986 or before. Please provide references showing where you got this information. Here are the hypotheses: "Is it more likely that cervical cancer is caused by Human Papillomavirus or by Herpes Simplex virus?"

### ChatGPT response

As of 1986 or earlier, there was an active debate in the scientific community regarding the possible viral causes of cervical cancer. The two primary viral candidates considered were:

1. Human Papillomavirus (HPV)
2. Herpes Simplex Virus (HSV), particularly HSV-2

To evaluate which hypothesis was better supported by the evidence available by 1986, we need to consider the epidemiological, molecular, and pathological evidence as it existed at the time.

**Evidence Supporting HPV as a Cause of Cervical Cancer (as of 1986)**

1. Detection of HPV DNA in Cervical Carcinomas

- Multiple studies had already shown the presence of HPV DNA in cervical cancer tissues, particularly types 16 and 18.
- In 1983, Harald zur Hausen and colleagues identified HPV-16 DNA in cervical carcinoma tissues (Dürst et al., 1983).
  - 📘 Durst M, Gissmann L, Ikenberg H, zur Hausen H. (1983). A papillomavirus DNA from a cervical carcinoma and its prevalence in cancer biopsy samples from different geographic regions. Proc Natl Acad Sci U S A. 80(12):3812-5.
- In 1984, they also reported HPV-18 in cervical carcinoma cell lines (Schwarz et al., 1985).
  - 📘 Schwarz E, Freese UK, Gissmann L, Mayer W, Roggenbuck B, Stremlau A, zur Hausen H. (1985). Structure and transcription of human papillomavirus sequences in cervical carcinoma cells. Nature. 314(6006):111–114.

2. Biological Plausibility

- HPVs were known to cause benign genital warts and had oncogenic potential in animal models (e.g., cottontail rabbit papillomavirus).
- HPV DNA was often found integrated into the host genome in cancerous tissues, which is consistent with a causal role in carcinogenesis.

3. Epidemiological Evidence

- Case-control studies (e.g., Meisels & Fortin, 1976) associated cytologic evidence of koilocytosis (a marker of HPV infection) with cervical intraepithelial neoplasia and carcinoma in situ.
  - 📘 Meisels A, Fortin R. (1976). Condylomatous lesions of the cervix and vagina I: Cytologic patterns. Acta Cytol. 20(6):505–509.

🧬 **Evidence Supporting HSV-2 as a Cause of Cervical Cancer (as of 1986)**

1. Epidemiological Associations

- Several epidemiological studies in the 1970s and early 1980s showed an association between HSV-2 seropositivity and cervical cancer risk.
- A prominent early study:
  - 📘 Rawls WE, Melnick JL, Glicksman JM, Kaufman RH. (1968). Herpesvirus type 2: Association with carcinoma of the cervix. Science. 161(3847):1255–1256.
- However, this evidence was correlational, and subsequent studies showed inconsistent associations.

2. Molecular Evidence Was Weaker

- Unlike HPV, HSV DNA was not consistently found in cervical cancer tissues.
- Some early claims of HSV DNA in cervical tumors (e.g., using hybridization techniques) were later challenged or not replicable with improved methods.

3. Mechanistic Uncertainty

- The idea was that HSV-2 might act as a co-factor or indirect carcinogen, possibly through chronic inflammation or DNA damage.
- However, HSV does not typically integrate into the host genome, which weakened the case for a direct oncogenic mechanism.

🔍 **Scientific Consensus as of 1986**

- By the mid-1980s, the HPV hypothesis was gaining stronger support, particularly due to molecular evidence identifying high-risk HPV types in cervical carcinomas.
- Harald zur Hausen was widely recognized for pioneering the HPV-cervical cancer link and was later awarded the Nobel Prize in 2008 for this work.

While HSV-2 was still being investigated and discussed, the weight of the evidence by 1986 favored HPV as the more likely causative agent.

✅ **Conclusion (Using Information Available by 1986)**

I**t was more likely that cervical cancer is caused by Human Papillomavirus (HPV) than by Herpes Simplex Virus (HSV)** based on molecular, pathological, and emerging epidemiological evidence available up to 1986.

📚 **Key References (All pre-1986)**

- Dürst, M., Gissmann, L., Ikenberg, H., & zur Hausen, H. (1983). A papillomavirus DNA from a cervical carcinoma and its prevalence in cancer biopsy samples from different geographic regions. Proc Natl Acad Sci U S A, 80(12), 3812–3815.
- Schwarz, E., Freese, U. K., Gissmann, L., Mayer, W., Roggenbuck, B., Stremlau, A., & zur Hausen, H. (1985). Structure and transcription of human papillomavirus sequences in cervical carcinoma cells. Nature, 314(6006), 111–114.
- Meisels, A., & Fortin, R. (1976). Condylomatous lesions of the cervix and vagina I: Cytologic patterns. Acta Cytol, 20(6), 505–509.
- Rawls, W. E., Melnick, J. L., Glicksman, J. M., & Kaufman, R. H. (1968). Herpesvirus type 2: Association with carcinoma of the cervix. Science, 161(3847), 1255–1256.

Let me know if you’d like scanned or quoted excerpts from these historical papers.

### Comments on above

The LLM correctly determines HPV to be a more likely cause of cervical cancer by 1986, and the GPT-4o model seems to rely upon legitimate papers that were before 1986 to come up with its answer and reference. However a few clues in the text show that it is also using its training information post 1986, for example when saying: “**Harald zur Hausen** was widely recognized for pioneering the HPV-cervical cancer link and was later awarded the Nobel Prize in 2008 for this work”. Thus **although all references are legitimate and pre-1986, it seems very likely that it is using some information post-1986.**

1. Dürst et al. 1983 is a **legitimate reference** pre-1986.
2. Schwarz et al. 1985 is a **legitimate reference** pre-1986.
3. Meisels & Fortin is a **legitimate reference** pre-1986.
4. Rawls et al. 1968 is a **legitimate reference** pre-1986.

### Gemini response

Based on information available in 1986 or before, it was becoming increasingly likely that Human Papillomavirus (HPV) was the primary cause of cervical cancer, with the role of Herpes Simplex Virus (HSV) being questioned and largely unsupported as the main etiological agent.

Here's an evaluation of the competing hypotheses with supporting references from 1986 or earlier:

**Evidence for Human Papillomavirus (HPV) as the Cause of Cervical Cancer (Pre-1986):**

- Hypothesis and Early Detection: Harald zur Hausen, a German virologist, first hypothesized in 1976 that HPV, rather than HSV-2, caused cervical cancer. This went against the prevailing view at the time.
  - Reference: zur Hausen, H. (1976). Condylomata acuminata and human genital cancer. Cancer Research, 36(2 Part 2), 794.
- Identification of Oncogenic HPV Types:
  - In 1983, zur Hausen and his colleagues identified HPV 16 in precursor lesions of genital cancer.
  - In 1984, they identified HPV 18 in cervical cancer tumor cells.
  - These discoveries were pivotal as HPV 16 and 18 were consistently found in a significant proportion of cervical cancer biopsies.
  - References:
    - Dürst, M., Gissmann, L., Ikenberg, H., & zur Hausen, H. (1983). A papillomavirus DNA from a cervical carcinoma and its prevalence in cancer biopsy samples from different geographic^1^ regions. Proceedings of the National Academy^2^ of Sciences, 80(12), 3812-3815.
    - Boshart, M., Gissmann, L., Ikenberg, H., Kleinheinz, A., Scheurlen, W., & zur Hausen, H. (1984). A new type of papillomavirus DNA, its presence in genital cancer biopsies and in cell lines derived from cervical cancer.^3^ The EMBO Journal, 3(5), 1151-1157.
- HPV DNA in Cancer Cells and Gene Expression: By 1985, research further revealed the genetic organization of HPV DNA in cervical cancer cells and demonstrated the active transcription of HPV in these cells. This provided stronger evidence for a causal link.
  - Reference: (This is implied by the Harvard Medical School article referencing 1985 findings, though a specific 1985 paper citation would be ideal if readily available within the given search results. The Nobel Prize press release also notes this development by 1985.) Scientists Who Linked HPV with Cervical Cancer Win 20th Annual Alpert Prize. (2008, August 29). Harvard Medical School.

**Evidence Against Herpes Simplex Virus (HSV) as the Primary Cause of Cervical Cancer (Pre-1986):**

- Inconsistent HSV-2 DNA Detection: While HSV-2 was initially considered a major suspect since the late 1960s, Harald zur Hausen's research in 1976 found inconsistent results when looking for HSV-2 DNA in cervical cancer tumor samples. Not all samples contained HSV-2 DNA, which led him to question the causal link.
  - Reference: Harald zur Hausen (1936–2023). Embryo Project Encyclopedia. (This source summarizes zur Hausen's shift in focus from HSV to HPV, citing his 1976 hypothesis).
- Mixed Results and Lack of Consistent Evidence: Even within the pre-1986 period, studies examining the role of HSV in cervical cancer yielded mixed results. While some independent epidemiological studies suggested a correlation between HSV-2 infections and an increased incidence of cervical carcinoma, others found no such correlation. Crucially, the consistency of HPV detection in cervical cancer was not matched by HSV.
  - Reference: Jones, C. M. R. (1995). Cervical Cancer: Is Herpes Simplex Virus Type II a Cofactor? Veterinary Science Papers. (While this paper is from 1995, it reviews the historical understanding and explicitly states, "Since the end of the 1960s, herpes simplex virus type 2 (HSV-2) had been considered the major cause of invasive cervical carcinoma, but a longitudinal seroepidemiologic study (1), together with inconsistent detection of HSV-2 DNA and consistent identification of human papillomavirus (HPV) DNA in cervical carcinoma^4^ (2), led to a shift in focus." This indicates that by 1986, the inconsistency of HSV-2 findings was already recognized.)

**Conclusion in 1986:**

By 1986, **the evidence strongly favored HPV as the more likely cause of cervical cancer.** While HSV-2 had been a leading candidate for years, the consistent detection of specific HPV types (especially 16 and 18) in cervical cancer lesions and the active expression of HPV genes within these cells provided compelling evidence that HPV played a direct causal role. The inconsistent findings regarding HSV-2 DNA in cervical cancer cells, combined with the emerging strong evidence for HPV, led the scientific community to shift its focus towards HPV as the primary etiologic agent.

### Comments on above:

The LLM correctly determines HPV to be the cause of cervical cancer by 1986. While some references are within the correct time frame, the LLM is still finding references beyond the cutoff date, despite this time being after the publication of the landmark paper. Interestingly, it does not mention the landmark paper (Dürst et al., 1983) at all.

1. zur Hausen, H. (1976) reference is **legitimate and relevant.**
2. Dürst, M., Gissmann, L., Ikenberg, H., & zur Hausen, H. (1983) is also a **legitimate reference** (it is the landmark paper).
3. Boshart, M., Gissmann, L., Ikenberg, H., Kleinheinz, A., Scheurlen, W., & zur Hausen, H. (1984) is a **legitimate reference**.
4. For reference 4, this information is provided: “This is implied by the Harvard Medical School article referencing 1985 findings, though a specific 1985 paper citation would be ideal if readily available within the given search results. The Nobel Prize press release also notes this development by 1985…”. Thus **this reference is incomplete and probably not from before 1986.**
5. Harald zur Hausen (1936–2023). Embryo Project Encyclopedia. This source is **beyond the cutoff date.**
6. Jones, C. M. R. (1995). This source is a historical review **beyond the cutoff date.**

### DeepSeek response

To evaluate the competing hypotheses regarding the causation of cervical cancer—Human Papillomavirus (HPV) versus Herpes Simplex Virus (HSV)—using only information available up to 1986, we can rely on the scientific literature from that time period. Here’s the assessment:

**1. Evidence Supporting HPV as a Cause of Cervical Cancer (Pre-1986)**

By the mid-1980s, accumulating epidemiological and molecular evidence strongly suggested an association between HPV and cervical cancer:

- Harald zur Hausen’s work (1976-1986): Zur Hausen and colleagues identified HPV DNA in cervical cancer tissues, particularly HPV-16 (1983) and HPV-18 (1984), suggesting a causal role ([zur Hausen et al., 1983](https://www.ncbi.nlm.nih.gov/pmc/articles/PMC384391/); [Dürst et al., 1983](https://pubmed.ncbi.nlm.nih.gov/6329740/)).

- Epidemiological studies: Case-control studies found HPV (especially HPV-16/18) more frequently in cervical cancer cases than in controls ([Boshart et al., 1984](https://pubmed.ncbi.nlm.nih.gov/6329740/)).

- Mechanistic plausibility: HPV was shown to integrate into the host genome and express oncoproteins (E6/E7) that disrupt tumor suppressor genes (p53, Rb) ([Schwarz et al., 1985](https://pubmed.ncbi.nlm.nih.gov/2999965/)).

**2. Evidence Supporting HSV as a Cause of Cervical Cancer (Pre-1986)**

Earlier (1970s–early 1980s), HSV-2 was hypothesized as a potential cause due to:

- Serological associations: Some studies reported higher HSV-2 antibodies in cervical cancer patients ([Nahmias et al., 1974](https://pubmed.ncbi.nlm.nih.gov/4374405/)).

- In vitro transformation: HSV-2 could transform cells in culture ([Frenkel et al., 1972](https://pubmed.ncbi.nlm.nih.gov/4343964/)).

However, by the mid-1980s:

- Lack of consistent viral DNA in tumors: Unlike HPV, HSV-2 DNA was rarely detected in cervical cancer biopsies ([McDougall et al., 1986](https://pubmed.ncbi.nlm.nih.gov/3007830/)).

- Weaker epidemiological support: Associations were inconsistent, and HSV was not definitively linked to oncogenesis in humans.

**Conclusion (Pre-1986 Evidence)**

By 1986, HPV was considered the more likely cause of cervical cancer due to:

1. Stronger molecular evidence (HPV DNA in tumors, oncogenic mechanisms).

2. More consistent epidemiological data compared to HSV.

3. Declining support for HSV as key studies failed to confirm its direct role.

While HSV-2 was an early suspect, the pre-1986 evidence increasingly favored HPV as the primary etiological agent.

**Key References (Pre-1986)**

1. zur Hausen, H., et al. (1983). PNAS, 80(12), 3812–3815. [HPV-16 in cervical cancer](https://www.ncbi.nlm.nih.gov/pmc/articles/PMC384391/).

2. Dürst, M., et al. (1983). Nature, 301(5896), 643–646. [HPV-18 discovery](https://pubmed.ncbi.nlm.nih.gov/6329740/).

3. McDougall, J.K., et al. (1986). J Virol, 57(1), 353–356. [Lack of HSV-2 DNA in tumors](https://pubmed.ncbi.nlm.nih.gov/3007830/).

Thus, pre-1986 data supported HPV as the more likely cause of cervical cancer.

### Comments on above:

In general the LLM is correct that pre-1986 data supported HPV as a more likely cause of cervical cancer, and sources used are pre-1986 or from 1986. However many of the details of the citations are incorrect or the papers do not exist.

1. zur Hausen, H., et al. (1983). The **link to Pubmed is incorrect** here, it goes to an error page. It is unclear if maybe it was meant to be the Dürst 1983 paper because zur Hansen is the last author on that paper, but regardless the link does not work.
2. Dürst et al.,1983 reference points to the Boshart et al.,1984 paper on Pubmed. The actual DOI link is this: <https://doi.org/10.1073/pnas.80.12.3812>. Thus **it is incorrect.**
3. Boshart et al., 1984 reference **is correct** and this is where they found HPV 18 connected to cancer.
4. Schwarz et al., 1985: the link here leads to a paper by Floren about hepatic binding of triglycerides (DOI: [10.3109/00365518509155255](https://doi.org/10.3109/00365518509155255)), which is unrelated. There is a Schwarz et al., 1985 paper about how HPV 18 integrated DNA into the genome: <https://doi.org/10.1038/314111a0>, so **the citation is incorrect.**
5. Nahmias et al., 1974: this link leads to a paper about inhibiting keratinization of epidermal cells in guinea pig by Delescluse et al. (<https://doi.org/10.1111/j.1432-0436.1974.tb00369.x>), so unrelated and the citation is wrong. There is a Nahmias 1974 paper, but it is also unrelated: <https://doi.org/10.1287/mnsc.20.9.1276>. Thus the **paper is irrelevant and citation is incorrect.**
6. Frenkel et al., 1972: link also leads us to an unrelated paper by Finkielman in 1972: <https://doi.org/10.1073/pnas.69.11.3341>. There is a Frenkel 1972 paper about finding a HSV-2 DNA fragment in cervical cancer: <https://doi.org/10.1073/pnas.69.12.3784>, but there are also many other Frenkel 1972 papers that are unrelated. Thus this is counted as being **incorrect.**
7. I could not find the McDougall paper from 1986, and the link goes to an unrelated 1986 paper: <https://doi.org/10.1254/jjp.40.95>. I think the **paper does not exist.**

### Perplexity

By 1986, emerging evidence increasingly supported human papillomavirus (HPV) as the primary cause of cervical cancer, while herpes simplex virus (HSV) was largely relegated to a potential cofactor or alternative pathway in a minority of cases. Here's the evaluation based on pre-1987 evidence:

Human Papillomavirus (HPV) Hypothesis

1. Technological Advances:
    The development of HPV DNA detection methods in the early 1980s enabled researchers to identify HPV in cervical cancer tissues[1](https://pmc.ncbi.nlm.nih.gov/articles/PMC1769629/)[3](https://en.wikipedia.org/wiki/Cervical_cancer). By 1983–1986, HPV types 16 and 18 were specifically linked to cervical cancer in multiple studies[1](https://pmc.ncbi.nlm.nih.gov/articles/PMC1769629/)[3](https://en.wikipedia.org/wiki/Cervical_cancer).
2. Epidemiological Consistency:
    Case-control studies conducted in the early 1980s demonstrated HPV DNA in 90–100% of cervical cancer cases, compared to 5–20% in controls[1](https://pmc.ncbi.nlm.nih.gov/articles/PMC1769629/)[3](https://en.wikipedia.org/wiki/Cervical_cancer). This stark contrast satisfied criteria for causality, including temporal association and biological plausibility[1](https://pmc.ncbi.nlm.nih.gov/articles/PMC1769629/).
3. Mechanistic Evidence:
    HPV’s E6 and E7 oncoproteins were shown to interfere with tumor suppressor proteins (p53 and pRb), providing a direct mechanism for carcinogenesis[1](https://pmc.ncbi.nlm.nih.gov/articles/PMC1769629/)[3](https://en.wikipedia.org/wiki/Cervical_cancer). This contrasted with HSV, which lacked analogous oncogenic mechanisms[2](https://pmc.ncbi.nlm.nih.gov/articles/PMC172875/)[4](https://pubmed.ncbi.nlm.nih.gov/8827359/).

Herpes Simplex Virus (HSV) Hypothesis

1. Early Suspicions:
    Before HPV’s discovery, HSV-2 was suspected due to its sexual transmission profile and association with cervical dysplasia in 1960s–1970s studies[2](https://pmc.ncbi.nlm.nih.gov/articles/PMC172875/)[3](https://en.wikipedia.org/wiki/Cervical_cancer). However, by the early 1980s, HSV DNA was rarely detected in cervical tumors[3](https://en.wikipedia.org/wiki/Cervical_cancer).
2. Cofactor Role:
    Some pre-1986 studies suggested HSV-2 might act as a cofactor by inducing chromosomal damage or unscheduled DNA synthesis[2](https://pmc.ncbi.nlm.nih.gov/articles/PMC172875/)[4](https://pubmed.ncbi.nlm.nih.gov/8827359/). However, these effects were not specific to cervical cancer and lacked consistent epidemiological support[2](https://pmc.ncbi.nlm.nih.gov/articles/PMC172875/)[4](https://pubmed.ncbi.nlm.nih.gov/8827359/).
3. Declining Support:
    By 1986, HSV’s role was limited to 5–10% of HPV-negative cervical cancers, with no conclusive evidence as a primary cause[4](https://pubmed.ncbi.nlm.nih.gov/8827359/). Most risk factors (e.g., multiple sexual partners) aligned more strongly with HPV’s transmission[1](https://pmc.ncbi.nlm.nih.gov/articles/PMC1769629/)[3](https://en.wikipedia.org/wiki/Cervical_cancer).

Key Pre-1987 References

- HPV:
  - HPV DNA identified in cervical cancer tissues (early 1980s)[1](https://pmc.ncbi.nlm.nih.gov/articles/PMC1769629/)[3](https://en.wikipedia.org/wiki/Cervical_cancer).
  - Mechanistic studies linking E6/E7 to oncogenesis (mid-1980s)[1](https://pmc.ncbi.nlm.nih.gov/articles/PMC1769629/)[3](https://en.wikipedia.org/wiki/Cervical_cancer).
- HSV:
  - Limited detection in tumors and inconsistent epidemiological links (pre-1986)[2](https://pmc.ncbi.nlm.nih.gov/articles/PMC172875/)[4](https://pubmed.ncbi.nlm.nih.gov/8827359/).
  - Proposed cofactor role via chromosomal damage (1970s–1980s)[2](https://pmc.ncbi.nlm.nih.gov/articles/PMC172875/)[4](https://pubmed.ncbi.nlm.nih.gov/8827359/).

Conclusion

By 1986, HPV had surpassed HSV as the leading etiological agent for cervical cancer due to stronger molecular and epidemiological evidence. HSV remained a speculative cofactor in rare HPV-negative cases, but its primary causal role was effectively disproven[1](https://pmc.ncbi.nlm.nih.gov/articles/PMC1769629/)[3](https://en.wikipedia.org/wiki/Cervical_cancer)[4](https://pubmed.ncbi.nlm.nih.gov/8827359/).

1. <https://pmc.ncbi.nlm.nih.gov/articles/PMC1769629/>
2. <https://pmc.ncbi.nlm.nih.gov/articles/PMC172875/>
3. <https://en.wikipedia.org/wiki/Cervical_cancer>
4. <https://pubmed.ncbi.nlm.nih.gov/8827359/>
5. <https://academic.oup.com/jnci/article/103/5/368/905734>
6. <https://pmc.ncbi.nlm.nih.gov/articles/PMC145302/>
7. <https://www.sciencedirect.com/science/article/pii/S0042682208007721>
8. <https://www.cdc.gov/pinkbook/hcp/table-of-contents/chapter-11-human-papillomavirus.html>
9. <https://www.sciencedirect.com/science/article/pii/0738081X84900695>
10. <https://pmc.ncbi.nlm.nih.gov/articles/PMC6346234/>
11. <https://www.sciencedirect.com/science/article/pii/S0959804917300278>
12. <https://pmc.ncbi.nlm.nih.gov/articles/PMC10378257/>
13. <https://www.nature.com/articles/nrc798>
14. <https://www.cancer.gov/research/progress/discovery/hpv-vaccines>
15. <https://www.guttmacher.org/journals/ipsrh/2003/06/cervical-cancer-risk-rises-if-women-hpv-also-have-herpes-infection>
16. <https://www.nature.com/articles/6600918>

### Comments about above

Perplexity gives the correct answer of HPV being more likely, but still seems to gather evidence from more recent years past the cut-off date.

1. Reference 1 is a 2002 paper, **so beyond the cut-off date.**
2. Reference 2 is a 1995 paper, **so beyond the cut-off date.**
3. Reference 3 is a Wikipedia article on cervical cancer (**thus beyond the cutoff date**).
4. Reference 4 is a 1996 article, **so beyond the cut-off date.**
5. Reference 5 is a 2011 article, **so beyond the cut-off date.**
6. Reference 6 is a 2003 article, **so beyond the cut-off date.**
7. Reference 7 is a 2009 article, **so beyond the cut-off date.**
8. Reference 8 is a 2024 article from the CDC, **so beyond the cut-off date.**
9. Reference 9 is a **legitimate article** from 1984 that discusses HSV-2 and cervical cancer.
10. Reference 10 is a 2019 article, **so beyond the cut-off date.**
11. Reference 11 is a 2017 article, **so beyond the cut-off date.**
12. Reference 12 is a 2023 article, **so beyond the cut-off date.**
13. Reference 13 is a 2002 article, **so beyond the cut-off date.**
14. Reference 14 is a NIH website about HPV vaccines, **so beyond the cut-off date.**
15. Reference 15 is a 2003 article, **so beyond the cut-off date.**
16. Reference 16 is a 2003 article, **so beyond the cut-off date.**

### ChatGPT-5 response

Here’s an evaluation of the two competing hypotheses — “cervical cancer is caused by Human Papillomavirus (HPV)” vs. “cervical cancer is caused by Herpes Simplex Virus (HSV)” — using only evidence available up to 1986 (or very close to it). I will summarise the evidence for each, then assess which hypothesis is more strongly supported by the data as of that time.

**Evidence for HPV as the cause of cervical cancer**

Supporting evidence up to ~1986:

1. The hypothesis that HPV might cause cervical cancer was first proposed by Harald zur Hausen in the 1970s when he reasoned that genital wart viruses (papillomaviruses) might play a role in cervical cancer, rather than HSV. [PMC+3Cancer Prevention+3Embryo Project Encyclopedia+3](https://prevention.cancer.gov/about-dcp/history-and-timeline/commemoration-50th/harald-zur-hausen?utm_source=chatgpt.com)
2. In 1983, zur Hausen’s team (and collaborators) detected HPV type 16 DNA in cervical carcinoma biopsies (and in some cell lines) from different geographic regions. [PMC+3NobelPrize.org+3Embryo Project Encyclopedia+3](https://www.nobelprize.org/uploads/2018/06/advanced-medicineprize2008.pdf?utm_source=chatgpt.com)
3. In 1984-85, they identified HPV type 18 in a proportion of cervical cancer samples. [Embryo Project Encyclopedia+1](https://embryo.asu.edu/pages/harald-zur-hausens-experiments-human-papillomavirus-causing-cervical-cancer-1976-1987?utm_source=chatgpt.com)
4. The magnitude of association: Some review sources note that by the 1980s and early 1990s, the link between HPV and cervical squamous cell carcinoma was seen as stronger than the link between smoking and lung cancer. [PMC+1](https://pmc.ncbi.nlm.nih.gov/articles/PMC145302/?utm_source=chatgpt.com)
5. The logic of causality: One of the foundational papers (the Nobel‐prize document) notes that earlier prevailing hypotheses (e.g., HSV) were supplanted by the HPV hypothesis once HPV DNA was found integrated into tumour host genomes. [NobelPrize.org](https://www.nobelprize.org/uploads/2018/06/advanced-medicineprize2008.pdf?utm_source=chatgpt.com)

**Caveats (as of ~1986):**

- Although HPV DNA was found in many cervical cancers, it was not yet universally accepted that HPV alone was the cause (by 1986).
- The natural history (i.e., persistence of HPV infection → dysplasia → cancer) was still being unravelled (much more so after 1986).
- Some early epidemiologic studies still looked at multiple risk factors (sexual behaviour, multiple partners, early sexual activity) and didn’t exclusively point at HPV.

**Conclusion for HPV hypothesis (as of ~1986):**
 By the mid-1980s the HPV hypothesis had strong mechanistic, molecular and epidemiological support (HPV DNA in cancers, identification of high-risk types, plausible biology). It was clearly the leading viral hypothesis for cervical cancer by then.

**Evidence for HSV (especially HSV-2) as the cause of cervical cancer**

Supporting evidence up to ~1986:

1. HSV-2 had long been suspected (since the 1960s/70s) as a causative agent because cervical cancer had features reminiscent of a sexually transmitted disease (multiple partners, early onset of sexual activity) and HSV was a known genital herpes virus. [PMC+2Wiley Online Library+2](https://pmc.ncbi.nlm.nih.gov/articles/PMC7089001/?utm_source=chatgpt.com)
2. Some early laboratory studies (for example, Dreesman et al., 1980) reported that HSV2 antigen (or HSV‐associated antigens) could be found in some cervical dysplasia or carcinoma tissues: e.g., “Expression of herpesvirus-induced antigens in human cervical cancer” (1980) reported HSV2-specific DNA‐binding antigen expression in ~38% of dysplasia/carcinoma tissues. [Nature](https://www.nature.com/articles/283591a0?utm_source=chatgpt.com)
3. Some sero‐epidemiological studies found higher rates of HSV2 antibodies in women with cervical neoplasia compared with controls (although these studies were heterogeneous and many confounded by sexual behaviour). Some risk‐factor studies around 1986 still included HSV2 antibody status (e.g., Rawls et al. 1986). [Wiley Online Library+1](https://onlinelibrary.wiley.com/doi/abs/10.1002/ijc.2910370411?utm_source=chatgpt.com)

**Evidence against HSV hypothesis (as of ~1986):**

- The mechanistic evidence was weak: while HSV2 could infect epithelial cells and perhaps push them toward unscheduled DNA synthesis, the evidence of genuine transformation, persistent viral gene expression in tumour cells, integration, etc., was far less convincing than for HPV. (For example, the HSV2 antigen results were modest and inconsistent).
- Later (post-1986) prospective serologic studies (e.g., in Finland) found no increased risk of cervical neoplasia with prior HSV2 infection. For example, a study covering samples from 1968-72 with follow-up 12 years (to 1981) found that HSV2 antibodies did not predict cervical neoplasia (RR ~0.5, 95% CI 0.2-1.6) — while this is slightly after 1986 (published 1992), it’s still relevant to the assessment of this hypothesis, and the trend of results was anti‐causal. [SpringerLink+1](https://link.springer.com/article/10.1007/BF00146886?utm_source=chatgpt.com)
- The consensus (by late 1980s) was shifting: as the HPV evidence grew, the HSV hypothesis was increasingly seen as at best a possible cofactor rather than a direct cause.

**Conclusion for HSV hypothesis (as of ~1986):**
 By 1986 the HSV2 hypothesis had some suggestive epidemiologic and laboratory evidence, but the evidence was far weaker, inconsistent, and inconclusive compared with HPV. There was no consensus that HSV2 was the causal agent of cervical cancer; many experts already considered it more plausible as a cofactor if anything.

**Comparative evaluation & verdict**

Given the evidence up to 1986 (or very near that time), one can compare the two hypotheses:

- The HPV hypothesis had: direct detection of viral DNA in tumour tissue, identification of specific high-risk HPV types, a plausible biological mechanism (viral oncoproteins, integration), and a strong epidemiologic pattern consistent with sexual transmission.
- The HSV hypothesis had: some epidemiologic association and some antigen detection, but lacked consistent molecular evidence (e.g., viral integration into tumour genome, strong prospective epidemiology) and was confounded by sexual behaviour and other risk factors.

Therefore, the hypothesis that cervical cancer is caused by HPV is much more likely (given the evidence up to ~1986) than the hypothesis that HSV is the causal agent. In scientific terms, HPV meets, or nearly meets, many of the criteria for causality (strong association, plausibility, consistency, detection in tumours) whereas HSV does not.

If I were to express this probabilistically: based on the pre-1986 evidence, one would assign a high likelihood to HPV (say > 80%) and a much lower likelihood to HSV as the primary cause (perhaps < 20%), with the caveat that HSV could still act as a cofactor in some cases but not as the main causal agent.

**Final summary**

- The evidence strongly favoured HPV (especially types 16, 18) as the main causal virus for cervical cancer by the mid-1980s.
- The HSV (HSV-2) hypothesis had some early support but lacked the robust molecular, consistent epidemiological and mechanistic evidence needed to claim causality.
- Thus, the more plausible hypothesis is**: cervical cancer is caused by HPV, and not by HSV.**

If you like, I can look up all available studies up to 1986 side-by-side (serology, molecular detection, cohort studies) and tabulate the strength of evidence for each hypothesis. Would you like me to do that?

You said:

Can you list out your sources?

ChatGPT said:

Yes — here’s a list of primary and secondary sources published in or before 1986 (plus a few very near that date) that support the evaluation above. I’ve grouped them by topic (HPV or HSV hypothesis).

All of these are real, citable publications from the time period in question or shortly after (summarizing pre-1986 research).

🧬 **Evidence for HPV and cervical cancer (≤1986)**

1. zur Hausen H. (1976).
 “Condylomata acuminata and human genital cancer.” Cancer Research 36(2 pt 2): 794–798.

- Proposed that genital warts (caused by papillomaviruses) could be linked to cervical cancer, challenging the then-dominant HSV hypothesis.
   ➡️ First clear articulation of the HPV hypothesis.

2. Gissmann, L., Boshart, M., & zur Hausen, H. (1983).
 “Presence of human papillomavirus DNA in genital tumors.” Proceedings of the National Academy of Sciences USA 80(2): 560–563.

- Isolated HPV 16 DNA from cervical carcinoma biopsies.
   ➡️ First direct evidence of HPV DNA in cervical cancers.

3. Boshart, M., Gissmann, L., Ikenberg, H., Kleinheinz, A., Scheurlen, W., & zur Hausen, H. (1984).
 “A new type of papillomavirus DNA, its presence in genital cancer biopsies and in cell lines derived from cervical cancer.” EMBO Journal 3(5): 1151–1157.

- Identified HPV 18 DNA in cervical cancer tissues and cell lines.
   ➡️ Strengthened molecular link between HPV and cancer.

4. Syrjänen, K. J. (1983).
 “Histological changes identical to those of condylomatous lesions found in cervical intraepithelial neoplasia: suggested role of papillomavirus infection in cervical carcinogenesis.” Archives of Gynecology 233(4): 241–254.

- Showed that early cervical precancerous lesions have papillomavirus-like histology.
   ➡️ Provided morphological evidence connecting HPV infection and cervical precancer.

5. Durst, M., Gissmann, L., Ikenberg, H., & zur Hausen, H. (1983).
 “A papillomavirus DNA from a cervical carcinoma and its prevalence in cancer biopsy samples from different geographic regions.” PNAS USA 80(12): 3812–3815.

- Demonstrated global presence of HPV 16 DNA in cervical carcinomas.
   ➡️ Evidence of causality and ubiquity.

6. zur Hausen, H. (1986).
 “Papillomaviruses in anogenital cancer as a model to understand the role of viruses in human cancers.” Cancer Research 46(10): 4698–4706.

- Review consolidating 1980s evidence for HPV causality.
   ➡️ By 1986, HPV had become the leading viral explanation.

🦠 **Evidence for HSV and cervical cancer (≤1986)**

1. Nahmias, A. J., Naib, Z. M., Josey, W. E., et al. (1970).
 “Antibodies to herpesvirus hominis types 1 and 2 in humans with cervical cancer.” Science 170(3954): 1238–1240.

- Early report of elevated HSV-2 antibodies in women with cervical cancer.
   ➡️ One of the first studies linking HSV-2 serology to cervical cancer.

2. Rawls, W. E., Tompkins, W. A. F., Melnick, J. L. (1973).
 “Herpesvirus type 2: association with carcinoma of the cervix.” Science 181(4100): 191–193.

- Reported correlation between HSV-2 antibodies and cervical carcinoma.
   ➡️ Foundational evidence for HSV hypothesis (later shown to be confounded).

3. Dreesman, G. R., et al. (1980).
 “Expression of herpesvirus-induced antigens in human cervical cancer.” Nature 283(5748): 591–593.

- Claimed detection of HSV-2 antigens in 38% of cervical cancer tissues.
   ➡️ Suggestive but not reproducible evidence for viral involvement.

4. Sever, J. L., Madden, D. L., Kuff, E. L., et al. (1982).
 “Viral etiology of cervical carcinoma.” Obstetrics & Gynecology 59(5 Suppl): 51S–57S.

- Review assessing HSV-2 and other viruses as possible causes.
   ➡️ Acknowledged HSV-2 correlation but called evidence inconclusive.

5. Rawls, W. E., et al. (1986).
 “Prospective study of risk factors for cervical intraepithelial neoplasia: II. Serological evidence.” International Journal of Cancer 37(4): 641–645.

- Studied HSV-2 antibodies vs. cervical neoplasia risk.
   ➡️ Reported only weak or no independent effect once sexual behaviour was controlled.

📚 Reviews & general context (≤1986)

1. zur Hausen, H. (1982).
 “Human genital cancer: synergism between two virus infections or two viral genes?” Lancet 2(8302): 1370–1372.
 ➡️ Proposed HPV as the causal virus, HSV as a possible cofactor.

2. McDougall, J. K., et al. (1983).
 “Viruses and cervical cancer.” Cancer 51(5): 873–879.
 ➡️ Reviewed evidence for HSV vs. HPV; concluded HSV hypothesis was weakening.

3. zur Hausen, H. (1984).
 “Papillomaviruses and cancer: from basic studies to clinical application.” Nature 311(5983): 271–275.
 ➡️ Summarized experimental evidence that HPV, not HSV, integrates into host genomes and drives malignant transformation.

🔍 **Summary of references and their implications**

| Year | Key Reference | Virus | Evidence Type | Conclusion |
| --- | --- | --- | --- | --- |
| 1970 | Nahmias et al., Science | HSV-2 | Serology | Association (not causal) |
| 1973 | Rawls et al., Science | HSV-2 | Serology | Weak link |
| 1980 | Dreesman et al., Nature | HSV-2 | Antigen detection | Inconsistent |
| 1983 | Durst et al., PNAS | HPV | DNA detection | Strong causal evidence |
| 1984 | Boshart et al., EMBO J. | HPV | Molecular | Strong causal evidence |
| 1986 | zur Hausen, Cancer Res. | HPV | Review | HPV favored as main cause |

✅ **Overall evaluation (based only on ≤1986 evidence):**

- HPV: Strong molecular, histological, and epidemiological evidence.
- HSV-2: Mostly epidemiologic associations, likely confounded; no consistent viral DNA in tumors.

➡️ By 1986, it was **far more likely that cervical cancer was caused by Human Papillomavirus than by Herpes Simplex Virus.**

### Comments on above:

Here the LLM correctly identifies the HPV hypothesis to be more likely by 1986. However, it did not list the sources right away, and instead had a little button to click the sources (see **Sources** below). These first sources were almost exclusively post-1986 (31 out of 36 links), consisting of reviews or websites that talked about the history of cervical cancer, essentially summarizing what happened with the HSV-2, and then HPV hypotheses. They are listed below as “Sources”. Then when I asked it to list its sources, it gave me the “References”, which claim to be from 1986 or before. So it is obvious that it used more recent information to come up with its answers, but then back-cited old sources, which were probably cited in the newer information that it used. In the References section, many of these citations are incorrect or do not exist. See **References** below.

**Sources:**

- [Cancer Prevention](https://prevention.cancer.gov/about-dcp/history-and-timeline/commemoration-50th/harald-zur-hausen?utm_source=chatgpt.com)
- [Harald zur Hausen, MD, D.Sc. - Division of Cancer Prevention](https://prevention.cancer.gov/about-dcp/history-and-timeline/commemoration-50th/harald-zur-hausen?utm_source=chatgpt.com)
- [In 1976, he published his theory that it was HPV that caused cervical cancer, not HSV. Subsequent experiments proved his theory correct, as he identified HPV-16 ...](https://prevention.cancer.gov/about-dcp/history-and-timeline/commemoration-50th/harald-zur-hausen?utm_source=chatgpt.com)
- [NobelPrize.org](https://www.nobelprize.org/uploads/2018/06/advanced-medicineprize2008.pdf?utm_source=chatgpt.com)
- [[PDF] 1 The discoveries of human papilloma viruses that cause cervical ...](https://www.nobelprize.org/uploads/2018/06/advanced-medicineprize2008.pdf?utm_source=chatgpt.com)
- [The awarded discovery of human papilloma virus causing cervical cancer. In 1974 zur Hausen published his first report at attempting to find HPV DNA in ...](https://www.nobelprize.org/uploads/2018/06/advanced-medicineprize2008.pdf?utm_source=chatgpt.com)
- [Embryo Project Encyclopedia](https://embryo.asu.edu/pages/harald-zur-hausens-experiments-human-papillomavirus-causing-cervical-cancer-1976-1987?utm_source=chatgpt.com)
- [Harald zur Hausen's Experiments on Human Papillomavirus ...](https://embryo.asu.edu/pages/harald-zur-hausens-experiments-human-papillomavirus-causing-cervical-cancer-1976-1987?utm_source=chatgpt.com)
- [March 8, 2017 — Zur Hausen and his research team concluded that HPV 6 and 11 caused genital warts, while HPV 16 and 18 caused cervical cancer.](https://embryo.asu.edu/pages/harald-zur-hausens-experiments-human-papillomavirus-causing-cervical-cancer-1976-1987?utm_source=chatgpt.com)
- [PMC](https://pmc.ncbi.nlm.nih.gov/articles/PMC145302/?utm_source=chatgpt.com)
- [Human Papillomavirus and Cervical Cancer - PMC](https://pmc.ncbi.nlm.nih.gov/articles/PMC145302/?utm_source=chatgpt.com)
- [The link between genital HPV infections and cervical cancer was first demonstrated in the early 1980s by Harold zur Hausen, a German virologist. Since then, ...](https://pmc.ncbi.nlm.nih.gov/articles/PMC145302/?utm_source=chatgpt.com)
- [PMC](https://pmc.ncbi.nlm.nih.gov/articles/PMC7089001/?utm_source=chatgpt.com)
- [The HPV Vaccine Story - PMC](https://pmc.ncbi.nlm.nih.gov/articles/PMC7089001/?utm_source=chatgpt.com)
- [Research on the cause of cervical cancer first appeared in the scientific literature in the 1840s. As a common cancer, it was contrasted with breast cancer ...](https://pmc.ncbi.nlm.nih.gov/articles/PMC7089001/?utm_source=chatgpt.com)
- [Nature](https://www.nature.com/articles/283591a0?utm_source=chatgpt.com)
- [Expression of herpesvirus-induced antigens in human ...](https://www.nature.com/articles/283591a0?utm_source=chatgpt.com)
- [by GR Dreesman · 1980 · Cited by 121 — Herpes simplex virus type 2 (HSV2) was first associated with human cervical cancer by seroepidemiologic studies over 10 years ago, ...](https://www.nature.com/articles/283591a0?utm_source=chatgpt.com)
- [Wiley Online Library](https://onlinelibrary.wiley.com/doi/abs/10.1002/ijc.2910370411?utm_source=chatgpt.com)
- [Comparison of risk factors for cervical cancer in different ...](https://onlinelibrary.wiley.com/doi/abs/10.1002/ijc.2910370411?utm_source=chatgpt.com)
- [by WE Rawls · 1986 · Cited by 31 — Risk factors of cervical cancer include early age at first marriage, multiple marriages and antibodies to herpes simplex virus type 2 (HSV-2).](https://onlinelibrary.wiley.com/doi/abs/10.1002/ijc.2910370411?utm_source=chatgpt.com)
- [SpringerLink](https://link.springer.com/article/10.1007/BF00146886?utm_source=chatgpt.com)
- [Herpes simplex virus type 2 infection and cervical cancer](https://link.springer.com/article/10.1007/BF00146886?utm_source=chatgpt.com)
- [by M Lehtinen · 1992 · Cited by 30 — This study was initiated to investigate the role of past herpes simplex virus type 2 (HSV-2) infection, as determined by serum antibody analysis, ...](https://link.springer.com/article/10.1007/BF00146886?utm_source=chatgpt.com)

More

- [cdc.gov](https://www.cdc.gov/pinkbook/hcp/table-of-contents/chapter-11-human-papillomavirus.html?utm_source=chatgpt.com)
- [Chapter 11: Human Papillomavirus | Pink Book - CDC](https://www.cdc.gov/pinkbook/hcp/table-of-contents/chapter-11-human-papillomavirus.html?utm_source=chatgpt.com)
- [April 22, 2024 — In the early 1980s, cervical cancer cells were shown to contain HPV DNA. Epidemiologic studies demonstrating a consistent association between ...](https://www.cdc.gov/pinkbook/hcp/table-of-contents/chapter-11-human-papillomavirus.html?utm_source=chatgpt.com)
- [who.int](https://www.who.int/news-room/fact-sheets/detail/cervical-cancer?utm_source=chatgpt.com)
- [Cervical cancer - World Health Organization (WHO)](https://www.who.int/news-room/fact-sheets/detail/cervical-cancer?utm_source=chatgpt.com)
- [March 4, 2024 — Cervical cancer is the fourth most common cancer in women globally with around 660 000 new cases and around 350 000 deaths in 2022.](https://www.who.int/news-room/fact-sheets/detail/cervical-cancer?utm_source=chatgpt.com)
- [getteal.com](https://www.getteal.com/post/a-brief-history-of-cervical-cancer-screening?utm_source=chatgpt.com)
- [A Brief History of Cervical Cancer Screening - Teal Health](https://www.getteal.com/post/a-brief-history-of-cervical-cancer-screening?utm_source=chatgpt.com)
- [January 7, 2024 — The 1980s brought on the discovery that human papillomavirus (HPV) causes more than 90 percent of cervical cancers. This evidence led to the ...](https://www.getteal.com/post/a-brief-history-of-cervical-cancer-screening?utm_source=chatgpt.com)
- [genefirst.com](https://www.genefirst.com/news-full/the-story-of-hpv?utm_source=chatgpt.com)
- [the-story-of-hpv - GeneFirst](https://www.genefirst.com/news-full/the-story-of-hpv?utm_source=chatgpt.com)
- [December 18, 2022 — However, it was not until the late 1990s that researchers identified HPV as a leading cause of cervical cancer. Since then, we have made ...](https://www.genefirst.com/news-full/the-story-of-hpv?utm_source=chatgpt.com)
- [cancerresearchuk.org](https://news.cancerresearchuk.org/2022/11/21/into-the-archives-the-story-of-hpv-and-cervical-cancer/?utm_source=chatgpt.com)
- [Into the archives: the story of HPV and cervical cancer](https://news.cancerresearchuk.org/2022/11/21/into-the-archives-the-story-of-hpv-and-cervical-cancer/?utm_source=chatgpt.com)
- [November 20, 2022 — In the early 1980s, zur Hausen and his team continued their search and discovered HPV-16, which their small studies detected in about half of ...](https://news.cancerresearchuk.org/2022/11/21/into-the-archives-the-story-of-hpv-and-cervical-cancer/?utm_source=chatgpt.com)
- [umn.edu](https://cancer.umn.edu/mncctn/news/cervical-cancer-awareness-month-history-first-cancer-screening-test?utm_source=chatgpt.com)
- [Cervical Cancer Awareness Month: History of the First Cancer ...](https://cancer.umn.edu/mncctn/news/cervical-cancer-awareness-month-history-first-cancer-screening-test?utm_source=chatgpt.com)
- [January 2, 2022 — HPV causes about 90 percent of cervical cancers, and it is preventable through HPV vaccination. In 2021, it is estimated that about 14,000 new ...](https://cancer.umn.edu/mncctn/news/cervical-cancer-awareness-month-history-first-cancer-screening-test?utm_source=chatgpt.com)
- [mlo-online.com](https://www.mlo-online.com/home/article/21115348/a-history-of-diagnosing-hpv?utm_source=chatgpt.com)
- [A history of diagnosing HPV | Medical Laboratory Observer](https://www.mlo-online.com/home/article/21115348/a-history-of-diagnosing-hpv?utm_source=chatgpt.com)
- [November 24, 2019 — The link between HPV and cervical cancer was discovered in the 1980s by Harald zur Hausen—a discovery that led to the 2008 Nobel Prize for ...](https://www.mlo-online.com/home/article/21115348/a-history-of-diagnosing-hpv?utm_source=chatgpt.com)
- [nih.gov](https://pmc.ncbi.nlm.nih.gov/articles/PMC5691619/?utm_source=chatgpt.com)
- [Natural History of HPV Infection across the Lifespan: Role of Viral ...](https://pmc.ncbi.nlm.nih.gov/articles/PMC5691619/?utm_source=chatgpt.com)
- [September 20, 2017 — A minority of HPV infections are persistently detected beyond 12 months, increasing the risk of carcinogenic progression to cervical pre-cancer ...](https://pmc.ncbi.nlm.nih.gov/articles/PMC5691619/?utm_source=chatgpt.com)
- [cancer.gov](https://www.cancer.gov/research/progress/discovery/hpv-vaccines?utm_source=chatgpt.com)
- [Preventing Cervical Cancer with HPV Vaccines - NCI](https://www.cancer.gov/research/progress/discovery/hpv-vaccines?utm_source=chatgpt.com)
- [December 1, 2016 — In the 1980s, researchers at the German Cancer Research Center found types of HPV in many cervical tumors. HPV's role in cervical cancer seemed ...](https://www.cancer.gov/research/progress/discovery/hpv-vaccines?utm_source=chatgpt.com)
- [harvard.edu](https://hms.harvard.edu/news/scientists-who-linked-hpv-cervical-cancer-win-20th-annual-alpert-prize?utm_source=chatgpt.com)
- [Scientists Who Linked HPV with Cervical Cancer Win 20th Annual ...](https://hms.harvard.edu/news/scientists-who-linked-hpv-cervical-cancer-win-20th-annual-alpert-prize?utm_source=chatgpt.com)
- [August 28, 2008 — In 1983, the scientists identified HPV 16 in precursor lesions of genital cancer, and in 1985, they revealed the genetic organization and ...](https://hms.harvard.edu/news/scientists-who-linked-hpv-cervical-cancer-win-20th-annual-alpert-prize?utm_source=chatgpt.com)
- [sciencedirect.com](https://www.sciencedirect.com/science/article/pii/S0753332200886424?utm_source=chatgpt.com)
- [The cause of invasive cervical cancer could be multifactorial](https://www.sciencedirect.com/science/article/pii/S0753332200886424?utm_source=chatgpt.com)
- [by H Haverkos · 2000 · Cited by 120 — Epidemiologic and clinical data suggest that human papillomaviruses, especially HPV-16 and HPV-18, play the major role in the etiology of cervical cancer.](https://www.sciencedirect.com/science/article/pii/S0753332200886424?utm_source=chatgpt.com)
- [sciencedirect.com](https://www.sciencedirect.com/science/article/pii/0738081X84900695?utm_source=chatgpt.com)
- [Herpes simplex virus type 2 and cervical cancer](https://www.sciencedirect.com/science/article/pii/0738081X84900695?utm_source=chatgpt.com)
- [by L Aurelian · 1984 · Cited by 15 — Dreesman, J Burck, E Ada, et al. Expression of herpesvirus induced antigens in human cervical cancer. Nature, 283 (1980), pp. 591-593.](https://www.sciencedirect.com/science/article/pii/0738081X84900695?utm_source=chatgpt.com)
- [nih.gov](https://pmc.ncbi.nlm.nih.gov/articles/PMC80931/?utm_source=chatgpt.com)
- [Cervical cancer: epidemiology, prevention and the role of human ...](https://pmc.ncbi.nlm.nih.gov/articles/PMC80931/?utm_source=chatgpt.com)
- [Human papillomavirus (HPV) infection is now recognized as the main cause of cervical cancer, the role of coexisting factors is better understood.](https://pmc.ncbi.nlm.nih.gov/articles/PMC80931/?utm_source=chatgpt.com)
- [nih.gov](https://pmc.ncbi.nlm.nih.gov/articles/PMC4081157/?utm_source=chatgpt.com)
- [The paediatric story of human papillomavirus (Review) - PMC](https://pmc.ncbi.nlm.nih.gov/articles/PMC4081157/?utm_source=chatgpt.com)
- [It was not until the 1970s, that a role of HPV in cervical cancer was postulated for the first time by Professor Harald zur Hausen, the 'Father of HPV Virology' ...](https://pmc.ncbi.nlm.nih.gov/articles/PMC4081157/?utm_source=chatgpt.com)
- [nih.gov](https://pmc.ncbi.nlm.nih.gov/articles/PMC4515308/?utm_source=chatgpt.com)
- [Evolution of cervical cancer screening and prevention in United ...](https://pmc.ncbi.nlm.nih.gov/articles/PMC4515308/?utm_source=chatgpt.com)
- [Although the first HPV test was FDA-approved in 1988, the HC2 test was only FDA-approved in 1999 for follow-up of ASC-US cytology to identify women who may ...](https://pmc.ncbi.nlm.nih.gov/articles/PMC4515308/?utm_source=chatgpt.com)
- [nih.gov](https://pmc.ncbi.nlm.nih.gov/articles/PMC10378257/?utm_source=chatgpt.com)
- [Herpes Simplex Virus, Human Papillomavirus, and ...](https://pmc.ncbi.nlm.nih.gov/articles/PMC10378257/?utm_source=chatgpt.com)
- [by DG Sausen · 2023 · Cited by 43 — This review provides an overview of HSV and HPV infections and then delves into the possible links between HPV, HSV, and cervical cancer.](https://pmc.ncbi.nlm.nih.gov/articles/PMC10378257/?utm_source=chatgpt.com)
- [nih.gov](https://pmc.ncbi.nlm.nih.gov/articles/PMC172875/?utm_source=chatgpt.com)
- [Cervical cancer: is herpes simplex virus type II a cofactor?](https://pmc.ncbi.nlm.nih.gov/articles/PMC172875/?utm_source=chatgpt.com)
- [by C Jones · 1995 · Cited by 84 — Several independent studies suggest that HSV-2 infections correlate with a higher than normal incidence of cervical cancer.](https://pmc.ncbi.nlm.nih.gov/articles/PMC172875/?utm_source=chatgpt.com)
- [nih.gov](https://pubmed.ncbi.nlm.nih.gov/8665469/?utm_source=chatgpt.com)
- [Cervical cancer: is herpes simplex virus type II a cofactor?](https://pubmed.ncbi.nlm.nih.gov/8665469/?utm_source=chatgpt.com)
- [by C Jones · 1995 · Cited by 84 — Several independent studies suggest that HSV-2 infections correlate with a higher than normal incidence of cervical cancer.](https://pubmed.ncbi.nlm.nih.gov/8665469/?utm_source=chatgpt.com)
- [gorgas.gob.pa](https://www.gorgas.gob.pa/wp-content/uploads/external/BiblioGorgas/pdf/An%20analysis%20of%20the%20association%20between%20herpes%20simplex%20virus%20type%202%20antibodies%20and%20cervical%20cancer.%20William%20Rawls.pdf?utm_source=chatgpt.com)
- [An Analysis of the Association Between Herpes Simplex ...](https://www.gorgas.gob.pa/wp-content/uploads/external/BiblioGorgas/pdf/An%20analysis%20of%20the%20association%20between%20herpes%20simplex%20virus%20type%202%20antibodies%20and%20cervical%20cancer.%20William%20Rawls.pdf?utm_source=chatgpt.com)
- [1980). The association between cervical cancer and HSV 2 could represent one in which the virus is causally related to the development of the malignancy, or ...](https://www.gorgas.gob.pa/wp-content/uploads/external/BiblioGorgas/pdf/An%20analysis%20of%20the%20association%20between%20herpes%20simplex%20virus%20type%202%20antibodies%20and%20cervical%20cancer.%20William%20Rawls.pdf?utm_source=chatgpt.com)
- [researchgate.net](https://www.researchgate.net/publication/274431983_Herpes_Simplex_Virus_and_Risk_of_Cervical_Cancer_A_Longitudinal_Nested_Case-Control_Study_in_the_Nordic_Countries?utm_source=chatgpt.com)
- [Herpes Simplex Virus and Risk of Cervical Cancer](https://www.researchgate.net/publication/274431983_Herpes_Simplex_Virus_and_Risk_of_Cervical_Cancer_A_Longitudinal_Nested_Case-Control_Study_in_the_Nordic_Countries?utm_source=chatgpt.com)
- [Aug 5, 2025 — Herpes simplex virus type 2 (HSV2) was first associated with human cervical cancer by seroepidemiologic studies over 10 years ago, but there ...](https://www.researchgate.net/publication/274431983_Herpes_Simplex_Virus_and_Risk_of_Cervical_Cancer_A_Longitudinal_Nested_Case-Control_Study_in_the_Nordic_Countries?utm_source=chatgpt.com)
- [iarc.fr](https://screening.iarc.fr/doc/RH_natural_history_of_cc_fs.pdf?utm_source=chatgpt.com)
- [[PDF] Natural History of Cervical Cancer - IARC Screening Group](https://screening.iarc.fr/doc/RH_natural_history_of_cc_fs.pdf?utm_source=chatgpt.com)
- [Few women who develop dysplasia will progress to cervical cancer. • Progression to detect- able, precancerous lesions can take as long as 10 years. One ...](https://screening.iarc.fr/doc/RH_natural_history_of_cc_fs.pdf?utm_source=chatgpt.com)
- [oup.com](https://academic.oup.com/aje/article/156/8/687/78122?utm_source=chatgpt.com)
- [Herpes Simplex Virus and Risk of Cervical Cancer](https://academic.oup.com/aje/article/156/8/687/78122?utm_source=chatgpt.com)
- [by M Lehtinen · 2002 · Cited by 147 — Since the end of the 1960s, herpes simplex virus type 2 (HSV-2) had been considered the major cause of invasive cervical carcinoma, but a longitudinal ...](https://academic.oup.com/aje/article/156/8/687/78122?utm_source=chatgpt.com)
- [stjude.org](https://sjr-redesign.stjude.org/content/dam/research-redesign/centers-initiatives/hpv-cancer-prevention-program/hpv-advocacy-campaign/history-hpv-vaccination.pdf?utm_source=chatgpt.com)
- [[PDF] History of HPV Vaccination - Home | St. Jude Research](https://sjr-redesign.stjude.org/content/dam/research-redesign/centers-initiatives/hpv-cancer-prevention-program/hpv-advocacy-campaign/history-hpv-vaccination.pdf?utm_source=chatgpt.com)
- [Scientists discovered that HPV causes cancer. After centuries of misconceptions surrounding the causes of cervical cancer, Dr. Richard Shope hypothesized ...](https://sjr-redesign.stjude.org/content/dam/research-redesign/centers-initiatives/hpv-cancer-prevention-program/hpv-advocacy-campaign/history-hpv-vaccination.pdf?utm_source=chatgpt.com)
- [ajog.org](https://www.ajog.org/article/S0002-9378%2802%2971396-3/abstract?utm_source=chatgpt.com)
- [Herpes simplex virus type II is not a cofactor to human ...](https://www.ajog.org/article/S0002-9378%2802%2971396-3/abstract?utm_source=chatgpt.com)
- [by D Tran-Thanh · 2003 · Cited by 73 — Conclusion: Although herpes simplex virus type 2 Bgl IIN transforms epithelial cells in vitro, it was not detected in cervical cancer specimens. (Am J Obstet ...](https://www.ajog.org/article/S0002-9378%2802%2971396-3/abstract?utm_source=chatgpt.com)
- [biomedcentral.com](https://bmccancer.biomedcentral.com/articles/10.1186/s12885-017-3734-2?utm_source=chatgpt.com)
- [Seropositivity to herpes simplex virus type 2, but not type 1 is ...](https://bmccancer.biomedcentral.com/articles/10.1186/s12885-017-3734-2?utm_source=chatgpt.com)
- [by S Li · 2017 · Cited by 58 — HSV2 seropositivity was associated with cancer occurrence, cervical cancer and uterine cancer, but not breast cancer or ovarian cancer. In ...](https://bmccancer.biomedcentral.com/articles/10.1186/s12885-017-3734-2?utm_source=chatgpt.com)
- [unl.edu](https://digitalcommons.unl.edu/cgi/viewcontent.cgi?article=1038&context=vetscipapers&utm_source=chatgpt.com)
- [Cervical Cancer: Is Herpes Simplex Virus Type II a Cofactor?](https://digitalcommons.unl.edu/cgi/viewcontent.cgi?article=1038&context=vetscipapers&utm_source=chatgpt.com)
- [by CJ Jones · 1995 · Cited by 84 — There is no doubt that cervical cells can survive infection by HPV plus HSV-2 and then progress to cervical cancer, because DNA from both viruses has been ...](https://digitalcommons.unl.edu/cgi/viewcontent.cgi?article=1038&context=vetscipapers&utm_source=chatgpt.com)
- [wiley.com](https://onlinelibrary.wiley.com/doi/abs/10.1002/ijc.2910380314?utm_source=chatgpt.com)
- [Failure to induce cervical cancer in mice by long‐term ...](https://onlinelibrary.wiley.com/doi/abs/10.1002/ijc.2910380314?utm_source=chatgpt.com)
- [by B Meignier · 1986 · Cited by 14 — The results do not support the ability of herpes simplex viruses to cause genital neoplasia in mice.](https://onlinelibrary.wiley.com/doi/abs/10.1002/ijc.2910380314?utm_source=chatgpt.com)
- [karger.com](https://karger.com/books/book/chapter-pdf/2026987/000400861.pdf?utm_source=chatgpt.com)
- [The Genital Herpes-Cervical Cancer Hypothesis](https://karger.com/books/book/chapter-pdf/2026987/000400861.pdf?utm_source=chatgpt.com)
- [About 10 years have passed since the Emory University group first noted an association between genital herpes simplex virus (HSV) infection and](https://karger.com/books/book/chapter-pdf/2026987/000400861.pdf?utm_source=chatgpt.com)

**References:**

1. The first reference, zur Hausen H. (1976), **is correct.**
2. The Gissmann (1983) citation **is incorrect,** it should be: Gissmann L, Boshart M, Dürst M, Ikenberg H, Wagner D, zur Hausen H. Presence of human papillomavirus in genital tumors. J Invest Dermatol. 1984 Jul;83(1 Suppl):26s-28s. doi: 10.1111/1523-1747.ep12281143.
3. Reference 3, Boshart, M., Gissmann, L., Ikenberg, H., Kleinheinz, A., Scheurlen, W., & zur Hausen, H. (1984), **is correct.**
4. Reference 4 Syrjänen, K. J. (1983) **is incorrect.** It should be: Syrjänen KJ. Histological changes identical to those of condylomatous lesions found in esophageal squamous cell carcinomas. Arch Geschwulstforsch. 1982;52(4):283-92. PMID: 7138240.
5. Reference 5 is the landmark paper, Durst, M., Gissmann, L., Ikenberg, H., & zur Hausen, H. (1983), and **is correct.**
6. Reference 6, the zurHausen 1986 paper, **is incorrect.** It should be: zur Hausen H. Papillomaviruses in anogenital cancer as a model to understand the role of viruses in human cancers. Cancer Res. 1989 Sep 1;49(17):4677-81. PMID: 2547512.
7. Reference 7 (referred to as reference 1 in the HSV section), Nahmias, A. J., Naib, Z. M., Josey, W. E., et al. (1970)., **is incorrect,** it should be: Nahmias AJ, Josey WE, Naib ZM, Luce CF, Guest BA. Antibodies to Herpesvirus hominis types 1 and 2 in humans. II. Women with cervical cancer. Am J Epidemiol. 1970 Jun;91(6):547-52. doi: 10.1093/oxfordjournals.aje.a121166. PMID: 4315624.
8. The reference Rawls, W. E., Tompkins, W. A. F., Melnick, J. L. (1973) **is incorrect.** It should be: Rawls WE, Tompkins WA, Figueroa ME, Melnick JL. Herpesvirus type 2: association with carcinoma of the cervix. Science. 1968 Sep 20;161(3847):1255-6. doi: 10.1126/science.161.3847.1255. PMID: 4299806.
9. The reference Dreesman, G. R., et al. (1980) **is correct**.
10. The reference Sever, J. L., Madden, D. L., Kuff, E. L., et al. (1982) **doesn’t exist.** There is an article with the same title, but with different authors from a different journal, on a different date. The citation is: Bosch FX, Muñoz N. The viral etiology of cervical cancer. Virus Res. 2002 Nov;89(2):183-90. doi: 10.1016/s0168-1702(02)00187-9. PMID: 12445658.
11. The Rawls W.E., et al. (1986) paper also **doesn’t exist.** There is a 1992 paper with a similar title: ​​Parazzini F, La Vecchia C, Negri E, Fedele L, Franceschi S, Gallotta L. Risk factors for cervical intraepithelial neoplasia. Cancer. 1992 May 1;69(9):2276-82. doi: 10.1002/1097-0142(19920501)69:9<2276::aid-cncr2820690912>3.0.co;2-q. PMID: 1562973.
12. The next reference starts the “reviews” section and is labeled as reference 1: zur Hausen, H. (1982). “Human genital cancer: synergism between two virus infections or two viral genes?” Lancet 2(8302): 1370–1372. **It is incorrect** and should be: zur Hausen H. Human genital cancer: synergism between two virus infections or synergism between a virus infection and initiating events? Lancet. 1982 Dec 18;2(8312):1370-2. doi: 10.1016/s0140-6736(82)91273-9. PMID: 6129466.
13. The McDougall, J. K., et al. (1983) reference **is incorrect.** The title is correct, but everything else is wrong. The closest I found was a Letter in BMJ: Mohanty, K.C., Viruses and Cervical Cancer. BMJ. 1991 Mar 16;302(6777):659–660.
14. Finally the zur Hausen (1984) reference is actually from 2002, **so it is beyond the cutoff date and it is incorrect.** It should be: zur Hausen, H. Papillomaviruses and cancer: from basic studies to clinical application. Nat Rev Cancer 2, 342–350 (2002). https://doi.org/10.1038/nrc798

## Question- response to LLMs about scrapie being caused by protein or virus before 1985

### Scrapie Question:

Please evaluate the following competing hypotheses using only information from 1985 or before. Please provide references showing where you got this information. Here are the hypotheses: "Is it more likely that scrapie is caused by a viral infection or by a protein infection?"

### ChatGPT response

To evaluate the competing hypotheses about the cause of **scrapie**—a transmissible spongiform encephalopathy (TSE) in sheep and goats—we must examine the state of scientific understanding up to **1985**. The two hypotheses are:

1. **Scrapie is caused by a viral infection.**
2. **Scrapie is caused by a protein infection (i.e., a prion).**

**Background**

Scrapie has been recognized for centuries, but its etiology remained mysterious throughout most of the 20th century. By the 1960s–1980s, scrapie was known to be **transmissible**, but its resistance to standard viral inactivation procedures posed a challenge to the viral hypothesis.

**Hypothesis 1: Scrapie is caused by a viral infection**

Evidence in Favor (pre-1985):

- Early studies often assumed that scrapie was caused by a **slow virus**, similar to how certain neurological diseases like kuru or Creutzfeldt-Jakob disease (CJD) were being understood at the time.
- **Gajdusek and Zigas (1957)** described kuru as a slow viral infection, and this framework was extended to scrapie:

   *Gajdusek, D. C., & Zigas, V. (1957). Degenerative disease of the central nervous system in New Guinea: The epidemic of kuru in the native population. New England Journal of Medicine, 257(20), 974–979.*
- The “unconventional virus” idea persisted into the 1970s and early 1980s. **Pattison (1965)** emphasized the infectious nature of the agent, consistent with a virus:

   *Pattison, I. H. (1965). Experiments on the cause of scrapie: a transmissible disease of sheep. Journal of Comparative Pathology, 75, 159–171.*
- Studies showed the scrapie agent could be **transmitted to rodents** and **retained infectivity** after filtration and passage through species barriers—features compatible with certain types of viruses.

**Hypothesis 2: Scrapie is caused by a protein infection (a prion)**

Evidence in Favor (pre-1985):

- Beginning in the late 1970s, **Stanley Prusiner** proposed that the scrapie agent might not contain nucleic acids, but instead be composed **entirely of protein**—a “**prion**” (proteinaceous infectious particle).
- In **1982**, Prusiner published a key paper suggesting that scrapie was caused by a protein:

   *Prusiner, S. B. (1982). Novel proteinaceous infectious particles cause scrapie. Science, 216(4542), 136–144.* Prusiner showed that scrapie infectivity co-purified with a protein and resisted treatments that would normally destroy nucleic acids.
- In further studies, Prusiner and colleagues demonstrated that:
  - The agent was **resistant to nucleases** (which degrade DNA/RNA),
  - It remained infectious after **UV radiation** that would typically inactivate viruses,
  - It was **inactivated by protein-denaturing agents**, supporting the protein-only hypothesis.
- By **1984**, Prusiner refined and defended the prion hypothesis:

   *Prusiner, S. B. (1984). Some speculations about prions, amyloid, and Alzheimer’s disease. New England Journal of Medicine, 310(10), 661–663.*

Evaluation as of 1985

Support for the Viral Hypothesis:

- It had been the prevailing view for decades.
- Scrapie was transmissible and followed incubation patterns typical of infectious agents.

Problems with the Viral Hypothesis:

- The agent **lacked detectable nucleic acids**, even with increasingly sensitive methods.
- It withstood **UV radiation**, **nucleases**, and **formaldehyde**, all of which would inactivate most viruses.

Support for the Protein (Prion) Hypothesis:

- Experimental data strongly suggested the infectious agent was a **protein without nucleic acid**.
- The term **"prion"** was introduced and experimentally backed by Prusiner and others between 1982–1985.

**Conclusion (as of 1985)**

By 1985, the **protein (prion) hypothesis** was **more strongly supported** by experimental evidence than the viral hypothesis. Though controversial, it better explained the **unusual resistance** of the scrapie agent to conventional viral inactivation and the **lack of detectable nucleic acids**.

Thus, as of 1985, **it was more likely that scrapie was caused by a protein infection**.

**Key References (pre-1985)**

1. Prusiner, S. B. (1982). *Novel proteinaceous infectious particles cause scrapie*. Science, 216(4542), 136–144.
2. Prusiner, S. B. (1984). *Some speculations about prions, amyloid, and Alzheimer’s disease*. New England Journal of Medicine, 310(10), 661–663.
3. Pattison, I. H. (1965). *Experiments on the cause of scrapie: a transmissible disease of sheep*. Journal of Comparative Pathology, 75, 159–171.
4. Gajdusek, D. C., & Zigas, V. (1957). *Degenerative disease of the central nervous system in New Guinea: The epidemic of kuru in the native population*. New England Journal of Medicine, 257(20), 974–979.

### Comments on above

The LLM correctly predicted that by 1985 the protein hypothesis had stronger support. Three out of four references are correct.

1. Prusiner, 1982 was the landmark study saying that scrapie was caused by a protein. This reference **is correct.**
2. Prusiner, S. B. (1984) is also a **correct reference.**
3. Pattison I. H. (1965) **does not seem to exist.** There is a 1961 article about observations of scrapie affecting goats and sheep by I.H. Pattison and G.C. Millison: <https://doi.org/10.1016/S0368-1742(60)80018-5> , but the title and date and journal issue and page number are all wrong.
4. Gajdusek, D. C., & Zigas, V. (1957) is a **legitimate reference.**

### Gemini response

In 1985, the scientific community was actively debating the nature of the scrapie infectious agent. While the "slow virus" hypothesis had been dominant for some time due to the long incubation periods of transmissible spongiform encephalopathies (TSEs) like scrapie, growing evidence was pointing towards a proteinaceous infectious particle.^1^

**Evidence for a Viral Infection (pre-1985):**

- **"Slow Virus" Concept:** The term "slow virus" was coined in 1954 by Bjorn Sigurdsson, drawing parallels between scrapie's long incubation period and other slow viral infections.^2^ This concept was influential in framing the early understanding of these diseases.
  - **Reference:** Sigurdsson, B. (1954).^3^ Observations on maedi, a chronic progressive pneumonia of sheep. *British Veterinary Journal*, *110*(6), 255-270. (While this paper is on maedi, Sigurdsson's work on slow infections and the concept's application to scrapie were well-established by 1985).
- **Transmissibility:** Scrapie was successfully transmitted between animals by inoculating brain and cerebral spinal fluid, which historically supported the idea of an infectious agent like a virus.
  - **Reference:** Plummer, H. (1946). Transmission of scrapie by intracerebral inoculation of tissue from scrapie-infected sheep. *Journal of Comparative Pathology and Therapeutics*, *56*(1), 22-26. (While this specific paper is from 1946, the concept of transmissibility as evidence for a viral agent was still relevant.)
- **Host-controlled Incubation Period (Sinc Gene):** Research by Dickinson and colleagues identified a gene (later named *Sinc* for scrapie incubation) in mice that seemed to control the incubation period of scrapie, suggesting a genetic interaction that could be compatible with a viral infection where host genes influence susceptibility.^4^
  - **Reference:** Dickinson, A. G., Meikle, V. M. H., & Fraser, H. (1968).^5^ Identification of a gene (Sinc) controlling the duration of scrapie incubation period in mice.^6^ *Journal of Comparative Pathology*, *78*(3), 293-299. (Again, this paper is pre-1985, but the concept it introduced was still debated.)
- **Detection of RNA:** Some studies in 1985 using [3H]uridine for labeling found differences in RNA levels in various fractions of scrapie-infected brain, suggesting the presence of metabolically stable RNA.
  - **Reference:** Diringer, H., & Kimberlin, R. H. (1985). Scrapie: concept of a virus‐induced amyloidosis of the brain. *The EMBO Journal*, *4*(9), 1721-1725.

**Evidence for a Protein Infection (Prion Hypothesis) (pre-1985):**

- **Resistance to Inactivation:** A significant challenge to the viral hypothesis was the extraordinary resistance of the scrapie agent to inactivation methods known to destroy nucleic acids, such as ionizing radiation, UV radiation, extreme heat, and certain chemicals.^7^ This led some researchers to suggest that the agent replicated without nucleic acid.
  - **Reference:** Alper, T., Cramp, W. A., Haig, D. A., & Clarke, M. C. (1967).^8^ Does the agent of scrapie replicate without nucleic acid? *Nature*, *214*(5090), 764-766.^9^
  - **Reference:** Hunter, G. D., & Millson, G. C. (1964).^10^ Inactivation of the scrapie agent by UV irradiation. *Journal of General Microbiology*, *37*(3), 369-373.
- **Proteinaceous Nature:** J.S. Griffith, in 1967, proposed that the scrapie agent could be a protein, offering several theoretical models for how a protein could be an infectious agent.^11^
  - **Reference:** Griffith, J. S. (1967).^12^ Self-replication and scrapie. *Nature*, *215*(5105), 1043-1044.
- **Coined Term "Prion":** In 1982, Stanley Prusiner coined the term "prion" (proteinaceous infectious particle) to describe the infectious agent of scrapie, emphasizing its protein nature and resistance to nucleic acid-modifying procedures.^13^
  - **Reference:** Prusiner, S. B. (1982).^14^ Novel proteinaceous infectious particles cause scrapie.^15^ *Science*, *216*(4542), 136-144.
- **Identification and Purification of PrP 27-30:** In the early 1980s, Prusiner and colleagues identified a specific protein, PrP 27-30, that purified with scrapie infectivity.^16^ This protein was resistant to proteinase K digestion.
  - **Reference:** Bolton, D. C., McKinley, M. P., & Prusiner, S. B. (1982). Identification of a protein that purifies with the scrapie prion. *Science*, *218*(4579), 1309-1311.^17^
  - **Reference:** Bolton, D. C., McKinley, M. P., & Prusiner, S. B. (1984). Molecular characteristics of the major scrapie prion protein. *Biochemistry*, *23*(25), 5898-5906.
- **Host-Encoded Protein:** Crucially, by 1985, it was shown that the mRNA for the prion protein (PrP) was present in both scrapie-infected and uninfected brains, indicating that the protein was encoded by the host genome, not by a foreign viral genome.^18^
  - **Reference:** Oesch, B., Westaway, D., Wälchli, M., McKinley, M. P., Kent, S. B., Aebersold, P. R., ... & Prusiner, S. B. (1985). A cellular gene encodes scrapie PrP 27-30 protein.^19^ *Cell*, *40*(4), 735-746.
  - **Reference:** Chesebro, B., Race, R., Wehrly, K., Nishio, J., Bloom, M., Lechner, D., ... & Keith, J. M. (1985). Identification of scrapie prion protein-specific mRNA in scrapie-infected and uninfected brain. *Nature*, *315*(6017),^20^ 331-333.
- **Similarities with CJD:** The purification procedures and molecular properties of the Creutzfeldt-Jakob disease (CJD) agent were found to be strikingly similar to those of scrapie prions, suggesting a common proteinaceous nature for these transmissible spongiform encephalopathies.^21^
  - **Reference:** Prusiner, S. B., Cochran, S. P., & Alpers, M. P. (1985). Scrapie and Creutzfeldt-Jakob disease prion proteins share physical properties and antigenic determinants.^22^ *Proceedings of the National Academy of Sciences*,^23^ *82*(4), 997-1001.

**Evaluation:**

Based on the information available in 1985, **it was more likely that scrapie was caused by a protein infection (prion) than a viral infection.**

While the "slow virus" hypothesis had a historical basis due to the transmissibility and long incubation periods, the evidence against a nucleic acid component of the scrapie agent was substantial and accumulating rapidly in the early 1980s.^24^ The extreme resistance of the infectivity to treatments that destroy nucleic acids, coupled with the identification of a specific host-encoded protein (PrP 27-30) that co-purified with infectivity, strongly supported the protein-only hypothesis proposed by Prusiner.^25^ The crucial finding that the PrP gene was present in both healthy and infected individuals further undermined the viral theory, as it indicated that the infectious agent was not introducing novel genetic material. While some studies still reported the presence of stable RNA in infected brains, the overall weight of evidence strongly favored a proteinaceous infectious agent.

### Comments on above:

The LLM correctly predicts that scrapie is caused by a protein not a virus. For references, first while some pre-1985 sources are cited throughout the text, Gemini also gives a list of its sources to the side. These include:

1. A 2020 review article about pathogenesis, detection, and control of scrapie virus: <https://doi.org/10.2460/ajvr.81.7.600> **(beyond the cut-off date).**
2. A review written by Prusiner about prions in 1998: <https://doi.org/10.1073/pnas.95.23.13363> **(beyond the cut-off date).**
3. A 2017 article about protein aggregation and neurodegenerative diseases: <https://doi.org/10.3389/fnagi.2017.00187> **(beyond the cut-off date).**
4. A 1968 article about a gene that controls some strains of the scrapie agent: <https://doi.org/10.1016/0021-9975(68)90005-4>
5. A 2021 review about prion mechanisms in sheep and relation to Alzheimers disease: [**https://doi.org/10.3390/ijms22094861**](https://doi.org/10.3390/ijms22094861) **(beyond the cut-off date).**
6. A 2000 article about high heat resistance of a scrapie agent: <https://doi.org/10.1073/pnas.97.7.3418> **(beyond the cut-off date).**
7. A 1967 article about scrapie replicating without a nucleic acid: <https://doi.org/10.1038/214764a0>
8. A 2015 article about the brief history of prions: <https://doi.org/10.1093/femspd/ftv087> **(beyond the cut-off date).**
9. A 2008 article about prions in fungi: <https://doi.org/10.1038/nrmicro1708> **(beyond the cut-off date).**
10. A 2012 letter to the editor of Emerging INfectious diseaeses about what a prion is: <https://doi.org/10.3201/eid1806.120271> **(beyond the cut-off date).**
11. A data sheet on scrapie from 2019: <https://doi.org/10.1079/cabicompendium.64847> **(beyond the cut-off date).**
12. A 1985 article about scrapie prion proteins: <https://doi.org/10.1073/pnas.82.4.997>
13. A 2013 article about prions: <https://doi.org/10.1093/brain/awt179> **(beyond the cut-off date).**
14. A 1985 article about scrapie protein-specific mRNA in both infected and uninfected brains: <https://doi.org/10.1038/315331a0>
15. 2021 article about prion diseases: <https://doi.org/10.3390/biom11020207> **(beyond the cut-off date).**

So in the sources section, 11 out of 15 sources are after 1985, many of them historical accounts that were written about the prion much later. This indicates that the other references produced in the text were likely taken from these later accounts.

As for the references cited within the answer:

1. Sigurdsson, B. (1954). This reference is **cited incorrectly** (date and journal are wrong), and this has to do with maedi, not scrapie.
2. Plummer, H. (1946). This reference **doesn’t exist.**
3. Dickinson, A. G., Meikle, V. M. H., & Fraser, H. (1968) is a **real and legitimate reference.**
4. Diringer, H., & Kimberlin, R. H. (1985). This reference has been **cited incorrectly.** The authors should be Diringer and Braig: <https://doi.org/10.1002/j.1460-2075.1985.tb03931.x>
5. Alper, T., Cramp, W. A., Haig, D. A., & Clarke, M. C. (1967). This is a **legitimate reference.**
6. Hunter, G. D., & Millson, G. C. (1964). This is actually a 1985 paper (at least the title is from a 1985 paper). Authors, date of publication, etc are all wrong, thus the **citation is incorrect.** <https://doi.org/10.1099/0022-1317-66-4-845>
7. Griffith, J. S. (1967) is **a legitimate article**
8. Prusiner, S. B. (1982). Was the landmark paper (**is a legitimate reference**).
9. Bolton, D. C., McKinley, M. P., & Prusiner, S. B. (1982). **Is a legitimate paper.**
10. Bolton, D. C., McKinley, M. P., & Prusiner, S. B. (1984). **is a legitimate reference.**
11. Oesch, B et al. (1985) **is a legitimate paper.**
12. Chesebro, B et al. 1985 **is a legitimate paper.**
13. For Prusiner, S. B., Cochran, S. P., & Alpers, M. P. (1985), the authors are wrong, should be Bendheim, Bockman, Kingsbury, and Prusiner: <https://doi.org/10.1073/pnas.82.4.997>, and is thus **incorrect.**

### DeepSeek response

To evaluate the hypotheses regarding the cause of scrapie using only information available by 1985, we need to rely on the scientific understanding of the time. Here’s an analysis based on pre-1985 evidence:

**Hypothesis 1: Scrapie is caused by a viral infection**

By 1985, the dominant theory for scrapie's cause was an unconventional "slow virus." Key evidence included:

1. Transmissibility: Scrapie could be transmitted between animals via inoculation of infected brain tissue (Cuillé & Chelle, 1936).

2. Filterability: The infectious agent passed through filters that blocked bacteria, suggesting a virus-like entity (Sigurdsson, 1954).

3. Long incubation period: Similar to other slow viral infections like kuru or Creutzfeldt-Jakob disease (Gajdusek, 1977).

However, no traditional virus (with nucleic acid) had been isolated, and the agent resisted UV radiation (which typically inactivates viruses) (Alper et al., 1967).

**Hypothesis 2: Scrapie is caused by a protein (prion)**

By 1985, the prion hypothesis was emerging but not yet widely accepted. Key evidence included:

1. Resistance to treatments: The scrapie agent resisted procedures that destroy nucleic acids (radiation, nucleases) but was inactivated by proteases (Prusiner et al., 1982).

2. Protein-only experiments: Stanley Prusiner's 1982 paper proposed the term "prion" (proteinaceous infectious particle) after purifying an infectious protein (PrP) from scrapie-infected brains (Prusiner, 1982).

3. No immune response: Unlike viruses, the agent did not provoke an immune reaction, suggesting no foreign nucleic acid.

**Conclusion (Pre-1985 Evidence)**

**Before 1985, the viral hypothesis was more widely accepted** due to transmissibility and filterability, though the agent's unusual properties (e.g., resistance to UV) made it controversial. The prion hypothesis was still speculative, with key experimental support (Prusiner, 1982) but not yet conclusive.

**References (Pre-1985 Sources)**

1. Cuillé, J., & Chelle, P. L. (1936). La maladie dite tremblante du mouton est-elle inoculable? Comptes Rendus de l'Académie des Sciences, 203, 1552–1554.

2. Sigurdsson, B. (1954). Rida, a chronic encephalitis of sheep. British Veterinary Journal, 110, 341–354.

3. Alper, T., et al. (1967). Does the agent of scrapie replicate without nucleic acid? Nature, 214, 764–766.

4. Gajdusek, D. C. (1977). Unconventional viruses and the origin and disappearance of kuru. Science, 197, 943–960.

5. Prusiner, S. B. (1982). Novel proteinaceous infectious particles cause scrapie. Science, 216, 136–144.

Thus, pre-1985 evidence slightly favored the viral hypothesis, but the prion theory was gaining traction with key experimental results. The definitive proof for prions came later (e.g., PrP gene cloning in 1985).

### Comments about above:

The LLM decides that a viral causation was more likely, although this is after the landmark paper is published so it should be the protein hypothesis. It does say that prion theory was gaining ground. For the references, many were legitimate but not all.

References:

1. Cuille and Chelle (1936): **Citation is wrong**, should be: Cuillé J, Chelle PL. La tremblante du mouton est bien inoculable. C.R. Séances Acad. Sci. Paris. 1936;206:78-79 or Cuillé, J. and Chelle, PL (1936) Scrapie in sheep is easily inoculable. CR Séances Acad. Sci. Paris, 206, 78–79 in English. Likely obtained from a review or wikipedia as this article is referenced in many reviews, but the actual article is difficult to find. It does not come up in google scholar, but with only reviews mentioning it.
2. Sigurdsson (1954) is **a correct reference** although the title has been shortened here. <https://doi.org/10.1016/S0007-1935(17)50172-4>
3. Alper, T., et al. (1967) **is correct and is a legitimate reference.**
4. Gajdusek, D. C. (1977) **is a real and relevant reference.**
5. Prusiner, S. B. (1982) is the landmark paper identifying scrapie as a prion and is **correctly cited.**

### Perplexity response

By 1985, experimental evidence strongly supported the protein infection hypothesis for scrapie over viral causation.

**Key findings include:**

**Evidence Against Viral Etiology**

1. Resistance to pathogen-inactivating treatments:
   - Formalin fixation failed to inactivate scrapie agent in vaccines (1944)[^3^](https://pmc.ncbi.nlm.nih.gov/articles/PMC4626585/)[^5^](https://en.wikipedia.org/wiki/Prion)
   - UV radiation (1966) and heat (1965) didn't reduce infectivity[^1^](https://avmajournals.avma.org/view/journals/ajvr/81/7/ajvr.81.7.600.xml)[^3^](https://pmc.ncbi.nlm.nih.gov/articles/PMC4626585/)
   - Ionizing radiation experiments suggested <1.5% nucleic acid content[^1^](https://avmajournals.avma.org/view/journals/ajvr/81/7/ajvr.81.7.600.xml)[^3^](https://pmc.ncbi.nlm.nih.gov/articles/PMC4626585/)
2. Lack of immune response: No antibodies detected against scrapie agent[^3^](https://pmc.ncbi.nlm.nih.gov/articles/PMC4626585/)

**Evidence for Protein-Only Hypothesis**

1. Prion protein isolation:
   - Prusiner purified protease-resistant PrP27-30 (1982)[^1^](https://avmajournals.avma.org/view/journals/ajvr/81/7/ajvr.81.7.600.xml)[^2^](https://pubmed.ncbi.nlm.nih.gov/6801762/)
   - Infectivity co-purified with PrP concentration[^1^](https://avmajournals.avma.org/view/journals/ajvr/81/7/ajvr.81.7.600.xml)[^2^](https://pubmed.ncbi.nlm.nih.gov/6801762/)
2. Genetic evidence:
   - Host-encoded PrP gene identified (1985)[^1^](https://avmajournals.avma.org/view/journals/ajvr/81/7/ajvr.81.7.600.xml)[^3^](https://pmc.ncbi.nlm.nih.gov/articles/PMC4626585/)[^5^](https://en.wikipedia.org/wiki/Prion)
   - PrP mRNA detected in both infected/uninfected brains (1985)[^1^](https://avmajournals.avma.org/view/journals/ajvr/81/7/ajvr.81.7.600.xml)[^3^](https://pmc.ncbi.nlm.nih.gov/articles/PMC4626585/)
3. Theoretical framework:
   - Griffith's 1967 models for protein-based replication[^1^](https://avmajournals.avma.org/view/journals/ajvr/81/7/ajvr.81.7.600.xml)[^4^](https://citeseerx.ist.psu.edu/document?repid=rep1&type=pdf&doi=ae54aff98e5fd405a7609dc9225be1bbe1e21854)[^5^](https://en.wikipedia.org/wiki/Prion)
   - Prusiner's prion hypothesis (1982) explaining protein-only transmission[^2^](https://pubmed.ncbi.nlm.nih.gov/6801762/)[^3^](https://pmc.ncbi.nlm.nih.gov/articles/PMC4626585/)

**Critical Experiments**

- Prion infectivity survived nucleases but was destroyed by proteases[^2^](https://pubmed.ncbi.nlm.nih.gov/6801762/)[^3^](https://pmc.ncbi.nlm.nih.gov/articles/PMC4626585/)
- PrP-deficient mice showed scrapie resistance[^1^](https://avmajournals.avma.org/view/journals/ajvr/81/7/ajvr.81.7.600.xml)[^3^](https://pmc.ncbi.nlm.nih.gov/articles/PMC4626585/)
- Familial clustering of CJD suggested host protein involvement [^3^](https://pmc.ncbi.nlm.nih.gov/articles/PMC4626585/)

While viral theories dominated early research, the accumulated evidence through 1985 – particularly the agent's biochemical properties, genetic linkages, and protein characterization – made the prion hypothesis the most parsimonious explanation. However, some researchers remained skeptical due to the radical nature of protein-only replication[^3^](https://pmc.ncbi.nlm.nih.gov/articles/PMC4626585/)[^4^](https://citeseerx.ist.psu.edu/document?repid=rep1&type=pdf&doi=ae54aff98e5fd405a7609dc9225be1bbe1e21854).

**References:** [1](https://avmajournals.avma.org/view/journals/ajvr/81/7/ajvr.81.7.600.xml)[2](https://pubmed.ncbi.nlm.nih.gov/6801762/)[3](https://pmc.ncbi.nlm.nih.gov/articles/PMC4626585/)[4](https://citeseerx.ist.psu.edu/document?repid=rep1&type=pdf&doi=ae54aff98e5fd405a7609dc9225be1bbe1e21854)[5](https://en.wikipedia.org/wiki/Prion)

1. <https://avmajournals.avma.org/view/journals/ajvr/81/7/ajvr.81.7.600.xml>
2. <https://pubmed.ncbi.nlm.nih.gov/6801762/>
3. <https://pmc.ncbi.nlm.nih.gov/articles/PMC4626585/>
4. <https://citeseerx.ist.psu.edu/document?repid=rep1&type=pdf&doi=ae54aff98e5fd405a7609dc9225be1bbe1e21854>
5. <https://en.wikipedia.org/wiki/Prion>
6. <https://pmc.ncbi.nlm.nih.gov/articles/PMC1114482/>
7. <https://www.sciencedirect.com/topics/pharmacology-toxicology-and-pharmaceutical-science/scrapie>
8. <https://www.sciencedirect.com/topics/biochemistry-genetics-and-molecular-biology/scrapie>
9. <https://www.j-alz.com/editors-blog/posts/prion-hypothesis-forty-enlightening-or-deceptive>
10. <https://pmc.ncbi.nlm.nih.gov/articles/PMC1681234/>
11. <https://www.veterinary-practice.com/article/scrapie-an-overview>
12. <https://en.wikipedia.org/wiki/Scrapie>
13. <https://pubmed.ncbi.nlm.nih.gov/2579394/>
14. <https://www.cabidigitallibrary.org/doi/10.1079/cabicompendium.64847>
15. <https://pmc.ncbi.nlm.nih.gov/articles/PMC1888142/>
16. <https://www.nature.com/articles/315331a0>
17. <https://avmajournals.avma.org/downloadpdf/journals/ajvr/81/7/ajvr.81.7.600.xml>

### Comments on above:

The LLM correctly predicts that the protein hypothesis has more support in 1985, but most references it used are post 1985.

1. Reference 1 is a review article from 2019 **(post cut-off date)**.
2. Reference 2 is the Prusiner 1982 landmark paper, thus **legitimate**.
3. Reference 3 is a 2015 review paper **(post cut-off date)**.
4. Reference 4 is a 2011 review **(post cut-off date)**.
5. Reference 5 is a wikipedia article (so not from 1985 or before) about prions **(post cut-off date)**.
6. Reference 6 is a historical article from 1998 **(post cut-off date)**.
7. Reference 7 is a chapter in a book from 2015 **(post cut-off date)**.
8. Reference 8 is a chapter from a 2008 book **(post cut-off date)**.
9. Reference 9 is an article from 2022 **(post cut-off date)**.
10. Reference 10 is a 1989 paper about a scrapie outbreak **(post cut-off date)**.
11. Reference 11 is a 2023 article about scrapie **(post cut-off date)**.
12. Reference 12 is a wikipedia article about scrapie **(post cut-off date)**.
13. Reference 13 is a 1985 article comparing scrapie with Creutzfeldt-Jakob disease, thus **legitimate**.
14. Reference 14 is a datasheet on scrapie from 2019 **(post cut-off date)**.
15. Reference 15 is a 1986 article which is **beyond the cutoff**.
16. Reference 16 is a 1985 article about scrapie and mRNA association and is a **legitimate reference.**
17. Reference 17 is the same as reference 1, a 2019 article **(post cut-off date)**.

### ChatGPT-5 response

Excellent question — by 1985, scrapie had been investigated for several decades as a mysterious transmissible neurodegenerative disease in sheep and goats, and the central debate at the time was indeed whether it was caused by a “slow virus” or by an infectious protein (non-nucleic acid agent). Below is an evaluation of both hypotheses as they stood using evidence available in 1985 or earlier, with references to contemporary sources.

**1. Hypothesis 1: Scrapie is caused by a viral infection (“slow virus”)**

**Evidence supporting this view (pre-1985)**

From the 1950s through the early 1970s, the dominant view was that scrapie belonged to a group of “slow virus” diseases:

- Sigurdsson (1954, 1959) introduced the term “slow infection” for diseases with long incubation periods and progressive course (e.g., maedi, visna, and scrapie).
  - References:
    - Sigurdsson, B. (1954). Maedi, a chronic, progressive infection of sheep. J. Infect. Dis. 95:166–175.
    - Sigurdsson, B. (1959). Rida, a chronic encephalitis of sheep with general similarities to scrapie. Brit. Vet. J. 115:163–182.
- Alper et al. (1967, 1970) and others attempted to characterize a viral particle but found the scrapie agent resistant to ultraviolet (UV) radiation at levels that should destroy nucleic acids. Despite this, many still assumed the agent was a virus with an unusually resistant genome or protected by a protein coat.
  - References:
    - Alper, T., Haig, D. A., & Clarke, M. C. (1967). The exceptionally small size of the scrapie agent. Biochem. Biophys. Res. Commun. 29:134–138.
    - Alper, T., Cramp, W. A., Haig, D. A., & Clarke, M. C. (1967). Does the agent of scrapie replicate without nucleic acid? Nature 214:764–766.
    - Alper, T. (1970). The scrapie enigma: insights into the nature of the agent. Nature 225:562–563.
- Many researchers compared scrapie to visna virus and kuru, both thought to be slow viral infections.
  - Example: Gajdusek, D. C., Gibbs, C. J., & Alpers, M. (1966). Experimental transmission of a kuru-like syndrome to chimpanzees. Nature 209:794–796.

Thus, through the 1970s, the mainstream assumption was that scrapie was due to an unconventional, perhaps defective, virus.

**2. Hypothesis 2: Scrapie is caused by a protein infection (proteinaceous infectious particle, or “prion”)**

**Evidence supporting this view (pre-1985)**

By the late 1970s and early 1980s, Stanley Prusiner and others began to challenge the viral hypothesis.

- Griffith (1967) first proposed that scrapie might be caused by a self-replicating protein that could induce conformational change in normal host protein—without nucleic acid.
  - Reference:
    - Griffith, J. S. (1967). Self-replication and scrapie. Nature 215:1043–1044.
- Prusiner et al. (1982) purified scrapie agent preparations and found no detectable nucleic acid, proposing that the infectious entity was a “proteinaceous infectious particle,” or prion.
  - References:
    - Prusiner, S. B. (1982). Novel proteinaceous infectious particles cause scrapie. Science 216:136–144.
    - Bolton, D. C., McKinley, M. P., & Prusiner, S. B. (1982). Identification of a protein that purifies with the scrapie prion. Science 218:1309–1311.
- These studies found the scrapie agent resistant to UV and ionizing radiation (which destroy nucleic acids) but sensitive to protein-denaturing treatments (e.g., proteases, phenol).
  - Reference: Alper et al., 1978. Resistance of scrapie agent to ionizing radiation. Nature 271:763–764.

By 1985, this “protein-only” hypothesis remained highly controversial, but it had gained significant attention due to the biochemical evidence.

**3. Evaluation (as of 1985)**

**Viral hypothesis:**

- Strengths (by 1985):
  - Long-standing tradition of infectious disease paradigms; transmissibility demonstrated since the 1930s.
  - Similarities to other presumed “slow virus” diseases (visna, kuru).
- Weaknesses:
  - No viral particles had ever been visualized by electron microscopy.
  - No nucleic acid could be detected despite sensitive methods.
  - Extraordinary resistance to radiation that should destroy any virus.

**Protein hypothesis:**

- Strengths (by 1985):
  - Explained resistance to nucleic-acid–destroying treatments.
  - Supported by purification and biochemical analyses showing protein dependence.
- Weaknesses:
  - No known biological mechanism for replication of a protein-only agent.
  - Viewed skeptically by many virologists as violating the central dogma.

**4. Conclusion (state of knowledge as of 1985)**

By 1985, both hypotheses were actively debated, but the weight of biochemical and radiobiological evidence was increasingly difficult to reconcile with a viral agent. The protein-only (prion) hypothesis proposed by Griffith (1967) and advanced by Prusiner (1982–1984) was gaining support, though it was not yet accepted by most scientists.

Thus, as of 1985, it was more likely, based on available evidence, that scrapie was caused by an infectious protein rather than a conventional virus, though the mechanism remained unexplained and controversial.

**Key References (≤1985)**

1. Sigurdsson, B. (1954, 1959). Maedi, Rida and the slow infection concept.
2. Alper, T. et al. (1967). Nature 214:764–766.
3. Griffith, J. S. (1967). Self-replication and scrapie. Nature 215:1043–1044.
4. Gajdusek, D. C. et al. (1966). Nature 209:794–796.
5. Alper, T. et al. (1978). Resistance of scrapie agent to ionizing radiation. Nature 271:763–764.
6. Prusiner, S. B. (1982). Novel proteinaceous infectious particles cause scrapie. Science 216:136–144.
7. Bolton, D. C., McKinley, M. P., & Prusiner, S. B. (1982). Identification of a protein that purifies with the scrapie prion. Science 218:1309–1311.

### Comments on above

Here the LLM correctly says that scrapie is most likely caused by an infectious protein, which was the prevailing hypothesis for 1985. For the references, some were correct and others were not:

1. The first 2 references, Sigurdsson, B. (1954, 1959), do not seem to exist as cited:
   1. Sigurdsson, B. (1954). Maedi, a chronic, progressive infection of sheep. J. Infect. Dis. 95:166–175.
      1. This reference should be: SIGURDSSON B, GRIMSSON H, PALSSON PA. Maedi, a chronic, progressive infection of sheep's lungs. J Infect Dis. 1952 May-Jun;90(3):233-41. doi: 10.1093/infdis/90.3.233. PMID: 14938612. Thus the **citation is incorrect.**
   2. Sigurdsson, B. (1959). Rida, a chronic encephalitis of sheep with general similarities to scrapie. Brit. Vet. J. 115:163–182.
      1. This reference should be: Sigurdsson,B., 19552201995, English, Journal article, 0007-1935, 110, British Veterinary Journal, (341–354), Rida a chronic encephalitis of sheep with general remarks on infections which develop slowly and some of their special characteristics., (1954), thus the **citation is incorrect.**
2. Alper, T., Cramp, W. A., Haig, D. A., & Clarke, M. C. (1967). *Does the agent of scrapie replicate without nucleic acid?* Nature 214:764–766. **Is correct.**
3. Alper, T., Haig, D. A., & Clarke, M. C. (1967). *The exceptionally small size of the scrapie agent.* Biochem. Biophys. Res. Commun. 29:134–138. **Is incorrect**. It should be: Alper T, Haig DA, Clarke MC. The exceptionally small size of the scrapie agent. Biochem Biophys Res Commun. 1966 Feb 3;22(3):278-84.
4. Alper, T. (1970). *The scrapie enigma: insights into the nature of the agent.* Nature 225:562–563. **Doesn’t exist.**
5. Griffith, J. S. (1967). *Self-replication and scrapie.* Nature 215:1043–1044. **Is correct.**
6. Prusiner, S. B. (1982). *Novel proteinaceous infectious particles cause scrapie.* Science 216:136–144. **Is correct.**
7. Bolton, D. C., McKinley, M. P., & Prusiner, S. B. (1982). *Identification of a protein that purifies with the scrapie prion.* *Science* 218:1309–1311. **Is correct.**

## Question- response to LLMs about whether peptic ulcers are more likely caused by stress or bacteria pre-1987

### Peptic ulcer question:

Please evaluate the following competing hypotheses using only information from 1987 or before. Please provide references showing where you got this information. Here are the hypotheses: "Is it more likely that peptic ulcer is caused by psychological stress or by a bacterial infection?"

### ChatGPT response

As of 1987 or earlier, the cause of peptic ulcers was a subject of active scientific debate, with two major competing hypotheses:

✅ Hypothesis 1: Peptic ulcers are caused by psychological stress

✅ Hypothesis 2: Peptic ulcers are caused by bacterial infection, especially Helicobacter pylori (then called Campylobacter pyloridis)

🔍 **Overview of Scientific Evidence by 1987**

🔹 1. **Psychological Stress Hypothesis**

Historical Background:
 From the early to mid-20th century, the dominant view in medicine was that emotional stress and personality traits (e.g., Type A personality) were key contributors to peptic ulcer development.

Key Supporting Sources Before 1987:

- Alexander, F. (1950). Psychosomatic Medicine: Its Principles and Applications.
  - Proposed that ulcers developed in people who internalized stress and had unresolved emotional conflicts.
  - Psychological stress was believed to increase gastric acid secretion, damaging the mucosa.
  - [Widely cited in psychosomatic medicine literature through the 1950s–1970s.]
- Engel, G.L. (1959). “Psychologic Development in Health and Disease.”
  - Argued for a biopsychosocial model, using peptic ulcer as a key example.
  - Claimed that life stress and internal conflict could manifest physically in the gastrointestinal system.
- Broadhurst, A.M. (1957). Peptic ulcer and personality. British Journal of Medical Psychology, 30(1): 1–13.
  - Suggested a correlation between certain personality traits and ulcer incidence.
- Clinical consensus:
  - As of the 1970s, textbooks and medical education emphasized stress, smoking, and diet (e.g., spicy foods) as contributing factors, though empirical mechanisms were unclear.

❗ Limitations:

- Evidence was largely correlational or based on case studies.
- No reproducible mechanistic model directly linking stress to ulcer formation.
- Treatments focused on antacids, diet, and stress reduction, often with variable success and frequent recurrence.

🔹 **2. Bacterial Infection Hypothesis (Helicobacter pylori)**

Emerging Paradigm by the Early 1980s:

- Marshall & Warren (1983): Reported isolation of Campylobacter pyloridis (later renamed Helicobacter pylori) from the gastric mucosa of ulcer patients.
   📄 Warren, J.R., & Marshall, B.J. (1983). "Unidentified curved bacilli on gastric epithelium in active chronic gastritis." The Lancet, 321(8336), 1273–1275.
  - Found bacteria consistently in biopsies from patients with gastritis and duodenal ulcers.
- Marshall et al. (1985): Demonstrated that treatment with antibiotics could eradicate the bacterium and reduce ulcer recurrence.
   📄 Marshall, B.J., Armstrong, J.A., McGechie, D.B., & Glancy, R.J. (1985). "Attempt to fulfill Koch’s postulates for pyloric Campylobacter." Medical Journal of Australia, 142(8): 436–439.
  - Included Marshall’s famous self-experiment in which he ingested H. pylori and developed gastritis.
- Blaser, M.J. (1987). "Campylobacter pylori and the pathogenesis of gastroduodenal inflammation." Journal of Infectious Diseases, 155(3): 524–529.
  - Reviewed growing evidence that C. pyloridis played a key role in chronic gastritis and peptic ulcer disease.

❗ Limitations (as of 1987):

- Still controversial and not widely accepted.
- Critics argued that the bacterium might be a commensal or secondary colonizer rather than a primary cause.
- Mechanism of acid resistance and exact pathogenicity was not fully elucidated yet.

🔍 **Comparative Evaluation as of 1987**

| Factor | Psychological Stress | Bacterial Infection (H. pylori) |
| --- | --- | --- |
| Scientific Acceptance | Widely accepted in mid-20th century | Emerging but controversial by 1987 |
| Evidence Type | Correlational, observational | Experimental, microbiological |
| Mechanism | Vague (via stress → acid secretion) | Colonization → inflammation → ulceration |
| Treatment Effectiveness | Stress relief, acid suppression (partial) | Antibiotics + acid suppression (more effective) |
| Paradigm Shift? | Older, entrenched theory | Gaining traction, especially post-1984 |

✅ **Conclusion (as of 1987):**

Although the stress hypothesis remained influential, by 1987 the bacterial hypothesis was increasingly supported by direct experimental evidence, including the isolation of H. pylori and successful antibiotic treatment. While not yet universally accepted, the evidence for bacterial infection was stronger and mechanistically grounded, signaling a major paradigm shift in ulcer research.

Thus, **as of 1987, it was more likely—based on the available evidence—that peptic ulcers were caused by a bacterial infection than by psychological stress.**

📚 **References**

1. Warren, J.R., & Marshall, B.J. (1983). Unidentified curved bacilli on gastric epithelium in active chronic gastritis. The Lancet, 321(8336), 1273–1275.
2. Marshall, B.J., et al. (1985). Attempt to fulfill Koch’s postulates for pyloric Campylobacter. Medical Journal of Australia, 142(8): 436–439.
3. Blaser, M.J. (1987). Campylobacter pylori and the pathogenesis of gastroduodenal inflammation. Journal of Infectious Diseases, 155(3): 524–529.
4. Alexander, F. (1950). Psychosomatic Medicine: Its Principles and Applications. New York: W.W. Norton.
5. Engel, G.L. (1959). Psychologic Development in Health and Disease.
6. Broadhurst, A.M. (1957). Peptic ulcer and personality. British Journal of Medical Psychology, 30(1): 1–13.

Let me know if you'd like PDFs or quotes from any of these sources.

### Comments on above:

The LLM correctly states that in 1987 peptic ulcers were more likely to be caused by bacterial infections and for references it gets the landmark paper by Warren & Marshall and Marshall’s follow-up paper correct. The other references, however, either have incorrect citations or cannot be found.

1. Warren, J.R., & Marshall, B.J. (1983) is the landmark paper that proposed bacteria as the cause of ulcers (**source is legitimate**).
2. Marshall, B.J., et al. (1985). Is the famous experimental paper where Marshall drinks the *H. pylori* cocktail that resulted in gastritis (**source is legitimate**).
3. Blaser (1987). **This citation is wrong**- the paper was published in 1990 (so after the cutoff date) and was titled “*Helicobacter pylori* and the pathogenesis of gastroduodenal inflammation”
4. Alexander, F. (1950). Is a book reference that is difficult to verify what it says about stress and ulcers because there are no page numbers or a chapter to refer to. Thus this **reference is incomplete.**
5. The Engel (1959) book has the **wrong citation** and is not verifiable without any page numbers or chapters or a publisher. Citation should be for 1962, and title should be “Psychological Development in Health and Disease”.
6. The Broadhurst, A.M. (1957). **This paper does not exist.**

### Gemini response

In 1987, the understanding of peptic ulcer disease was undergoing a significant shift, with two main hypotheses competing for prominence: psychological stress and bacterial infection.

**Hypothesis 1: Peptic Ulcer is Caused by Psychological Stress**

Evidence in 1987:

- Long-standing Belief: For many years prior to 1987, the prevailing medical view was that peptic ulcers were primarily caused by psychological stress, emotional factors, and lifestyle choices such as a rich diet or spicy foods. This idea was deeply ingrained in medical doctrine. (Britannica, "Robin Warren"; ResearchGate, "Stress and peptic ulcer")
- Clinical Observations: Clinicians observed that stress could exacerbate ulcer symptoms. Some studies, even if later critiqued for methodological flaws, suggested an association between stressful life events and ulcer occurrence.^1^ (Journal of Mental Health & Clinical Psychology, "A Novel Psychopathological Model Explains the Pathogenesis of Gastric Ulcers"; ResearchGate, "Stress and peptic ulcer")
- "Psychosomatic" View: Peptic ulcers were often categorized as a "psychosomatic disease," meaning that psychological factors were believed to play a significant role in their development.^2^ (Journal of Mental Health & Clinical Psychology, "A Novel Psychopathological Model Explains the Pathogenesis of Gastric Ulcers"; ResearchGate, "Stress and peptic ulcer")
- Treatment Approaches: Treatment often focused on managing stress, dietary modifications (avoiding irritating foods), and antacids to reduce stomach acid, which was considered the primary aggressive factor. (Practo Consult, "Cure Peptic Ulcer"; The Pharmacologist, "Barry Marshall: Curing Peptic Ulcers")

**References:**

- "A Novel Psychopathological Model Explains the Pathogenesis of Gastric Ulcers." Journal of Mental Health & Clinical Psychology. Available at:<https://www.mentalhealthjournal.org/articles/a-novel-psychopathological-model-explains-the-pathogenesis-of-gastric-ulcers.pdf>
- Britannica. "Robin Warren." Available at:<https://www.britannica.com/biography/Robin-Warren>
- Practo Consult. "Cure Peptic Ulcer." Available at:<https://www.practo.com/consult/cure-peptic-ulcer-i-was-diagnosed-with-peptic-ulcer-last-year-i-have-consulted-gastroenterologist-once-in-three-months/q>
- ResearchGate. "Stress and peptic ulcer: Life beyond Helicobacter." Available at:<https://www.researchgate.net/publication/51299433_Stress_and_peptic_ulcer_Life_beyond_Helicobacter>
- The Pharmacologist. "Barry Marshall: Curing Peptic Ulcers." Available at:<https://thepharmacologist.org/curing-peptic-ulcers/>

**Hypothesis 2: Peptic Ulcer is Caused by a Bacterial Infection**

Evidence in 1987:

- Discovery of Helicobacter pylori: In the early 1980s, Australian scientists Barry Marshall and Robin Warren identified spiral-shaped bacteria, later named Helicobacter pylori (H. pylori), in the stomachs of patients with gastritis and peptic ulcers. Warren first observed these bacteria in 1979, and by the early 1980s, he and Marshall began their collaborative research.^3^ (Nobel Prize, "Barry J. Marshall - Nobel Lecture"; Britannica, "Robin Warren"; The University of Western Australia, "Vale Robin Warren")
- Association with Gastritis and Ulcers: Their studies, including a significant one involving 100 stomach biopsies, consistently showed the presence of H. pylori in almost all patients with gastritis, duodenal ulcers, and gastric ulcers. This suggested a strong correlation between the bacterium and these conditions. (Nobel Prize, "Barry J. Marshall - Nobel Lecture"; Britannica, "Robin Warren")
- Challenging Dogma: This discovery directly challenged the long-held belief that bacteria could not survive in the highly acidic environment of the stomach. The medical community was largely skeptical and resistant to this new idea. (Nobel Prize, "Barry J. Marshall - Nobel Lecture"; Barry Marshall - Wikipedia)
- Marshall's Self-Experimentation (1984, published 1985): To demonstrate the pathogenicity of H.^4^ pylori, Barry Marshall famously drank a culture of the bacteria himself in 1984.^5^ He developed symptoms of acute gastritis, and H. pylori was subsequently cultured from his stomach, fulfilling Koch's postulates for gastritis. This experiment was published in 1985, providing compelling evidence for the bacterial cause of gastritis, a precursor to ulcers. (Barry Marshall - Wikipedia; The Pharmacologist, "Barry Marshall: Curing Peptic Ulcers")
- Early Treatment Success with Antibiotics: While not widely accepted as the standard of care by 1987, early research by Marshall indicated that antibiotic and bismuth salt regimens that killed H. pylori resulted in the cure of duodenal ulcers. (Barry Marshall - Wikipedia)

**References:**

- "Barry J. Marshall - Nobel Lecture." NobelPrize.org. Available at:<https://www.nobelprize.org/uploads/2018/06/marshall-lecture.pdf>
- "Barry Marshall." Wikipedia. Available at:<https://en.wikipedia.org/wiki/Barry_Marshall>
- Britannica. "Robin Warren." Available at:<https://www.britannica.com/biography/Robin-Warren>
- The Pharmacologist. "Barry Marshall: Curing Peptic Ulcers." Available at:<https://thepharmacologist.org/curing-peptic-ulcers/>
- The University of Western Australia. "Vale Robin Warren." Available at:<https://www.uwa.edu.au/news/article/2024/july/vale-robin-warren>

**Evaluation in 1987**

In 1987, the psychological stress hypothesis remained the more widely accepted and dominant view regarding the cause of peptic ulcers within the medical community. Decades of teaching and clinical practice supported this theory, and treatments were largely based on managing stress and acid secretion.

However, the bacterial infection hypothesis, specifically involving Helicobacter pylori, was rapidly gaining ground and presenting a significant challenge to the established paradigm. While still met with considerable skepticism and resistance, the compelling evidence from Marshall and Warren's work, particularly the consistent identification of the bacterium in ulcer patients and Marshall's self-experimentation demonstrating its ability to cause gastritis, provided a strong foundation for the infectious cause.

It is important to note that by 1987, the full implications and widespread acceptance of H. pylori as the primary cause of peptic ulcers had not yet occurred. The paradigm shift would take several more years of research and clinical trials to solidify. Therefore, while psychological stress was still seen as the leading cause by many, the bacterial hypothesis had emerged as a powerful and increasingly credible alternative, laying the groundwork for a revolution in understanding and treating peptic ulcer disease.

### Comments on above:

The LLM seems a bit wishy-washy here with its answer, claiming psychological stress was still a leading cause in 1987, but the bacterial hypothesis was a credible alternative. It seemed to be leaning more towards stress here, so that is the result that was marked, but it seems not completely sure. Even though the cutoff date is beyond the date of the landmark publication, **all references are still beyond the cutoff date**. Also, some references are repeated.

1. The first reference: “A Novel Psychopathological Model Explains the Pathogenesis of Gastric Ulcers." is a **2022 article**.
2. Reference 2: Britannica. "Robin Warren." is an online encyclopedia Britannica article about Robin Warren (so **post-1987**).
3. Reference 3: Practo Consult is an online consulting site that talks about peptic ulcers (so **post-1987**).
4. Reference 4: "Stress and peptic ulcer: Life beyond Helicobacter." is a **1998 article** about stress and peptic ulcer
5. Reference 5: "Barry Marshall: Curing Peptic Ulcers." is an online post about Warren and Marshall and ends with them winning the nobel prize in 2005 (so is **post-1987**)
6. Reference 6: "Barry J. Marshall - Nobel Lecture." is the lecture from Marshall for his nobel prize in **2005**.
7. Reference 7: "Barry Marshall." Wikipedia. Is the Barry Marshall wikipedia article, so **post-1987**.
8. Reference 8: is the same as Reference 2, so **post 1987**.
9. Reference 9: is the same as Reference 5.
10. Reference 10: The University of Western Australia. "Vale Robin Warren." is a UWA article about Robin Warren after his death **in 2024**.

Also note that while everything has links, there are no proper citations.

### DeepSeek response

Based on information available up to 1987, the prevailing medical opinion largely favored psychological stress as the primary cause of peptic ulcers, though some early research suggested a possible bacterial component. Here’s the evidence:

**Hypothesis 1: Psychological Stress as the Cause (Dominant View Before 1987)**

Most medical textbooks and research before the mid-1980s attributed peptic ulcers to stress, lifestyle factors (e.g., spicy foods, smoking), and excess stomach acid. Key references:

- Schwartz’s dictum (1910): "No acid, no ulcer" was a widely accepted principle, emphasizing gastric acid as the main culprit.

- Selye’s stress theory (1936): Hans Selye’s work on stress and disease influenced the belief that psychological stress contributed to ulcers by increasing acid secretion.

- Medical textbooks (pre-1987): Standard references like Harrison’s Principles of Internal Medicine (e.g., 1983 edition) described stress and hyperacidity as major factors.

**Hypothesis 2: Bacterial Infection (Emerging but Controversial Before 1987)**

The bacterial theory (later linked to Helicobacter pylori) was proposed in the early 1980s but faced skepticism:

- Warren & Marshall (1983, 1984): Robin Warren and Barry Marshall first observed spiral bacteria in ulcer patients and suggested a link, but their findings were initially dismissed.

- The Lancet (1983): Warren’s initial observations.

- The Lancet (1984): Marshall’s self-experiment (ingesting H. pylori and developing gastritis).

- Resistance to the theory: Many gastroenterologists rejected the idea, citing lack of widespread acceptance and the dominance of the stress-acid model.

**Conclusion for 1987 Context**

Before 1987, psychological stress was the more widely accepted explanation for peptic ulcers, though bacterial infection was an emerging (but not yet mainstream) hypothesis. The H. pylori theory gained definitive support only in the 1990s after further research.

Would you like more details on any of these references?

**Comment:**

I then ask for the LLM to provide the full references.

Certainly! Below are the full references for the sources mentioned, based on information available up to 1987 regarding peptic ulcer disease:

---

1. Psychological Stress as the Dominant Theory (Pre-1987)

Schwartz’s Dictum ("No Acid, No Ulcer")

1- Schwartz, K. (1910). Über penetrierende Magen- und Jejunalgeschwüre. [On penetrating gastric and jejunal ulcers]. Beiträge zur Klinischen Chirurgie, 67, 96–128.

- This early 20th-century work established the role of gastric acid in ulcer formation, leading to the famous medical axiom.

Selye’s Stress Theory

2- Selye, H. (1936). A syndrome produced by diverse nocuous agents. Nature, 138(3479), 32.

- Introduced the concept of stress as a biological response affecting disease, later applied to peptic ulcers.

3- Selye, H. (1950). The Physiology and Pathology of Exposure to Stress. Montreal: Acta Inc.

- Expanded on stress-induced gastrointestinal damage, influencing ulcer theories for decades.

Medical Textbook References (Pre-1987)

4- Braunwald, E., Isselbacher, K. J., Petersdorf, R. G., Wilson, J. D., Martin, J. B., & Fauci, A. S. (Eds.). (1983). Harrison’s Principles of Internal Medicine (10th ed.). McGraw-Hill.

- Standard medical reference that emphasized stress, acid hypersecretion, and lifestyle factors in ulcer pathogenesis.

5- Sleisenger, M. H., & Fordtran, J. S. (Eds.). (1983). Gastrointestinal Disease: Pathophysiology, Diagnosis, Management (3rd ed.). W.B. Saunders.

- Another major gastroenterology textbook that downplayed bacterial causes in favor of acid and stress.

---

2. Emerging Bacterial Theory (Pre-1987)

Warren & Marshall’s Early Work on H. pylori

6- Warren, J. R. (1983). Unidentified curved bacilli on gastric epithelium in active chronic gastritis. The Lancet, 1(8336), 1273–1275.

- First published observation of spiral bacteria in ulcer patients (later named Helicobacter pylori).

7- Marshall, B. J., & Warren, J. R. (1984). Unidentified curved bacilli in the stomach of patients with gastritis and peptic ulceration. The Lancet, 1(8390), 1311–1315.

- Key paper linking the bacteria to gastritis and ulcers.

8- Marshall, B. J. (1985). Attempt to fulfill Koch’s postulates for pyloric Campylobacter. Medical Journal of Australia, 142(8), 436–439.

- Marshall’s famous self-experiment (drinking H. pylori culture) proving causation.

Skepticism and Resistance

9- Editorial. (1985). Campylobacter pylori: Is peptic disease an infectious disease? The Lancet, 2(8468), 1125–1126.

- Reflects the initial dismissal of the bacterial theory by many gastroenterologists.

**Conclusion for 1987 Context**

**Before 1987, stress and acid were the mainstream explanations**, supported by decades of medical literature. The bacterial hypothesis (H. pylori) was new, controversial, and not yet widely accepted, though Warren and Marshall’s work laid the foundation for a paradigm shift in the 1990s.

Would you like access to any of these papers or further details on specific claims?

### Comments on above:

Even though the date here is set beyond the landmark paper, the LLM still finds psychological stress to be the more likely cause. Additionally many references are incorrect, not relevant, or unverifiable.

1. Schwartz, K. "Beitrage uber penetrierende Magen-and Jejunal-geschwure/Klin." Chirurg 57 (1910): 96-128.: **This article is unverifiable as I can not access it anywhere, although the citation is available from google scholar**. Furthermore, it seems some summaries/ reviews of peptic ulcer mention it, so it probably is a legitimate source but I am guessing the LLM found it via another review paper since the original article can’t be accessed.
2. Selye, H. (1936). Is a real article but it has nothing to do with peptic ulcers or anything gastric related, only that severe injuries in rats show a typical “shock” syndrome”. Therefore it is **not particularly relevant**.
3. Selye, H. (1950). The Physiology and Pathology of Exposure to Stress. Montreal: Acta Inc. is a real article but while it mentions a 1948 book about how to deal with stress and some mention of the adreno gland and its removal, it does not mention anything to do with gastric function and is **irrelevant.**
4. References **4 & 5** are textbooks but are unverifiable due to lack of page numbers or chapters associated, and cannot be readily accessed (may need to purchase them, if they are even still available). Thus the **citations are incomplete.**
5. Reference 6: Warren, J. R. (1983). Is the landmark paper about finding bacteria in a stomach with gastritis, but should be Warren and Marshall- so **citation is incorrect.**
6. Reference 7 is the same as reference 6 above, but the date is 1984 and it should be 1983, so is **also incorrect**: ​​Warren JR, Marshall B. Unidentified curved bacilli on gastric epithelium in active chronic gastritis. Lancet. 1983 Jun 4;1(8336):1273-5. PMID: 6134060.
7. Reference 8: Marshall, B. J. (1985). Is Marshall’s famous paper when he does the self-experiment: **this citation is correct and relevant.**
8. Reference 9 **does not exist.**

### Perplexity

**Evaluation of Competing Hypotheses for the Cause of Peptic Ulcer (Pre-1987 Evidence)**

**Overview**

This report evaluates whether, based on information available up to 1987, peptic ulcers are more likely caused by psychological stress or by bacterial infection. All statements are supported by references to sources reflecting the state of knowledge before or during 1987.

1**. Psychological Stress as a Cause**

- **Prevailing Theory:** For much of the 20th century, psychological stress was widely believed to be a primary cause of peptic ulcers. This view was supported by epidemiological observations linking stressful events, occupations, and life circumstances to higher ulcer incidence[1](https://www.mentalhealthjournal.org/articles/painting-a-complete-picture-of-the-pathogenesis-of-peptic-ulcers.html).
- **Supporting Evidence:** Studies from the 1950s through the early 1980s reported correlations between stressful life events and the onset of ulcer symptoms[1](https://www.mentalhealthjournal.org/articles/painting-a-complete-picture-of-the-pathogenesis-of-peptic-ulcers.html). For example, a 1951 survey found higher ulcer rates in stressful occupations and lower rates among agricultural workers, and other studies noted increased ulcer incidence after major social upheavals[1](https://www.mentalhealthjournal.org/articles/painting-a-complete-picture-of-the-pathogenesis-of-peptic-ulcers.html).
- **Limitations:** Despite these associations, the exact causal mechanisms were not established, and the stress theory could not fully explain all characteristics of peptic ulcer disease. By the early 1980s, the role of stress was increasingly questioned, especially as alternative explanations emerged[1](https://www.mentalhealthjournal.org/articles/painting-a-complete-picture-of-the-pathogenesis-of-peptic-ulcers.html).

2. **Bacterial Infection as a Cause**

- **Emergence of Bacterial Theory:** In 1983, Dr. J. Robin Warren and Dr. Barry Marshall in Australia reported finding a previously unidentified bacterium (later named *Helicobacter pylori*) in the stomachs of patients with gastritis and peptic ulcers[2](https://web-archive.southampton.ac.uk/cogprints.org/677/1/ulcers.htm)[3](http://cogsci.uwaterloo.ca/Articles/Pages/Ulcers.one.html)[4](https://www.jyi.org/2005-february/2005/2/9/delayed-gratification-why-it-took-everybody-so-long-to-acknowledge-that-bacteria-cause-ulcers).
- **Key Findings Before 1987:**
  - Warren and Marshall observed that these spiral bacteria were present in nearly all patients with active chronic gastritis, duodenal ulcer, or gastric ulcer[2](https://web-archive.southampton.ac.uk/cogprints.org/677/1/ulcers.htm)[3](http://cogsci.uwaterloo.ca/Articles/Pages/Ulcers.one.html).
  - Their 1984 publication in *The Lancet* suggested a probable etiological role for these bacteria in peptic ulceration, though they acknowledged that cause-and-effect was not yet definitively proven[2](https://web-archive.southampton.ac.uk/cogprints.org/677/1/ulcers.htm)[3](http://cogsci.uwaterloo.ca/Articles/Pages/Ulcers.one.html).
  - Early experiments, including self-infection by Marshall, demonstrated that the bacteria could induce gastritis, and associations with ulcers were strong, though not every infected individual developed ulcers[2](https://web-archive.southampton.ac.uk/cogprints.org/677/1/ulcers.htm)[3](http://cogsci.uwaterloo.ca/Articles/Pages/Ulcers.one.html).
- **Medical Community Response:** The bacterial hypothesis was initially met with skepticism, as it contradicted the prevailing view that the stomach was too acidic for bacteria to survive and that ulcers were mainly due to stress or excess acid[2](https://web-archive.southampton.ac.uk/cogprints.org/677/1/ulcers.htm)[3](http://cogsci.uwaterloo.ca/Articles/Pages/Ulcers.one.html)[4](https://www.jyi.org/2005-february/2005/2/9/delayed-gratification-why-it-took-everybody-so-long-to-acknowledge-that-bacteria-cause-ulcers). However, by 1987, accumulating evidence was shifting opinion toward a bacterial cause[5](https://en.wikipedia.org/wiki/Timeline_of_peptic_ulcer_disease_and_Helicobacter_pylori)[2](https://web-archive.southampton.ac.uk/cogprints.org/677/1/ulcers.htm)[3](http://cogsci.uwaterloo.ca/Articles/Pages/Ulcers.one.html).

3. Comparative Evaluation (Pre-1987)

| **Hypothesis** | **Supporting Evidence (Pre-1987)** | **Limitations (Pre-1987)** |
| --- | --- | --- |
| Psychological Stress | Epidemiological correlations between stress and ulcer incidence; widely accepted theory for decades[1](https://www.mentalhealthjournal.org/articles/painting-a-complete-picture-of-the-pathogenesis-of-peptic-ulcers.html). | No established causal mechanism; could not explain all cases; theory increasingly questioned by early 1980s[1](https://www.mentalhealthjournal.org/articles/painting-a-complete-picture-of-the-pathogenesis-of-peptic-ulcers.html). |
| Bacterial Infection | Discovery of *H. pylori* in ulcer patients; strong associations in clinical studies; early experimental support[2](https://web-archive.southampton.ac.uk/cogprints.org/677/1/ulcers.htm)[3](http://cogsci.uwaterloo.ca/Articles/Pages/Ulcers.one.html). | Causality not yet fully proven for ulcers (only for gastritis); initial skepticism from medical community[2](https://web-archive.southampton.ac.uk/cogprints.org/677/1/ulcers.htm)[3](http://cogsci.uwaterloo.ca/Articles/Pages/Ulcers.one.html)[4](https://www.jyi.org/2005-february/2005/2/9/delayed-gratification-why-it-took-everybody-so-long-to-acknowledge-that-bacteria-cause-ulcers). |

4. **Conclusion**

As of 1987, the hypothesis that peptic ulcers are caused by psychological stress was widely accepted but increasingly questioned due to lack of definitive causal evidence[1](https://www.mentalhealthjournal.org/articles/painting-a-complete-picture-of-the-pathogenesis-of-peptic-ulcers.html). The bacterial infection hypothesis, specifically involving *Helicobacter pylori*, had strong and growing empirical support by 1987, including clinical associations and experimental findings, though full causality for ulcers (as opposed to gastritis) was not yet universally accepted[2](https://web-archive.southampton.ac.uk/cogprints.org/677/1/ulcers.htm)[3](http://cogsci.uwaterloo.ca/Articles/Pages/Ulcers.one.html)[4](https://www.jyi.org/2005-february/2005/2/9/delayed-gratification-why-it-took-everybody-so-long-to-acknowledge-that-bacteria-cause-ulcers). Thus, by 1987, it was becoming more likely—based on emerging scientific evidence—that peptic ulcers were caused by a bacterial infection rather than by psychological stress[2](https://web-archive.southampton.ac.uk/cogprints.org/677/1/ulcers.htm)[3](http://cogsci.uwaterloo.ca/Articles/Pages/Ulcers.one.html)[4](https://www.jyi.org/2005-february/2005/2/9/delayed-gratification-why-it-took-everybody-so-long-to-acknowledge-that-bacteria-cause-ulcers).

**References**

1. <https://www.mentalhealthjournal.org/articles/painting-a-complete-picture-of-the-pathogenesis-of-peptic-ulcers.html>
2. <https://web-archive.southampton.ac.uk/cogprints.org/677/1/ulcers.htm>
3. <http://cogsci.uwaterloo.ca/Articles/Pages/Ulcers.one.html>
4. <https://www.jyi.org/2005-february/2005/2/9/delayed-gratification-why-it-took-everybody-so-long-to-acknowledge-that-bacteria-cause-ulcers>
5. <https://en.wikipedia.org/wiki/Timeline_of_peptic_ulcer_disease_and_Helicobacter_pylori>
6. <https://blogs.the-hospitalist.org/content/stress-independently-predicts-peptic-ulcers>
7. <https://pmc.ncbi.nlm.nih.gov/articles/PMC3952291/>
8. <https://journals.lww.com/md-journal/fulltext/2017/08250/the_association_between_peptic_ulcer_diseases_and.26.aspx>
9. <https://histmodbiomed.history.qmul.ac.uk/sites/default/files/44836.pdf>
10. <https://www.thelancet.com/journals/lancet/article/PIIS0140-6736(05)67587-3/fulltext>
11. <https://pubmed.ncbi.nlm.nih.gov/25111233/>
12. <https://pmc.ncbi.nlm.nih.gov/articles/PMC3613748/>
13. <https://www.nytimes.com/2024/06/17/well/mind/stress-ulcers-anxiety.html>
14. <https://www.sciencedirect.com/science/article/abs/pii/0022399987900535>
15. <https://pmc.ncbi.nlm.nih.gov/articles/PMC5126869/>
16. <https://www.mentalhealthjournal.org/articles/a-novel-psychopathological-model-explains-the-pathogenesis-of-gastric-ulcers.pdf>
17. <https://pmc.ncbi.nlm.nih.gov/articles/PMC6139371/>
18. <https://www.ncbi.nlm.nih.gov/books/NBK2432/>

### Comments on above

The LLM is correct in finding bacterial infection to be more likely in 1987. Despite the cut-off date being after the landmark paper, **all but one of the references are after the cut-off date**.

1. Reference 1 is an article from **2022**.
2. Reference 2 is a **1997** webpage about ulcers and bacteria.
3. Reference 3 is the **same as Reference 2.**
4. Reference 4 is a **2005 blog post**
5. Reference 5 is a wikipedia article about peptic ulcer disease, thus **beyond the cut-off date**.
6. Reference 6 is a **2019** blog news article about peptic ulcers.
7. Reference 7 is a **2014** article about the overview of the history of peptic ulcers.
8. Reference 8 is a **2017** study about peptic ulcers and mental health.
9. Reference 9 is a transcript of a seminar held in **2000**.
10. Reference 10 is a **2005** article about Barry and Marshall winning the Nobel prize.
11. Reference 11 is a **2015** article linking stress and ulcers.
12. Reference 12 is a **2012** article.
13. Reference 13 is a **2024** NY Times article.
14. Reference 14 is a 1987 article about psychological stress and ulcers, so it **is within the correct time period and legitimate**.
15. Reference 15 is a **2016** study.
16. Reference 16 is an article from **2022** linking stress and ulcers.
17. Reference 17 is a **2018** article.
18. Reference 18 is a book chapter written by Marshall in **2001**.

### Chat GPT 5 Response

**Question:**

Please evaluate the following competing hypotheses using only information from 1987 or before. Please provide references showing where you got this information. Here are the hypotheses: "Is it more likely that peptic ulcer is caused by psychological stress or by a bacterial infection?"

**ChatGPT said:**

Great question — and to make sure I’m following your instructions: I’ll only consider sources published **1987 or earlier**, and I’ll weigh the two competing hypotheses:

1. **Peptic ulcer is caused by psychological stress.**
2. **Peptic ulcer is caused by a bacterial infection.**

**Background up to 1987**

**Stress Hypothesis (Traditional View)**

- For most of the 20th century, peptic ulcer disease (PUD) was commonly attributed to **psychological stress**, diet, and excess gastric acid.
- **Wolf & Wolff (1943)**: In their classic monograph *Human Gastric Function: An Experimental Study of a Man and His Stomach*, they argued that emotions and stress influenced gastric secretion and ulcer development.
- Clinical teaching throughout the 1950s–1970s emphasized the “Type A personality” and stressful life events as ulcer triggers (e.g., **Almy 1959, Gastroenterology**).
- Reviews in the 1970s (e.g., **Weiss & English, 1970, *Psychosomatic Medicine* Handbook**) reinforced stress as an important factor, though evidence was mostly correlational.

**Bacterial Hypothesis (Emerging View)**

- **1979–1982 (Australia)**: Robin Warren, a pathologist in Perth, observed spiral bacteria in gastric biopsies from gastritis and ulcer patients (**Warren 1983, *Lancet* correspondence**).
- **Marshall & Warren (1984, *Lancet*)**: Reported isolation of *Campylobacter-like organisms* (later named *Helicobacter pylori*) from gastric mucosa, strongly associated with active chronic gastritis.
- **Marshall et al. (1985, *Med J Aust*)**: Suggested a causal link between *H. pylori* infection and peptic ulceration. Marshall even performed a self-experiment, swallowing cultured bacteria and developing gastritis (**Marshall et al., 1985, *Aust N Z J Med***).
- By **1986–87**, controlled studies (e.g., **Langenberg et al., 1986, *Gastroenterology***; **Blaser, 1987, *Rev Infect Dis*** review) showed very high prevalence of *H. pylori* in duodenal ulcer patients.

**Evaluation by 1987**

- **Stress hypothesis**: Still widely taught, but lacked solid mechanistic or prospective evidence. Most studies were observational or based on physiological correlations (e.g., acid secretion during stress).
- **Bacterial hypothesis**: By 1987, growing experimental and epidemiological evidence supported *H. pylori* as a primary cause of gastritis and a major factor in peptic ulcer disease. Antibiotic treatment trials were not yet widespread, but the association was strong and reproducible.

**Thus, as of 1987, it was becoming more likely that peptic ulcer was caused by a bacterial infection (*H. pylori*) rather than by psychological stress, although the stress model was still entrenched in medical teaching.**

**References (≤1987)**

1. Wolf S, Wolff HG. *Human Gastric Function: An Experimental Study of a Man and His Stomach.* New York: Oxford Univ Press, 1943.
2. Almy TP. “Experimental studies on the relationship between emotion and the function of the human alimentary tract.” *Gastroenterology*. 1959;37:429–450.
3. Weiss SM, English JT. *Handbook of Psychosomatic Medicine.* St. Louis: Mosby, 1970.
4. Warren JR. “Unidentified curved bacilli on gastric epithelium in active chronic gastritis.” *Lancet*. 1983;1:1273–1275.
5. Marshall BJ, Warren JR. “Unidentified curved bacilli in the stomach of patients with gastritis and peptic ulceration.” *Lancet*. 1984;1:1311–1315.
6. Marshall BJ, Armstrong JA, McGechie DB, Glancy RJ. “Attempt to fulfil Koch’s postulates for pyloric Campylobacter.” *Med J Aust*. 1985;142:436–439.
7. Marshall BJ, Royce H, Annear DI, et al. “Original isolation of Campylobacter pyloridis from human gastric mucosa.” *Aust N Z J Med*. 1985;15:709–718.
8. Langenberg ML, Tytgat GN, Schipper ME, et al. “Campylobacter-like organisms in the stomach of patients and healthy individuals.” *Gastroenterology*. 1986;91(3):579–589.
9. Blaser MJ. “Campylobacter pyloridis and the etiology of gastroduodenal disease.” *Rev Infect Dis*. 1987;9(3):447–461.

### Comments on above:

While the LLM gets the correct hypothesis for 1987 (bacteria), many references are incorrect or not found. For references:

1. Wolf & Wolff **citation exists** but can’t find the actual article (not available online).
2. Almy TP. “Experimental studies on the relationship between emotion and the function of the human alimentary tract.” Gastroenterology. 1959;37:429–450. **Does not exist.** There is an article written by Almy TP in 1951 in a different journal called “Experimental studies on the irritable colon”. This is the closest I found.
3. Weiss SM, English JT. Handbook of Psychosomatic Medicine. St. Louis: Mosby, 1970. **Does not exist.**
4. Reference 4 (Warren): The first Warren citation is mostly correct, but **fails to cite Marshall as a co-author.** The citation should be: Warren, J. Robin, and Barry Marshall. "Unidentified curved bacilli on gastric epithelium in active chronic gastritis." The lancet 321.8336 (1983): 1273-1275.
5. Reference 5 is the same as reference 4, except that it correctly cites Marshall, but **incorrectly has the wrong date and volume/page numbers**.
6. Marshall reference 6 **is correct.**
7. Marshall reference 7 **does not have the journal and date correct**. It should be: Marshall, B. J., Royce, H., Annear, D. I., Goodwin, C. S., Pearman, J. W., Warren, R., & Armstrong, J. A. (1984). Original isolation of Campylobacter pyloridis from human gastric mucosa. *Microbios Letters*, *25*(98), 83-88.
8. For the Langenberg reference 8, the year and journal **are incorrect**. It should be: Langenberg, M. L., Tytgat, G. N. J., Schipper, M. E. I., Rietra, P. J. G. M., Zanen, H. C., Burnett, R. A., ... & Fricker, C. R. (1984). Campylobacter-uke organisms in the stomach of patients and healthy individuals. *The Lancet*, *323*(8390), 1348-1349.
9. Finally the Blaser MJ **paper does not exist**. There is a paper from 1987 where Blaser is the second author, but the title, authors, journal, volume and pages would all be incorrect. Perez-Perez GI, Blaser MJ. Conservation and diversity of Campylobacter pyloridis major antigens. Infect Immun. 1987 May;55(5):1256-63. doi: 10.1128/iai.55.5.1256-1263.1987. PMID: 3552997; PMCID: PMC260499.

## Robin results (using Crow)

Here we are testing an agentic system to see how well it answers the hypothesis question of cervical cancer before and after the study about HPV causing cervical cancer came out. Thus we ask the same question for different years 1979-1984.

### Results from Crow, 1979:

**Question:**

In 1979, was it more likely that cervical cancer was caused by herpes simplex virus (HSV) or human papillomavirus (HPV)? Please only use references from 1979 and before.

**Results:**

Pre‐1979 research focused predominantly on herpes simplex virus (HSV), particularly HSV‐2, as a potential cause of cervical cancer. Multiple studies noted that women with cervical cancer exhibited higher HSV‐2 antibody levels and, in some cases, even detectable viral DNA in tumor specimens, suggesting an association between HSV‐2 infection and subsequent cervical neoplasia (1.1, 2.1). Investigations during this period frequently explored seroepidemiologic evidence to support the idea that prior HSV infections could predispose individuals to malignant cervical lesions (1.2, 3.1).

In contrast, none of the cited studies mention human papillomavirus (HPV) in connection with cervical cancer. The absence of HPV in these discussions indicates that before 1979, HPV was not recognized or investigated as a possible etiologic factor in cervical carcinogenesis (4.1, 4.2). Researchers of that era were primarily concerned with the oncogenic potential of HSV, and the epidemiologic evidence at the time was interpreted to favor HSV as a candidate virus in the development of cervical cancer (3.2, 1.3).

Scientific consensus at the time, based on available evidence, leaned toward considering HSV—especially HSV‐2—as a likely causal agent in cervical cancer, while HPV was not part of contemporaneous etiological paradigms.

**Cervical cancer in 1979 was more likely viewed as caused by HSV rather than HPV.** The prevailing data from that period supported an association between HSV infection and cervical cancer, with HPV remaining unrecognized as a causative factor.

paper search

query: cervical cancer herpes simplex virus causation 1979 or before|cervical cancer human papillomavirus causation 1979 or before|cervical cancer HSV etiology 1979 or before|cervical cancer HPV etiology 1979 or before, max_year: 1979

1. The association of herpesvirus type 2 and carcinoma of the uterine cervix. WILLIAM E. RAWLS, W. A. F. TOMPKINS, JOSEPH L. MELNICK. American journal of epidemiology, Apr 1969

DOMAIN LEADING

Citations 378

1. Cytology and histopathology of cervical herpes simplex infection. Zuher M. Naib, Andre J. Nahmias, William E. Josey. Cancer, Jun 1966

DOMAIN LEADING

Citations 264

1. Association of Herpes simplex virus (HSV) with cervical cancer by lymphocyte reactivity with HSV-1 and HSV-2 antigens. JERRY W. SMITH, JOSE E. TORRES, NELSON D. HOLMQUIST. American journal of epidemiology, Jul 1979

DOMAIN LEADING

Citations 18

1. Antibodies to Herpesvirus hominis types 1 and 2 in humans. II. Women with cervical cancer. ANDRE J. NAHMIAS, WILLIAM E. JOSEY, ZUHER M. NAIB, CAROLYN F. LUCE, BEVERLY A. GUEST. American journal of epidemiology, May 1970

DOMAIN LEADING

Citations 323

1. Some aspects of the role of viruses in cancer. C W Potter. Postgraduate Medical Journal, Jan 1979

PEER REVIEWED

Citations 9

1. Human papillomavirus testing using existing nucleic acid testing platforms to screen women for cervical cancer: implementation studies from five sub-Saharan African countries. Jessica Joseph, Owen Demke, Lola Ameyan, Joseph Bitilinyu-Bango, Blandine Bourgoin, Mamadou Diop, Babacar Guèye, Jibrin Kama, Marvin Lubega, Bernard Madzima, Tatenda Maparo, Tasimba Mhizha, Andrew Musoke, Susan Nabadda, Twambilire Phiri, Timothy Tchereni, Owens Wiwa, Karen Hariharan, Trevor Peter, Shaukat Khan. BMJ Open, Dec 2022

PEER REVIEWED

Citations 8

1. Vooraf* A. Van Wyk. In die Skriflig/In Luce Verbi, Jun 1979

0

1. Long‐term follow‐up studies on herpes simplex antibodies in the course of cervical cancer. II. Antibodies to surface antigen of herpes simplex virus infected cells. Brith Christenson, Åke Espmark. International Journal of Cancer, Mar 1976

DOMAIN LEADING

Citations 36

1. Herpes simplex viruses 1 and 2--basic and clinical aspects. André J. Nahmias, Bodil Norrild. Disease-a-month : DM, Jun 1979

Citations 36

1. Seroepidemiologic studies of Herpesvirus type 2 and carcinoma of the cervix. III. Houston, Texas. Ervin Adam, Raymond H. Kaufman, Joseph L. Melnick, Allan H. Levy, William E. Rawls. American Journal of Epidemiology, Nov 1972

DOMAIN LEADING

Citations 9

**gather evidence** 7 out of 10 papers deemed relevant

question: In 1979 or before, was cervical cancer more likely considered to be caused by herpes simplex virus (HSV) or human papillomavirus (HPV)?

Added 19 pieces of evidence. Best evidence(s) for the current question:

- The role of herpes simplex virus type 2 (HSV-2) in cervical cancer was a subject of investigation in the 1970s. Various studies provided epidemiological evidence of HSV-2 infection in patients with dysplasia and carcinoma of the cervix, revealing a significant prevalence of the virus in biopsy specimens. However, there remains contention regarding HSV-2 as a primary cause of cervical cancer, with arguments highlighting that promiscuity may confound findings linking the virus to the malignancy. Furthermore, the oncogenic potential of herpesvirus in other species has drawn attention to possible contributions to cervical cancer. In contrast, the excerpt does not mention human papillomavirus (HPV), thus indicating that the focus during that time leaned more towards HSV, but significant debate and uncertainty still existed about its direct causative role.

- The excerpt discusses the potential association of the herpes simplex virus type 2 (HSV-2) with cervical cancer, mentioning studies that identify HSV-2 DNA in cervical tumor cells and the frequency of neutralizing antibodies in sera from patients with cervical cancer. However, it also acknowledges conflicting reports and the need for further studies to confirm the role of HSV-2. The excerpt does not mention human papillomavirus (HPV), suggesting that in 1979, HSV-2 was under investigation as a potential factor in cervical cancer but HPV was not addressed.

- The excerpt discusses various studies and findings regarding the association of herpes simplex virus (HSV) with cervical cancer. It highlights the exploration of lymphocyte reactivity and cell-mediated immunity concerning HSV and its potential link to cervical cancer. Specific references are made to research on the immune response to HSV antigens in women diagnosed with cervical cancer, suggesting a focus on HSV’s role in cancer development, although the specific mentions do not compare it directly with HPV.

- The excerpt discusses a study that suggests a possible association between herpesvirus type 2 (HSV-2) and cervical cancer. It notes that women with cervical cancer had a higher prevalence of antibodies to HSV-2 compared to control women, indicating that earlier infections with HSV-2 might be linked to the subsequent development of cervical cancer. This implies that there was a recognition of an association between HSV-2 and cervical cancer, at least in the context of the studies discussed around that time. Although this specific excerpt does not mention human papillomavirus (HPV), the focus on herpesvirus suggests that HSV-2 was studied in relation to cervical cancer, which could indicate a consideration of HSV in the etiology of cervical cancer prior to 1980.

- The provided excerpts from C W Potter's article mention key studies concerning the association of cervical cancer with infectious agents. Specifically, there is a reference to a study concerning the role of herpes simplex virus type 2 in cervical carcinogenesis, suggesting that HSV was considered relevant in discussions of cervical cancer etiology during that period. There are no direct mentions of human papillomavirus (HPV) in the provided excerpts, which may imply that discussions regarding HPV in the context of cervical cancer were not as prevalent or documented in the literature referenced by Potter by the year 1979.

### Comments on above:

Here the agent correctly ascertained that HSV-2 was thought to be a causal agent in 1979, and the explanation is reasonable. Despite being more reliable on references, agentic systems can still mess up citations. Here one paper is beyond the cutoff date (Reference 6 published 2022), and one paper is irrelevant (Reference 7 is in Afrikaans and translation is just a biography of someone).

### Results from Crow, 1980:

**Question:**

In 1980, was it more likely that cervical cancer was caused by herpes simplex virus (HSV) or human papillomavirus (HPV)? Please only use references from 1980 and before.

**Results:**

By 1980, the preponderance of evidence from cytological and epidemiological studies suggested that human papillomavirus (HPV) was more likely the primary causative agent in cervical cancer than herpes simplex virus (HSV). Research such as that by Coleman 1979 demonstrated that HPV infection was characterized by distinct cellular changes—most notably koilocytosis—that preceded the formation of clinically detectable lesions, indicating an early and pivotal role in cervical neoplasia (1.1, 1.2). In contrast, several investigations into HSV-2 from the mid‐1970s reported an association between HSV antibodies and cervical cancer, and noted the detection of viral components in tumor cells (2.1, 2.2, 2.3, 2.4). However, these studies consistently emphasized that, despite suggestive epidemiological correlations, the causal link for HSV was less definitive and required further investigation. As a result, the consensus by 1980 favored HPV over HSV as the more plausible etiologic factor, with HPV being recognized as instrumental in the early development of lesions that could progress to malignancy.

**Cervical cancer in 1980 was more likely caused by human papillomavirus than by herpes simplex virus.** The accumulated evidence from that period supports a primary role for HPV in cervical carcinogenesis.

**Paper search:**

query: cervical cancer cause herpes simplex virus OR HSV OR human papillomavirus OR HPV, min_year: 1900, max_year: 1980

1. The association of Herpes simplex virus and cervical cancer: a review. John W. Simon. Gynecologic oncology, Feb 1976

DOMAIN LEADING

Citations 15

1. A review of primate herpes viruses. K McCarthy, F A Tosolini. Proceedings of the Royal Society of Medicine, Feb 1975

Citations 38

1. Human Papillomaviruses and Neoplasia. MA Lutzner. The Lancet, Aug 1983

HIGHEST QUALITY

0

1. Cytological diagnosis of virus-infected cells in Papanicolaou smears and its application in clinical practice. D V Coleman. Journal of Clinical Pathology, Oct 1979

PEER REVIEWED

Citations 22

1. Microinvasive carcinoma of the cervix.* Edward W. Savage. Obstetrics and gynecology annual, Jun 1972

Citations 43

Evidence/ Reasoning:

**gather evidence**

question: In 1980, was it more likely that cervical cancer was caused by herpes simplex virus (HSV) or human papillomavirus (HPV)?

Added 8 pieces of evidence. Best evidence(s) for the current question:

- The excerpt discusses a potential etiological link between Herpes Simplex Virus Type 2 (HSV-2) and cervical cancer, presenting evidence from various studies indicating that women with cervical cancer or its precursors show a higher prevalence of antibodies against HSV-2 compared to controls. It also references epidemiological studies that suggest a sexual transmission relationship with cervical cancer, indicating that HSV-2 may be a significant factor in the disease's development. Although it does not directly compare this relationship with that of human papillomavirus (HPV), it highlights that significant evidence was gathered supporting HSV-2 as a possible cause for cervical cancer by the time of the article's publication in 1976.

- The excerpt discusses the association between herpes simplex virus type 2 (HSV-2) and cervical cancer, providing evidence that supports the idea that HSV-2 infection can induce carcinoma. It notes that significant evidence includes the detection of HSV-2 antigens in cervical carcinoma cells, as well as the isolation of viral genome components in tumors. This suggests a strong correlation between HSV-2 and cervical cancer, indicating that by 1980, HSV-2 was a recognized potential cause of cervical cancer. However, it lacks specific information regarding human papillomavirus (HPV) in this context, which limits comprehensive comparisons between the two viruses' roles in cervical cancer causation.

- The excerpt discusses the relationship between herpes simplex virus (HSV) and cervical cancer, suggesting that HSV may play a role in the development of the disease. It presents three possible explanations for the observed differences in antibody prevalence between patients and controls, focusing on the potential interactions of HSV with cervical cells and other factors such as sexual behavior. The text notes that some studies indicate a high prevalence of HSV antibodies among cervical cancer patients compared to controls but does not mention human papillomavirus (HPV) specifically. There is evidence discussed regarding the infection rates of HSV and its potential carcinogenic effects, indicating that the virus may not act only as a secondary invader but could influence cancer development more directly. Thus, the connection between HSV and cervical cancer in 1980 appears significant, though the excerpt does not provide comparable information on HPV.

- In the context of cervical cancer etiology around 1980, human papillomavirus (HPV) was increasingly recognized as a significant causative factor, with research emphasizing its association with cervical and other cancers. Evidence from various studies highlighted the immunological and genetic relationships among papillomaviruses, suggesting a distinct role for HPV in oncogenesis. Comparatively, herpes simplex virus (HSV) was not as prominently linked to cervical cancer in the existing research or discourse, indicating that HPV was viewed more convincingly as a contributor to cervical malignancy during that time period.

- The excerpt discusses the relationship between human papillomavirus (HPV) and cervical lesions. It highlights that papilloma viruses are associated with benign tumors, and koilocytosis, indicative of HPV infection, may represent an early stage before clinically detectable lesions form. Moreover, it points out potential issues in diagnosing premalignant cervical lesions as they can be confused with lesions arising from HPV infection, suggesting a significant link between HPV and conditions that could lead to cervical cancer.

### Comments on above:

Here the agent says that HPV is more likely to cause cervical cancer, although in 1980 this was before the landmark paper and should be more likely caused by HSV-2, or at least roughly 50/50. It is interesting because the LLM says that the Coleman paper (Reference 4) discusses that "papilloma viruses are associated with benign tumors.. and cervical lesions", and this is the reason for saying HPV is more likely. The Coleman paper is about identifying the different types of viruses (HSV, HPV, etc) by looking at infected cells and mentions that HPV is associated with these cervical lesions, but does not imply or show causality of cervical cancer. There are also several papers in the references that associate HSV-2 with cervical cancer, so if the LLM is basing its decision on association, it shouldn’t choose HPV over HSV.

Additionally, Reference 3 (Lutzner, “Human Papillomaviruses and Neoplasia”), which is cited for linking HPV to cervical cancer, doesn't exist. There is a review by Lutzner in 1983 on human papillomaviruses that describes the history of HPV and the different types of HPV, but it is in Arch Dermatology journal. There is also a Lutzner, M. A. "Human Papillomaviruses and Neoplasia." JAMA 76 (1921): 440-42., but the actual title in the archives is “[The Etiology Of Common Warts and their production in the Second Generation](https://jamanetwork-com.ezproxy.library.wisc.edu/journals/jama/fullarticle/226573)”. Finally there is a Lutzner paper in the Lancet in 1983, but it is entitled: “Detection of Human Papillomavirus type 5 DNA in skin cancers of an immunosuppressed renal allograft recipient”. It seems that this reference is made up. Taken together, it seems like the agent is using its training data (which of course is after 1980) to come up with the answer of HPV, while claiming to cite papers prior to 1980.

### Results from Crow, 1981:

**Question:**

In 1981, was it more likely that cervical cancer was caused by herpes simplex virus (HSV) or human papillomavirus (HPV)? Please only use references from 1981 and before.

**Results:**

Prior to and during 1981, the majority of available evidence pointed to herpes simplex virus type 2 as the primary candidate implicated in cervical carcinogenesis. Several studies documented a significantly higher prevalence of HSV-2 antibodies among cervical cancer patients compared to controls, and investigators demonstrated that HSV-2 met many of the proposed criteria for being a causal agent based on seroepidemiological findings and, in some cases, in vitro transformation experiments (1.1, 1.2, 2.1). Although a few reports acknowledged that cervical cancer might also be associated with other sexually transmitted agents, including human papillomavirus, these mentions were ancillary and lacked the direct supporting evidence that characterized the HSV-2 hypothesis (2.2, 3.1).

Moreover, the research focus of that period was strongly oriented toward establishing a link between HSV-2 infection and subsequent cervical dysplasia and carcinoma, with multiple studies reinforcing this association through immune response assessments and epidemiologic correlations (4.1, 5.1). In contrast, there was comparatively little direct evidence from 1981 or earlier implicating human papillomavirus in the etiology of cervical cancer (6.1).

By 1981, the prevailing view was that **herpes simplex virus, particularly HSV-2, was the more likely causative agent of cervical cancer.** In the absence of robust data supporting HPV, HSV-2 was considered the primary candidate responsible for cervical carcinogenesis

**References:**

paper search

query: cervical cancer cause HSV OR herpes simplex virus, max_year: 1981

1. Cytology and histopathology of cervical herpes simplex infection. Zuher M. Naib, Andre J. Nahmias, William E. Josey. Cancer, Jun 1966

DOMAIN LEADING

Citations 264

1. Herpes simplex virus and cancer of the cervix. G. Plummer, J.G. Masterson. American journal of obstetrics and gynecology, Aug 1971

HIGHEST QUALITY

Citations 57

1. The association of Herpes simplex virus and cervical cancer: a review. John W. Simon. Gynecologic oncology, Feb 1976.

DOMAIN LEADING

Citations 15

1. Herpes simplex virus type 2 and cervical cancer. F. Rapp. Current problems in cancer, Sep 1981

PEER REVIEWED

Citations 15

1. Herpes genitalis and its relationship to cervical cancer. R. H. Kaufman, W. E. Rawls. CA: A Cancer Journal for Clinicians, Aug 1974

DOMAIN LEADING

Citations 13

1. Herpes simplex virus and carcinoma of the cervix. Lise Thiry. European journal of cancer, Oct 1976

DOMAIN LEADING

Citations 9

1. Herpes Simplex Virus Type 2 and Human Cervical Cancer: Relationship Between Cellular and Humoral Immune Assays for the Detection of Previous Infection2, 3. Jerry W. Smith, Jose E. Torres, Nelson D. Holmquist. JNCI: Journal of the National Cancer Institute, May 1981

Citations 12

**02 Paper search:**

1. Human papillomavirus and cancer of the uterine cervix. Alexander Meisels, Carol Morin. Gynecologic oncology, Sep 1981

DOMAIN LEADING

Citations 270

1. Genital cancer and viruses. Fred Rapp, Frank J. Jenkins. Gynecologic oncology, Sep 1981

DOMAIN LEADING

Citations 25

1. Association of human papillomavirus with neoplastic processes in the genital tract of four women with impaired immunity. Siamak Shokri-Tabibzadeh, L. G. Koss, John J. Molnar, Seymour L. Romney. Gynecologic Oncology, Sep 1981

DOMAIN LEADING

Citations 62

1. Papillomavirus and Cutaneous Malignancy. Mitchell E. Bender, Franklin Franklin. International Journal of Dermatology, Aug 1981.

PEER REVIEWED

Citations 22

1. Viruses and gynecologic cancers: Herpesvirus protein (ICP 10/AG‐4), a cervical tumor antigen that fulfills the criteria for a marker of carcinogenicity. Laure Aurelian, Irving I. Kessler, Neil B. Rosenshein, Gene Barbour. Cancer, Jul 1981

DOMAIN LEADING

Citations 61

1. Analysis of human cancers, normal tissues, and verruce plantares for DNA sequences of human papillomavirus types 1 and 2. Maurice Green, Gerard Orth, William S.M. Wold, Pat R. Sanders, Jesse K. Mackey, M. Favre, O. Croissant. Virology, Apr 1981

PEER REVIEWED

Citations 26

1. Immunoperoxidase localization of papillomavirus antigens in cervical dysplasia and vulvar condylomas. Robert J. Kurman, Kishor H. Shah, Wayne D. Lancaster, A.Bennett Jenson. American journal of obstetrics and gynecology, Aug 1981

HIGHEST QUALITY

Citations 204

**gather evidence**

question: Was cervical cancer believed to be caused by HSV or herpes simplex virus in the literature before and during 1981?

Added 25 pieces of evidence. Best evidence(s) for the current question:

- The literature indicates a hypothesis that cervical cancer may be induced by sexually transmitted HSV-2 infection, which is backed by compelling evidence. Studies have detected HSV-2 antigens in exfoliated cells from cervical carcinoma, suggesting a direct association between the virus and cancer. The findings provide support to earlier observations regarding the connection between herpes simplex virus and cervical cancer, setting the stage for further investigation into this relationship.

- In the literature before and during 1981, there was a noted association between HSV-2 (herpes simplex virus type 2) and squamous cancer of the cervix. Researchers argued that the evidence presented was compelling due to its consistency across different studies and disciplines. Moreover, the authors asserted that HSV-2 meets all the suggested criteria for establishing causality in the development of cervical cancer. This representation in the literature suggests a recognition of the potential role of HSV-2 in cervical cancer during that time period.

- The literature prior to and during 1981 suggested a potential relationship between Herpes Simplex Virus Type 2 (HSV-2) and cervical cancer, particularly squamous cell carcinoma. Multiple studies indicated that women diagnosed with cervical cancer or its precursors exhibited higher levels of antibody activity against HSV-2 compared to control groups. The concept that cervical cancer may be linked to a sexually transmitted agent was supported by epidemiological findings, establishing a connection between sexual activity and increased incidence of the disease. Evidence was further backed by seroepidemiological studies demonstrating a higher prevalence of HSV-2 antibodies in cervical cancer patients.

- The excerpt discusses the relationship between herpes simplex virus type 2 (HSV-2) and cervical cancer, highlighting that prior to and during 1981, the literature recognized HSV-2 as a significant risk factor for cervical cancer (CIS) and associated conditions. It synthesizes various studies which demonstrate the presence of antibodies related to HSV-2 in patients with cervical cancer, indicating a correlation between the infection and cancer progression. The evidence compiled shows that exposure to HSV-2 was more prevalent in cervical cancer cases compared to controls and that immune responses to the virus are indicative of the disease's progression. The authors conclude that the existence of viral proteins and the immune response are tied to cancer development, confirming the link between HSV-2 and cervical cancer in the literature of the time.

- The literature before and during 1981 shows various studies investigating the association between herpesviruses, particularly type 2 and cytomegalovirus, and cervical cancer. Notable studies, including seroepidemiologic works, indicated a potential link between herpesvirus infections and cervical malignancies. Cited works pointed to an association of herpesvirus antibodies with cervical cancer, including studies on carcinoma in situ, suggesting that the relationship between herpes, particularly HSV, and cervical cancer was a topic of investigation.

### Comments on Above:

In this case, the agent gives the correct response that in 1981 HSV-2 was more likely. The references are mostly correct except for Reference 1 (Naib et al.), the link doesn’t work, but it is a real and legitimate reference.

### Results from Crow, 1982:

**Question:**

In 1982, was it more likely that cervical cancer was caused by herpes simplex virus (HSV) or human papillomavirus (HPV)? Please only use references from 1982 and before.

**Results:**

Evidence available up to 1982 provided contrasting views regarding viral etiologies in cervical cancer. Studies by Reid et al. demonstrated a robust statistical association between subclinical human papillomavirus (HPV) infection and cervical neoplasia, with HPV markers present in approximately 91% of cancer cases versus 12.5% in controls, and these studies did not detect a comparable link with herpes simplex virus (HSV) (1.1, 1.2, 1.3, 1.4). In contrast, contemporaneous investigations by Aurelian et al. and Rapp and Jenkins reported serologic reactivity and transformation evidence for HSV-2 in cervical cancer, yet these findings were less consistently replicated and lacked the quantitative strength observed in HPV studies (2.1, 2.2, 2.3, 2.4, 2.5, 2.6, 3.1, 3.2, 3.3). Moreover, morphological analyses highlighted by CP Crum further emphasized the significance of HPV in the development of cervical neoplasia (4.1).

**HPV was considered the more likely causative agent of cervical cancer in 1982.** The predominance of epidemiologic evidence and high detection rates in cancer cases supported HPV over HSV.

**References:**

paper search

query: cervical cancer causes herpes simplex virus HPV, min_year: 1900, max_year: 1982

1. Viruses and gynecologic cancers: Herpesvirus protein (ICP 10/AG‐4), a cervical tumor antigen that fulfills the criteria for a marker of carcinogenicity. Laure Aurelian, Irving I. Kessler, Neil B. Rosenshein, Gene Barbour. Cancer, Jul 1981

DOMAIN LEADING

Citations 61

1. Viruses and squamous neoplasia of the lower female genital tract: an update. CP Crum. 1982

Citations 4

1. Genital warts and cervical cancer. I. Evidence of an association between subclinical papillomavirus infection and cervical malignancy. Richard Reid, C. Robert Stanhope, Barry R. Herschman, Earnest Booth, Garth D. Phibbs, Julian P. Smith. Cancer, Jul 1982

DOMAIN LEADING

Citations 313

1. Genital cancer and viruses. Fred Rapp, Frank J. Jenkins. Gynecologic oncology, Sep 1981

DOMAIN LEADING

Citations 25

1. Importance of the male factor in cancer of the cervix. D.C.G. Skegg, P.A. Corwin, Charlotte Paul, Richard Doll. The Lancet, Sep 1982

HIGHEST QUALITY

Citations 220

**Evidence/Reasoning:**

question: In 1982, was it more likely that cervical cancer was caused by herpes simplex virus (HSV) or human papillomavirus (HPV)?

Added 18 pieces of evidence. Best evidence(s) for the current question:

- The excerpt presents strong evidence suggesting that human papillomavirus (HPV) is significantly associated with cervical neoplasia, indicating a possible causal relationship. Statistical analyses reveal a highly significant association between subclinical HPV infection and cervical malignancy, with a large proportion of cervical cancer cases showing evidence of HPV infection. The persistence of HPV infection and its potential role in the progression to cervical neoplasia is highlighted, distinguishing HPV's impact on cervical cancer risk. In contrast, there is no mention of herpes simplex virus (HSV) in this context, implying that HPV is the virus more implicated in cervical cancer cases in 1982.

- The excerpt discusses the association between herpes simplex virus type 2 (HSV-2) and cervical cancer, emphasizing compelling evidence of causation. It states that there is a clear link established between HSV-2 and squamous cell carcinoma of the cervix, while no similar association has been observed with other viruses, including human papillomavirus (HPV). It is noted that the evidence for HSV-2's role in cervical cancer is robust and multi-faceted, fulfilling the criteria for causality, whereas HPV does not have the same level of demonstrated association with cervical cancer.

- The excerpt discusses the association between subclinical papillomavirus infection and cervical neoplasia, suggesting that there is a strong possibility of a causal relationship. It highlights the biologic plausibility of this connection, as HPV is known to cause persistent infections in the cervical transformation zone, which can lead to cervical cancer. In contrast, there is no mention of herpes simplex virus (HSV) in relation to cervical cancer within this context, indicating that during this time, HPV was more closely associated with cervical malignancy.

- The excerpt cites a study that identifies a significant statistical link between cervical neoplasia and subclinical papillomavirus infection (SPI), highlighting that 91% of women with cervical neoplasia had evidence of HPV infection, compared to only 12.5% of matched controls. The study argues that the strength and specificity of the relationship between HPV and cervical cancer suggests that HPV may be a precursor to cervical malignancy. Although herpes simplex virus (HSV2) is mentioned as a potentially oncogenic infection, the data presented strongly favors HPV as a more likely cause of cervical cancer.

- The study conducted by Reid et al. provides substantial evidence suggesting a strong association between subclinical papillomavirus infection (SPI) and cervical malignancy. The analysis revealed a significant difference in the prevalence of SPI between women with and without cervical neoplasia, indicating that HPV infection was much more common in those with cervical cancer. Specifically, 91% of women with cervical neoplasia tested positive for HPV, compared to only 12.5% of matched controls. The researchers employed a variety of analytical methods to minimize bias and corroborate their findings, which concluded that SPI is significantly related to cervical cancer, thereby suggesting that HPV is a contributing factor to the malignancy. In contrast, the study provided no evidence supporting a similar link between herpes simplex virus (HSV) and cervical cancer, implying that HPV is more likely associated with the disease than HSV.

### Comments on above:

Here the LLM claims HPV is the stronger hypothesis, but based on the papers it references, there are papers based on associations of HPV and cervical cancer and/ or lesions, as well as associations of HSV-2 with cervical cancer. Thus the evidence for each hypothetical cause is roughly equal. Additionally this is before the 1983 landmark paper that shows causality of HPV for cervical cancer. While most of the references are correct, the last reference (Skegg, 1982) is irrelevant.

### Results from Crow, 1983:

**Question:**

In 1983, was it more likely that cervical cancer was caused by herpes simplex virus (HSV) or human papillomavirus (HPV)? Please only use references from 1983 and before.

**Result:**

Evidence from studies published up to 1983 overwhelmingly indicates that human papillomavirus (HPV), not herpes simplex virus (HSV), is primarily associated with cervical cancer. Several investigations, including a pivotal 1982 study by Reid et al., demonstrated that a high percentage of women with cervical neoplasia had subclinical HPV infection, with HPV detected in 91% of cases compared to a much lower rate in controls (1.1, 1.2). Additional studies in the American Journal of Obstetrics and Gynecology provided corroborative findings, showing HPV’s strong association with cervical dysplasia and its potential to induce oncogenic changes in the cervical epithelium (2.1, 2.2). Lancet reports from 1983 further reinforced HPV’s role in the carcinogenic process by documenting distinct cellular alterations related to HPV infection while not emphasizing HSV as a primary factor (3.1, 3.2).

In contrast, earlier work by Simon in 1976 offered only circumstantial evidence linking HSV-2 to cervical cancer through the detection of viral antigens and DNA, but these results were inconsistent and did not establish a clear causal relationship (4.1, 4.2, 4.3). Later studies from the early eighties seldom highlighted HSV as a major etiologic factor, thereby supporting the contention that HPV was the predominant viral agent in cervical cancer (3.3, 1.3).

**Cervical cancer in the pre-1983 literature is more robustly linked to HPV than to HSV.** The evidence consistently demonstrates a strong association between HPV infection and cervical neoplasia, while the data for HSV remain inconclusive.

**References:**

paper search

query: cervical cancer herpes simplex virus causation OR cervical cancer HSV causation OR cervical cancer human papillomavirus causation OR cervical cancer HPV causation, max_year: 1983

1. Immunoperoxidase localization of papillomavirus antigens in cervical dysplasia and vulvar condylomas. Robert J. Kurman, Kishor H. Shah, Wayne D. Lancaster, A.Bennett Jenson. American journal of obstetrics and gynecology, Aug 1981

HIGHEST QUALITY

Citations 204

1. ON THE NATURE OF THE DEEP CELLULAR DISTURBANCES IN HUMAN-PAPILLOMA-VIRUS INFECTION OF THE SQUAMOUS CERVICAL EPITHELIUM. Stewart Fletcher, Mary Norval. The Lancet, Sep 1983

HIGHEST QUALITY

Citations 24

1. The association of Herpes simplex virus and cervical cancer: a review. John W. Simon. Gynecologic oncology, Feb 1976

DOMAIN LEADING

Citations 15

1. Genital warts and cervical cancer. I. Evidence of an association between subclinical papillomavirus infection and cervical malignancy. Richard Reid, C. Robert Stanhope, Barry R. Herschman, Earnest Booth, Garth D. Phibbs, Julian P. Smith. Cancer, Jul 1982

DOMAIN LEADING

Citations 313

**Evidence:**

question: In 1983, was cervical cancer more likely thought to be caused by herpes simplex virus (HSV) or by human papillomavirus (HPV)?

Added 13 pieces of evidence. Best evidence(s) for the current question:

- In the study from 1982, there is significant evidence supporting a causal association between human papillomavirus (HPV) infection and cervical neoplasia, including histologic evidence of subclinical HPV infection in a high percentage of subjects. The findings indicate that HPV may play a crucial role in the carcinogenic process associated with cervical cancer, with a mounting body of circumstantial evidence. Meanwhile, the study does not present strong evidence pointing to herpes simplex virus (HSV) as a prominent causative factor for cervical cancer, suggesting that by 1983 HPV was increasingly recognized as a primary concern regarding cervical malignancy. Overall, the results reinforce the suspicion of HPV's oncogenic potential while indicating limited retrospective studies on HSV's role in cervical cancer during that period.

- The excerpt discusses the association between herpes simplex virus type 2 (HSV-2) and cervical cancer, suggesting an etiologic relationship. It notes various studies indicating that women with cervical cancer or its precursors tend to have higher levels of antibodies against HSV-2 compared to controls. Additionally, it mentions evidence supporting HSV-2 as a significant factor tied to cervical malignancy, including the presence of HSV-2 antigens and DNA in neoplastic lesions. While the text primarily addresses HSV-2, it does not mention human papillomavirus (HPV), which was also a major focus in cervical cancer discussions in the 1980s.

- The excerpt discusses the relationship between cervical neoplasms and various viruses, particularly noting that HSV-2 DNA was often found absent in neoplasms associated with cervical carcinoma. It suggests that while herpes simplex virus (HSV-2) has been linked to cervical cancer through circumstantial evidence, it emphasizes a more clear common association of dysplasia with human papillomavirus (HPV) infection and its role in the transformation to dysplasia. This indicates that the earlier belief leans more towards HPV as a significant factor in cervical cancer, while the role of HSV-2 is less definitive based on the data mentioned in the text.

- The excerpt discusses the association between subclinical papillomavirus infection and cervical neoplasia, suggesting a potentially causal relationship. It highlights that HPV is recognized as a sexually transmitted oncogenic virus that leads to persistent subclinical infections in areas of the cervix. This implies that at least by 1982, HPV was front and center in the discussion regarding cervical cancer causation, despite also referencing other viral pathogens like cytomegalovirus. The evidence pointed toward HPV as a significant factor contributing to cervical cancer risk, whereas the role of herpes simplex virus (HSV) in this context is not emphasized or mentioned in the excerpt.

- The excerpt discusses the uncertainty surrounding the causes of cervical cancer in 1983, with particular reference to HPV and HSV. It notes that the causative role of HPV in cervical cancer is not definitively established, as HPV infection is rarely found as the sole cause of cervical malignancy. Additionally, there is skepticism explicitly mentioned regarding the evidence for a causal link between HSV-2 and cervical cancer, indicating that doubts persisted about both viruses' roles in the disease's etiology. This suggests that during this time, neither virus could be conclusively pointed to as the main cause of cervical cancer, highlighting the complexity of the disease's relationship with these infections.

### Comments on above:

Here the agent correctly states HPV to be the cause of cervical cancer. All the references are legitimate here, however based on these references it is unclear how the agent came up with HPV for its answer. It is interesting that it did not pull the landmark paper here. It seems like the main evidence is coming from the 1982 Reid paper (Reference 4), which suggests HPV could be involved in malignant cervical cancers, but does not show that with evidence as the landmark paper did. There is also the Kurman paper (Reference 1), which shows HPV antigens in cervical dysplasia, but this is not cancerous. The Fletcher paper (Reference 2) states HPV infection may be a cause of dysplasia, but is not certain, and again dysplasia is abnormal cells not full blown cancer. The other paper (Simon, Reference 3) associates HSV with cervical cancer. Thus based on these papers, evidence may slightly lean towards the cause being HPV, but not “overwhelmingly” as the agent states.

### Results from Crow, 1984:

**Question:**

In 1984, was it more likely that cervical cancer was caused by herpes simplex virus (HSV) or human papillomavirus (HPV)? Please only use references from 1984 and before.

**Result:**

Prior to 1984, early epidemiologic studies frequently observed that women with cervical cancer had higher HSV‐2 antibody titers, suggesting an association between HSV-2 and cervical carcinogenesis (1.1, 1.2). However, subsequent work revealed significant difficulties in reliably detecting HSV-specific DNA in cervical tumor samples, a shortcoming that undermined HSV’s candidacy as the direct causative agent (2.1, 2.2). Concurrently, investigations in the early 1980s increasingly demonstrated the presence of human papillomavirus (HPV) markers—viral particles, antigens, and nucleic acids—in cervical dysplasias and carcinomas, thereby establishing a stronger etiologic link for HPV (2.3, 3.1, 4.1). While some reports noted that HSV might have a contributory or ‘hit and run’ role, the overall body of evidence by that time was shifting markedly toward HPV as the central and more consistent factor in cervical neoplasia (5.1, 5.2).

In summary, by 1984 the preponderance of **evidence pointed to HPV rather than HSV as the more likely cause of cervical cancer.** HPV emerged as the principal etiologic agent reflected in improved detection methods and accumulating pathological data.

**References:**

paper search

query: cervical cancer herpes simplex virus human papillomavirus, min_year: 1900, max_year: 1984

1. On the nature of the deep cellular disturbances in human-papilloma-virus infection of the squamous cervical epithelium. Stewart Fletcher, Mary Norval The Lancet, Sep 1983

HIGHEST QUALITY

Citations 24

1. Human papillomavirus types 6 and 11 DNA sequences in genital and laryngeal papillomas and in some cervical cancers. L. Gissmann, LuTz WOLNIKt, H. Ikenberg, Ursula KOLDOVSKYt, Hans Georg, SCHNURCHt, Harald, Zur, Hausen. Proceedings of the National Academy of Sciences of the United States of America, **Dec 1982**

HIGHEST QUALITY

Citations 1218

1. The association of Herpes simplex virus and cervical cancer: a review. John W. Simon. Gynecologic oncology, Feb 1976

DOMAIN LEADING

Citations 15

1. Human papillomavirus and cancer of the uterine cervix. Alexander Meisels, Carol Morin. Gynecologic oncology, Sep 1981

DOMAIN LEADING

Citations 270

1. Genital warts and cervical cancer. I. Evidence of an association between subclinical papillomavirus infection and cervical malignancy. Richard Reid, C. Robert Stanhope, Barry R. Herschman, Earnest Booth, Garth D. Phibbs, Julian P. Smith. Cancer, Jul 1982

DOMAIN LEADING

Citations 313

1. Human genital cancer: synergism between two virus infections or synergism between a virus infection and initiating events? HZ Hausen. The Lancet, Dec 1982

HIGHEST QUALITY

Citations 700

1. HSV, CMV, and HPV in human neoplasia. James K. Mcdougall, Jay A. Nelson, David Myerson, Anna M. Beckmann, Denise A. Galloway. The Journal of investigative dermatology, Jun 1984

Citations 41

1. Clinical significance of human papillomavirus infection of the uterine cervix in the development of cervical intraepithelial neoplasia. D. Cheetham, J. Smith, C. Wilson, P. Munday, D. Coleman. Sexually Transmitted Infections, May 1984

PEER REVIEWED

Citations 18

1. Prospective study on the relationship between cervical neoplasia and herpes simplex type‐2 virus. II. Herpes simplex type‐2 antibody presence in sera taken at enrolment. V. Vonka, J. Kanaka, I. Hirsch, H. Závadová, M. Krčmář, A. Suchánková, D. Řezácová, J. Brouček, M. Press, E. Domorázková, B. Svoboda, A. Havránková, J. Jelinek

**Evidence/ Reasoning:**

question: In 1984 and before, was cervical cancer more likely thought to be caused by herpes simplex virus (HSV) or human papillomavirus (HPV)?

Added 16 pieces of evidence. Best evidence(s) for the current question:

- The excerpt discusses the association between Herpes Simplex Virus Type 2 (HSV-2) and cervical cancer, providing evidence that suggests a potential etiological relationship. Various studies indicated that women with cervical cancer or its precursors showed higher antibody activity against HSV-2 than matched controls. Furthermore, it highlights that research has accumulated evidence supporting the notion that HSV-2 could be the sexually transmitted agent responsible for cell transformation associated with cervical carcinoma. While the text does not mention human papillomavirus (HPV) in detail, it underscores the prominence of HSV-2 as a candidate linked to cervical cancer prior to 1984.

- The excerpt discusses the investigations into the causes of cervical cancer, highlighting the roles of both human papillomaviruses (HPV) and herpes simplex virus (HSV). Initially, there was considerable epidemiological evidence linking HSV with cervical cancer, as women with cervical cancer were found to have higher antibody titres to HSV-2 compared to controls. However, the lack of detectable HSV DNA in biopsy samples led researchers to explore other potential causing agents, notably HPV, which was hypothesized to be involved due to its presence in genital warts. Therefore, by 1984 and before, while there were indications of HSV's involvement, the growing emphasis on HPV as a causative factor became prevalent.

- The excerpt discusses the roles of herpes simplex virus (HSV) and human papillomavirus (HPV) in the development of cervical cancer. It indicates that there has been difficulty in detecting HSV-specific DNA in cervical cancer patients, suggesting that the role of HSV-2 as a causative agent has been undermined. In contrast, there is evidence of papillomavirus involvement, with numerous references to the detection of papillomavirus particles, antigens, and nucleic acids in cervical dysplasia and carcinomas. This indicates a stronger association between HPV and cervical cancer than HSV during the time period discussed.

- The excerpt discusses the role of various viruses, including herpes simplex virus (HSV) and human papillomavirus (HPV), in cervical dysplasia and cancer. It describes how HSV might act as a 'hit and run' initiator of neoplasia, while HPV is implicated in the transformation of cervical cells leading to dysplastic changes. There is a suggestion of synergistic effects between HPV and other carcinogenic factors, indicating that the understanding of cervical cancer etiology was evolving, highlighting the significant association with HPV but also recognizing the involvement of HSV.

- The excerpt discusses the association between herpes simplex virus (HSV) and cervical cancer, suggesting significant antibody prevalence among cancer patients. It presents multiple theories regarding the relationship between HSV and cervical cancer, including the possibility that HSV acts as a secondary invader or a direct carcinogen. The data indicated that individuals with invasive cervical carcinoma had higher rates of antibodies against HSV compared to matched controls, regardless of sexual promiscuity. Although the excerpt does not mention human papillomavirus (HPV), it highlights the importance of HSV in the context of cervical cancer prior to 1984, indicating that HSV was recognized as a significant factor, but also implying that other factors could contribute to the disease's etiology.

### Comments on above:

Here the agent correctly identifies HPV as the more likely cause. The papers referenced are mostly correct and the search includes the zur Hansen landmark paper which provides the main evidence for the HPV claim. The agent does give the incorrect date for the zur Hansen paper, saying it is from 1982 and it is actually from 1983.
